# Diretriz Brasileira de Atendimento à Dor Torácica na Unidade de Emergência – 2025

**DOI:** 10.36660/abc.20250620

**Published:** 2025-10-13

**Authors:** Pedro Gabriel Melo de Barros e Silva, Alexandre de Matos Soeiro, Carlos Eduardo Ornelas, Gilson Soares Feitosa-Filho, Renato D. Lopes, Danielli Oliveira da Costa Lino, Remo Holanda de Mendonça Furtado, Hélio Penna Guimarães, André Volschan, Bruno Ferraz de Oliveira Gomes, Carisi Anne Polanczyk, Carlos Eduardo Rochitte, Carlos Vicente Serrano, Cláudio Marcelo Bittencourt das Virgens, Claudio Tinoco Mesquita, Edgardo Jorge Menendez, Eduardo Leal Adam, Fabio Mastrocola, Fábio Serra Silveira, Felipe Souza Maia da Silva, Giovanni Possamai Dutra, Humberto Graner Moreira, Isly Maria Lucena de Barros, João Luiz Fernandes Petriz, José Roberto de Oliveira Silva, Julio Flavio Meirelles Marchini, Louis Nakayama Ohe, Ludhmila Abrahão Hajjar, Maria Camila Lunardi, Mucio Tavares de Oliveira, Nivaldo Menezes Filgueiras, Odilson Marcos Silvestre, Paolo Blanco Villela, Paulo Rogério Soares, Pedro Paulo Nogueres Sampaio, Renée Sarmento de Oliveira, Ronaldo de Souza Leão Lima, Sandro Pinelli Felicioni, Sergio Timerman, Tatiana de Carvalho Andreuci Torres Leal, Wilson Mathias

**Affiliations:** 1 Hospital do Coração São Paulo SP Brasil Hospital do Coração (Hcor), São Paulo, SP – Brasil; 2 Brazilian Clinical Research Institute São Paulo SP Brasil Brazilian Clinical Research Institute, São Paulo, SP – Brasil; 3 Centro Universitário São Camilo São Paulo SP Brasil Centro Universitário São Camilo, São Paulo, SP – Brasil; 4 Hospital das Clínicas da Faculdade de Medicina da Universidade de São Paulo Instituto do Coração São Paulo SP Brasil Instituto do Coração (Incor) do Hospital das Clínicas da Faculdade de Medicina da Universidade de São Paulo (HCFMUSP), São Paulo, SP – Brasil; 5 Hospital Felício Rocho Belo Horizonte MG Brasil Hospital Felício Rocho, Belo Horizonte, MG – Brasil; 6 Escola Bahiana de Medicina e Saúde Pública Salvador BA Brasil Escola Bahiana de Medicina e Saúde Pública, Salvador, BA – Brasil; 7 Hospital Santa Izabel Salvador BA Brasil Hospital Santa Izabel, Salvador, BA – Brasil; 8 Santa Casa de Misericórdia da Bahia Salvador BA Brasil Santa Casa de Misericórdia da Bahia, Salvador, BA – Brasil; 9 Duke University Medical Center Durham EUA Duke University Medical Center, Durham – EUA; 10 Hospital de Messejana Dr. Carlos Alberto Studart Gomes Fortaleza CE Brasil Hospital de Messejana Dr. Carlos Alberto Studart Gomes, Fortaleza, CE – Brasil; 11 Hospital Israelita Albert Einstein São Paulo SP Brasil Hospital Israelita Albert Einstein, São Paulo, SP – Brasil; 12 Universidade de São Paulo São Paulo SP Brasil Universidade de São Paulo (USP), São Paulo, SP – Brasil; 13 Hospital Pró-Cardíaco Rio de Janeiro RJ Brasil Hospital Pró-Cardíaco, Rio de Janeiro, RJ – Brasil; 14 Hospital Barra D’Or Rio de Janeiro RJ Brasil Hospital Barra D’Or, Rio de Janeiro, RJ – Brasil; 15 Universidade Federal do Rio de Janeiro Hospital Universitário Clementino Fraga Filho Rio de Janeiro RJ Brasil Hospital Universitário Clementino Fraga Filho, Universidade Federal do Rio de Janeiro (UFRJ), Rio de Janeiro, RJ – Brasil; 16 Hospital de Clínicas da Universidade Federal do Rio Grande do Sul Porto Alegre RS Brasil Hospital de Clínicas da Universidade Federal do Rio Grande do Sul (UFRS), Porto Alegre, RS – Brasil; 17 Universidade Federal da Bahia Hospital Universitário Professor Edgard Santos Salvador BA Brasil Hospital Universitário Professor Edgard Santos (HUPES), Universidade Federal da Bahia, Salvador, BA – Brasil; 18 Universidade Federal Fluminense Rio de Janeiro RJ Brasil Universidade Federal Fluminense (UFF), Rio de Janeiro, RJ – Brasil; 19 Hospital Churruca Buenos Aires Argentina Hospital Churruca, Buenos Aires – Argentina; 20 Adam Cardiologia Curitiba PR Brasil Adam Cardiologia, Curitiba, PR – Brasil; 21 Universidade Federal do Rio Grande do Norte Hospital Universitário Onofre Lopes Natal RN Brasil Hospital Universitário Onofre Lopes, Universidade Federal do Rio Grande do Norte (UFRN), Natal, RN – Brasil; 22 Clínica do Coração Aracaju SE Brasil Clínica do Coração, Aracaju, SE – Brasil; 23 Hospital Quinta D’Or Rio de Janeiro RJ Brasil Hospital Quinta D’Or, Rio de Janeiro, RJ – Brasil; 24 Universidade do Estado do Rio de Janeiro Hospital Universitário Pedro Ernesto Rio de Janeiro RJ Brasil Hospital Universitário Pedro Ernesto, Universidade do Estado do Rio de Janeiro (UERJ), Rio de Janeiro, RJ – Brasil; 25 Universidade Federal de Goiás Goiânia GO Brasil Universidade Federal de Goiás (UFG), Goiânia, GO – Brasil; 26 Pronto Socorro Cardiológico de Pernambuco Recife PE Brasil Pronto Socorro Cardiológico de Pernambuco (PROCAPE), Recife, PE – Brasil; 27 Universidade de Pernambuco Recife PE Brasil Universidade de Pernambuco (UPE), Recife, PE – Brasil; 28 Hospital Sírio Libanês São Paulo SP Brasil Hospital Sírio Libanês, São Paulo, SP – Brasil; 29 Instituto Dante Pazzanese de Cardiologia São Paulo SP Brasil Instituto Dante Pazzanese de Cardiologia, São Paulo, SP – Brasil; 30 Hospital das Clínicas da Faculdade de Medicina da Universidade de São Paulo São Paulo SP Brasil Hospital das Clínicas da Faculdade de Medicina da Universidade de São Paulo (HCFMUSP), São Paulo, SP – Brasil; 31 Universidade do Estado da Bahia Salvador BA Brasil Universidade do Estado da Bahia (UNEB), Salvador, BA – Brasil; 32 Clínica Silvestre Santé Rio Branco AC Brasil Clínica Silvestre Santé, Rio Branco, AC – Brasil; 33 Instituto do Coração Fundação Zerbini São Paulo SP Brasil Fundação Zerbini, Instituto do Coração (Incor), São Paulo, SP – Brasil; 34 Hospital Samaritano Rio de Janeiro RJ Brasil Hospital Samaritano, Rio de Janeiro, RJ – Brasil; 35 Universidade Federal do Rio de Janeiro Rio de Janeiro RJ Brasil Universidade Federal do Rio de Janeiro (UFRJ), Rio de Janeiro, RJ – Brasil; 36 Universidade Federal do Estado do Rio de Janeiro Rio de Janeiro RJ Brasil Universidade Federal do Estado do Rio de Janeiro (UNIRIO), Rio de Janeiro, RJ – Brasil; 37 Fonte Imagem Rio de Janeiro RJ Brasil Fonte Imagem, Rio de Janeiro, RJ – Brasil

**Table:** 

Diretriz Brasileira de Atendimento à Dor Torácica na Unidade de Emergência – 2025	
O relatório abaixo lista as declarações de interesse conforme relatadas à SBC pelos especialistas durante o período de desenvolvimento deste posicionamento, 2024/2025.	
Especialista	Tipo de relacionamento com a indústria
Alexandre de Matos Soeiro	Declaração financeira B - Financiamento de pesquisas sob sua responsabilidade direta/pessoal (direcionado ao departamento ou instituição) provenientes da indústria farmacêutica, de órteses, próteses, equipamentos e implantes, brasileiras ou estrangeiras: - Biomerieux.
André Volschan	Outros relacionamentos Participação societária de qualquer natureza e qualquer valor economicamente apreciável de empresas na área de saúde, de ensino ou em empresas concorrentes ou fornecedoras da SBC: - Sócio de Clínica Privada.
Bruno Ferraz de Oliveira Gomes	Nada a ser declarado
Carisi Anne Polanczyk	Nada a ser declarado
Carlos Eduardo Ornelas	Declaração financeira A - Pagamento de qualquer espécie e desde que economicamente apreciáveis, feitos a (i) você, (ii) ao seu cônjuge/ companheiro ou a qualquer outro membro que resida com você, (iii) a qualquer pessoa jurídica em que qualquer destes seja controlador, sócio, acionista ou participante, de forma direta ou indireta, recebimento por palestras, aulas, atuação como proctor de treinamentos, remunerações, honorários pagos por participações em conselhos consultivos, de investigadores, ou outros comitês, etc. Provenientes da indústria farmacêutica, de órteses, próteses, equipamentos e implantes, brasileiras ou estrangeiras: - Bayer: Firialta; Novartis: Sybrava; Chiesi: Trimbow. B - Financiamento de pesquisas sob sua responsabilidade direta/pessoal (direcionado ao departamento ou instituição) provenientes da indústria farmacêutica, de órteses, próteses, equipamentos e implantes, brasileiras ou estrangeiras: - Novartis: Sybrava; Daiichi Sankyo: ácido bempedóico. Outros relacionamentos Financiamento de atividades de educação médica continuada, incluindo viagens, hospedagens e inscrições para congressos e cursos, provenientes da indústria farmacêutica, de órteses, próteses, equipamentos e implantes, brasileiras ou estrangeiras: - Chiesi: Trimbow.
Carlos Eduardo Rochitte	Declaração financeira A - Pagamento de qualquer espécie e desde que economicamente apreciáveis, feitos a (i) você, (ii) ao seu cônjuge/ companheiro ou a qualquer outro membro que resida com você, (iii) a qualquer pessoa jurídica em que qualquer destes seja controlador, sócio, acionista ou participante, de forma direta ou indireta, recebimento por palestras, aulas, atuação como proctor de treinamentos, remunerações, honorários pagos por participações em conselhos consultivos, de investigadores, ou outros comitês, etc. Provenientes da indústria farmacêutica, de órteses, próteses, equipamentos e implantes, brasileiras ou estrangeiras: - Canon: Health Imaging Technology; GE: ressonância e tomografia cardiovascular.
Carlos Vicente Serrano Jr.	Nada a ser declarado
Cláudio Marcelo Bittencourt das Virgens	Nada a ser declarado
Claudio Tinoco Mesquita	Declaração financeira A - Pagamento de qualquer espécie e desde que economicamente apreciáveis, feitos a (i) você, (ii) ao seu cônjuge/ companheiro ou a qualquer outro membro que resida com você, (iii) a qualquer pessoa jurídica em que qualquer destes seja controlador, sócio, acionista ou participante, de forma direta ou indireta, recebimento por palestras, aulas, atuação como proctor de treinamentos, remunerações, honorários pagos por participações em conselhos consultivos, de investigadores, ou outros comitês, etc. Provenientes da indústria farmacêutica, de órteses, próteses, equipamentos e implantes, brasileiras ou estrangeiras: - Servier: doença coronariana; Novartis: Pluvicto; Pfizer: amiloidose. B - Financiamento de pesquisas sob sua responsabilidade direta/pessoal (direcionado ao departamento ou instituição) provenientes da indústria farmacêutica, de órteses, próteses, equipamentos e implantes, brasileiras ou estrangeiras: - AstraZeneca: amiloidose; Alnylam: amiloidose.
Danielli Oliveira da Costa Lino	Declaração financeira A - Pagamento de qualquer espécie e desde que economicamente apreciáveis, feitos a (i) você, (ii) ao seu cônjuge/ companheiro ou a qualquer outro membro que resida com você, (iii) a qualquer pessoa jurídica em que qualquer destes seja controlador, sócio, acionista ou participante, de forma direta ou indireta, recebimento por palestras, aulas, atuação como proctor de treinamentos, remunerações, honorários pagos por participações em conselhos consultivos, de investigadores, ou outros comitês, etc. Provenientes da indústria farmacêutica, de órteses, próteses, equipamentos e implantes, brasileiras ou estrangeiras: - Boehringer: Tenecteplase. Outros relacionamentos Financiamento de atividades de educação médica continuada, incluindo viagens, hospedagens e inscrições para congressos e cursos, provenientes da indústria farmacêutica, de órteses, próteses, equipamentos e implantes, brasileiras ou estrangeiras: - Boehringer: Tenecteplase.
Edgardo Jorge Menendez	Nada a ser declarado
Eduardo Leal Adam	Nada a ser declarado
Fabio Mastrocola	Declaração financeira A - Pagamento de qualquer espécie e desde que economicamente apreciáveis, feitos a (i) você, (ii) ao seu cônjuge/ companheiro ou a qualquer outro membro que resida com você, (iii) a qualquer pessoa jurídica em que qualquer destes seja controlador, sócio, acionista ou participante, de forma direta ou indireta, recebimento por palestras, aulas, atuação como proctor de treinamentos, remunerações, honorários pagos por participações em conselhos consultivos, de investigadores, ou outros comitês, etc. Provenientes da indústria farmacêutica, de órteses, próteses, equipamentos e implantes, brasileiras ou estrangeiras: - Novo Nordisk: semaglutida.
Fábio Serra Silveira	Nada a ser declarado
Felipe Souza Maia da Silva	Nada a ser declarado
Gilson Soares Feitosa-Filho	Declaração financeira B - Financiamento de pesquisas sob sua responsabilidade direta/pessoal (direcionado ao departamento ou instituição) provenientes da indústria farmacêutica, de órteses, próteses, equipamentos e implantes, brasileiras ou estrangeiras: - Amgen: Olpasiran; Idorsia: Selatogrel; Anthos: Abelacimabe; Jansen e Bayer: Milvexiana. Outros relacionamentos Financiamento de atividades de educação médica continuada, incluindo viagens, hospedagens e inscrições para congressos e cursos, provenientes da indústria farmacêutica, de órteses, próteses, equipamentos e implantes, brasileiras ou estrangeiras: - Servier/Trimetazidina: Inscrição em Congresso.
Giovanni Possamai Dutra	Nada a ser declarado
Hélio Penna Guimarães	Nada a ser declarado
Humberto Graner Moreira	Declaração financeira A - Pagamento de qualquer espécie e desde que economicamente apreciáveis, feitos a (i) você, (ii) ao seu cônjuge/ companheiro ou a qualquer outro membro que resida com você, (iii) a qualquer pessoa jurídica em que qualquer destes seja controlador, sócio, acionista ou participante, de forma direta ou indireta, recebimento por palestras, aulas, atuação como proctor de treinamentos, remunerações, honorários pagos por participações em conselhos consultivos, de investigadores, ou outros comitês, etc. Provenientes da indústria farmacêutica, de órteses, próteses, equipamentos e implantes, brasileiras ou estrangeiras: - Pfizer: amiloidose e imunizações; Novo Nordisk: obesidade e inflamação; Novartis: dislipidemia; Daichii-Sankyo: dislipidemia; Bayer: cardio-oncologia. Outros relacionamentos Financiamento de atividades de educação médica continuada, incluindo viagens, hospedagens e inscrições para congressos e cursos, provenientes da indústria farmacêutica, de órteses, próteses, equipamentos e implantes, brasileiras ou estrangeiras: - Novo Nordisk: obesidade.
Isly Maria Lucena de Barros	Nada a ser declarado
João Luiz Fernandes Petriz	Declaração financeira A - Pagamento de qualquer espécie e desde que economicamente apreciáveis, feitos a (i) você, (ii) ao seu cônjuge/ companheiro ou a qualquer outro membro que resida com você, (iii) a qualquer pessoa jurídica em que qualquer destes seja controlador, sócio, acionista ou participante, de forma direta ou indireta, recebimento por palestras, aulas, atuação como proctor de treinamentos, remunerações, honorários pagos por participações em conselhos consultivos, de investigadores, ou outros comitês, etc. Provenientes da indústria farmacêutica, de órteses, próteses, equipamentos e implantes, brasileiras ou estrangeiras: - Servier: Vastarel.
José Roberto de Oliveira Silva Filho	Declaração financeira A - Pagamento de qualquer espécie e desde que economicamente apreciáveis, feitos a (i) você, (ii) ao seu cônjuge/ companheiro ou a qualquer outro membro que resida com você, (iii) a qualquer pessoa jurídica em que qualquer destes seja controlador, sócio, acionista ou participante, de forma direta ou indireta, recebimento por palestras, aulas, atuação como proctor de treinamentos, remunerações, honorários pagos por participações em conselhos consultivos, de investigadores, ou outros comitês, etc. Provenientes da indústria farmacêutica, de órteses, próteses, equipamentos e implantes, brasileiras ou estrangeiras: - Chiesi: Trimbow; GSK: Vacinas; Libbs: ticagrelor, ramipril e candesartana; Biolab: vasopressina. Outros relacionamentos Financiamento de atividades de educação médica continuada, incluindo viagens, hospedagens e inscrições para congressos e cursos, provenientes da indústria farmacêutica, de órteses, próteses, equipamentos e implantes, brasileiras ou estrangeiras: - Daiichi Sankyo: prasugrel.
Julio Flavio Meirelles Marchini	Nada a ser declarado
Louis Nakayama Ohe	Declaração financeira A - Pagamento de qualquer espécie e desde que economicamente apreciáveis, feitos a (i) você, (ii) ao seu cônjuge/ companheiro ou a qualquer outro membro que resida com você, (iii) a qualquer pessoa jurídica em que qualquer destes seja controlador, sócio, acionista ou participante, de forma direta ou indireta, recebimento por palestras, aulas, atuação como proctor de treinamentos, remunerações, honorários pagos por participações em conselhos consultivos, de investigadores, ou outros comitês, etc. Provenientes da indústria farmacêutica, de órteses, próteses, equipamentos e implantes, brasileiras ou estrangeiras: - Abbott: Coroflow; BBraun: Sequent Please; Libbs: Artag; Meril: Stent.
Ludhmila Abrahão Hajjar	Nada a ser declarado
Maria Camila Lunardi	Nada a ser declarado
Mucio Tavares de Oliveira Junior	Declaração financeira A - Pagamento de qualquer espécie e desde que economicamente apreciáveis, feitos a (i) você, (ii) ao seu cônjuge/ companheiro ou a qualquer outro membro que resida com você, (iii) a qualquer pessoa jurídica em que qualquer destes seja controlador, sócio, acionista ou participante, de forma direta ou indireta, recebimento por palestras, aulas, atuação como proctor de treinamentos, remunerações, honorários pagos por participações em conselhos consultivos, de investigadores, ou outros comitês, etc. Provenientes da indústria farmacêutica, de órteses, próteses, equipamentos e implantes, brasileiras ou estrangeiras: - Sanofi-Pasteur: Efluelda/influenza; Torrent: Iccor/insuficiência cardíaca; Merck: Concor/insuficiência cardíaca; Biolab: Simdax/insuficiência cardíaca; GSK: Arexvy/vírus sincicial respiratório; Pfizer: Abrysvo/vírus sincicial respiratório; Ache: Edistride/insuficiência cardíaca. B - Financiamento de pesquisas sob sua responsabilidade direta/pessoal (direcionado ao departamento ou instituição) provenientes da indústria farmacêutica, de órteses, próteses, equipamentos e implantes, brasileiras ou estrangeiras: - Sanofi-Pasteur: Efluelda/Influenza.
Nivaldo Menezes Filgueiras Filho	Declaração financeira A - Pagamento de qualquer espécie e desde que economicamente apreciáveis, feitos a (i) você, (ii) ao seu cônjuge/ companheiro ou a qualquer outro membro que resida com você, (iii) a qualquer pessoa jurídica em que qualquer destes seja controlador, sócio, acionista ou participante, de forma direta ou indireta, recebimento por palestras, aulas, atuação como proctor de treinamentos, remunerações, honorários pagos por participações em conselhos consultivos, de investigadores, ou outros comitês, etc. Provenientes da indústria farmacêutica, de órteses, próteses, equipamentos e implantes, brasileiras ou estrangeiras: - AstraZeneca: Lokelma, Forxiga; Servier: Adesão/Acertanlo, Acertil; Boehringer: Jardiance. Outros relacionamentos Financiamento de atividades de educação médica continuada, incluindo viagens, hospedagens e inscrições para congressos e cursos, provenientes da indústria farmacêutica, de órteses, próteses, equipamentos e implantes, brasileiras ou estrangeiras: - Servier: Acertanlo; AstraZeneca: Lokelma.
Odilson Marcos Silvestre	Nada a ser declarado
Paolo Blanco Villela	Declaração financeira A - Pagamento de qualquer espécie e desde que economicamente apreciáveis, feitos a (i) você, (ii) ao seu cônjuge/ companheiro ou a qualquer outro membro que resida com você, (iii) a qualquer pessoa jurídica em que qualquer destes seja controlador, sócio, acionista ou participante, de forma direta ou indireta, recebimento por palestras, aulas, atuação como proctor de treinamentos, remunerações, honorários pagos por participações em conselhos consultivos, de investigadores, ou outros comitês, etc. Provenientes da indústria farmacêutica, de órteses, próteses, equipamentos e implantes, brasileiras ou estrangeiras: - FQM: Exforge HCT. Outros relacionamentos Financiamento de atividades de educação médica continuada, incluindo viagens, hospedagens e inscrições para congressos e cursos, provenientes da indústria farmacêutica, de órteses, próteses, equipamentos e implantes, brasileiras ou estrangeiras: - FQM: Flebon.
Paulo Rogério Soares	Declaração financeira A - Pagamento de qualquer espécie e desde que economicamente apreciáveis, feitos a (i) você, (ii) ao seu cônjuge/ companheiro ou a qualquer outro membro que resida com você, (iii) a qualquer pessoa jurídica em que qualquer destes seja controlador, sócio, acionista ou participante, de forma direta ou indireta, recebimento por palestras, aulas, atuação como proctor de treinamentos, remunerações, honorários pagos por participações em conselhos consultivos, de investigadores, ou outros comitês, etc. Provenientes da indústria farmacêutica, de órteses, próteses, equipamentos e implantes, brasileiras ou estrangeiras: - Merck: Glifage XR/ Concor; Libbs: programa de residentes; AstraZeneca: Forxiga. Outros relacionamentos Financiamento de atividades de educação médica continuada, incluindo viagens, hospedagens e inscrições para congressos e cursos, provenientes da indústria farmacêutica, de órteses, próteses, equipamentos e implantes, brasileiras ou estrangeiras: - AstraZeneca: Congresso Europeu de 2024.
Pedro Gabriel Melo de Barros e Silva	Declaração financeira B - Financiamento de pesquisas sob sua responsabilidade direta/pessoal (direcionado ao departamento ou instituição) provenientes da indústria farmacêutica, de órteses, próteses, equipamentos e implantes, brasileiras ou estrangeiras: - Bayer, Novartis, Idorsia, Roche Diagnostics.
Pedro Paulo Nogueres Sampaio	Outros relacionamentos Participação em comitês de compras de materiais ou fármacos em instituições de saúde ou funções assemelhadas: - Coordenador da Cardiologia do Hospital Samaritano.
Remo Holanda de Mendonça Furtado	Declaração financeira A - Pagamento de qualquer espécie e desde que economicamente apreciáveis, feitos a (i) você, (ii) ao seu cônjuge/ companheiro ou a qualquer outro membro que resida com você, (iii) a qualquer pessoa jurídica em que qualquer destes seja controlador, sócio, acionista ou participante, de forma direta ou indireta, recebimento por palestras, aulas, atuação como proctor de treinamentos, remunerações, honorários pagos por participações em conselhos consultivos, de investigadores, ou outros comitês, etc. Provenientes da indústria farmacêutica, de órteses, próteses, equipamentos e implantes, brasileiras ou estrangeiras: - AstraZeneca: Forxiga; Bayer: Firialta; Boehringer: Trayenta; Daiichi-Sankyo: Lixiana; Servier: Diamicron; Libbs: Artag; Novartis: Sybrava. B - Financiamento de pesquisas sob sua responsabilidade direta/pessoal (direcionado ao departamento ou instituição) provenientes da indústria farmacêutica, de órteses, próteses, equipamentos e implantes, brasileiras ou estrangeiras: - Aché: consultoria de protocolo; Libbs: consultoria de protocolo; AstraZeneca: Insuficiência cardiaca; Pfizer: fibrilação atrial; Regeneron: fibrilação atrial; Viatris: infarto do miocárdio; Brainfarma: analgesia; Bayer: câncer de próstata; Novartis: Insuficiência cardíaca; Roche: análise estatística. Outros relacionamentos Financiamento de atividades de educação médica continuada, incluindo viagens, hospedagens e inscrições para congressos e cursos, provenientes da indústria farmacêutica, de órteses, próteses, equipamentos e implantes, brasileiras ou estrangeiras: - Novartis: Sybrava.
Renato D. Lopes	Declaração financeira A - Pagamento de qualquer espécie e desde que economicamente apreciáveis, feitos a (i) você, (ii) ao seu cônjuge/ companheiro ou a qualquer outro membro que resida com você, (iii) a qualquer pessoa jurídica em que qualquer destes seja controlador, sócio, acionista ou participante, de forma direta ou indireta, recebimento por palestras, aulas, atuação como proctor de treinamentos, remunerações, honorários pagos por participações em conselhos consultivos, de investigadores, ou outros comitês, etc. Provenientes da indústria farmacêutica, de órteses, próteses, equipamentos e implantes, brasileiras ou estrangeiras: - Pfizer, Daiichi Sankyo, Novo Nordisk, Bayer, Boehringer Ingelheim, Bristol-Myers Squibb. B - Financiamento de pesquisas sob sua responsabilidade direta/pessoal (direcionado ao departamento ou instituição) provenientes da indústria farmacêutica, de órteses, próteses, equipamentos e implantes, brasileiras ou estrangeiras: - Amgen, Bristol-Myers Squibb, GlaxoSmithKline, Medtronic, Pfizer, Sanofi-Aventis. Outros relacionamentos Financiamento de atividades de educação médica continuada, incluindo viagens, hospedagens e inscrições para congressos e cursos, provenientes da indústria farmacêutica, de órteses, próteses, equipamentos e implantes, brasileiras ou estrangeiras: - Pfizer, Daiichi Sankyo, Novo Nordisk, Novartis.
Renée Sarmento de Oliveira	Declaração financeira A - Pagamento de qualquer espécie e desde que economicamente apreciáveis, feitos a (i) você, (ii) ao seu cônjuge/ companheiro ou a qualquer outro membro que resida com você, (iii) a qualquer pessoa jurídica em que qualquer destes seja controlador, sócio, acionista ou participante, de forma direta ou indireta, recebimento por palestras, aulas, atuação como proctor de treinamentos, remunerações, honorários pagos por participações em conselhos consultivos, de investigadores, ou outros comitês, etc. Provenientes da indústria farmacêutica, de órteses, próteses, equipamentos e implantes, brasileiras ou estrangeiras: - Novo Nordisk: palestra semaglutida. Outros relacionamentos Financiamento de atividades de educação médica continuada, incluindo viagens, hospedagens e inscrições para congressos e cursos, provenientes da indústria farmacêutica, de órteses, próteses, equipamentos e implantes, brasileiras ou estrangeiras: - Novo Nordisk: semaglutida (Wegovy).
Ronaldo de Souza Leão Lima	Outros relacionamentos Participação societária de qualquer natureza e qualquer valor economicamente apreciável de empresas na área de saúde, de ensino ou em empresas concorrentes ou fornecedoras da SBC: - Sócio da Fonte Imagem.
Sandro Pinelli Felicioni	Nada a ser declarado
Sergio Timerman	Nada a ser declarado
Tatiana de Carvalho Andreuci Torres Leal	Outros relacionamentos Financiamento de atividades de educação médica continuada, incluindo viagens, hospedagens e inscrições para congressos e cursos, provenientes da indústria farmacêutica, de órteses, próteses, equipamentos e implantes, brasileiras ou estrangeiras: - Eli Lilly: Mounjaro.
Wilson Mathias Junior	Nada a ser declarado

## Sumário

**Figura S1:**
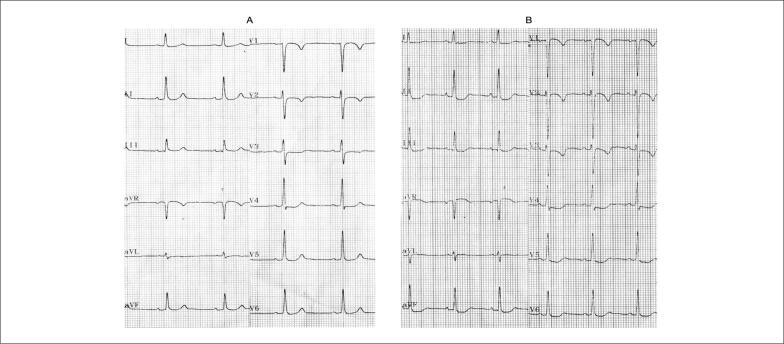
Mulher de 52 anos atendida com resolução da angina (duração de 15 minutos). A) ECG na admissão; B) ECG no momento de recorrência da angina evidenciando infradesnivelamento horizontal do segmento ST > 0,5 mm, em DII, DIII, aVF, V3 a V6. Diagnóstico de SCASSST.

**Figura S2:**
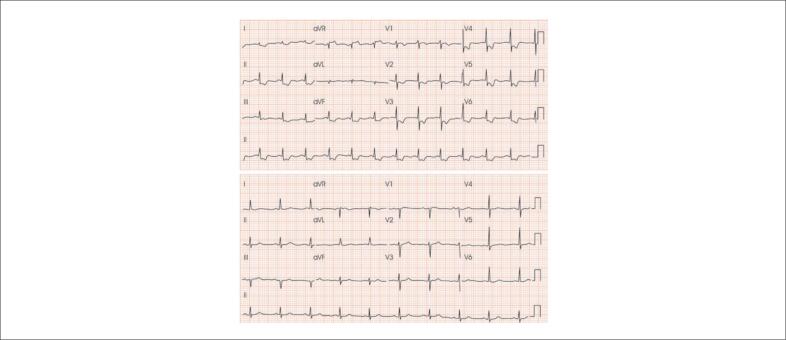
Homem de 73 anos, tabagista e dislipidêmico com dor precordial em queimação de início há 30 minutos. Observar a presença de infradesnível do segmento ST de morfologia descendente, de forma difusa associada a supradesnível em aVR > 0,5 mm. Após administração de nitrato sublingual, houve melhora da dor e reversão das alterações no segmento ST. Este padrão é associado à obstrução de TCE e/ou doença de três vasos. Paciente deve ser submetido precocemente à cinecoronariografia.

**Figura S3:**
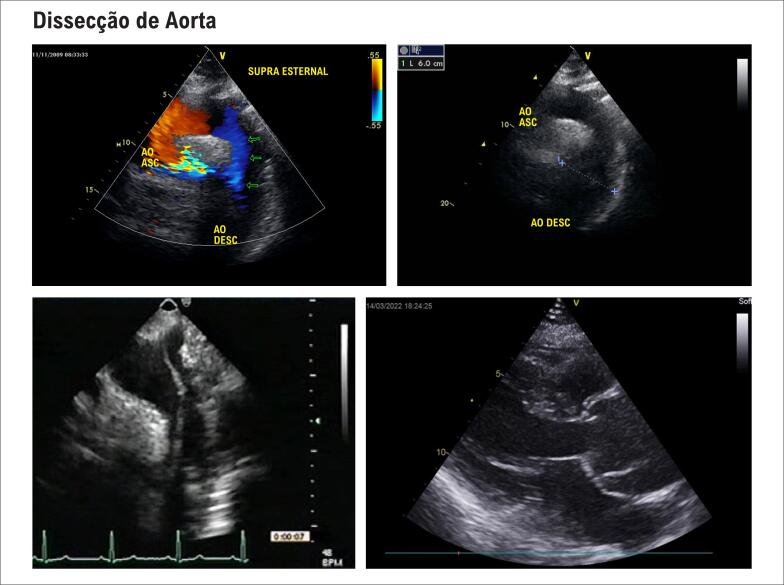
Exemplos de dissecção de aorta. A: observe que o fluxo (em azul) segue pela luz verdadeira. B: observe o aneurisma de aorta descendente. C: linha de dissecção visualizada. D: dissecção e aneurisma em aorta ascendente no corte paraesternal longitudinal.

**Figura S4:**
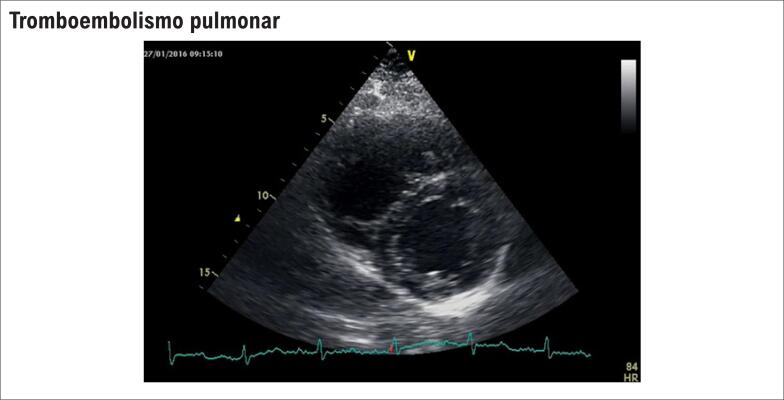
Sobrecarga pressórica do VD sobre o VE. Repare que o VD tem diâmetros aumentados e empurra o septo interventricular em direção ao VE.

**Figura S5:**
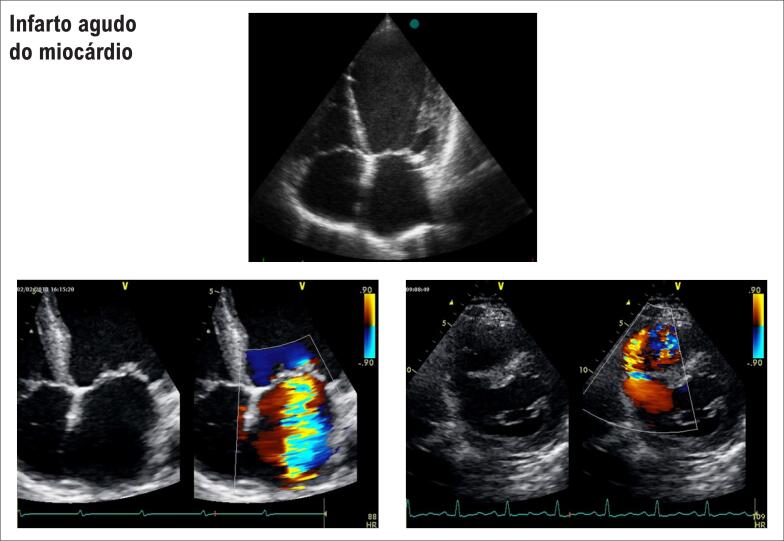
Exemplos de alterações provocadas pelo infarto. A: importante dilatação do VE com contraste espontâneo em seu interior; B: insuficiência mitral grave secundário à isquemia de músculo papilar; C: comunicação interventricular pós-IAM. Em vermelho, observe o fluxo do VE para o VD.

**Figura S6:**
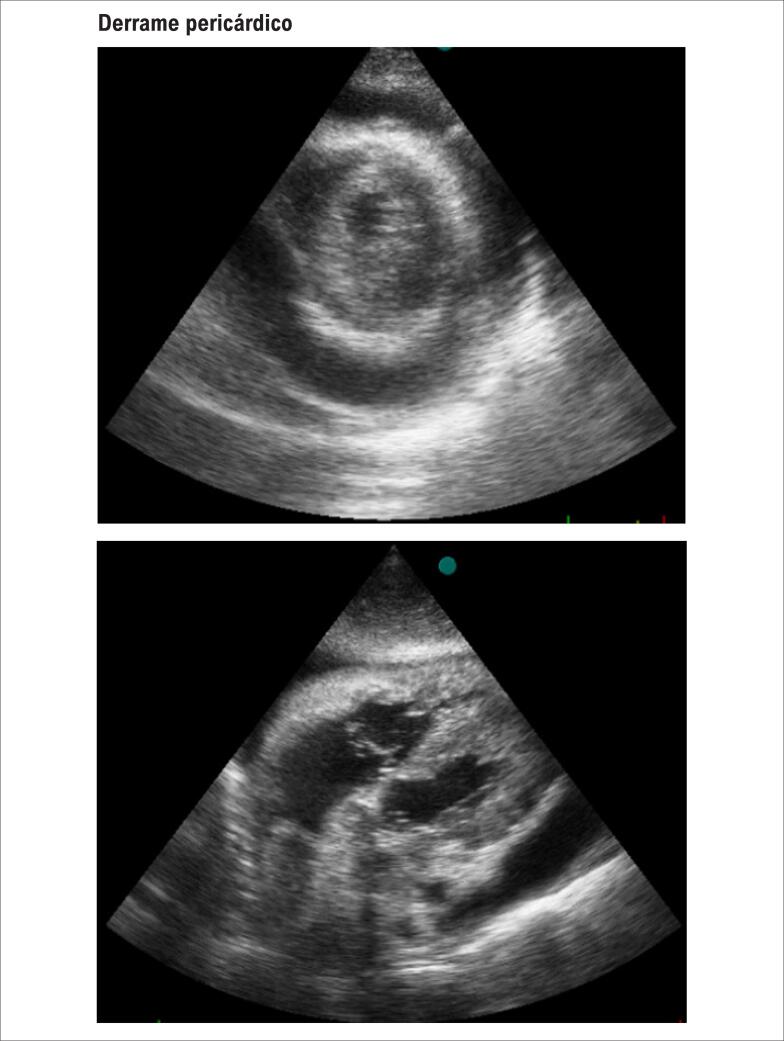
Derrame pericárdico volumoso. A: janela paraesternal transversal, onde se observa líquido ao redor de todo coração. B: janela subcostal, onde se observa o derrame ao redor de todo coração.

**Figura S7:**
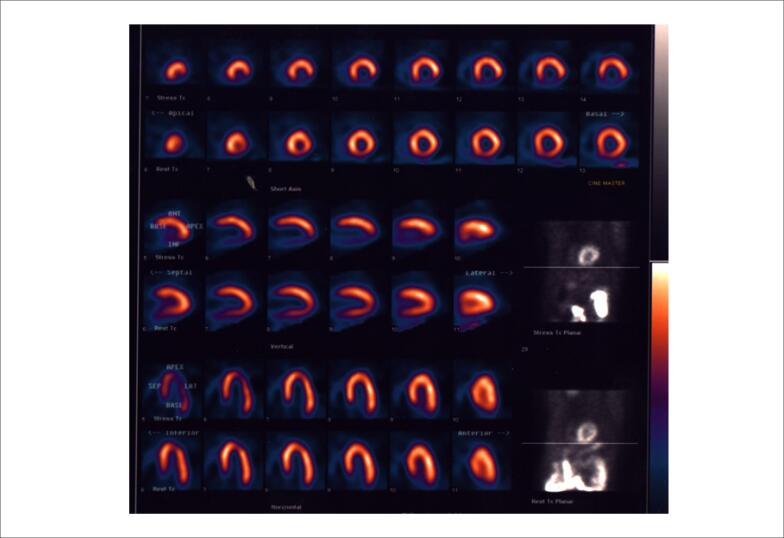
Fluxograma de atendimento ao paciente com dor torácica e instabilidade hemodinâmica.

**Figura S8:**
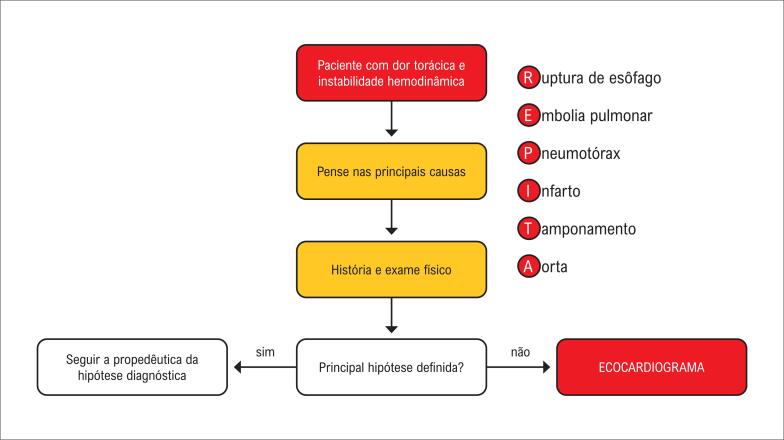
A cintilografia de perfusão miocárdica de estresse físico demonstrou defeitos de perfusão miocárdica reversíveis em território da artéria coronária direita correspondendo a 20% do miocárdio.

**1. Introdução** 10
**1.1. Relevância do Problema** 10
**1.2. Papel das Unidades de Dor Torácica** 11
**1.3. Objetivos da Diretriz** 11
**2. Métodos** 12
**2.1. Definições das Recomendações e Evidências** 12
**3. Atendimento Inicial** 12
**3.1. Critérios de Inclusão no Protocolo de Dor Torácica** 12
**3.1.1. Conceito de Primeiro Atendimento (First Medical Contact)** 12
**3.1.2. Avaliação Inicial de Possíveis Causas de Dor Torácica** 13
**3.1.3. Critérios Gerais de Elegibilidade para o Protocolo de Dor Torácica** 14
**3.1.4. Critérios Específicos para Abertura do Protocolo de Dor Torácica pela Enfermagem** 14
**3.1.5. Critérios Subjetivos por Decisão Médica** 14
**3.2. O Que Fazer Após a Abertura do Protocolo de Dor Torácica?** 15
**3.2.1. Quando e Como Realizar a Avaliação Detalhada da Dor Torácica (Diagnóstico Diferencial)?** 15
**3.3. Melhores Práticas para o Tempo Porta-ECG** 15
**3.3.1. Importância do ECG nos primeiros 10 minutos** 15
**3.3.2. Como Medir o Tempo Porta-ECG** 16
**3.3.3. Recomendações para Obter o Tempo Porta-ECG ideal** 16
**3.4. Interpretação do Eletrocardiograma** 17
**3.4.1. O Papel do Eletrocardiograma na Avaliação de Pacientes com Dor Torácica** 17
**3.4.2. Qual a Sensibilidade de um Eletrocardiograma Isolado na Avaliação de Pacientes com Dor Torácica? Qual o Ganho Adicional com Eletrocardiogramas Seriados?** 18
**3.4.3. Critérios Diagnósticos da Síndrome Coronariana Aguda ao Eletrocardiograma** 18
**3.4.4. Diagnósticos Diferenciais ao Eletrocardiograma** 19
**3.4.4.1. Alterações Primárias e Secundárias de Repolarização** 19
**3.4.4.2. Outros Diagnósticos ao Eletrocardiograma** 19
**3.4.4.3. Eletrocardiograma Não Diagnóstico** 19
**3.5. Como é Definido o Diagnóstico de Síndrome Coronariana Aguda?** 22
**3.5.1. Critérios Atuais para Diagnóstico de Angina Instável** 22
**3.5.2. Como Classificar a Síndrome Coronariana Aguda pelo Eletrocardiograma?** 22
**4. Rota de Investigação Diagnóstica após o ECG Inicial** 22
**4.1. Abordagem do Paciente com Critério de Instabilidade** 23
**4.1.1. Rotina Ecocardiográfica em Paciente com Dor Torácica e Instabilidade** 27
**4.2. Escores Clínicos de Risco e Probabilidade Diagnóstica** 27
**4.2.1. Escores Clínicos para Casos de Suspeita de Síndrome Coronariana Aguda** 29
**4.2.1.1. Em Qual Paciente Devo Aplicar o Escore HEART?** 30
**4.2.2. Escores Clínicos para Casos de Suspeita de Síndromes Aórticas Agudas** 30
**4.2.3. Escores Clínicos para Casos de Suspeita de Tromboembolismo Pulmonar** 30
**4.2.4. Outros Diagnósticos Diferenciais** 34
**4.3. Algoritmos de Troponina** 35
**4.3.1. Aplicação e Interpretação dos Marcadores de Injúria (Lesão) Miocárdica** 35
**4.3.1.1. Qual o Marcador de Escolha para o Diagnóstico de Infarto Agudo do Miocárdio?** 35
**4.3.1.2. Como Interpretar Exame de Troponina Alterado?** 35
**4.3.1.3. Como Interpretar Exames de Troponina Seriados?** 35
**4.3.1.4. Critérios Diagnósticos de Infarto Agudo do Miocárdio** 36
**4.3.1.5. Qual o Tempo Hábil para Descartar o Diagnóstico de Infarto Agudo de Miocárdio?** 36
**4.3.1.6. Qual o Algoritmo de Escolha?** 36
**4.3.2. Direcionamento Conforme Classificação no Algoritmo** 36
**4.3.2.1. SCA Descartada (Rule-Out)** 39
**4.3.2.2. Admissão Confirmada (Rule-In)** 40
**4.3.3. Direcionamento Inicial dos Casos em Zona Intermediária (Observação)** 41
**4.3.4. O que Fazer nos Casos em que há Injúria Miocárdica mas Coronária sem Obstruções?** 42
**4.3.4.1. Investigação de Casos de Troponina Elevada sem Obstrução Coronária (Troponin-Positive Nonobstructive Coronary Arteries - TINOCA ou TpNOCA)** 42
**4.4. Uso Racional de Testes Não Invasivos** 42
**4.4.1. O Papel do Teste de Esforço** 42
**4.4.2. Ecocardiografia na Avaliação da Dor Torácica** 46
**4.4.3. Cintilografia de Perfusão Miocárdica (SPECT) e Tomografia por Emissão de Pósitrons (PET-CT)** 47
**4.4.3.1. Indicações para o uso de Testes de Medicina Nuclear** 47
**4.4.3.2. Considerações Especiais em Mulheres com Dor Torácica Aguda** 49
**4.4.4. Tomografia Computadorizada Cardiovascular** 50
**4.4.4.1. Descarte Triplo** 52
**4.4.5. Ressonância Magnética Cardiovascular** 52
**4.4.5.1. Detecção de Isquemia Miocárdica** 52
**4.4.5.2. Diagnóstico Diferencial de Troponina Positiva com Coronárias Normais (TINOCA / MINOCA)** 53
**4.4.5.3. Miocardites** 54
**5. Modelo para Implantação das Unidades de Dor Torácica** 55
**5.1. Investigação de Síndrome Coronariana Aguda em Ambiente Pré-hospitalar** 55
**5.1.1. Percepção do Paciente e Rede Pré-hospitalar Organizada** 55
**5.1.2. Papel do Eletrocardiograma no Ambiente Pré-hospitalar** 55
**5.1.3. Papel da Troponina no Ambiente Pré-hospitalar** 55
**5.1.4. Papel dos Escores de Estratificação de Risco** 55
**5.1.5. Outras Ferramentas Diagnósticas** 55
**5.2. Modelo para Implantação das Unidades de Dor Torácica: Evidências para Melhores Práticas** 56
**5.2.1. Avaliação do Paciente** 57
**5.2.2. Estrutura Organizacional e Recursos Humanos** 58
**5.2.3. Necessidades Técnicas** 58
**5.2.4. Terapias Específicas** 59
**5.2.5. Indicadores de Gestão** 59
**5.2.6. Avanços Tecnológicos** 59
**Suplemento** 60
**Figura S1** 60
**Figura S2** 61
**Figura S3** 61
**Figura S4** 62
**Figura S5** 62
**Figura S6** 63
**Figura S7** 64
**Figura S8** 64
**Referências** 65

## 1. Introdução

### 1.1. Relevância do Problema

As doenças cardiovasculares (DCVs) representam a principal causa de morte no Brasil e no mundo, sendo a síndrome coronariana aguda (SCA) a principal causa específica de óbito cardiovascular.^1^ A SCA representa o espectro de manifestações clínicas e laboratoriais da isquemia miocárdica aguda e, de acordo com as definições atuais, é composta pela angina instável (AI) e pelo infarto agudo do miocárdio (IAM) sem (IAMSSST) ou com supradesnivelamento (elevação) do segmento ST (IAMCSST), com frequência de 30%, 34% e 36%, respectivamente, no registro ACCEPT (em português, Registro de Avaliação da Prática Assistencial em Síndrome Coronariana Aguda).^2^

Nos Estados Unidos da América (EUA), a prevalência de infarto agudo do miocárdio (IAM) é de aproximadamente 3% em adultos, sendo a doença coronariana responsável por cerca de 41% de todas as mortes cardiovasculares em 2020.^3 , 4^ No Brasil, a prevalência de IAM foi de cerca de 4,0% em pesquisa transversal envolvendo 7.260 indivíduos de diferentes etnias e regiões.^5^ Do ponto de vista econômico, o infarto do miocárdio representa o mais alto custo financeiro entre as doenças cardíacas para o Sistema Único de Saúde (SUS) (R$ 22,4 bilhões/6,9 bilhões de dólares em 2015).^6^ Tendo em vista que a SCA representa não só a principal causa de morte na população em geral,^7^ mas também a doença cardiológica de maior custo para o nosso sistema de saúde, a aplicação de métodos para diagnóstico precoce, preciso e eficiente desses pacientes representa um conjunto de ações prioritárias em termos de saúde pública.

O principal sintoma de um paciente com quadro de SCA é a dor torácica. Estima-se que sejam vistos anualmente nas salas de emergência dos EUA, cinco a oito milhões de indivíduos com dor torácica (ou sintomas equivalentes que geram suspeita de isquemia miocárdica aguda).^8^ Este número representa cerca de 5% de todos os atendimentos emergenciais naquele país, demonstrando a relevância do problema para a sociedade.^8 – 10^ Além de ser uma queixa frequente no pronto-socorro (PS), a dor torácica pode representar até 40% de admissões hospitalares pelo departamento de emergência, gerando alto custo para o sistema de saúde.^10 , 11^ Apesar de a dor torácica ser o sintoma mais comum no reconhecimento de um paciente com SCA, no ambiente do PS, apenas 5 a 20% dos pacientes com dor torácica têm SCA, e mais da metade dos casos de dor torácica não tem causa cardíaca.^12 – 14^ Uma atuação rápida e assertiva para diagnóstico e tratamento pode melhorar de forma significativa o prognóstico desses pacientes e reduzir os custos no sistema de saúde.

Em decorrência da elevada incidência dessa queixa em PS, o atendimento a pacientes com dor torácica é realizado por muitos médicos e/ou centros de saúde que não são especializados em DCVs. Dessa maneira, é fundamental a orientação adequada de todas as unidades e profissionais para o melhor manejo desses casos, no sentido de oferecer um cuidado seguro ao paciente e eficiente para o sistema de saúde. Essa avaliação visa não apenas o reconhecimento precoce dos casos de SCA, mas também de outros diagnósticos que apresentam risco de óbito na emergência, como dissecção de aorta e tromboembolismo pulmonar (TEP) – ou seja, embora a SCA seja a causa mais frequente de morte na população com dor torácica, há outros diagnósticos potencialmente fatais e tempo-sensíveis que também devem ser considerados na avaliação desses pacientes.

### 1.2. Papel das Unidades de Dor Torácica

Na década de 60, foram estabelecidas as unidades coronarianas (UCOs), cujo objetivo principal era oferecer um ambiente seguro para confirmação do diagnóstico e, principalmente, para o tratamento dos casos de IAM.^15^ Essas unidades trouxeram um impacto clínico relevante, com redução de cerca de 50% na mortalidade nos casos de IAM, especialmente pelo reconhecimento precoce e tratamento eficaz de arritmias e da parada cardíaca.^15 , 16^ Apesar do benefício clínico para os casos confirmados de IAM, a existência de um ambiente mais seguro levou a uma conduta mais liberal na prática clínica, em que os médicos também internavam em UCO muitos casos não confirmados, ou seja, pacientes apenas com a suspeita clínica de SCA.^15 , 16^ Como consequência dessa abordagem baseada em julgamento clínico subjetivo e heterogêneo (ao invés de protocolos objetivos), mais da metade dos pacientes internados nas UCOs não tinham, na verdade, SCA.^16^ Dessa forma, grande parte desses leitos de alta complexidade e alto custo passaram a ser ocupados por pacientes de baixa probabilidade diagnóstica e baixo risco de complicações, resultando não somente em saturação das UCOs, mas também em uma utilização inadequada de recursos.

A ausência de protocolos que possibilitassem maior assertividade na seleção de pacientes para internação gerou tanto a situação de superlotação das unidades coronárias, quanto um cenário de risco elevado para altas inadvertidas, ou seja, liberar do PS um paciente com IAM não reconhecido. Historicamente, nos EUA, de 1 a 10% dos pacientes que realmente estavam sofrendo um IAM acabavam sendo inapropriadamente liberados da sala de emergência por não terem a sua doença reconhecida, e esse grupo, liberado de maneira inadvertida, apresentava maior risco de morte.^17 – 20^ Em locais com menores recursos e/ou cujos médicos têm menor preparo para esse tipo de atendimento, a taxa de IAM não reconhecida é maior.^20^ Dessa forma, embora a SCA represente uma menor parte dos casos de dor torácica, na ausência de um atendimento sistematizado (protocolo de dor torácica), o paciente se torna mais vulnerável a falhas no diagnóstico (tanto subdiagnóstico como sobrediagnóstico), a despeito dos recursos disponíveis. Isso implica em maior risco de complicações fatais (riscos na liberação inadvertida de casos de IAM para casa) e maiores custos hospitalares (uso excessivo de uma unidade de alta complexidade, como a UCO, para pacientes de menor risco).

Tendo em vista esse cenário, as unidades de dor torácica (UDTs) foram criadas em 1982.^8 , 21 – 23^ Elas não representam necessariamente um espaço físico dedicado ao atendimento da dor torácica, mas são prioritariamente fluxos e processos que permitem uma avaliação sistematizada e baseada nas melhores práticas para os pacientes que apresentam suspeita diagnóstica de SCA.^24 , 25^ Para isso, é necessário que a equipe de médicos e enfermeiros esteja treinada e habituada ao manejo das urgências e emergências cardiovasculares.

Além da importância no melhor direcionamento diagnóstico, o protocolo de dor torácica possibilita a rápida aplicação de terapias baseadas em evidências. Embora os diagnósticos potencialmente fatais representem habitualmente < 10 a 20% dos casos de dor torácica, tais situações são tempo-sensíveis; portanto, quanto mais precoce o diagnóstico, mais rápida será a intervenção e, por consequência, mais vidas serão salvas, especialmente em situações como IAMCSST e dissecção aguda de aorta (DAA).

Em resumo, o protocolo para atendimento sistematizado do paciente com dor torácica ou sintoma suspeito de SCA visa:

1. Prover acesso fácil e prioritário ao paciente com dor torácica (ou sintoma equivalente) que procura a unidade de emergência (atendimento inicial);
2. Fornecer uma estratégia diagnóstica e terapêutica organizada na unidade de emergência, objetivando rapidez e alta qualidade de cuidados para atingir os melhores desfechos possíveis para os pacientes e, ao mesmo tempo, tendo foco na eficiência sobre o uso dos recursos disponíveis, ou seja, com entrega de valor em saúde (rota de investigação diagnóstica e rota terapêutica).^25^

### 1.3. Objetivos da Diretriz

- Fornecer orientação prática para médicos (cardiologistas ou não) e equipe multiprofissional sobre a abordagem inicial dos pacientes com início agudo de dor torácica (ou sintomas equivalentes), de acordo com as melhores práticas baseadas em evidências;
- Estabelecer a rotina de atendimento de pacientes com suspeita de SCA (e seus diagnósticos diferenciais), minimizando os tempos para a realização de procedimentos diagnósticos e terapêuticos críticos para o mais rápido restabelecimento do paciente;
- Orientar a utilização de recursos, tendo como prisma a entrega de valor em saúde e que seja adaptável ao cenário de atendimento;
- Orientar a coleta de informações sobre a prática assistencial para que seja possível avaliar a qualidade do atendimento ao estabelecer metas mensuráveis dos tempos envolvidos e das melhores práticas no protocolo de dor torácica. Essa informação é fundamental para identificar lacunas no atendimento e avaliar o resultado de ações contínuas da melhoria de qualidade.

De uma forma resumida, há três grandes alvos na implementação de um protocolo de dor torácica ( Tabela 1 ) que visam:

**Tabela 1:** Objetivos do protocolo de dor torácica e a ação correspondente que é necessária para atingir cada objetivo

Objetivos	Ação
Identificar, de forma precoce, pacientes com SCA (e outras doenças tempo-sensíveis)	ECG em até 10 minutos (repetir ECG, seriar marcadores e solicitar exames para diagnósticos diferenciais conforme indicado)
Evitar altas inadvertidas e internações desnecessárias	Algoritmos de decisão (pathways) baseados nas melhores práticas
Salvar vidas e reduzir sequelas (por exemplo, insuficiência cardíaca pós-infarto)	Tratamento precoce baseado em evidências (seguir diretrizes de tratamento específicas, como as diretrizes de SCA)

SCA: síndrome coronariana aguda; ECG: eletrocardiograma.

- A excelência em indicadores sobre o processo de atendimento (por exemplo, métricas de tempo para o diagnóstico);
- Melhor eficiência para o sistema de saúde (métricas que avaliem a alocação apropriada de recursos);
- Redução nos percentuais de desfechos negativos para os casos confirmados de doenças potencialmente fatais (ex: reduzir desfechos como morte e disfunção ventricular na SCA).

## 2. Métodos

### 2.1. Definições das Recomendações e Evidências

#### Recomendações

**Classe I:** condições para as quais há evidências conclusivas, ou, na sua falta, consenso geral de que o procedimento é seguro e útil/eficaz.

**Classe II:** condições para as quais há evidências conflitantes e/ou divergência de opinião sobre segurança e utilidade/eficácia do procedimento.

**Classe IIa:** peso ou evidência/opinião a favor do procedimento. A maioria aprova.

**Classe IIb:** segurança e utilidade/eficácia menos bem estabelecida, não havendo predomínio de opiniões a favor.

**Classe III:** condições para as quais há evidências e/ou consenso de que o procedimento não é útil/eficaz e, em alguns casos, pode ser prejudicial.

#### Evidências

**Nível A:** dados obtidos a partir de mais de um estudo randomizado, concordantes e/ou de metanálise robusta de estudos clínicos randomizados.

**Nível B:** dados obtidos a partir de metanálise menos robusta, a partir de um único estudo randomizado e/ou de estudos não randomizados (observacionais).

**Nível C:** dados obtidos de opiniões consensuais de especialistas e/ou pequenos estudos não randomizados (observacionais).

## 3. Atendimento Inicial

### 3.1. Critérios de Inclusão no Protocolo de Dor Torácica

#### 3.1.1. Conceito de Primeiro Atendimento (First Medical Contact)

O termo "first medical contact" (FMC) se refere ao primeiro contato com profissional/serviço de saúde, idealmente realizado no pré-hospitalar. A abordagem inicial dos pacientes com dor torácica é sempre feita no sentido de confirmar ou afastar o diagnóstico de SCA ou de outra condição potencialmente fatal (por exemplo, DAA). Em virtude dos inúmeros diagnósticos diferenciais, realizar o diagnóstico adequado da dor torácica tem sido um desafio para os médicos que trabalham na linha de frente das emergências, unidades de pronto atendimento e sistemas de ambulância pré-hospitalar.

O profissional da emergência, particularmente quem faz o atendimento inicial, diante de um paciente com queixa de dor torácica, deve definir sobre o início urgente ou não de terapias específicas, considerando que algumas patologias agudas têm risco imediato de óbito, como síndromes aórticas agudas (dissecção, aneurisma e hematoma), SCA (AI e IAM), TEP e pneumotórax hipertensivo. Na unidade de emergência, esse primeiro atendimento (do inglês, *first medical contact [FMC]* ) pode ser realizado por um profissional médico ou não (por exemplo, enfermeiro da triagem).

**Figura 1:**
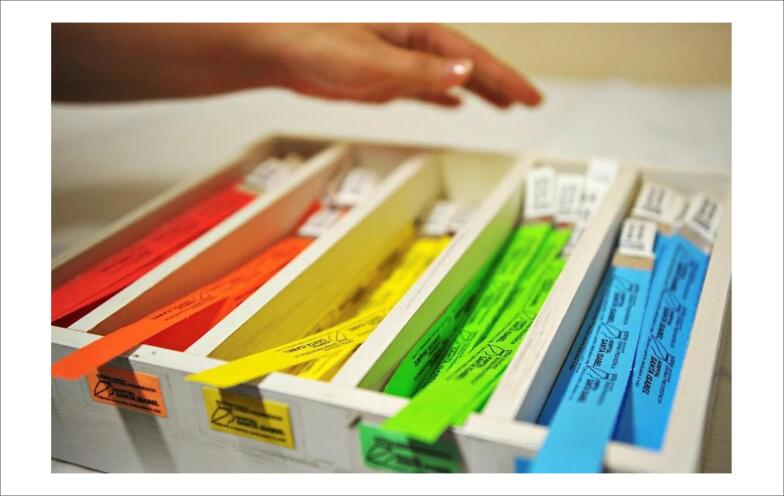
Triagem de Manchester – pulseiras de identificação.

A rapidez do atendimento adequado é fundamental e os sistemas de triagem nas unidades de emergência baseados em algoritmos têm relevância neste processo. Um destes exemplos é a triagem de Manchester que é um protocolo de seleção de pacientes que classifica o nível de urgência/emergência por um código de cores. Essa metodologia surgiu em 1997, na cidade de Manchester, na Inglaterra, e, por sua eficiência, rapidamente se tornou um sistema global. No entanto, esse modelo de triagem chegou de forma efetiva ao Brasil cerca de 10 anos depois, na tentativa de reduzir as filas em hospitais e possíveis perdas de pacientes que se encontravam em estados mais graves. O protocolo de Manchester funciona a partir de um código de cinco cores, atribuídas às pulseiras de identificação dos pacientes, conforme visto na Figura 1 .^26^ Tendo em vista que todo paciente que se apresenta na unidade de emergência com dor ou sintoma equivalente sugestivo de isquemia deve receber atendimento médico dentro de 10 minutos,^27^ portanto, ao aplicar a triagem de Manchester nos pacientes contemplados nesta diretriz, os profissionais da área da saúde deverão estabelecer níveis de prioridade "vermelho" ou "laranja", garantindo agilidade e segurança para esses pacientes (em ambos os casos, o ECG deve ser realizado nos primeiros 10 minutos). Estudos demonstraram a capacidade da enfermagem usando a triagem Manchester (TM) em detectar pacientes de alto risco com dor torácica,^28^ o impacto da utilização da TM na mortalidade em curto prazo de pacientes com SCA,^29^ a sensibilidade e especificidade da TM para pacientes com SCA^30^ e se esse sistema de triagem tem sido usado efetivamente em pacientes admitidos com diagnóstico de SCA.^31^ Adaptações do protocolo de Manchester (ou equivalente) podem ser feitas de acordo com avaliações contínuas da qualidade assistencial local e de acordo com as especificidades de cada serviço, tendo como objetivo a melhor *performance* na triagem desses pacientes.

Finalmente, a inclusão adequada no protocolo de dor torácica pode evitar a liberação inapropriada de pacientes com SCA e reduzir internações e exames diagnósticos desnecessários. O protocolo é um guia que traz segurança e evita variabilidade desnecessária; entretanto, a avaliação clínica é um elemento fundamental para a melhor aplicação do protocolo de dor torácica.^32 , 33^

#### 3.1.2. Avaliação Inicial de Possíveis Causas de Dor Torácica

Ao avaliar um paciente com dor torácica, deve-se considerar as causas que necessitam de conduta mais rápida, não apenas por seu alto potencial de letalidade, mas também pelo curto horizonte de tempo para intervenção. Não existem sinais patognômicos de uma ou outra patologia e o ancoramento a uma única doença, mesmo que seja a doença mais prevalente, levará a erros de conduta. Essa seção sugere uma lógica inicial de avaliação focada nas condições que precisam ser manejadas de forma mais precoce.

Dessa forma, a primeira causa em termos de gravidade é a dissecção de aorta. Apesar da prevalência de dissecção de aorta ser menor (um caso para cada 20 a 90 casos de IAM),^34 , 35^ contudo, se o diagnóstico não for considerado, nunca será feito e a letalidade estimada é de cerca de 50% em 48 horas. Além disso, caso seja confundido com uma SCA, o uso inicial de antiagregação ou anticoagulação (contraindicado nos casos de dissecção) será deletério para os pacientes.^36 , 37^

- Dissecção de aorta: deve-se avaliar a presença de fatores de risco para dissecção de aorta, déficits de perfusão (diferença de pulso ou pressão arterial) e alargamento de mediastino. Existem fluxogramas descritos abaixo, como o como o *aortic dissection detection risk score* (ADD-RS) e o *aorta simplified score* (AORTA), para auxiliar na tomada de decisão. O ADD-RS contou com a inclusão de pacientes brasileiros no Instituto do Coração, melhorando sua aplicabilidade em nossa população. No caso de suspeita de dissecção de aorta, qualquer antiagregante ou anticoagulante deve ser adiado até a confirmação ou descarte do diagnóstico pelo fluxograma escolhido (vide mais detalhes na seção 4.2.2);
- SCA: a SCA é a causa mais comum de dor torácica potencialmente fatal e será discutida em mais detalhes a partir da próxima seção;^38^
- TEP: esta é outra causa de dor torácica com potencial de letalidade alto. A dor torácica do TEP é descrita como pleurítica (aumenta com inspiração) e de início súbito. Situações que promovem imobilidade, como lesões/tratamentos ortopédicos ou outros pós-operatórios, aumentam a probabilidade de formação de trombose venosa profunda com posterior embolização pulmonar. Escores como o Wells e Genebra (do inglês, Geneva) podem ser usados para estratificar o risco de TEP e trilhar o fluxograma para o seu diagnóstico. Caso seja indicada a dosagem de D-dímero, usar como ponto de corte para nível "positivo" o valor ≥500 ng/mL (μg/L) ou usar valor ajustado por idade (idade x 10 para pacientes a partir dos 50 anos), o qual ajuda na especificidade do ponto de corte do exame (se utilizar os critérios YEARS, ponto de corte do D-dímero por ser 500 ou 1000 ng/mL de acordo, respectivamente, com a presença ou a ausência de critérios clínicos do YEARS).^39 , 40^ Além disso, em pacientes com média ou alta probabilidade de TEP e sem sangramento, poderá estar indicada indicada a primeira dose da anticoagulação plena (vide mais detalhes na seção 4.2.3);
- Pneumotórax: o pneumotórax simples pode se apresentar como dor torácica, que pode até ocorrer de forma espontânea em pacientes longilíneos, mas geralmente é um quadro benigno. Já o pneumotórax hipertensivo é grave e deve ser tratado imediatamente. No entanto, comumente acontece no contexto do trauma, e sua identificação e tratamento fazem parte da avaliação primária de casos de trauma torácico ou politraumatismo. No ambiente intra-hospitalar, o pneumotórax hipertensivo pode ocorrer como complicação de procedimentos como intubação orotraqueal (perfuração traqueal) ou acesso venoso central;^41^
- Pericardiopatias: o tamponamento cardíaco pode ser referido como um desconforto torácico. Pacientes com esse quadro se apresentam hipotensos ou em franco choque. A tríade de Beck apresenta utilidade limitada. As sensibilidades dos componentes individuais da tríade de Beck para tamponamento cardíaco são baixas: hipotensão (26 ± 10%), sons cardíacos diminuídos (28 ± 7%) e pressão venosa jugular elevada (76 ± 14%).^42^ Outro estudo encontrou sensibilidade ainda mais baixa para a tríade de Beck.^43^ A diferenciação desse quadro, em relação a outros choques circulatórios, tornou-se muito mais fácil com a ultrassonografia à beira-leito e permite o manejo guiado por ultrassom;
- Condições esofágicas graves: estas também podem se manifestar como dor torácica. A rotura esofágica com mediastinite é um quadro bastante raro, com incidência relatada em 3 a cada 1.000.000,^44^ geralmente associada a vômitos forçados em pacientes etilistas (síndrome de Boerhaave). A avaliação e manejo precoce impactam o prognóstico;^45^
- Outros diagnósticos: após exclusão dos diagnósticos iniciais, outros diagnósticos com potenciais riscos de complicações também poderão ser estabelecidos, especialmente em cenários de injúria miocádica (elevação de troponina). Um exemplo seria a síndrome do coração partido, ou Takotsubo, que é uma cardiomiopatia a qual, em 2/3 dos casos, pode estar associada a fatores emocionais ou orgânicos fortes, como sepse ou hemorragia cerebral. Provoca uma disfunção miocárdica intensa, habitualmente com preservação dos segmentos basais do ventrículo esquerdo (VE) (existem variantes também). Muitas vezes, o quadro será indistinguível de uma síndrome coronariana aguda e o diagnóstico será retrospectivo. Em casos de choque cardiogênico, é importante conhecer a associação de até 1/5 dos casos com obstrução de via de saída de ventrículo esquerdo, que pode se agravar com administração de inotrópicos.^46^

#### 3.1.3. Critérios Gerais de Elegibilidade para o Protocolo de Dor Torácica

Todos os pacientes atendidos com origem domiciliar ou transferidos de outros serviços de saúde, que tenham história de dor torácica, ou sintoma equivalente, de início agudo, são elegíveis para o protocolo de dor torácica.

#### 3.1.4. Critérios Específicos para Abertura do Protocolo de Dor Torácica pela Enfermagem

Os critérios gerais, citados acima, delimitam a população contemplada nesta diretriz. Entretanto, para que seja possível a abertura de um protocolo por diversos profissionais, como médicos e enfermeiros, com níveis de experiência diferentes, devemos ter critérios objetivos e de alta sensibilidade. Embora o objetivo geral deste tipo de critério seja oferecer segurança, pela sua alta sensibilidade (para não deixar que quantidade significativa de casos graves tenham atraso no reconhecimento), por outro lado, critérios muito amplos podem gerar sobrecarga nas unidades de emergência, por incluírem pacientes com probabilidade muito baixa de serem beneficiados. Desse modo, os critérios para abertura do protocolo de dor torácica devem ser definidos de acordo com o objetivo específico do diagnóstico precoce desejado, tendo um padrão mínimo a ser seguido ( Tabelas 2 e 3 ).

**Tabela 2:** Critérios rotineiros para abertura do protocolo de dor torácica

	Classe de recomendação	Nível de evidência
Qualquer dor ATUAL (vigente na admissão) entre a cicatriz umbilical e a mandíbula.	I	C
Qualquer dor torácica que tenha durado mais de 10 minutos (mesmo que ausente na admissão).	I	C

* Esses critérios rotineiros apresentam foco no reconhecimento de síndrome coronariana aguda com oclusão coronária e, portanto, com potencial benefício para terapia de reperfusão, além de diagnósticos de risco de letalidade (por exemplo, dissecção aguda de aorta).

**Tabela 3:** Critérios adicionais para abertura do protocolo de dor torácica

	Classe de recomendação	Nível de evidência
Paciente assintomático na admissão, mas que apresenta relato de queixa de desconforto epigástrico ou dor em braços ou mandíbula antes da entrada no pronto-socorro.	IIa	C
Equivalentes isquêmicos (por exemplo: dispneia, diaforese e/ou PAS < 90 mmHg) em pacientes acima dos 50 anos de idade e/ou com histórico de diabetes ou doença cardiovascular conhecida (por exemplo, história de infarto, angioplastia e acidente vascular cerebral).	IIa	C
Solicitação após avaliação médica, na ausência dos critérios acima (suspeita médica de síndrome coronariana aguda ou de outras condições potencialmente fatais, como dissecção de aorta).	IIa	C

PAS: pressão arterial sistólica.

* Tendo em vista que 10 a 30% dos pacientes com síndrome coronariana aguda não apresentam dor torácica (especialmente idosos ou com comorbidades como diabetes),^47 , 48^ a utilização de critérios mais sensíveis que incluam equivalentes possibilita uma detecção mais ampla de casos. Essa ampliação deve ser acompanhada de uma avaliação para uso adequado dos recursos (ou seja, manter vigilância e ações contínuas para evitar desperdícios e situações de superlotação de unidades de dor torácica).

#### 3.1.5. Critérios Subjetivos por Decisão Médica

Conforme a última linha de recomendação descrita na Tabela 3 , além dos casos que apresentam critérios objetivos que permitem a rápida identificação no atendimento de enfermagem, também podem ser incluídos no protocolo de dor torácica os pacientes que, após avaliação médica, apresentem suspeita diagnóstica de condições que possam ser confundidas com SCA ou outros diagnósticos potencialmente fatais (como embolia pulmonar, dissecção de aorta etc.).

Manifestações menos específicas como síncope, fraqueza, confusão mental ( *Delirium* ), "indigestão", náuseas e vômitos inexplicados também podem ser considerados equivalentes, especialmente em pacientes de alto risco (idosos, diabéticos, antecedente de doença cardiovascular). Nesses casos, pode ser solicitada abertura de protocolo de dor torácica pelo médico mesmo na ausência dos critérios objetivos. Como há recomendação de avaliação de ECG de rotina nos casos de síncope, essa manifestação pode ser incluída como um critério objetivo padrão para realizar ECG nos primeiros 10 minutos nas rotinas de pronto-atendimento (embora, não necessariamente, deva-se prosseguir a investigação de SCA, além do ECG).

### 3.2. O Que Fazer Após a Abertura do Protocolo de Dor Torácica?

Todo paciente atendido com quadro suspeito de SCA, ou seja, que preenche os critérios de abertura do protocolo de dor torácica, deve ser abordado como urgência/emergência médica, sendo encaminhado imediatamente para realização de eletrocardiograma (ECG), e a equipe médica deve ser avisada para avaliar prontamente o caso e o ECG ( Tabela 4 ). O paciente deve seguir, idealmente, o acrônimo MOVE:

M: M onitorização com desfibrilador prontamente disponível.
O2: Checar saturação de O 2 (oxigenioterapia se saturação < 90%).
V: Acesso v enoso (coletar sangue para exames laboratoriais durante a obtenção do acesso).
E: E letrocardiograma em até 10 minutos, com avaliação médica imediata.

**Tabela 4:** Conduta diagnóstica inicial após abertura do protocolo de dor torácica

	Classe de recomendação	Nível de evidência
Em pacientes com critérios para abertura do protocolo de dor torácica, deve-se realizar um eletrocardiograma de 12 derivações (idealmente, adicionar derivações complementares), que deve ser avaliado por um médico em até 10 minutos (tempo total desde o primeiro contato com profissional/serviço de saúde).	I	B

* No protocolo de dor torácica, recomenda-se realizar o eletrocardiograma com paciente monitorizado (especialmente se sintoma persistente) ou, pelo menos, em ambiente que possua desfibrilador prontamente disponível.

#### 3.2.1. Quando e Como Realizar a Avaliação Detalhada da Dor Torácica (Diagnóstico Diferencial)?

Tendo em vista que a avaliação inicial dos pacientes costuma ser na triagem de enfermagem da unidade de emergência e que também há uma recomendação classe I para realizar ECG em até 10 minutos de rotina em pacientes com dor torácica (ou sintomas equivalentes), todo paciente com critério de abertura do protocolo de dor torácica deve seguir para imediata realização do ECG e acionamento médico (em paralelo). Como essa regra vale tanto para quadros de dor "típica" como "atípica" (nomes em desuso), a prioridade é realizar o ECG rapidamente. A avaliação detalhada das características da dor torácica (e/ou sintomas equivalentes) deve ser feita em paralelo (não deve atrasar o ECG).

### 3.3. Melhores Práticas para o Tempo Porta-ECG

#### 3.3.1. Importância do ECG nos primeiros 10 Minutos

O ECG de 12 derivações em repouso é a ferramenta diagnóstica de primeira linha na avaliação de pacientes com suspeita de SCA. Deve ser realizado em até 10 minutos após a chegada do paciente ao PS ou, idealmente, no primeiro contato com os serviços médicos de emergência, em ambiente pré-hospitalar, e ser imediatamente interpretado por um médico qualificado.^49 , 50^ Isso implica que a realização do ECG pode preceder até mesmo a finalização de etapas fundamentais da avaliação clínica, como uma anamnese detalhada e o exame físico completo.^51^

Uma análise de mais de 7.500 pacientes de um registro nos EUA e no Canadá demonstrou que apenas 40% dos pacientes com IAMCSST receberam um ECG dentro de 10 minutos (tempo médio de 14 minutos), e que um tempo porta-ECG (TPE) superior a 10 minutos foi associado a risco aumentado de IAM recorrente ou morte ( *odds ratio* 3,95, IC 95% 1,06-14,72, p = 0,04).^52^

O fundamento para essa abordagem consiste na evidência de que, na presença de supradesnível do segmento ST de origem isquêmica (ou alteração equivalente), a obtenção precoce do ECG resulta em rapidez na terapia de reperfusão miocárdica e, consequentemente, em impacto positivo na morbidade e mortalidade. De fato, o aumento do TPE é associado a um aumento do tempo porta-balão para intervenção coronária percutânea^53^ e do tempo porta-agulha para fibrinólise.^54^ Além disso, o ECG realizado no atendimento pré-hospitalar, como em ambulâncias do Serviço de Atendimento Móvel de Urgência (SAMU), pode auxiliar na redução do tempo de diagnóstico e no adequado encaminhamento de pacientes com IAMCSST para centros capazes de realizar angioplastia primária de forma precoce, os quais podem receber o ECG antes da chegada da ambulância e, dessa forma, preparar-se para receber os pacientes diretamente na unidade de hemodinâmica, sem a necessidade de uma "parada" no departamento de emergência.^55^

#### 3.3.2. Como Medir o Tempo Porta-ECG

O aspecto mais importante na decisão sobre quais parâmetros usar para o TPE é escolher parâmetros objetivos, acessíveis e confiáveis nos respectivos serviços. Apesar do tempo "porta" remeter à entrada do paciente na unidade de emergência, nem sempre esse parâmetro é facilmente acessível para fins de controle de indicadores de qualidade. Recomenda-se usar os padrões descritos a seguir para comparação de indicadores.

Tempo porta: um método muito utilizado é o do FMC como tempo "porta", ou seja, o primeiro contato com um profissional da área da saúde (habitualmente este atendimento inicial ocorre na triagem do PS). A utilização desse parâmetro permite também comparações com serviços que seguem essa padronização, adotada internacionalmente.^56^ Um limitante seria o de eventuais atrasos entre o momento da entrada do paciente na unidade até a realização da triagem; portanto, mesmo que se adote o FMC como tempo "porta" (para fins de comparação com *benchmarkings* externos), é muito importante monitorar também o tempo pré-triagem (mesmo que seja para ações de melhoria interna de cada serviço).

Tempo ECG: como dito, embora teoricamente o ideal fosse o momento da análise do ECG (laudo*), nem sempre esses parâmetros estão disponíveis. Dessa forma, o horário registrado no ECG pode ser considerado, desde que esse dado esteja ajustado (monitoramento periódico da enfermagem e engenharia clínica do horário no aparelho) e que, na rotina do serviço, o ECG seja imediatamente entregue para o médico – não há benefício algum para o paciente quando um ECG é realizado nos primeiros 10 minutos, mas não é interpretado imediatamente.

#### 3.3.3. Recomendações para Obter o Tempo Porta-ECG Ideal

Na avaliação do TPE, inicialmente é recomendado que seja avaliado qual é o tempo atual em seu serviço, de cada etapa – desde a entrada até a realização do ECG –, para que haja um mapeamento de processos da prática local da instituição. Isso permitirá um maior entendimento sobre quais intervenções podem ter maior impacto nos seus processos mapeados. Deve-se também realizar *feedback* contínuo com toda a equipe envolvida, que inclui o médico e equipe de enfermagem do departamento de emergência.^54 – 58^

Uma estratégia comumente relatada em implementações bem-sucedidas envolve a adequação da triagem para permitir um ECG dedicado na unidade com técnico habilitado.^57 – 65^ Sprockel et al. avaliaram 373 pacientes, 204 na fase pré e 169 na fase pós-intervenção. A mediana do tempo foi de 16 minutos na fase anterior, e 41% dos casos foram inferiores a 10 minutos; após a implementação da mudança no processo de atenção à dor torácica, com disponibilidade de aparelho de ECG na triagem e técnico exclusivo, a mediana foi de 5 minutos, com 63% dos casos abaixo de 10 minutos, o que apresentou diferença estatisticamente significativa.^61^

Outra estratégia comumente recomendada é o treinamento da equipe de triagem tanto para priorizar a realização do ECG nos casos com critérios de abertura do protocolo como também para melhorar o reconhecimento de sinais e sintomas da SCA que vão além da dor torácica típica e incluem outros sintomas equivalentes (por exemplo, dor epigástrica).^60 , 62 , 63^

Phelan et al. identificaram inicialmente em seu serviço duas causas principais de TPE > 10 minutos: (1) atraso prioritário (por exemplo, conclusão de triagem e tarefas de entrada de dados de registro antes do ECG) e (2) falha em reconhecer pacientes com sintomas de SCA. Após as intervenções, que incluíram processo de priorização de pacientes para triagem, designação de equipe para relizar ECG de forma imediata, *feedback* contínuo e uma iniciativa educacional da equipe de triagem para identificar pacientes de alto risco, o tempo médio para ECG reduziu significativamente, de 21,28 ± 5,49 minutos para 9,47 ± 2,48 minutos (p < 0,033), representando uma melhora de 55%.^64^

Um estudo brasileiro realizado com o intuito de identificar os problemas na logística do TPE identificou três problemas principais: 1) a demora entre a chegada ao hospital e o primeiro atendimento médico (resolvida com o protocolo de triagem em horário integral); 2) a comunicação intra-hospitalar e falta de prioridade (melhoradas com o código padronizado); 3) o retardo diagnóstico, agilizado com a presença do médico emergencista cardiologista.^65^

Também existem dados que sugerem que sexo, raça, fluência, diabetes e tipo de sintomas estão associados a atrasos no diagnóstico do IAMCSST. Uma coorte retrospectiva de 3 anos, com 676 pacientes em 10 hospitais dos EUA, identificou que o TPE > 10 minutos, quando comparado com TPE ≤ 10 minutos, era mais provável de ocorrer em mulheres (32,8% *versus* 22,6%, p = 0,005), negros (23,4% *versus* 12,4%, p = 0,005), pessoas sem inglês fluente (24,6% *versus* 19,5%, p = 0,032), diabéticos (40,2% *versus* 30,2%, p = 0,010) e naqueles que com menos frequência relataram dor no peito (63,3% *versus* 87,4%, p < 0,001).^66^

Em resumo, não podemos afirmar que há uma única estratégia específica para redução do TPE, mas sim uma combinação de ações que podem se adequar ao seu serviço ( Tabela 5 ). A maioria dos estudos lançou mão de uma multiplicidade de intervenções simultâneas, o que muitas vezes dificulta a identificação específica de uma melhor estratégia isoladamente. Portanto, devemos mapear e entender o contexto local, identificar as dificuldades para, então, podermos implementar uma abordagem agrupada de forma eficiente, com o intuito principal de reduzir o TPE ( Tabela 6 ).

**Tabela 5:** Principais ações para redução do tempo porta-ECG

1. Mapeamento de processos para reconhecimento de falhas
2. ECG dedicado com técnico treinado na triagem
3. Treinamento da equipe da triagem para reconhecimento de sintomas suspeitos
4. Organização de fluxo da triagem (diminuir burocracias e priorizar ECG nos casos com indicação)
5. *Feedback* constante à equipe do TPE

ECG: eletrocardiograma; TPE: tempo porta–ECG.

**Tabela 6:** Tempo porta-eletrocardiograma

	Classe de recomendação	Nível de evidência
Serviços de emergência devem monitorar o tempo porta-eletrocardiograma, realizar *feedback* contínuo para a equipe responsável por cada etapa do atendimento inicial e implementar ações de melhoria de acordo com as oportunidades identificadas.	I	B

### 3.4. Interpretação do Eletrocardiograma

A avaliação médica (anamnese e exame físico) pode ocorrer de forma concomitante à realização e interpretação do ECG. Vale lembrar que casos de IAMCSST podem ser ocasionados por dissecção de aorta tipo A com comprometimento coronário. Dessa forma, embora o ECG seja realizado de forma imediata (primeiros 10 minutos), a avaliação clínica deve ocorrer de forma paralela e, mesmo nos casos com critérios inequívocos no ECG, devemos ter atenção também a outros diagnósticos possivelmente associados.^67^

#### 3.4.1. O Papel do Eletrocardiograma na Avaliação de Pacientes com Dor Torácica

O ECG é uma ferramenta diagnóstica amplamente disponível, de baixo custo e de fácil interpretação local (e até mesmo à distância), possibilitando seu uso em diversos sistemas de atendimento em saúde.^68^ A realização do ECG constitui uma etapa fundamental na avaliação diagnóstica de pacientes com dor torácica (e sintomas equivalentes) em diferentes ambientes (sala de emergência, pré-hospitalar). Baseado nos seus achados, podemos identificar pacientes que se beneficiam imediatamente da terapia de reperfusão, identificar a artéria coronária envolvida e o local da obstrução, estratificar o risco e decidir a terapia inicial mais apropriada para cada caso.^69^

#### 3.4.2. Qual a Sensibilidade de um Eletrocardiograma Isolado na Avaliação de Pacientes com Dor Torácica? Qual o Ganho Adicional com Eletrocardiogramas Seriados?

Embora o ECG seja um exame de grande importância no diagnóstico e tratamento da SCA, um único ECG de 12 derivações realizado no atendimento inicial do paciente com dor torácica, apresenta baixa sensibilidade, podendo detectar menos de 50% dos IAMs (incluindo IAM com e sem supradesnível do segmento ST).^70 , 71^

A isquemia miocárdica aguda é frequentemente associada a alterações dinâmicas no ECG. Eletrocardiogramas seriados podem trazer informações relevantes e aumentar de maneira significativa a sensibilidade para até 70 a 90%.^72^ Algumas condições, como oclusão da artéria circunflexa, podem cursar sem alterações significativas ao ECG de 12 derivações, sendo indicada a realização das derivações V7, V8 e V9 para pesquisa de alterações como as relacionadas ao infarto da parede "posterior"^73^ (termo em desuso, pois após estudos de ressonância cardíaca mostrou-se tratar de porção da parede lateral).^74^ Essa situação sempre deve ser suspeitada, principalmente em pacientes que persistem com dor torácica sugestiva de isquemia miocárdica. Esses pacientes, com sintoma persistente e ECG inicial não diagnóstico, são também os que se recomenda repetir ECG com maior frequência (a cada 10 a 20 minutos), com os eletrodos em posição fixa, enquanto durar o sintoma ou esclarecer o diagnóstico – com pelo menos 2 a 4 repetições habitualmente – ou com o uso de registro contínuo de ECG de 12 derivações assistido por computador (se disponível) para detectar alterações dinâmicas no ECG.^75^

#### 3.4.3. Critérios Diagnósticos da Síndrome Coronariana Aguda ao Eletrocardiograma

Do ponto de vista prático, três mensagens são fundamentais.

**Figura 2:**
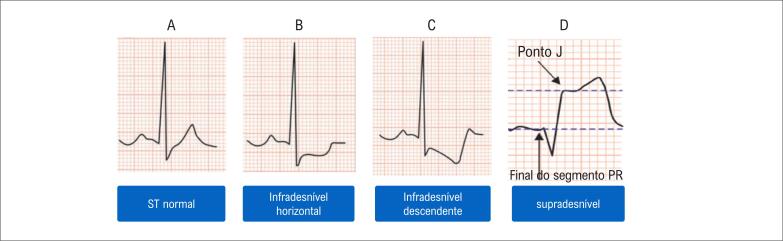
Representação das alterações no segmento ST. A) segmento ST normal, B) infradesnível horizontal e C) infradesnível descendente do ST, descritos nos laudos eletrocardiográficos como corrente de lesão subendocárdica; D) supradesnível do segmento ST, mostrando a maneira correta de medi-lo.

**Figura 3:**
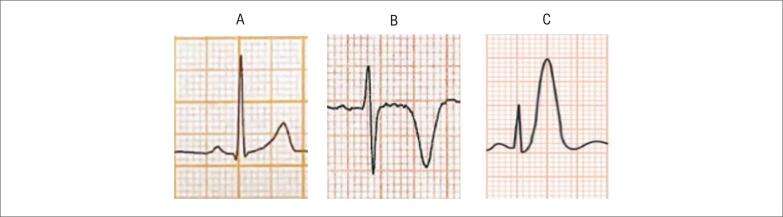
Representação das alterações na onda T. A) onda T normal, observar a assimetria com ascensão lenta e descenso mais rápido; B) a onda T isquêmica torna-se simétrica, invertida e de maior amplitude (isquemia subepicárdica ou pós-isquemia transmural); C) onda T de grande magnitude e simétrica, chamada de T hiperaguda (isquemia subendocárdica), costuma preceder o aparecimento do supradesnível do segmento ST. A magnitude representa a área (ou seja, onda T de base larga e que habitualmente apresenta também maior amplitude).

1. ECG normal ou com alterações inespecíficas não permite excluir SCA;
2. Eletrocardiogramas seriados aumentam a acurácia para o diagnóstico de SCA;
3. Alterações sugestivas de isquemia aguda se baseiam, principalmente, em modificações no segmento ST ( Figura 2 ) e/ou nas ondas T ( Figura 3 ); as alterações no segmento ST devem ser aferidas no ponto J, tendo como referência o segmento PR como linha de base.^19 , 21^

Os critérios eletrocardiográficos recomendados por esta diretriz para o diagnóstico de SCA permitem não apenas o diagnóstico de SCA com e sem elevação de ST, mas também a definição de oclusão coronária e o risco do paciente ( Tabela 7 ). Dessa forma, além dos critérios objetivos clássicos de elevação de ST, algumas alterações específicas têm sido identificadas como "equivalentes" à SCA com elevação de ST. Essas alterações podem indicar alta probabilidade de artéria coronária ocluída, mesmo sem critérios clássicos de SCA com elevação persistente de ST (SCACSST), sendo que a avaliação completa possibilita melhor embasamento para tomada de decisão.^76 – 80^ Detalhes sobre as evidências e racional de classificação dos principais padrões eletrocardiográficos estão descritos em artigo específico elaborado por parte dos autores desta diretriz.^78^

**Tabela 7:** Alterações eletrocardiográficas diagnósticas de síndrome coronariana aguda (quando associadas a quadro clínico compatível)^56 , 77 – 80^

SCA com elevação persistente de ST (SCACSST) sem bloqueio de ramo esquerdo
*Elevação nova do segmento ST (aferida no ponto J) em pelo menos 2 derivações contíguas com os seguintes pontos de corte:* *≥ 2,5 mm nos homens < 40 anos em V2 e V3* *≥ 2 mm nos homens ≥ 40 anos em V2 e V3* *≥ 1,5 mm nas mulheres em V2 e V3* ≥ 1 mm nas demais derivações (independentemente de idade ou sexo)* *No caso de derivações complementares (V7, V8, V9, V3R, V4R), elevação ≥ 0,5 mm em pelo menos 2 derivações contíguas pode ser considerada no diagnóstico. Elevações limítrofes em DI e aVL devem ser valorizadas, especialmente se depressão recíproca do ST nas derivações inferiores.
Síndrome coronariana aguda com elevação persistente de ST (SCACSST) em paciente com bloqueio de ramo esquerdo*
• Elevação concordante do segmento ST ≥ 1 mm em derivações com complexo QRS positivo • Depressão concordante do segmento ST ≥ 1 mm (Sgarbossa em V1-V3) • Elevação discordante do segmento ST ≥ 5 mm em derivações com complexo QRS negativo e/ou relação ST/S com amplitude do desvio do segmento ST de pelo menos 25% da onda S anterior** *Bloqueio de ramo esquerdo novo isoladamente não é mais considerado necessariamente critério diagnóstico para SCACSST, embora indique situação de maior risco (correlacionar clínica). **Valorizar também razão de ST/R discordante quando infradesnivelamento for pelo menos 25% da onda R anterior. No critério de Barcelona, desvio ≥ 1 mm discordante pode ser considerado em derivação com voltagem máxima (R/S) ≤ 6 mm.
**Achados compatíveis com oclusão coronária, ou seja, equivalentes à síndrome coronariana aguda com elevação persistente de ST (SCACSST)**
• Infarto "posterior" (ínfero-lateral). - Critérios a serem avaliados em V1-V3 (habitualmente há um ou mais dos critérios abaixo): Depressão do segmento ST Onda T positiva (terminal) nas derivações anteriores Onda R proeminente e ampla (> 30 ms), sendo habitualmente dominante (R>S) em V2. - Confirmado por: Elevação do segmento ST de ≥ 0,5 mm em pelo menos 1 das derivações V7-V9
• De Winter: - Ondas T altas, proeminentes e simétricas precedidas de depressão ascendente do segmento ST > 1 mm no ponto J nas derivações precordiais - Elevação do segmento ST de 0,5-1 mm pode ser observada na derivação aVR
• Ondas T hiperagudas: Ondas T amplas, simétricas e pontiagudas podem ser observadas precocemente no IAMCSST. Eletrocardiogramas seriados em intervalos muito curtos são úteis para confirmar a progressão para os critérios clássicos de elevação de ST
• Padrão de Aslanger: Elevação do segmento ST em DIII, mas não nas demais derivações inferiores (pode haver elevação de ST em aVR, além de DIII); depressão do segmento ST em qualquer das derivações entre V4 e V6 com onda T (pelo menos na sua fase terminal) positiva e elevação do segmento ST em V1 mais alta que em V2
• Distorção terminal do QRS: Ausência de uma onda S abaixo da linha isoelétrica e ausência de onda J nas derivações V2 e/ou V3
Síndrome coronariana aguda sem elevação persistente de ST, porém com critérios de maior risco ao ECG (obstrução de tronco de coronária esquerda ou multiarterial*)
• Elevação do segmento ST em aVR e/ou V1 (sem elevação contígua, mas com depressão do segmento ST em 6 ou mais derivações) *Mais frequentemente causada por isquemia subendocárdica difusa (circunferencial) e geralmente ocorre no contexto de obstrução significativa do tronco da coronária esquerda ou doença coronariana multiarterial.
**Síndrome coronariana aguda sem elevação persistente de ST, porém com critérios de maior risco ao ECG (obstrução crítica em descendente anterior proximal)**
• Síndrome de Wellens: Trata-se de síndrome clínica caracterizada por: - Ondas T bifásicas (tipo A – 25% dos casos) ou profundamente invertidas e simétricas (tipo B – 75% dos casos) nas derivações V2 e V3 (podem se estender de V1 até V6) - Ausência de ondas Q - Sem elevação significativa do ST (isoelétrica ou elevação < 1mm) - Progressão da onda R em derivações precordiais - Angina recente (as alterações típicas do ECG são mais visualizadas após melhora da dor)
**SCA sem elevação persistente de ST, porém com critérios de maior risco ao ECG (isquemia ativa)**
• Depressão do segmento ST/Inversão de onda T:* - Depressão do segmento ST horizontal ou descendente ≥ 0,5 mm no ponto J, em 2 ou mais derivações contíguas, é sugestivo de isquemia miocárdica e/ou - Onda T invertida ≥ 1 mm em complexo com onda R proeminente ou relação R/S >1 pode indicar isquemia na ausência de causas secundárias para alteração da repolarização ventricular (por exemplo, no caso de sobrecarga). Nos casos de infarto agudo do miocárdio, a onda T negativa pode ser associada a ondas Q (patológicas). * *Depressão do segmento ST e inversão de onda T têm valor diagnóstico ainda maior quando há alterações dinâmicas (se onda T negativa se tornar positiva, esse achado também é importante para o diagnóstico de SCASSST, sendo chamado de pseudonormalização).*

Obs.: Casos de elevação transitória do ST (por exemplo, vasoespasmo) devem ser abordados de forma diferenciada dos casos de Síndrome coronariana aguda com elevação persistente de ST.

Importante reforçar que os casos de onda T invertida e infradesnível do segmento ST só devem ser considerados como um diagnóstico de síndrome coronariana aguda sem supradesnivelamento do segmento ST (SCASSST) na ausência de outras explicações, incluindo ausência de critérios para IAMCSST (lembrar que é comum ter imagem em espelho no IAMCSST). A presença, extensão, persistência e magnitude do infradesnível ST na admissão possui um caráter prognóstico importante na definição do tratamento invasivo precoce.^77 – 80^

Além do ECG diagnóstico e do ECG não diagnóstico, a presente diretriz optou por incluir a categoria de ECG "preocupante" (duvidoso/limítrofe), para que esse tipo de situação (comum nos erros de tomada decisão) tenha um foco especial. O objetivo de criar essa categoria é reduzir falhas na conduta de uma situação tempo-sensível e trazer mais segurança, com medidas específicas recomendadas para esses casos desafiadores ( Tabela 8 ).

**Tabela 8:** Medidas rotineiras para casos de eletrocardiograma duvidoso/limítrofe

1) A primeira medida em situações de dúvida ao ECG deve ser a solicitação imediata de avaliação do ECG por um ou mais médicos experientes. Esse primeiro passo definirá se, de fato, o ECG é limítrofe ou a dúvida foi decorrente da limitação de experiência do médico assistente;
2) Quando disponível, o ECG prévio deve ser comparado ao ECG da fase aguda, pois pode auxiliar na identificação de novas alterações, desde que não retarde o início do tratamento – especialmente importante nos casos que já apresentam alteração de repolarização no ECG basal;
3) Outro ponto fundamental é a adequada correlação clínica, pois o valor preditivo de qualquer exame, incluindo o eletrocardiograma, depende também da probabilidade clínica pré-teste;
4) Finalmente, casos confirmados de ECG limítrofe (após validação de médico experiente) e/ou de instabilidade clínica (por exemplo, sintomas persistentes) demandam não apenas a manutenção da monitorização do paciente, mas também a realização de eletrocardiogramas seriados e avaliação rápida para outros diagnósticos diferenciais (ecocardiograma beira-leito costuma ser um exame adicional especialmente útil nessas situações).

#### 3.4.4. Diagnósticos Diferenciais ao Eletrocardiograma

##### 3.4.4.1. Alterações Primárias e Secundárias de Repolarização

A morfologia da repolarização ventricular, que é representada pelo segmento ST, onda T e ocasionalmente pela onda U, depende diretamente da despolarização ventricular, devendo sempre ser interpretada no contexto morfológico do QRS precedente. Alterações que ocorrem devido à isquemia (ou outro tipo de dano miocárdico intrínseco) são denominadas alterações primárias da repolarização, ao passo que, quando as alterações de repolarização se apresentam precedidas de distúrbios próprios da despolarização, como nos bloqueios de ramo, pré-excitação ventricular, marcapasso ventricular e sobrecarga ventricular esquerda, são chamadas de alterações secundárias da repolarização.^13 , 81^ O bloqueio de ramo direito (BRD) não limita o diagnóstico do IAMCSST como acontece no bloqueio de ramo esquerdo (BRE), mas devemos ter cautela no diagnóstico do infradesnível, pois é habitual no BRD a presença de infradesnivelamento do segmento ST de V1 a V4 (alterações secundárias da repolarização).

Os critérios eletrocardiográficos adotados como "padrão" para diagnóstico de isquemia coronária se referem a eletrocardiogramas com QRS estreito, ou seja, sem distúrbios da condução ventricular.^77 , 82^ Além da magnitude da elevação e do território afetado (derivações contíguas), os casos de supradesnível decorrente da chamada corrente de lesão (isquemia/infarto transmural) costumam ter as características apresentadas abaixo.

1. Padrão convexo (embora possa ser côncavo em sua fase inicial);
2. Evolução com alterações dinâmicas ao ECG seriado (incluindo desenvolvimento de onda Q patológica);
3. "Imagem em espelho", ou seja, outras alterações na repolarização ventricular nas derivações opostas.

Além dos critérios do ECG, há necessidade de correlação clínica (sintomas de SCA). A causa mais importante de elevação do segmento ST neste cenário é o IAM, que deve ser prontamente reconhecido, pois, quanto mais rápido for instituído o tratamento, menor será a morbimortalidade. Entretanto, é necessário conhecer inúmeras outras situações que podem apresentar supradesnivelamento do segmento ST^83^ ( Tabela 9 ) e, para tentar diferenciá-las ( Tabela 10 ), é imprescindível analisar as características eletrocardiográficas dentro do contexto clínico do paciente com dor torácica.

**Tabela 9:** Causas de supradesnível do segmento ST

Principais causas de supradesnivelamento do segmento ST
- Infarto agudo do miocárdio
- Bloqueio de ramo esquerdo
- Pericardite
- Hipertrofia ventricular esquerda
- Repolarização precoce
- Angina vasoespástica (usualmente supra transitório)
- Hipercalemia
- Síndrome de Takotsubo
- Marca-passo
- Síndrome de Brugada
- Pré-excitação ventricular
- Aneurisma de ventrículo esquerdo

**Tabela 10:** Diagnóstico diferencial entre infarto agudo do miocárdio, pericardite e repolarização precoce

ECG	IAM com supra	Pericardite aguda	Repolarização precoce
Morfologia do segmento ST	Convexidade para cima (pode ser côncavo nas fases iniciais, mas evolui se não tratado)	Concavidade para cima	Concavidade para cima
Imagem em espelho (infradesnível de ST em derivações opostas ao supra)	Presente na maioria dos casos	Ausente	Ausente
Ondas Q patológicas	Presentes em boa parte dos casos	Ausentes	Ausentes
Localização do supredesnível	Parede envolvida com o IAM	Difuso (costuma poupar V1 e aVR)	Mais frequente em derivações precordiais
Infradesnível do segmento PR	Normalmente ausente	Presente	Ausente
Inversão da onda T	Ocorre na vigência do supra	Ocorre após normalização do segmento ST	Sem mudança da onda T ao repouso
Relação amplitude do ST/onda T (diferenciar repolarização precoce de pericardite)	Não se aplica	≥ 0,25	< 0,25 (onda T costuma ter maior amplitude)

ECG: eletrocardiograma; IAM: infarto agudo do miocárdio. No caso da pericardite, apesar de o eletrocardiograma, via de regra, não respeitar a limitação dos territórios coronários e ter características específicas de alterações no segmento ST/onda T de concavidade superior, há casos excepcionais em que a pericardite (ou miopericardite) pode ser localizada.

##### 3.4.4.2. Outros Diagnósticos ao Eletrocardiograma

Além de informações para o diagnóstico de SCA, o ECG pode trazer informações relevantes para o diagnóstico de outras causas importantes de dor torácica:

- **Tromboembolia pulmonar aguda** : a taquicardia sinusal é a alteração eletrocardiográfica mais frequente. O clássico padrão S1Q3T3 (onda S larga em DI, onda Q e T invertida em D3) encontra-se ausente na maioria dos casos (esteve presente em 8,5% dos casos de TEP na emergência).^84^ Outras alterações eletrocardiográficas vistas no TEP: alterações da repolarização ventricular, BRD completo ou incompleto, qR em V1, inversão de T de V1 a V4 (associada a TEP de maior gravidade) e taquiarritmias atriais;^85^
- **Síndrome de Takotsubo ou cardiomiopatia de estresse** : importante diagnóstico diferencial de SCA, especialmente no contexto de mulheres na pós-menopausa, com quadro de dor torácica após estresse emocional e/ou físico. Os pacientes podem apresentar alterações eletrocardiográficas marcantes, como elevação do segmento ST em 44%, inversão de onda T em 41% e prolongamento do intervalo QT, que são frequentes. Infradesnível do segmento ST (8 %) e BRE (5%) são bem mais raros.^86^

Outros diagnósticos eletrocardiográficos, como bradi ou taquiarritmias, podem estar presentes em situações de isquemia miocárdica, porém, são inespecíficos. Dessa forma, embora nesses casos possa ser estabelecido um diagnóstico eletrocardiográfico (arritmia), isso não deve impedir a investigação de SCA, caso haja suspeita clínica, pois ambas podem ocorrer de forma concomitante (isquemia pode desencadear arritmia e vice-versa). Do ponto de vista de investigação para SCA, esse tipo de achado isoladamente ao ECG é considerado "não diagnóstico" – embora condutas apropriadas para bradi ou taquiarritmia possam ser estabelecidas.^87^

##### 3.4.4.3. Eletrocardiograma Não Diagnóstico

Em menos de 5% dos protocolos de dor torácica, o ECG apresenta critérios diagnósticos inequívocos. Nesses casos, se o quadro clínico do paciente for compatível, deve-se definir o diagnóstico específico (por exemplo, IAMCSST) e seguir a respectiva rota de tratamento, conforme diretrizes específicas.^88 , 89^ Entretanto, em mais de 90% dos protocolos, especialmente em pacientes com sintomas já resolvidos antes da admissão, o ECG se mostra "não diagnóstico" para isquemia aguda. Essa situação dá uma certa tranquilidade para o médico realizar uma avaliação clínica mais pormenorizada e definir melhor o quadro e possíveis diagnósticos diferenciais, inclusive com reavaliações em diferentes momentos.

**Figura 4:**
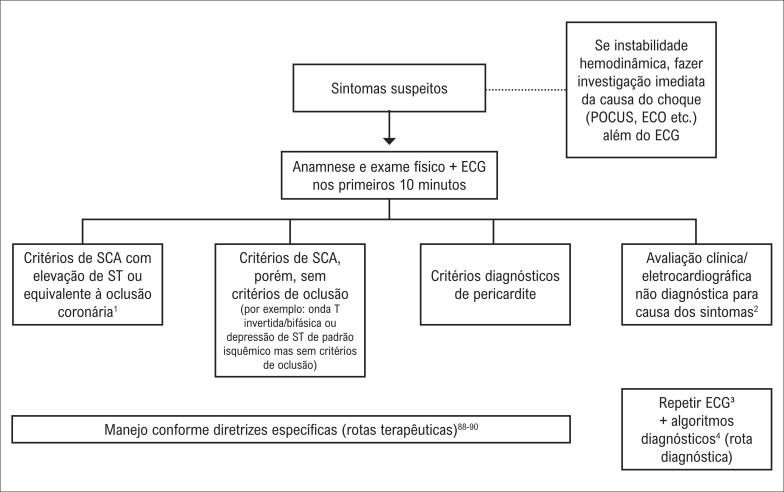
Classificação inicial do eletrocardiograma no protocolo de dor torácica. POCUS: point-of-care ultrasound; ECO: ecocardiograma; ECG: eletrocardiograma; SCA: síndrome coronariana aguda; TEP: tromboembolismo pulmonar. ^1^Ver Tabela 7 . ^2^No caso de ECG que define diagnóstico de situações que podem ocorrer de forma concomitante à SCA (por exemplo, arritmias), deve-se considerar investigação adicional de SCA (por exemplo, isquemia miocárdica pode ser causa de arritmias, assim como arritmia miocárdica pode levar à isquemia por desequilíbrio entre oferta e consumo). ^3^Repetir ECG (incluir derivações complementares) se: 1) ECG limítrofe/duvidoso e/ou sintomas persistentes (ECG a cada 10-20 minutos); 2) recorrência de sintomas ou piora clínica (imediatamente na recorrência/piora clínica); 3) troponina positiva (acima do percentil 99); 4) suspeita clínica mantida mesmo com troponina negativa (por exemplo, hipótese de angina instável). ^4^Na avaliação clínica, além do julgamento subjetivo, deve-se lançar mão de algoritmos centrados no paciente de acordo com suspeita (s) diagnóstica (s) (por exemplo, SCA, TEP, dissecção de aorta).

A repetição seriada do ECG é mandatória quando houver recorrência da dor precordial ou qualquer mudança no quadro clínico (repetição imediata); além disso, nos casos com sintomas persistentes, deve-se repetir o ECG a cada 10 a 20 minutos. As alterações dinâmicas do segmento ST e da onda T são extremamente valiosas na identificação de pacientes de alto risco com isquemia miocárdica e devem der buscadas de maneira ativa.^72 , 87^ Nas Figuras S1 e S2 (suplemento) podemos observar a alteração dinâmica do segmento ST ao evidenciar isquemia/corrente de lesão subendocárdica intermitente, enquanto a Figura 4 apresenta um fluxo para classificar o paciente de acordo com o ECG.

### 3.5. Como é Definido o Diagnóstico de Síndrome Coronariana Aguda?

O diagnóstico de SCA utiliza, como regra, os elementos abaixo:

1. Apresentação clínica;
2. ECG;
3. Marcador de injúria miocárdica (troponina).

De maneira geral, quando há dois elementos "positivos" (por exemplo, quadro clínico + ECG com padrões diagnósticos bem definidos), é possível concluir o diagnóstico de SCA.

#### 3.5.1. Critérios Atuais para Diagnóstico de Angina Instável

O advento da troponina de alta sensibilidade levou a redução expressiva no diagnóstico de angina instável e na gravidade destes pacientes, entretanto, casos que apresentam quadro clínico "típico" isoladamente (por exemplo, dor cardíaca isquêmica sem alterações de ECG e de troponina) e que não tenham diagnóstico alternativo, podem ser classificados como AI e conduzidos como SCA inicialmente – embora a confirmação definitiva dependerá habitualmente de outros achados como testes não invasivos ou angiografia coronária.

Além de angina em repouso, sintomas aos esforços em crescendo, ou seja, com piora na frequência, duração ou intensidade também são compatíveis com AI e devem levantar suspeita de instabilidade da lesão coronária (pode incluir também alterações na irradiação, alterações no padrão de resposta ao uso de nitratos). Angina pós-infarto e angina de início recente (habitualmente classe III, pela classificação da Canadian Cardiovascular Society, em um período inferior a 2 meses) também são manifestações de AI.

#### 3.5.2. Como Classificar a Síndrome Coronariana Aguda pelo Eletrocardiograma?

Qualquer que seja a manifestação clínica suspeita (critério de abertura do protocolo de dor torácica), o ECG é o primeiro método diagnóstico a ser realizado. Tendo em vista que o ECG realizado nos primeiros 10 minutos é o "divisor de águas" inicial, é importante fazer uma classificação do traçado do ECG que vai além da observação dos critérios de elevação de ST ( Tabelas 7 a 10 ). Dessa forma, embora os critérios de SCA com elevação de ST ainda devam ser valorizados na tomada de decisão, a diretriz atual recomenda que sejam buscadas outras alterações eletrocardiográficas, que não só possam definir o diagnóstico de SCA, mas também identificar alterações compatíveis com oclusão de coronária aguda, mesmo na ausência dos achados clássicos da SCA com elevação de ST ( Tabela 11 ).

**Tabela 11:** Classificação do eletrocardiograma

	Classe de recomendação	Nível de evidência
Pacientes com quadro clínico suspeito e alterações diagnósticas ao eletrocardiograma devem seguir diretrizes de tratamento específicas, de acordo com o diagnóstico estabelecido.	I	A
Além dos critérios de supradesnível de ST, devem ser procurados os "equivalentes de supradesnível ST" (ou de oclusão coronária aguda).	I	B
Casos em que eletrocardiograma NÃO permita o estabelecimento do diagnóstico com segurança (ECG "não diagnóstico") devem seguir rota de investigação diagnóstica da forma mais eficiente possível (de acordo com os recursos disponíveis).	I	B
Na rota diagnóstica, os casos com ECG limítrofe ou de difícil interpretação (ECG "preocupante") e/ou casos que apresentem critérios clínicos de instabilidade (Tabela 12) devem repetir ECG a cada 10 a 20 minutos (com derivações complementares e apoio de segundo médico experiente na interpretação), além de investigação ativa até definir causa da dor torácica.	I	C

ECG: eletrocardiograma.

**Figura 5:**
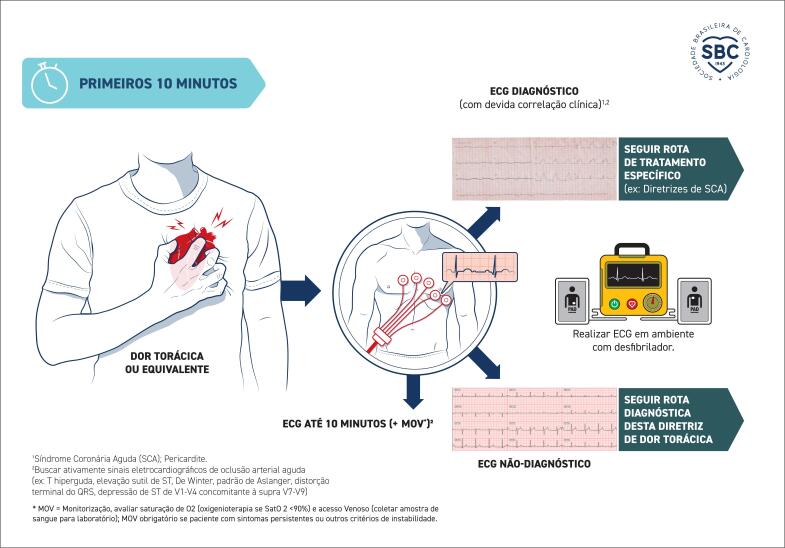
Passo 1: atendimento inicial do protocolo de dor torácica (primeiros 10 minutos).

A Figura 5 resume as condutas do atendimento inicial de dor torácica (Passo 1 da Figura Central ).

**Figure:**
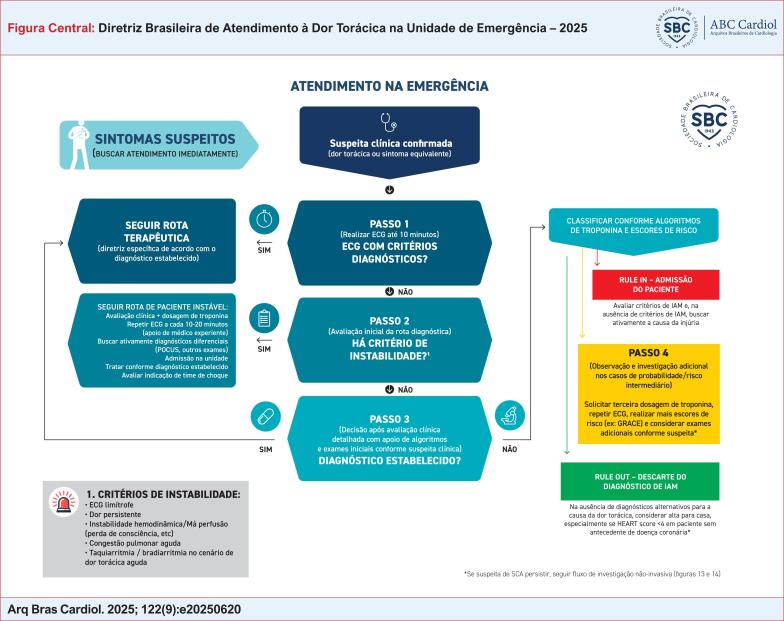
ECG: eletrocardiograma; POCUS: point-of-care ultrasound; IAM: infarto agudo do miocárdio.

## 4. Rota de Investigação Diagnóstica após o ECG Inicial

**Figura 6:**
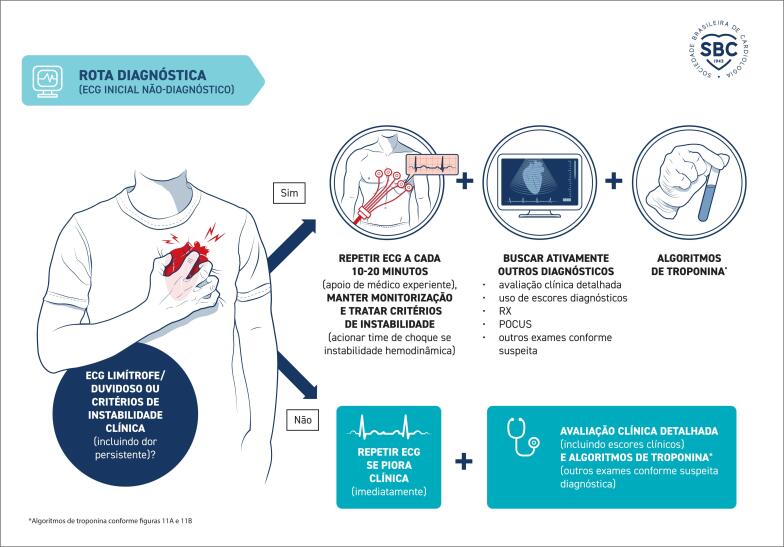
Passo 2: avaliação inicial da rota diagnóstica.

**Figura 13:**
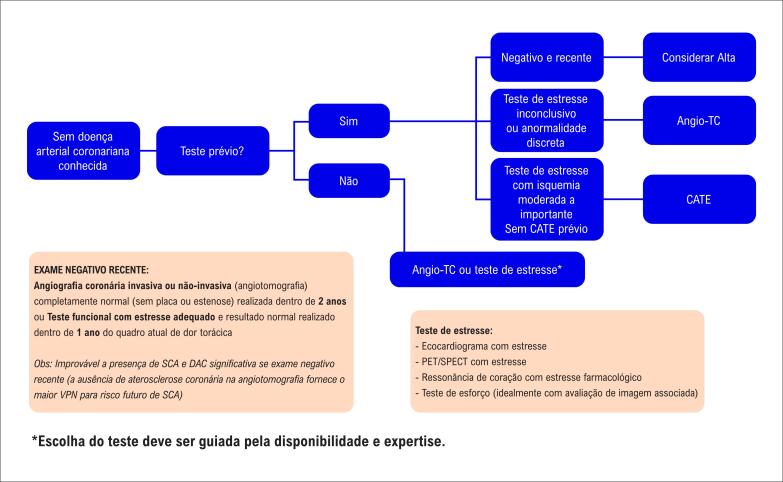
Fluxograma de investigação não invasiva em paciente sem doença arterial coronariana conhecida. SCA: síndrome coronariana aguda; DAC: doença arterial coronariana; CATE: cateterismo cardíaco; PET: tomografia por emissão de pósitrons; SPECT: tomografia computadorizada por emissão de fóton único; Angio-TC: angiotomografia coronária por tomografia computadorizada; VPN: valor preditivo negativo.

A investigação diagnóstica segue uma abordagem de até 4 passos ( Figura Central ). O primeiro passo faz parte do atendimento geral inicial, em que avaliação clínica inicial + ECG dentro de 10 minutos pode estabelecer diagnósticos em uma minoria dos casos ( Figuras 4 e 5 ) e rapidamente direcionar para uma rota terapêutica, conforme diagnóstico estabelecido (por exemplo, SCA com elevação de ST). Nos casos em que ECG não for diagnóstico (maioria dos casos), o paciente será direcionado para rota diagnóstica, em que os elementos analisados (incluindo algoritmos) irão direcionar a tomada de decisão no PS (Passo 3 da Figura Central )). Abaixo, um resumo de quatro passos essenciais no protocolo de dor torácica:

- Passo 1 (avaliação inicial): avaliação clínica geral (sinais e sintomas) + ECG nos primeiros 10 minutos para definir se preenche critérios diagnósticos (por exemplo, SCA, pericardite) e direcionar investigação subsequente nos casos sem diagnóstico estabelecido;
- Passo 2 (definir risco e probabilidade pré-teste inicial): a principal avaliação neste segundo passo seria definir se paciente apresenta sintoma persistente e/ou ECG "preocupante" e/ou instabilidade hemodinâmica uma vez que estes casos devem ter avaliação diferenciada, com repetição de ECG a cada 10 a 20 minutos e exame de imagem beira-leito (considerar *point-of-care ultrasound* [POCUS] e/ou ecocardiograma emergencial). Nos casos em que ECG não diagnóstico e avaliação clínica conjunta não forem suficientes para afastar com segurança síndromes agudas (por exemplo, SCA sem elevação de ST, TEP, síndrome aórtica aguda [SAA]), a investigação clínica deve ser feita de maneira ativa (passo 3), com avaliação de risco/probabilidade pré-teste das principais hipóteses diagnósticas (idealmente, por escores clínicos);
- Passo 3 (exames complementares, conforme hipótese diagnóstica e probabilidade pré-teste): além da avaliação do risco e probabilidade diagnóstica que são construídas desde o atendimento inicial (e podem mudar ao longo do tempo), neste terceiro passo, deve-se solicitar exames complementares, conforme hipótese diagnóstica (SCA, TEP, SAA). A probabilidade pré-teste definirá o melhor tipo de teste a ser realizado e como interpretá-lo (por exemplo, se suspeita de TEP, mas baixa probabilidade pré-teste, o D-dímero seria o exame indicado como finalidade de descartar o diagnóstico). A troponina (de preferência, de alta sensibilidade) é o exame mais importante para investigação de SCA quando avaliação clínica + ECG foram não diagnósticos (rotineiro, nesses casos e, mesmo quando não definem o diagnóstico, podem apoiar a tomada de decisão em seus algoritmos de decisão);
- Passo 4 (investigação anatômica e/ou funcional): esse quarto passo é realizado em uma minoria dos casos, sendo reservado para situações em que após avaliação pelos primeiros 3 passos, afastou-se diagnósticos diferenciais, mas ainda persiste incerteza quanto ao diagnóstico de SCA e/ou risco não é baixo o suficiente para liberar o paciente (por exemplo, algoritmo de troponina cardíaca de alta sensibilidade com valor intermediário). Nesses casos, exames adicionais podem ser solicitados antes da liberação para casa ou para realização de forma ambulatorial precoce (ver seções 4.3 e 4.4, e Figuras 13 e 14 ).
- Dessa forma, a rota diagnóstica é necessária para os casos em que atendimento inicial (clínica + ECG) não permitiu definir a causa dos sintomas. A rota se inicia pela avaliação clínica detalhada, e a primeira definição a ser realizada é se o paciente apresenta critérios de instabilidade uma vez que, nos casos instáveis, a rota diagnóstica deve ser acelerada e incluir exames de imagem beira-leito, além de repetição frequente de ECG ( Figura 6 resume o passo 2 da Figura Central ).

**Figura 14:**
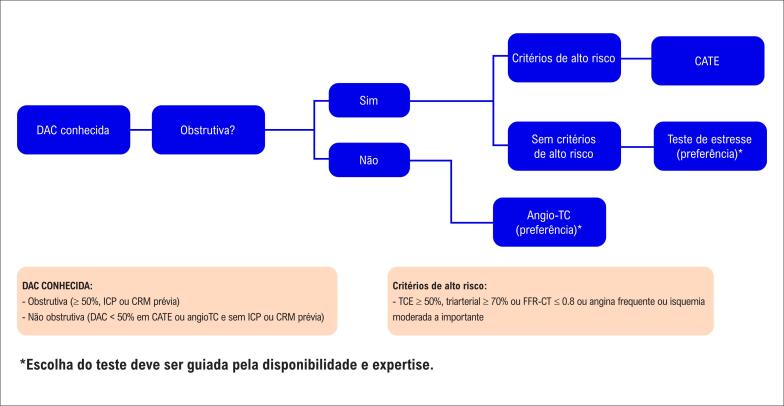
Fluxograma de investigação não invasiva em paciente com doença arterial coronariana conhecida. DAC: doença arterial coronariana; CATE: cateterismo cardíaco; ICP: intervenção coronariana percutânea; CRM: cirurgia de revascularização do miocárdio; Angio-TC: angiotomografia coronária por tomografia computadorizada; TCE: tronco da coronária esquerda; FFR-CT: reserva de fluxo fracionada obtida por tomografia computadorizada.

### 4.1. Abordagem do Paciente com Critério de Instabilidade

Na avaliação inicial do paciente com dor torácica na emergência, devemos buscar indícios de instabilidade ( Tabela 12 ) que irão denotar, necessariamente, pior prognóstico. Nesse cenário, uma abordagem acelerada, visando um diagnóstico ainda mais rápido, pode ser peça fundamental na intervenção terapêutica com impacto, inclusive, em redução de mortalidade ( Tabela 13 ).^91^

**Tabela 12:** Situações clínicas indicativas de instabilidade

Dor torácica persistente
Hipotensão arterial
Taquiarritmia / bradiarritmia
Dispneia intensa / edema agudo de pulmão
Diaforese
Extremidades frias
Pulsos distais reduzidos
Rebaixamento do nível de consciência / confusão mental
Redução do débito urinário
Aumento do tempo de enchimento capilar (> 3 segundos)

* Nos últimos anos, houve aumento da disponibilidade da ecocardiografia e do point-of-care ultrasound (POCUS) no ambiente de emergência e terapia intensiva. Essa ferramenta, quando bem utilizada, pode auxiliar no rápido diagnóstico diferencial de diversas condições graves.

**Tabela 13:** Recomendações para o paciente com dor torácica e instabilidade hemodinâmica

	Classe de recomendação	Nível de evidência
Na identificação de características clínicas sugestivas de instabilidade, as principais causas devem ser buscadas inicialmente através de história e exame físico sumário.	I	C
Especialmente nas situações em que o diagnóstico de choque é obscuro após avaliação clínica, está indicada a realização de ecocardiografia com urgência e/ou ultrassom *point of care* por profissional médico habilitado.	I	C

Além dos critérios clínicos de instabilidade ( Tabela 12 ), pacientes com ECG "preocupante" devem ter investigação acelerada. Em todos esses casos – critérios clássicos de instabilidade e ECG "preocupante" –, além da investigação acelerada com métodos de imagem beira-leito, deve-se repetir o ECG precocemente, para avaliar alterações dinâmicas e ajudar na rápida definição diagnóstica.

Importante reforçar que em situações ameaçadoras à vida, o paciente geralmente se apresenta muito ansioso, com dispneia intensa e diaforese. No protocolo de dor torácica, as principais condições que devem ser investigadas no paciente com instabilidade são: SCA, dissecção de aorta, TEP, tamponamento cardíaco, pneumotórax hipertensivo e ruptura de esôfago. Além da propedêutica básica clássica (anamnese, exame físico, ECG e radiografia de tórax), o ecocardiograma/POCUS é uma ferramenta essencial no diagnóstico diferencial destes pacientes:

- **Choque cardiogênico (falência ventricular):** situação mais comum de instabilidade não arrítmica durante avaliação de um protocolo de dor torácica (primeiras 24 horas). Os principais preditores de choque cardiogênico em doentes com SCA são: idade > 70 anos, hipotensão arterial, classificação de Killip ≥ 2 e taquicardia.^92^ Ao ecocardiograma, observa-se importante disfunção sistólica do ventrículo esquerdo (VE), dilatação de veia cava inferior (sem variação) e presença de linhas B à ultrassonografia pulmonar. A identificação precoce é importante para rápida revascularização e aplicação de dispositivos de assistência ventricular – medidas com potencial de reduzir a mortalidade em doentes com choque cardiogênico.^93^ Pode haver choque cardiogênico no IAM do ventrículo direito (VD), e os achados costumam ser disfunção sistólica do VD, com dilatação de veia cava inferior (sem variação), mas SEM linhas B à ultrassonografia pulmonar;
- **Complicações mecânicas do infarto:** situações habitualmente mais tardias, ocorrendo, geralmente, após dias de internação, e não no atendimento inicial da dor torácica. Ocorrem especialmente em doentes não revascularizados, que subitamente desenvolvem instabilidade hemodinâmica e que modificam seu exame físico (sopro novo, turgência jugular, novas crepitações pulmonares). O ecocardiograma pode apresentar alterações de acordo com tipo de complicação mecânica: derrame pericárdico (ruptura de parede livre), insuficiência mitral grave (ruptura de cordoalha ou músculo papilar) ou *shunt* no septo interventricular, com dilatação do VD (comunicação interventricular [CIV]);
- **Dissecção de aorta:** ^94^ a avaliação diagnóstica da dissecção de aorta, incluindo algoritmos de probabilidade clínica, está descrita no item 4.2.2. Quando a instabilidade está presente, devemos suspeitar de ruptura de aorta, insuficiência aórtica aguda ou tamponamento cardíaco, situações com alta mortalidade. Ao ecocardiograma, a janela supraesternal pode auxiliar na identificação de dissecção, assim como o paraesternal longitudinal. A presença de um *flap* diante de um paciente com quadro clínico compatível corrobora o diagnóstico. Nem sempre o *flap* estará visível. Nesse caso, dados indiretos como a visualização de dilatação da aorta reforçam a necessidade de seguimento da investigação diagnóstica de dissecção. De uma forma geral, o ecocardiograma transtorácico apresenta acurácia limitada para o diagnóstico SAA; portanto, esse método não é utilizado para estabelecer o diagnóstico final, apesar de informar acuradamente a presença de insuficiência aórtica e a função sistólica do VE. Já o ecocardiograma transesofágico mostra acurácia acima de 90%, com valor preditivo negativo próximo a 100%, sendo método preferencial em pacientes instáveis com impossibilidade de realizar a angiotomografia;
- **TEP:** é outra causa importante de dor torácica, que pode gerar instabilidade hemodinâmica. Assim como na SAA, a avaliação diagnóstica do TEP, incluindo algoritmos de probabilidade clínica, está descrita no item 4.2.3. Na presença de instabilidade, o TEP é de alto risco e reflete importante sobrecarga do VD, que irá comprimir o VE, gerando a queda da pressão arterial. Ao exame clínico, encontraremos turgência jugular e ausculta pulmonar sem evidências de congestão. O ecocardiograma irá demonstrar importante dilatação do VD com déficit contrátil importante. Podemos observar hipercontratilidade apical com acinesia das demais porções (sinal de McConnel). Nos casos de instabilidade, haverá importante sobrecarga pressórica do VD sobre o VE, podendo deixá-lo com formato de "D". Em pacientes instáveis, com suspeita clínica de TEP, se não houver condições para realizar a angiotomografia pulmonar, a evidência de sobrecarga de VD ao ecocardiograma (afastando outras causas de instabilidade) pode ser usada como evidência indireta para indicar tratamento no TEP instável. O ecocardiograma transesofágico, ao permitir visualizar o trombo na artéria pulmonar, é capaz de estabelecer o diagnóstico direto de TEP em pacientes hemodinamicamente instáveis;
- **Tamponamento cardíaco:** no tamponamento cardíaco, devemos lembrar que o diagnóstico deve ser clínico, onde habitualmente observaremos paciente com choque, turgência jugular e bulhas abafadas. O pulso paradoxal é um achado comum e reflete a interdependência ventricular. O ecocardiograma é útil para avaliar volume do derrame pericárdico e sinais de restrição ao enchimento ventricular. Todo paciente com choque e qualquer grau de derrame pericárdico deve ser abordado como possível tamponamento cardíaco (a velocidade da instalação do derrame pode ser mais importante do que o volume do mesmo na ocorrência do tamponamento);
- **Pneumotórax hipertensivo:** o diagnóstico do pneumotórax hipertensivo também é clínico e deve ser reconhecido rapidamente, visto que gera importante instabilidade, podendo causar morte. Geralmente, acomete casos de trauma torácico, pós-procedimento (ex: acidente de punção venosa central) e/ou pacientes mais jovens ou portadores de pneumopatias. Nos casos duvidosos, o ultrassom irá demonstrar ausência de deslizamento pleural e densificação das linhas A;
- **Ruptura de esôfago (síndrome de Boerhaave):** a ruptura de esôfago possui diagnóstico mais difícil. A principal chave para o diagnóstico é a identificação de enfisema subcutâneo e pneumomediastino em paciente com suspeita clínica (por exemplo, paciente com aumento de temperatura, vômitos, que evoluiu com dor súbita no tórax e/ou abdome superior e instabilidade hemodinâmica). A radiografia de tórax e a tomografia são exames importantes na investigação diagnóstica.

#### 4.1.1. Rotina Ecocardiográfica em Paciente com Dor Torácica e Instabilidade

O exame ecocardiográfico deve ser realizado nesses pacientes, buscando a causa da instabilidade. O ecocardiograma permitirá a avaliação da função ventricular global e regional, tamanho das câmaras cardíacas, doenças valvares, presença de doença pericárdica, identificação de doenças da aorta, avaliação da volemia e identificação de acometimento pulmonar (ultrassom pulmonar).^95 , 96^

Com isso, a rotina do exame ultrassonográfico desses pacientes recomendada é:

1. Janela paraesternal: avaliar a função ventricular esquerda, acometimento mitral, verificar diâmetro da aorta e tamanho do VD;
2. Apical 4 câmaras: avaliar função ventricular esquerda e direita, acometimento valvar e tamanhos cavitários;
3. Subcostal: avaliar função ventricular esquerda e direita, derrame pericárdico e volemia;
4. Supraesternal: útil para avaliar arco aórtico (por exemplo, suspeita de dissecção de aorta);
5. Linha hemiclavicular e axilar anterior: ultrassonografia pulmonar, buscando congestão pulmonar e/ou pneumotórax.

**Figura 7:**
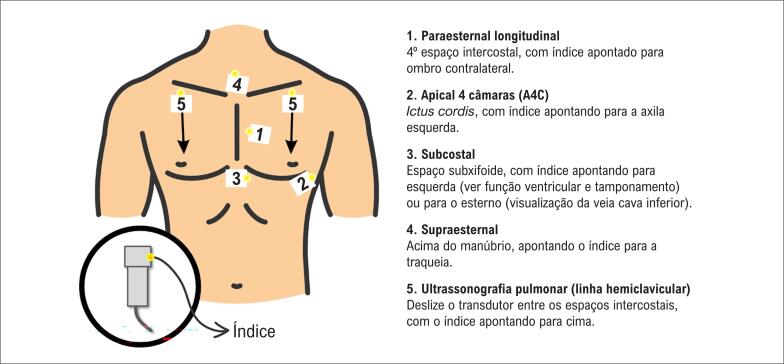
Janelas ecocardiográficas.

As Figuras 7 e 8 demonstram as janelas ecocardiográficas e as respectivas estruturas identificadas.

**Figura 8:**
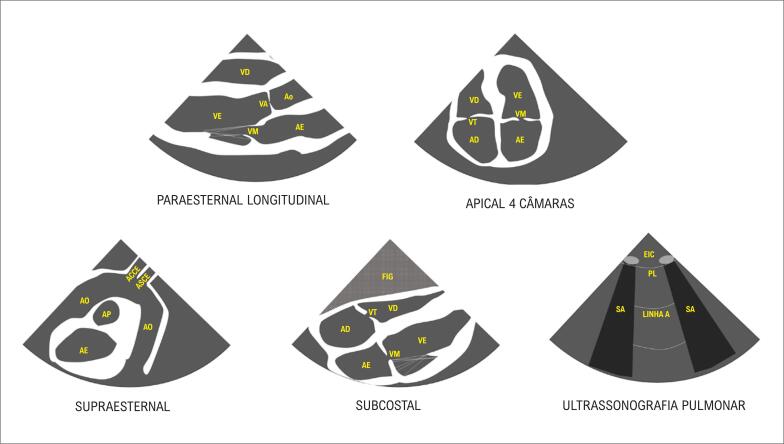
Identificação de estruturas de acordo com as janelas avaliadas. VE: ventrículo esquerdo; VD: ventrículo direito; AO: aorta; AE: átrio esquerdo; VM: valva mitral; VA: valva aórtica; VT: valva tricúspide, AP: artéria pulmonar; ACCE: artéria carótida comum esquerda; ASCE: artéria subclávia esquerda; FIG: fígado; EIC: espaço intercostal; SA: sombra acústica; PL: pleura.

No suplemento, há exemplos de algumas condições patológicas identificadas pelo exame ultrassonográfico (Figuras S3 a S6) e um algoritmo de avaliação do paciente com instabilidade ( Figura S7).

### 4.2. Escores Clínicos de Risco e Probabilidade Diagnóstica

A avaliação de dor torácica engloba uma extensa interpretação de preditores e achados clínicos para a avaliação de um diagnóstico mais provável. Diversas formas de classificação da dor torácica foram propostas (típica *versus* atípica, tipos A/B/C/D); entretanto, os autores da atual diretriz recomendam a classificação em 3 grupos: 1) sintoma altamente suspeito de SCA (por exemplo, dor isquêmica cardíaca), sintoma moderadamente suspeito (por exemplo, dor possivelmente isquêmica cardíaca) e sintoma pouco/nada suspeito (por exemplo, dor torácica não cardíaca). Essa classificação facilita a aplicação do escore HEART e evita termos que podem levar à falha de comunicação (por exemplo, dor atípica pode ser interpretada como dor não cardíaca). Além disso, sintomas equivalentes são contemplados quando não denominamos apenas com dor torácica. De qualquer maneira, essa classificação só representa parte da propedêutica básica, e outros elementos devem ser avaliados de forma conjunta.

Com o objetivo de evitar que uma avaliação subjetiva – dependente da experiência do avaliador – seja o único fator na decisão diagnóstica em pacientes com dor torácica (ou equivalente) e ECG não diagnóstico, o uso de escores de avaliação de risco e probabilidade diagnóstica pode ser útil e deve ser empregado rotineiramente na sala de emergência. Na tentativa de aperfeiçoar essa avaliação com investigações mais assertivas, melhor otimização dos recursos diagnósticos e, principalmente, evitando altas inadvertidas, vários escores foram validados internacionalmente e permitem avaliar o risco e a probabilidade diagnóstica das doenças de maior impacto em morbimortalidade.

Os escores validados na prática clínica auxiliam em pacientes com suspeita de SCA, síndromes aórticas agudas (DAA mais frequentemente) e TEP. Esses devem ser usados na avaliação de dor torácica e incorporam dados relacionados aos antecedentes pessoais, sintomas, achados do exame clínico, manifestações eletrocardiográficas/radiográficas e a dosagem de biomarcadores. Algoritmos de troponina podem ser utilizados de forma concomitante a esses escores clínicos, embora seja controverso o ganho incremental da adição de escores clínicos de SCA (ex: HEART score) aos algoritmos de troponina cardíaca de alta sensibilidade (ver Seção 4.3).

#### 4.2.1. Escores Clínicos para Casos de Suspeita de Síndrome Coronariana Aguda

Os escores contemplados na avaliação inicial na suspeita de SCA não têm a capacidade, ou mesmo a intenção, de excluir ou confirmar sua hipótese. Entretanto, são capazes de predizer a probabilidade diagnóstica e, principalmente, estratificar o risco desses pacientes apresentarem desfechos desfavoráveis no futuro, a depender da pontuação e de sua classificação.

O escore mais bem estudado na população com dor torácica aguda foi o HEART^97 – 101^ ( Tabela 14 ), e sua utilização tem maior aplicabilidade na avaliação em pronto-atendimento ou na sala de emergência. O estudo original avaliou em seus preditores a dosagem da troponina convencional. Todavia, há estudos mais recentes, que validaram a utilização da troponina cardíaca de alta sensibilidade (TCas) na aplicação do escore HEART, uma vez que atualmente é o biomarcador preferencial. Além disso, o estudo original avaliou o risco de eventos cardiovasculares maiores em 6 semanas; entretanto, como esse risco se associa à probabilidade clínica de SCA, o resultado do HEART score pode ajudar na decisão médica sobre investigação adicional (além da troponina).

**Tabela 14:** Escore HEART

Escore HEART	
História	2 = altamente suspeita
	1 = moderadamente suspeita
	0 = pouco/nada suspeita
Eletrocardiograma	2 = depressão significativa do segmento ST
	1 = distúrbios de repolarização inespecíficos
	0 = normal
Anos (idade)	2 = ≥ 65 anos
	1 = ≥ 45 anos e < 65 anos
	0 = < 45 anos
Risco (fatores * )	2 = ≥ 3 ou história de doença aterosclerótica
	1 = 1 ou 2
	0 = nenhum
Troponina (convencional) **	2 = ≥ 3x o limite superior
	1 = 1 – 3x o limite superior
	0 = ≤ limite superior

ECG: eletrocardiograma.

* Fatores de risco: Hipertensão, hipercolesterolemia, diabetes, obesidade (IMC >30 kg/m²), tabagismo (atual ou cessação do tabagismo ≤3 meses), histórico familiar positivo precoce (primeiro grau).

** O escore HEART original utilizou troponina convencional; a sua versão modificada para troponina de alta sensibilidade, baseia-se no escore HEAR (História, Anos/Idade, Eletrocardiograma, Risco) e utiliza também pontos de corte específicos dos algoritmos de troponina de alta sensibilidade.

Outros escores, como ADAPT^102^ e EDACS,^103^ embora sejam alternativas ao escore HEART, não apresentam vantagens significativas – além disso, o HEART apresentou melhor *performance* em estudo nacional.^104^ Alguns escores de estratificação de risco e prognóstico aplicados em pacientes portadores de SCA, tais como TIMI e GRACE também foram analisados no cenário de avaliação inicial da dor torácica e demonstraram *performance* inferior ao HEART.^104^ Dessa forma, o escore HEART é o preferencial, por ter melhor acurácia e validação, assim como alta facilidade para uso na prática clínica.^105 – 107^ Importante reforçar que casos com alterações diagnósticas de ECG ou troponina (junto com quadro clínico compatível), não precisam do HEART score e devem seguir rotas de tratamento, uma vez que o diagnóstico já estaria estabelecido (ver Figuras 4 e 11A e 11 ).

**Figura 11-A:**
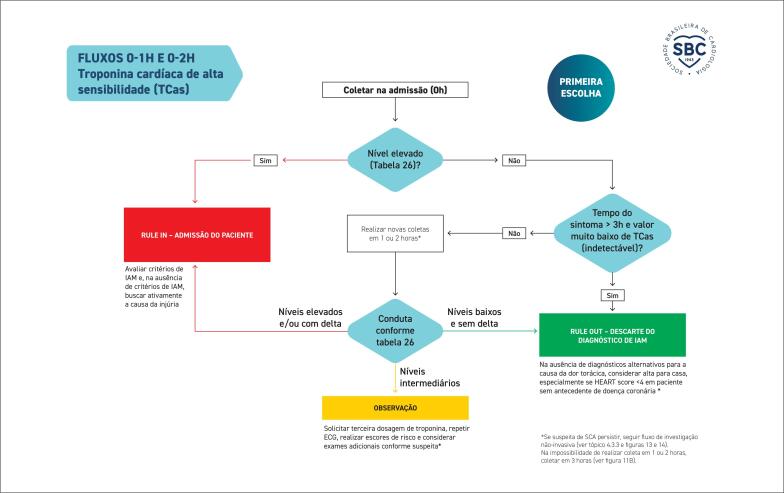
Fluxos 0-1H e 0-2H – Troponina cardíaca de alta sensibilidade (Tcas).

**Figura 11-B:**
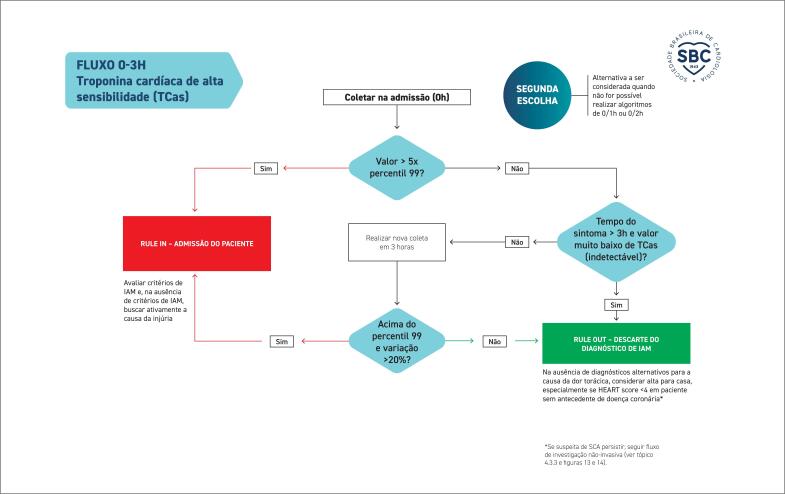
Valores de troponina a serem utilizados no fluxograma de 0-3 horas.

##### 4.2.1.1. Em Qual Paciente Devo Aplicar o Escore HEART?

O escore HEART foi desenvolvido para avaliar o risco de eventos cardiovasculares maiores em 6 semanas, em pacientes com suspeita de SCA (essa é a população que mais se beneficia do HEART *score* ). Assim, esse escore é o que mais se aplica a esta diretriz, sendo útil para ser utilizado nos casos em que o médico está no momento de decisão sobre a segurança de liberar o paciente para casa ( Tabela 15 ). Uma vez que o risco de eventos cardiovasculares em curto prazo (6 semanas) está relacionado com a probabilidade diagnóstica, o HEART *score* também é útil para apoiar a definição da probabilidade pré-teste (ajudar na decisão de exames complementares adicionais):

- HEART *SCORE* : 0 a 3 pontos (risco baixo de MACE em 6 semanas);
- HEART *SCORE* : 4 a 6 pontos (risco médio de MACE em 6 semanas);
- HEART *SCORE* : 7 a 10 pontos (risco elevado de MACE em 6 semanas).

**Tabela 15:** Estratificação de risco e probabilidade diagnóstica na avaliação de dor torácica na suspeita de síndrome coronariana aguda

	Classe de recomendação	Nível de evidência
O escore HEART é o escore clínico preferencial na avaliação de pacientes com dor torácica em investigação diagnóstica de síndrome coronariana aguda.	I	B

#### 4.2.2. Escores Clínicos para Casos de Suspeita de Síndromes Aórticas Agudas

Apesar de raras, as SAAs são condições clínicas extremamente graves e de alta letalidade. Os maiores registros internacionais descrevem uma taxa de mortalidade em torno de 1 a 2% por hora nas primeiras 48 horas.^108^ Dissecção aguda de aorta (DAA) corresponde a aproximadamente 70% das síndromes aórticas agudas.

A dissecção de aorta possui um amplo espectro de apresentações clínicas, mas a dor torácica é o sintoma mais comum, geralmente de alta intensidade, com irradiação para o dorso e sem fatores atenuantes. O alargamento do mediastino superior é o achado mais frequentemente encontrado na radiografia de tórax (60 a 90% dos casos). No entanto, é importante frisar que a normalidade desse exame não exclui o diagnóstico de dissecção aórtica.

Além da apresentação clínica heterogênea, as manifestações podem ser semelhantes às de outras causas de dor torácica.^109^ Dessa forma, a avaliação dos pacientes com suspeita de SAA é frequentemente desafiadora para o médico, que, por vezes, está sujeito a indicar um tratamento inadequado frente a uma sobreposição de sinais de sintomas, ou dispor excessivamente de exames de imagem para descartar o diagnóstico, ou mesmo dar alta hospitalar inadvertidamente. A falha no diagnóstico dessa doença tempo-sensível piora drasticamente o prognóstico e dificulta o tratamento.

Na tentativa de facilitar a avaliação inicial, reduzir a possibilidade de erros e melhorar a aplicabilidade de recursos de exames diagnósticos de imagem, foram desenvolvidos dois escores diagnósticos, o ADD-RS e o AORTA.^110 – 112^

O ADD-RS engloba 12 variáveis distribuídas em 3 categorias, como demonstra a Tabela 16 . Se o paciente apresenta quaisquer características em uma das categorias, recebe 1 ponto, podendo, portanto, pontuar até 3 pontos. Pacientes com até 1 ponto são caracterizados como baixo risco, e aqueles com 2 ou 3 pontos, como alto risco.^110 , 111^ A adição de POCUS ao escore ADD mostrou incremento na definição da probabilidade diagnóstica.^111^

**Tabela 16:** Aortic dissection detection risk score (ADD-RS)

Condições clínicas predisponentes	Características da dor	Achados no exame clínico
- Síndrome de Marfan ou outra doença do tecido conjuntivo - História familiar de aortopatia - História conhecida de valvopatia aórtica - Manipulação aórtica recente - Aneurisma de aorta torácico conhecido	Dor torácica ou abdominal descrita como: - Início súbito; - Intensidade severa; - Caracterizada como "rasgando"	- Déficit de pulso ou pressão arterial - Déficit neurológico focal (associado a dor) - Sopro de regurgitação aórtica (novo associado a dor) - Hipotensão ou choque

O AORTA por sua vez atribui pontos a seis características clínicas, conforme mostra a Tabela 17 . Pacientes com 0 a 1 pontos são classificados com baixo risco de SAA, e aqueles com 2 pontos ou mais, como alto risco. É um escore mais simplificado, com alta sensibilidade, mas ainda carece de maior validação para ter seu uso ampliado na prática clínica.^112 , 113^

**Tabela 17:** Escore AORTA – escore de probabilidade pré-teste para síndrome aórtica aguda

Característica clínica	Pontos
Hipotensão / choque	2
Aneurisma	1
Déficit de pulso	1
Déficit neurológico	1
Dor severa	1
Dor de início súbito	1

Vale ressaltar que o ADD-RS e o AORTA são escores de probabilidade pré-teste que usam apenas dados clínicos e, dessa forma, identificam aqueles pacientes com baixo ou alto risco de serem portadores de SAA. No entanto, nenhum dos dois tem acurácia suficiente para, isoladamente, definir ou descartar o diagnóstico de SAA ( Tabela 18 ).^113^

**Tabela 18:** Estratificação de risco e probabilidade diagnóstica na avaliação de dor torácica na suspeita de síndrome aórtica aguda

	Classe de recomendação	Nível de evidência
ADD-RS pode ser aplicado em pacientes com suspeita de SAA como parte da avaliação inicial.	IIa	B
POCUS feito por médico capacitado pode ser aplicado em adição ao ADD-RS para incremento na avaliação da probabilidade diagnóstica.	IIa	B
AORTA pode ser aplicado em pacientes com suspeita de SAA de forma adicional ou como alternativa ao ADD-RS.	IIb	C
ADD-RS e AORTA **NÃO** devem ser utilizados exclusivamente na avaliação inicial em pacientes com suspeita de SAA para exclusão ou confirmação diagnóstica.	III	B

POCUS: point-of-care ultrasound; SAA: síndrome aórtica aguda.

**Figura 9:**
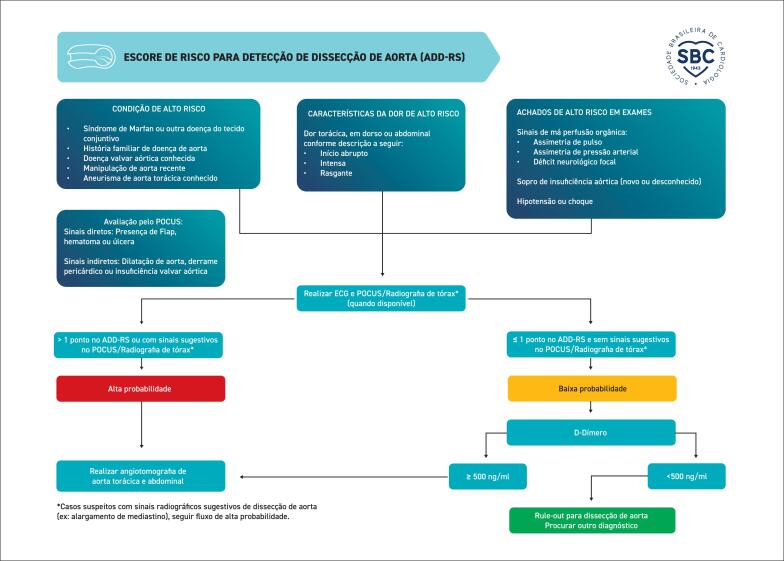
Fluxograma para investigação de síndrome aórtica aguda.

Nos casos em que há suspeita de SAA e se estabeleceu a probabilidade diagnóstica pré-teste, podemos prosseguir para a investigação adequada (passos 2 e 3), conforme fluxograma ( Figura 9 ):

- Probabilidade pré-teste baixa: realizar D-dímero (se negativo, buscar outro diagnóstico; se positivo, realizar exame de imagem*);
- Probabilidade pré-teste > baixa: realizar exame de imagem.*

*Exames de imagem habitualmente indicados para avaliar SAA: angiotomografia (mais utilizado), ecocardiograma transesofágico (quando não for possível realizar angiotomografia).

#### 4.2.3. Escores Clínicos para Casos de Suspeita de Tromboembolismo Pulmonar

No TEP, a dor torácica geralmente é súbita, associada à dispneia e sintomas pleuríticos. A identificação de fatores de risco para doença tromboembólica habitualmente auxilia no diagnóstico.

A radiografia de tórax é frequentemente anormal no TEP; entretanto, as anormalidades são geralmente inespecíficas (de qualquer forma, é um exame útil para afastar outras causas de dispneia e dor torácica, como pneumotórax). As alterações radiológicas mais frequentemente encontradas na embolia pulmonar são as áreas de atelectasia, a elevação da hemicúpula diafragmática, o derrame pleural e a dilatação do tronco e dos ramos da artéria pulmonar. Áreas de hipofluxo pulmonar segmentar (sinal de Westmark) e infiltrado pulmonar de forma triangular com a base voltada para a pleura (sinal de Hampton) são os achados mais específicos da embolia pulmonar, mas, infelizmente, são pouco sensíveis.

Em relação ao ECG, este só deve apresentar as alterações indicativas de sobrecarga de VD (inversão de onda T de V1 a V4; S1Q3T3, BRD completo ou incompleto) nos casos mais graves de TEP. Nos casos de menor gravidade, o achado mais comum seria taquicardia sinusal que, além de inespecífica, só está presente em cerca de 40% dos casos.

Tendo em vista que as principais manifestações de TEP são inespecíficas, aplicações de modelos de avaliação clínica são importantes para reduzir variações desnecessárias no julgamento subjetivo da probabilidade diagnóstica. A combinação de achados clínicos em pacientes com fatores de risco para TEP permite classificar os pacientes em categorias clínicas de probabilidades pré-teste, na tentativa de identificar qual exame complementar é mais indicado para diagnosticar ou descartar a doença.

Naqueles pacientes com suspeita de TEP, três são os escores habitualmente utilizados: escore de Wells, escore de Genebra (do inglês, Geneva) e o escore de Genebra simplificado. Vale ressaltar que esses escores não foram validados em gestantes e portadores de trombofilia.

O escore de Wells engloba uma pontuação para 7 variáveis ( Tabelas 19 e 20 ).^114^

**Tabela 19:** Escore de Wells: critérios e respectiva pontuação

Critérios	Pontos
Sinais clínicos de TVP	+3
Ausência de diagnóstico alternativo mais provável que TEP	+3
TVP ou TEP prévios	+1,5
FC > 100 bpm	+1,5
Imobilização por mais de 2 dias ou cirurgia nas últimas 4 semanas	+1,5
Hemoptise	+1
Câncer (atual tratado nos últimos 6 meses, ou se o paciente estiver em cuidados paliativos)	+1

TVP: trombose venosa profunda; TEP: tromboembolismo pulmonar; bpm: batimentos por minuto.

**Tabela 20:** Escore de Wells modificado e probabilidade/risco de tromboembolismo pulmonar, a depender da pontuação

	3 níveis	
Pontuação	Probabilidade de TEP	Probabilidade pré-teste
< 2	1 a 3%	Baixa
2-6	16 a 28%	Intermediária
> 6	> 40 %	Alta
	**2 níveis**	
**Pontuação**	**Probabilidade de TEP**	**Probabilidade pré-teste**
≤ 4	3%	Improvável
> 4	28 %	Provável

TEP: tromboembolismo pulmonar.

O escore de Genebra é mais trabalhoso para a prática clínica, mas também determina a probabilidade pré-teste dos pacientes com suspeita de TEP, assim como o de Wells.^115^ O escore de Genebra simplificado permite maior facilidade em sua aplicação e é validado em estudos com mesmos resultados que o escore de Genebra revisado ( Tabela 21 ).^116^

**Tabela 21:** Escore de Genebra revisado e simplificado e probabilidade e risco de tromboembolismo pulmonar, a depender da pontuação

Escore Genebra (do inglês, Geneva)	Revisado - pontos	Simplificado - pontos
Idade > 65 anos	1	1
TVP e TEP prévios	3	1
Cirurgia ou fratura no último mês	2	1
Neoplasia em atividade	2	1
Dor unilateral em membro inferior	3	1
Hemoptise	2	1
Dor a palpação de veias profundas de membro inferior/edema unilateral	4	1
FC 75-94 bpm	3	1
FC ≥ 95 bpm	5	2

**Pontuação – Genebra revisado**	**Probabilidade de TEP**	**Probabilidade pré-teste**
0-3	8%	Baixa
4-10	28%	Intermediária
≥ 11	> 60%	Alta

**Pontuação – Genebra simplificado (2 níveis)**	**Probabilidade pré-teste**	
**2 níveis**		
0-2	Improvável	
≥ 3	Provável	

TVP: trombose venosa profunda; TEP: tromboembolismo pulmonar; FC: frequência cardíaca; bpm: batimentos por minuto.

Alguns protocolos diagnósticos de TEP utilizam também a avaliação em dois níveis do escore de Wells e Genebra para categorizar com maior facilidade (TEP provável ou improvável, ao invés de probabilidade baixa, intermediária e alta). Considera-se TEP provável quando o paciente pontuar 5 ou mais no escore de Wells, ou 3 ou mais no Genebra simplificado. Por sua vez, é considerado TEP improvável quando a pontuação é menor ou igual a 4 para escore de Wells, ou menor ou igual a 2 para Genebra simplificado. Ambas as formas de categorização podem ser utilizadas (três níveis tradicionais e dois níveis), seguindo cada qual um protocolo específico de avaliação.^117^

Em pacientes cuja avaliação clínica inicial demonstre ao seu examinador que o caso tem como pouco provável o diagnóstico de TEP (probabilidade estimada menor que 15%; por exemplo, escore de Wells < 2), é possível utilizar o escore PERC ( *pulmonary embolism rule-out criteria* ). Trata-se de um escore de fácil aplicação, em que, na ausência de todos os itens ( Tabela 22 ), o diagnóstico pode ser afastado sem obrigatoriedade de complementação diagnóstica.^118^ Dessa forma, mais de um escore pode ser utilizado na avaliação da probabilidade diagnóstica de TEP ( Tabela 23 ).

**Tabela 22:** Pulmonary embolism rule-out criteria (PERC)

PERC
- Idade ≥ 50 anos
- Frequência cardíaca ≥ 100 bpm
- Saturação de O_2_ < 95% em ar ambiente
- Edema assimétrico dos membros inferiores
- Hemoptise
- Cirurgia ou trauma nas últimas 4 semanas
- História prévia de TVP ou TEP
- Tratamento hormonal

PERC: pulmonary embolism rule-out criteria; TVP: trombose venosa profunda; TEP: tromboembolismo pulmonar.

**Tabela 23:** Estratificação de risco e probabilidade diagnóstica na avaliação de dor torácica na suspeita de tromboembolismo pulmonar

	Classe de recomendação	Nível de evidência
Escore de Wells, Genebra revisado e Genebra simplificado devem ser aplicados em pacientes com suspeita de TEP na avaliação inicial de probabilidade pré-teste, como parte da avaliação inicial.	I	A
Escore PERC pode ser aplicado na avaliação inicial em pacientes com suspeita de TEP com baixa probabilidade pré-teste para protocolo de *rule out* .	IIa	B

TEP: tromboembolismo pulmonar; PERC: pulmonary embolism rule-out criteria.

**Figura 10:**
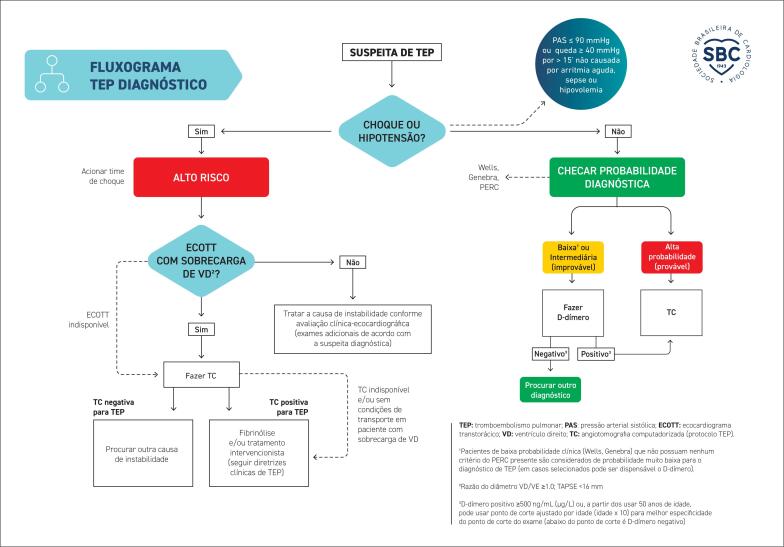
Fluxograma para diagnóstico de tromboembolismo pulmonar.

Nos casos em que há suspeita de TEP e se estabeleceu a probabilidade diagnóstica pré-teste, podemos prosseguir para a investigação adequada (passos 2 e 3), conforme fluxograma ( Figura 10 ):

- Probabilidade pré-teste baixa e PERC zero: não seria obrigatório a realização do D-dímero;
- Probabilidade pré-teste baixa (especialmente se PERC > 0) ou intermediária (TEP improvável numa avaliação em 2 níveis): realizar D-dímero tendo o valor ≥500 ng/mL (μg/L) como ponto de corte ou usar valor ajustado por idade (idade x 10 para pacientes a partir dos 50 anos); se positivo, realizar exame de imagem* (se negativo, buscar outro diagnóstico);
- Probabilidade pré-teste alta numa avaliação em 3 níveis (ou provável numa avaliação em 2 níveis): realizar exame de imagem.

*Exames de imagem habitualmente indicados para avaliar TEP: angiotomografia (mais utilizado), cintilografia ventilação-perfusão (em casos selecionados, quando houver limitação para angiotomografia), e ecocardiograma para avaliação diagnóstica de TEP (quando paciente com instabilidade e/ou sem condições para realizar angiotomografia).

#### 4.2.4. Outros Diagnósticos Diferenciais

Embora nem todos os diagnósticos diferenciais tenham escores de probabilidade validados, é importante ter em mente as principais características dessas síndromes no raciocínio clínico, como a pericardite. No contexto da dor precordial, a pericardite aguda ou a miopericardite (se pericardite associada à miocardite) causa dor precordial, habitualmente fásica (de acordo com o ciclo respiratório) e e que muda com posição (melhora com flexão anterior do tórax). Frequentemente se apresenta com febre e queda do estado geral. Casos com aumento rápido e/ou intenso do líquido pericárdico podem gerar restrição ao enchimento cardíaco (tamponamento). De uma forma geral, o diagnóstico de pericardite é definido quando dois dos quatro seguintes achados estão presentes:

1. Dor torácica característica;
2. Atrito pericárdico;
3. ECG característico;
4. Achado em exame de imagem (ecocardiograma, por exemplo).

Outras causas de dor torácica que representam situações de risco de morte (por exemplo, ruptura de esôfago, pneumotórax hipertensivo) são mais raras, mas devem ser consideradas na investigação de acordo com quadro clínico: o exame físico habitualmente é suficiente para o diagnóstico de pneumotórax hipertensivo (murmúrio vesicular abolido, hipertimpanismo, turgência jugular); no caso de rotura esofágica, dor torácica iniciada pós-vômitos e que podem evoluir com febre e hipotensão levantam suspeita desse diagnóstico (muitos casos apresentam alterações na radiografia de tórax compatíveis com pneumomediastino).

Uma vez afastados diagnósticos potencialmente fatais, situações de maior benignidade podem ser consideradas (ansiedade, dispepsia sem complicações graves etc.).

### 4.3. Algoritmos de Troponina

#### 4.3.1. Aplicação e Interpretação dos Marcadores de Injúria Miocárdica

Mais de 90% dos pacientes no protocolo de dor torácica têm o ECG "não diagnóstico". Mesmo nos casos que têm diagnóstico confirmado de SCA, cerca de metade apresenta ECG não diagnóstico.^119^ Dessa forma, quando o paciente com dor torácica tem ECG de repouso sem alterações características de SCA, deve-se dosar os marcadores de injúria miocárdica para confirmar ou afastar esse diagnóstico.

##### 4.3.1.1. Qual o Marcador de Escolha para o Diagnóstico de Infarto Agudo do Miocárdio?

Há necessidade de testes que sejam eficazes e rápidos em confirmar ou descartar a síndrome coronariana aguda. O marcador ideal deve ser o mais específico e sensível possível, ou seja, que possa detectar alterações apenas do músculo cardíaco, já em pequenas concentrações séricas.^77^

Atualmente, o marcador que melhor reúne essas características é a troponina, tanto a troponina T cardíaca (TnTc) como a troponina I cardíaca (TnIc). Em locais que têm disponibilidade de troponina quantitativa, não é recomendada a dosagem de CK-MB ou outros marcadores na avaliação diagnóstica (a troponina apresenta sensibilidade e especificidade superiores).^77 , 120^

Os ensaios para dosagem de troponina foram se aperfeiçoando, e temos várias "gerações" que variam em especial com relação à sensibilidade. A troponina cardíaca de alta sensibilidade (TCas) deve ser utilizada como marcador de injúria miocárdica preferencial em pacientes com suspeita de SCA. As TCas (também conhecidas como ultrassensíveis), como o próprio nome indica, são significativamente mais sensíveis do que as convencionais para diagnóstico de IAM.^77 , 120^

##### 4.3.1.2. Como Interpretar Exame de Troponina Alterado?

Troponina é um marcador de injúria miocárdica e, para definir a etiologia desse fenômeno (por exemplo, diagnóstico de IAM), devemos integrar informações clínicas e de outros exames. Por mais que a acurácia dos *kits* "ultrassensíveis" tenha melhorado nos últimos anos, várias condições clínicas, além do IAM do tipo 1, podem causar injúria miocárdica e elevar a troponina.

##### 4.3.1.3. Como Interpretar Exames de Troponina Seriados?

Os valores obtidos nos ensaios de TCas devem ser analisados de acordo com o tempo de início dos sintomas e devem ser seriados em intervalos de tempo pré-determinados, para adequada interpretação de uma eventual variabilidade entre as coletas.

A injúria miocárdica aguda é definida como uma ascensão e/ou queda dos valores de troponina de alta sensibilidade > 20% entre as medidas, desde que pelo menos um desses valores esteja acima do percentil 99 do *kit* utilizado.^77^ Há valores diferentes, de acordo com o sexo e a idade.^77 , 120 , 121^

A injúria miocárdica crônica é definida por uma variação nos valores de troponina ≤ 20% com pelo menos um desses valores acima do percentil 99 do *kit* utilizado. A análise dos valores absolutos é mais útil em pacientes com níveis menores de troponina, e, por isso, algoritmos de descarte precoce utilizam variações (delta) de valores absolutos.^121^

Além de ser necessária a avaliação da probabilidade pré-teste na interpretação dos resultados de troponina, é importante lembrar que, por mais que as troponinas de alta sensibilidade possuam uma acurácia elevada para injúria miocárdica, elas não definem a causa da injúria (ex: se é infarto ou outra causa) e, ainda que raros, podemos ter resultados falso positivos e/ou falso negativos.^122^

##### 4.3.1.4. Critérios Diagnósticos de Infarto Agudo do Miocárdio

Quando a causa da injúria miocárdica aguda for a insuficiência coronariana, estabelecemos o diagnóstico de IAM. Para isso, a injúria miocárdica aguda – ascensão e/ou queda de troponina com pelo menos um valor acima do percentil ^114^ – deve estar associada a outros elementos clínico/laboratoriais de isquemia para confirmar o infarto do miocárdio.^77^ Esses são:

1. Sintomas de isquemia miocárdica aguda;
2. Nova alteração isquêmica do ECG (incluindo desenvolvimento de nova onda Q);
3. Exame de imagem demonstrando nova perda de miocárdio viável ou nova alteração da contratilidade segmentar e que tenha padrão isquêmico;
4. Evidência angiográfica ou autópsia (esta última isoladamente pode ser suficiente para o diagnóstico de IAM)*.

*Trombo em angiografia ou autopsia é critério para IAM tipo 1, mas não faz parte dos critérios de IAM tipo 2 e tipo 3.

Os casos de IAM tipo 4b (trombose de *stent* ) e 4c (reestenose de *sten* t) apresentam os mesmos critérios de IAM tipo 1, entretanto, na angiografia há identificação de trombose ou reestenose de *stent* (enquanto no IAM tipo 1 o mecanismo seria, habitualmente, aterotrombose por erosão ou rotura de placa aterosclerótica). Casos de IAM tipo 3 representam situações em que não há tempo hábil para coletar exames e definir o diagnóstico de IAM, pois o paciente evolui a óbito rapidamente. Já os casos de IAM tipo 4a e tipo 5 são periprocedimentos e, portanto, os tipos 3, 4a e 5 fogem do escopo desta diretriz.

##### 4.3.1.5. Qual o Tempo Hábil para Descartar o Diagnóstico de Infarto Agudo de Miocárdio?

Há variação, de acordo com o *kit* de troponina.

Nos casos de troponina de alta sensibilidade, em um paciente com início dos sintomas há mais de 3 horas (e sem recorrência), um nível abaixo dos valores classificados como muito baixos seria suficiente para descartar IAM (embora não descarte outros diagnósticos, como AI). Nas demais situações, há necessidade de mais de uma dosagem de troponina além da inicial, embora valores muito elevados (por exemplo, acima de 5x o limite da normalidade) indiquem alta probabilidade de IAM em um contexto de suspeita clínica, mesmo em uma dosagem isolada. A interpretação das dosagens seriadas deve se basear idealmente em algoritmos ( *pathways* ) validados.^104^

Nas troponinas que não preenchem critérios de alta sensibilidade, deve-se esperar de 6 a 12 horas (depende do ponto de corte do kit da troponina convencional). Um valor negativo após este intervalo do último episódio de sintomas pode afastar IAM, embora não exclua outros diagnósticos de risco.

##### 4.3.1.6. Qual o Algoritmo de Escolha?

Diversos algoritmos com troponina de alta sensibilidade foram propostos com o objetivo de reduzir o tempo para definir admissão hospitalar ( *rule-in* ) e para descarte de IAM ( *rule-out* ). É recomendada a realização da primeira dosagem de troponina na admissão do paciente. Nos casos sem tempo hábil e níveis suficientes para descartar, deve-se repetir a dosagem do biomarcador.

O algoritmo de 0-3 horas, em que dois valores abaixo do percentil 99 descartam IAM, foi avaliado em diversos estudos, incluindo uma metanálise, tendo se mostrado inferior aos algoritmos mais precoces, como 0-1 hora e 0-2 horas.^123^ O uso de um algoritmo como de 0-1 ou 0-2 horas deve ser preferencial, respeitando os valores previamente determinados de corte e variações absolutas do *kit* utilizado ( Tabelas 24 , 25 e 26 e Figuras 11A e 11B )

**Tabela 24:** Algoritmos de decisão com troponina de alta sensibilidade

Abordagem	Critério para descarte (rule out)	Pontos positivos	Pontos negativos
**0-3 horas (apenas deve ser considerado na impossibilidade do uso dos algoritmos de 0/1h ou 0/2h)**	- Se sintomas > 6 horas (assintomático no momento), única dosagem < percentil 99 OU - Se < 6 horas de sintomas, com dosagens seriadas (a cada 3 horas) < percentil 99	Usar percentil 99 como limiar para decisão (similar ao uso do valor de referência da troponina convencional, com maior familiaridade dos médicos)	Menos pacientes atingem critério de descarte e, quando atingem, acontece mais tardiamente (menor sensibilidade)
**0 hora (única dosagem)**	Dosagem abaixo do limiar de detecção ou um *cutoff* validado em sintomas > 3 horas	Definição imediata (sem necessidade de segunda dosagem)	Recomenda-se que paciente busque precocemente o atendimento médico (busca tardia não é desejável em termos de saúde pública)
**0-1 hora (algoritmo de escolha)**	Usa troponina da admissão (0 hora) e variação (delta) em 1 hora para definir grupo: descarte ( *rule out* ), observação ou admissão ( *rule in* )	Evita os problemas inerentes ao percentil 99 e consegue descartar mais casos de forma mais precoce 1 *	O momento da coleta é muito relevante para a interpretação desse algoritmo, cujos valores são de difícil memorização 2 *
**0-2 horas (alternativa quando não for possível realizar 0/1h)**	Idêntico ao 0-1 hora, só que o delta seria avaliado em 2 horas (ao invés de 1 hora)	Pontos positivos semelhantes ao do 0-1 hora 1 * e é mais factível em centros que não conseguem 0-1 hora	Pontos negativos semelhantes ao do 0-1 hora, mas têm menor validação e definição ocorre com 1 hora de "atraso" 2 *
***High* -STEACS**	- Se sintomas > 3 horas (assintomático no momento), única dosagem < 5 ou 6 ng/L 3 * OU - Se mudança (delta) entre 0 e 3h for < 3 ng/L mantendo troponina < percentil 99 (ajustada por sexo)	Também se aproveita da sensibilidade e precisão da TCas e usa percentil ajustado ao sexo	Menos pacientes são categorizados no grupo descarte ( *rule-out* ) do que nos algoritmos 0-1 hora e 0-2 horas (além de menor definição de *rule-out* , este ocorre com maior atraso)

1 Ambos (0-1 hora e 0-2 horas) tomam proveito da melhor sensibilidade e precisão da troponina cardíaca de alta sensibilidade (TCas).

2 Ambos devem ter cuidado nos casos de troponina alterada porém sem variação (delta) em 1 ou 2 horas, pois pode ser fase de platô de um IAM (quando níveis de troponina são bastante elevados, esse problema seria mitigado, pois valor isolado muito alto já indicaria rule-in, independentemente do delta).

3 < 5 ng/L na troponina I de alta sensibilidade ou 6 ng/L na troponina T de alta sensibilidade.

**Tabela 25:** Aplicação dos algoritmos dos marcadores de injúria miocárdica

	Classe de recomendação	Nível de evidência
Troponina cardíaca de alta sensibilidade é o marcador de escolha para investigação de IAM.	I	B
Na ausência de troponina cardíaca de alta sensibilidade, usar investigações com dosagem de biomarcador disponível * a cada 3 horas, até tempo hábil para definir ou afastar diagnóstico de IAM (varia de acordo com tipo de biomarcador).	I	B
Os algoritmos de troponina cardíaca de alta sensibilidade 0-1 hora e 0-2 horas são preferíveis aos algoritmos de 0-3 horas.	IIa	B
Em pacientes com dor precordial há mais de 3 horas, uma única dosagem de troponina cardíaca de alta sensibilidade abaixo do limite de detecção do ensaio pode ser suficiente para descartar IAM, quando associada a escores clínicos de baixo risco, eletrocardiograma sem alterações isquêmicas e ausência de dor recorrente ou persistente.	IIa	B
Se troponina quantitativa disponível, não se deve solicitar CK-MB para investigação de IAM.	III	B

IAM: infarto agudo do miocárdio.

* Na ausência de troponina cardíaca de alta sensibilidade, pode-se utilizar troponina convencional ou, na ausência desta, CKMB-massa.

**Tabela 26:** Valores de troponina a serem utilizados no fluxograma de 0-1/2 hora, de acordo com o kit utilizado

Ensaio (kit)	Muito baixo	Baixo	Sem Δ 1h	Elevado	Δ 1h
hs-cTnT (Elecsys; Roche)	<5	<12	<3	≥52	≥5
hs-cTnI (Architect; Abbott)	<4	<5	<2	≥64	≥6
hs-cTnI (Centaur; Siemens)	<3	<6	<3	≥120	≥12
hs-cTnI (Access; Beckman Coulter)	<4	<5	<4	≥50	≥15
hs-cTnI (Clarity; Singulex)	<1	<2	<1	≥30	≥6
hs-cTnI (Vitros; Ortho-Clinical Diagnostics)	<1	<2	<1	≥40	≥4
hs-cTnI (Pathfast; LSI Medience)	<3	<4	<3	≥90	≥20
hs-cTnI (TriageTrue; Quidel)	<4	<5	<3	≥60	≥8
hs-cTnI (Dimension EXL; Siemens)	<9	<9	<5	≥160	≥100
**Ensaio (kit)**	**Muito baixo**	**Baixo**	**Sem Δ 2h**	**Elevado**	**Δ 2h**
hs-cTnT (Elecsys; Roche)	<5	<14	<4	≥52	≥10
hs-cTnI (Architect; Abbott)	<4	<6	<2	≥64	≥15
hs-cTnI (Centaur; Siemens)	<3	<8	<7	≥120	≥20
hs-cTnI (Access; Beckman Coulter)	<4	<5	<5	≥50	≥20
hs-cTnI (Clarity; Singulex)	<1	TBD	TBD	≥30	TBD
hs-cTnI (Vitros; Ortho-Clinical Diagnostics)	<1	<2	<3	≥40	≥5
hs-cTnI (Pathfast; LSI Medience)	<3	<4	<4	≥90	≥55
hs-cTnI (TriageTrue; Quidel)	<4	TBD	TBD	≥60	TBD

Os pontos de corte se aplicam independentemente da idade, sexo e função renal. Pontos de corte otimizados para pacientes com mais de 75 anos de idade e para pacientes com disfunção renal foram avaliados, mas não demonstraram consistentemente melhor equilíbrio entre segurança e eficácia em comparação com esses pontos de corte universais. Médico deve sempre checar no seu laboratório qual o kit utilizado e quais os pontos de corte recomendados (há testes e novos ensaios em desenvolvimento e pontos de corte podem modificar). TBD: a ser determinado (do inglês, to be determined).

Na ausência de troponina de alta sensibilidade, pode-se utilizar a troponina convencional quantitativa ( Tabela 25 ). Deve-se seguir padrão, com dosagem inicial (0 hora), seguida de repetição de coletas a cada 3 horas, até que se tenha uma amostra com tempo maior do que 6 a 12 horas do início dos sintomas (tempo hábil depende do ponto de corte do kit da troponina). Em casos de dor recorrente ou alta suspeita, podem ser coletadas troponinas adicionais. O valor acima do ponto de corte será o "balizador" para definir casos como "positivos" para injúria miocárdica (e provável IAM) ou "negativos" (sem critérios para injúria miocárdica e, por consequência, sem critérios para IAM, embora possam ser infartos caso fossem utilizadas análises de alta sensibilidade).

As Figuras 11A e 11B apresentam os algoritmos recomendados para troponina cardíaca de alta sensibilidade.

#### 4.3.2. Direcionamento Conforme Classificação no Algoritmo

Os protocolos de atendimento de pacientes com dor torácica na emergência buscam estabelecer o rápido diagnóstico de condições ameaçadoras à vida, ao mesmo tempo que procuram identificar os pacientes que podem ser liberados dos serviços de emergência sem a necessidade de internação hospitalar. Essa estratégia evita que, em pacientes com probabilidade/risco muito reduzido de SCA, ocorram investigações desnecessárias em ambientes de PS, enfermarias ou unidades coronarianas.^79 , 80 , 88^

**Figura 12:**
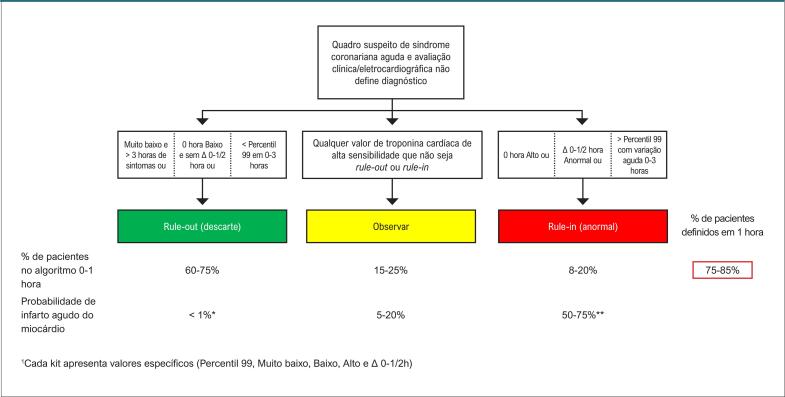
Classificação do paciente de acordo com algoritmos de troponina de alta sensibilidade (0-1h, 0-2h e 0-3h). ^1^ECG: eletrocardiograma; IAM: infarto agudo do miocárdio. *Exceto pacientes com histórico de DAC, cuja probabilidade de IAM > 1%, mesmo que algoritmo 0-1h o coloque no grupo rule-out (considerar também avaliação adicional se HEART > 3). **Se solicitação indiscriminada de troponina (pacientes de baixa suspeita clínica), valor preditivo positivo pode ficar abaixo de 50%.

Para tanto, a utilização conjunta de escores clínicos, do ECG e da dosagem da troponina, combinados com o julgamento médico, permite classificar os pacientes com dor torácica atendidos nos pronto-atendimentos em SCA descartada ( *rule-out* ), SCA confirmada ( *rule-in* ) e zona intermediária (observação)^88^ ( Figura 12 ).

##### 4.3.2.1. SCA Descartada (Rule-Out)

Pacientes sem diagnóstico definido após investigação adequada e que apresentam escores clínicos^126 , 127^ de baixo risco, como o HEART ≤ 3 ou EDACS < 16, associados a um ECG sem alterações isquêmicas e troponinas negativas em tempo hábil, são considerados de muito baixo risco/probabilidade para SCA ( *rule-out* ).

As troponinas de alta sensibilidade permitem afastar de forma mais precoce o diagnóstico de IAM e com maior acurácia em relação às troponinas convencionais. Assim como as convecionais, a coleta inicial deve ser realizada na chegada do paciente, independentemente do tempo de início de sintomas. Nos pacientes que apresentam valores iniciais de troponina de alta sensibilidade classificados como muito baixos (ou seja, valores geralmente abaixo do limite de detecção), uma única dosagem de troponina de alta sensibilidade pode ser suficiente para descartar IAM quando o ECG é normal e, especialmente, quando os escores clínicos são de baixo risco ou baixa probabilidade de SCA (HEART ≤ 3 ou EDACS < 16) – desde que o intervalo entre o início dos sintomas e a coleta da troponina de alta sensibilidade seja maior ou igual a 3 horas.^88 , 128 , 129^

Nos demais casos (sintomas < 3 horas e/ou troponina detectável na primeira dosagem), uma segunda coleta deve ser realizada com um intervalo de 1 ou 2 horas em relação à primeira (se *kit* de troponina de alta sensibilidade validado para algoritmos de 0-1 hora e/ou 0-2 horas). Valores de troponina de alta sensibilidade sem variação significativa após 1 ou 2 horas caracterizam pacientes com probabilidade muito baixa de SCA. É importante ressaltar que, assim como o percentil 99, os valores considerados para definir a variação da Tn de alta sensibilidade como significativa diferem conforme o ensaio utilizado ( Tabela 26 ).^130 – 133^

**Table:** 

O que fazer se não houver troponina de alta sensibilidade?
Nas situações em que não haja a disponibilidade de TCas, a troponina convencional poderá ser utilizada como biomarcador para diagnóstico de IAM. Devido ao maior tempo necessário para a elevação da troponina convencional, considera-se adequada a realização de duas ou mais coletas com um intervalo maior, sendo a primeira na chegada do paciente e a segunda entre 3 a 6 horas até tempo hábil (na maioria dos *kits* convencionais, é necessário mais de 6 horas).
Se houver elevação de troponina convencional num contexto clínico e/ou eletrocardiográfico compatível, faz-se o diagnóstico de IAM.
Apesar da menor sensibilidade para diagnóstico de IAM, se ao final do tempo de observação (varia geralmente entre 6 e 12 horas de acordo com kit de troponina convencional), os pacientes apresentarem troponina negativa e preencherem critérios clínicos de baixo risco (por exemplo, HEART score ≤ 3) com ECG sem alterações isquêmicas, estes poderão ser liberados do pronto-atendimento, conforme julgamento clínico da equipe médica (exclusão de diagnósticos de risco).^124 , 125^
A dosagem de CKMB só deve ser considerada na ausência de troponina e deve-se dar preferência para CKMB massa.

Quando não houver variação significativa de TCas em 1 ou 2 horas, afastados outros diagnósticos, os pacientes hemodinamicamente estáveis, sem recorrência de dor e ECG sem alterações isquêmicas podem ser liberados dos serviços de emergência para avaliação adicional em regime ambulatorial precoce, especialmente se os escores clínicos forem compatíveis com baixo risco.^134 – 137^ Esses algoritmos de 0 e 1 hora foram validados sem necessariamente ter a aplicação de escores clínicos de forma adicional. Entretanto, há evidências de que o algoritmo de troponina isoladamente não é suficiente para pacientes com história de doença coronária conhecida. ^136^ Portanto, uma conduta mais conservadora, com uso adicional de escores clínicos, pode trazer mais segurança ao liberar o paciente do PS após avaliação com algoritmo de troponina de alta sensibilidade.^136 – 142^ De qualquer maneira, com ou sem escore clínico, o julgamento do médico e sua avaliação clínica são fundamentais em todos os momentos de decisão, uma vez que TCas dentro dos valores normais afastam IAM, mas não descartam outros diagnósticos (por exemplo, AI).

##### 4.3.2.2. Admissão Confirmada (Rule-In)

Após a avaliação clínica e realização do ECG inicial, pacientes com critérios diagnósticos eletrocardiográficos são classificados como SCA confirmada e devem ser submetidos ao manejo, conforme diretrizes específicas.^88 – 89 ; 139 – 140^

Entretanto, tais pacientes representam a minoria dos casos. Na maior parte dos pacientes investigados, o ECG não é diagnóstico, e procede-se para a rota diagnóstica, que tem como um pilar a avaliação seriada de troponina para definição de um possível diagnóstico de IAM.

Nos casos em que a troponina de alta sensibilidade encontra-se acima do percentil 99 e/ou apresenta variações significativas dentro dos critérios de cada *kit* (mesmo que abaixo do percentil 99), esses são considerados habitualmente como *rule-in* ( Figura 12 ). É importante avaliar se injúria crônica ou aguda e, nos casos de injúria aguda, avaliar critérios para o diagnóstico de IAM. Se confirmado o diagnóstico, esses pacientes devem ser tratados conforme as diretrizes de SCA.^88 – 89 , 139 – 140^

Conforme ilustrado na Figura 12 , os algoritmos preferenciais de troponina de alta sensibilidade (0-1 hora e 0-2 horas) classificam os pacientes em três grupos e direcionam conduta subsequente, sempre aliada à avalição clínica ( Tabela 27 ). ^141 – 142^

**Tabela 27:** Direcionamento dos casos de acordo com algoritmo de troponina

	Classe de recomendação	Nível de evidência
Na ausência de outras hipóteses diagnósticas de risco, (ex: angina instável, dissecção de aorta), os pacientes com avaliação clínica-eletrocardiográfica "não-diagnóstica" e que apresentem classificação compatível com *rule-out* em algoritmos de troponina de alta sensibilidade de 0-1 hora ou 0-2 horas, podem ser considerados para alta para investigação complementar em regime ambulatorial, especialmente na ausência de DAC conhecida e escores clínico de baixo risco (HEART ≤ 3).	I	B
Pacientes que apresentem classificação compatível com *rule-in* em algoritmos de troponina de alta sensibilidade de 0-1 hora ou 0-2 horas devem ter ativamente checados os critérios diagnósticos de IAM espontâneo (tipo 1 ou 2) e de outras causas de injúria miocárdica, com abordagem inicialmente intra-hospitalar.	I	B
Situações que não se enquadrem nos grupos de *rule-in* ou *rule-out* (zona "cinza") e/ou quando avaliação clínica não caracterize baixo risco (por exemplo, escore HEART elevado, suspeita persistente de diagnósticos potencialmente fatais) devem ser consideradas como probabilidade intermediária e avaliadas de forma individualizada, considerando investigação adicional com exames não invasivos (grupo observação).	I	B

SCA: síndrome coronariana aguda; ECG: eletrocardiograma; DAC: doença arterial coronariana; IAM: infarto agudo do miocárdio.

#### 4.3.3. Direcionamento Inicial dos Casos em Zona Intermediária (Observação)

Na zona intermediária de algoritmos diagnósticos (ou seja, que não permitem classificar como *rule-in* ou *rule-out* ), a probabilidade de IAM é geralmente de 5 a 20%. Nessa situação, não há indicação formal de internação rotineira, entretanto, também não há segurança para liberar o paciente para casa; ferramentas adicionais para guiar o diagnóstico diferencial são necessárias, sendo recomendada a (re)checagem cuidadosa dos critérios de IAM espontâneo (tipo 1 ou 2).

Na ausência de critérios diagnósticos de IAM (situação comum nas zonas intermediárias dos algoritmos de TCas), não há indicação formal de estratificação invasiva e, portanto, a investigação deve prosseguir inicialmente com as seguintes estratégias:

- Novas dosagens de troponina (eventuais elevações adicionais após a segunda dosagem da troponina poderiam elevar probabilidade de IAM);
- Realização de ecocardiograma e busca ativa de diagnósticos diferenciais;
- Testes não invasivos para avaliar doença coronária/isquemia miocárdica podem ser considerados em pacientes de risco > baixo sem esclarecimento diagnóstico. A escolha do teste não invasivo deve se basear principalmente na disponibilidade e experiência de cada centro. Outros fatores podem ajudar nessa decisão, como o histórico ou não de (doença arterial coronariana) DAC prévia. Nos casos sem DAC conhecida, a existência de exame prévio recente pode influenciar a decisão do médico em prosseguir ou não a investigação ( Figura 13 ).^79 , 80^ Nos casos com DAC conhecida, a existência de doença obstrutiva (≥ 50%, ICP ou RM cirúrgica prévia) ou não também pode influenciar a escolha do método não invasivo ( Figura 14 ).^79 , 80^

Esses fluxogramas para escolha do teste preferencial ( Figuras 13 e 14 ) também podem ser utilizados em casos de forte suspeita clínica de dor cardíaca isquêmica em paciente estável que não preencheu critérios adicionais para SCA (ou seja, troponina e ECG sem alterações), mesmo que não esteja na categoria da zona "cinza" dos algoritmos de TCas (ex: paciente classificado como "rule-out" pelo algoritmo de TCas mas com critérios adicionais de risco/maior probabilidade de SCA).

Nos casos em que investigação afastou IAM e outros diagnósticos diferenciais de risco para o paciente, mas persiste a suspeita de SCA e o acesso a exames de imagem sejam mais limitados, a realização de teste ergométrico (na ausência de contraindicações) pode ajudar a refinar a probabilidade diagnóstica e direcionar para o melhor recurso.^143 – 161^

Se, ao final da investigação não invasiva dos casos de injúria miocárdica aguda, não for possível esclarecer o diagnóstico, a cineangiocoronariografia pode ser considerada em casos de alta suspeita clínica de IAM (especialmente se exames inconclusivos após investigação ou paciente desenvolver critérios de instabilidade durante observação). As principais recomendações para este grupo de probabilidade intermediária estão na Tabela 28 .

**Tabela 28:** Exames iniciais em casos de probabilidade intermediária

	Classe de recomendação	Nível de evidência
Solicitar pelo mais uma dosagem de troponina (terceira dosagem) e um novo traçado de eletrocardiograma (com derivações complementares).	I	C
Revisar possíveis diagnósticos diferenciais (com apoio de escores diagnósticos) e solicitar exames conforme probabilidade diagnóstica (por exemplo, D-dímero na suspeita de TEP com baixa probabilidade pré-teste).	I	C
Se troponina acima do percentil 99, checar de forma sistemática critérios de infarto agudo do miocárdio e também causas alternativas de injúria miocárdica.	I	C
Solicitar ecocardiograma para apoio no esclarecimento diagnóstico de casos indefinidos de probabilidade intermediária.	IIa	C
Realizar exames para investigação de doença arterial coronária e/ou isquemia (preferencialmente não invasivos) caso não haja esclarecimento diagnóstico após investigação inicial de pacientes com probabilidade intermediária de síndrome coronariana aguda.	IIa	C
A escolha do teste não invasivo deve se basear principalmente na disponibilidade e experiência de cada centro e no histórico ou não de doença arterial coronária conhecida.	IIa	C
Em pacientes que tenham angiotomografia de coronárias completamente normal nos últimos 2 anos ou teste de estresse sem critérios de isquemia e adequado (negativo) no último ano, pode-se considerar não repetir o teste não invasivo se sintomas estáveis.	IIb	C

TEP: tromboembolismo pulmonar.

#### 4.3.4. O que Fazer nos Casos com Injúria Miocárdica e Coronária sem Obstruções?

Todos os casos com troponina alterada ( *rule-in* e zona intermediária) que apresentaram indicação de avaliação angiográfica (incluindo os casos com critérios de IAM) devem ter uma definição diagnóstica, mesmo que angiografia não demonstre obstruções. Dessa forma, nos casos de troponina anormal em que não houver obstrução coronária na angiografia, os pacientes podem ser classificados inicialmente como TINOCA (do inglês, *troponin-positive, nonobstructive coronary arteries* ), grupo que pode ser dividido em três grandes subgrupos:

1. MINOCA (infarto do miocárdio com coronárias não obstrutivas);
2. Outras causas cardíacas (por exemplo, miocardite, Takotsubo);
3. Causas extracardíacas.

##### 4.3.4.1. Investigação de Casos de Troponina Elevada sem Obstrução Coronária (Troponin-Positive Nonobstructive Coronary Arteries - TINOCA ou TpNOCA)

Cerca de 5 a 15% dos casos diagnosticados como IAM que vão para cineagiocoronariografia não apresentam aterosclerose com obstrução ≥ 50% na avaliação angiográfica.^88^

**Figura 15:**
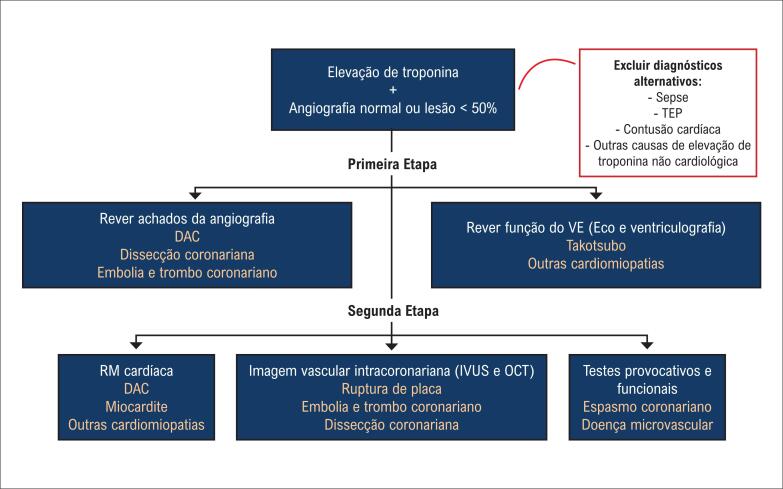
Fluxograma de diagnóstico em casos de infarto agudo do miocárdio com coronariografia normal. TEP: tromboembolismo pulmonar; DAC: doença arterial coronariana; RM: ressonância magnética; IVUS: ultrassonografia intravascular; OCT: tomografia de coerência óptica.

Muitas vezes, o paciente que vai para angiografia não tem o diagnóstico de IAM estabelecido e, por isso, esse grupo maior que apresenta injúria miocárdica e angiografia sem lesões coronarianas com obstrução ≥ 50%, independentemente da etiologia, recebe o nome de TINOCA,^88^ o qual inclui o grupo de MINOCA, quando houver confirmação diagnóstica de IAM. As recomendações para investigação de TINOCA estão apresentadas em fluxograma ( Figura 15 ) e tabela de recomendações ( Tabela 29 ).

**Tabela 29:** Investigação adicional de pacientes com injúria miocárdica e angiografia normal ou com lesões coronarianas < 50%

	Classe de recomendação	Nível de evidência
Confirmar se, de fato, trata-se de caso de TINOCA (rever imagem da angiografia em busca de obstrução, dissecção, embolia, trombo coronário; checar outros exames em busca de diagnósticos diferenciais como Takotsubo).	I	C
Solicitar ressonância magnética cardíaca e outros exames adicionais conforme suspeita clínica (ex.: imagem intracoronária e/ou testes para avaliação de espasmo ou doença microvascular).	IIa	C

### 4.4. Uso Racional de Testes Não Invasivos

A escolha racional de métodos não invasivos para avaliação de SCA é fundamental, tendo em vista que o diagnóstico diferencial de dor torácica é amplo e requer uma abordagem estruturada para adequada e rápida distinção entre causas cardíacas e não cardíacas, devido à mortalidade e morbidade associadas às DCVs. Esses testes podem ser feitos em observação hospitalar ou ambulatorial precoce (dependendo do risco do paciente e disponibilidade de recursos).

Na observação hospitalar, testes de estresse (físico ou farmacológico) só devem ser realizados em pacientes estáveis após afastar IAM com segurança (tempo suficiente varia conforme o período de início da dor torácica e o tipo de biomarcador utilizado, conforme descrito nas Seções 3.3, 4.1, 4.2 e 4.3).

Nos pacientes elegíveis com dor torácica aguda (ou equivalentes), o uso das técnicas não invasivas é útil para descartar DAC obstrutiva, para estratificar o risco de desfechos adversos e para diferenciar entre DCVs potencialmente fatais e condições de baixo risco.

Diversos fatores são importantes na decisão, como a disponibilidade, experiência local, o tipo de apresentação clínica e, principalmente, as características dos pacientes e as suas opções. Em casos desafiadores, mais de um método pode ser necessário, e a interpretação multimodalidade será fundamental nessas situações. Por estas razões, além das orientações gerais para seleção do método não invasivo ( Figuras 13 , 14 e 15 ), aspectos específicos de cada método serão discutidos em maiores detalhes nesta seção, para apoiar a melhor decisão em cada situação clínica (recomendações centradas no paciente). E, por fim, ao se mencionar métodos diagnósticos, o raciocínio sempre deve ser integrado ao teorema de Bayes, segundo o qual a probabilidade pós-teste de um evento ou doença está condicionada à sua probabilidade pré-teste.

#### 4.4.1. O Papel do Teste de Esforço

O teste de esforço (TE) é um método funcional amplamente utilizado para diagnóstico e avaliação de risco de eventos em diversos cenários clínicos, tendo acumulado um robusto conjunto de evidências ao longo de várias décadas. Apresenta as vantagens de ser bastante acessível, menos oneroso e não necessitar de exposição à radiação ionizante ou contraste.

No atendimento à dor torácica aguda na unidade de emergência, recorre-se ao TE desde o início dos anos de 1990, tanto como método auxiliar na exclusão de SCA quanto na estratificação do risco de morte no curto e médio prazos, o que aumenta a segurança da alta hospitalar.^144^ Grande parte da evidência dessa aplicação se baseia em estudos observacionais.^145 , 146^ Os raros ensaios clínicos randomizados encontrados são, em geral, de centro único e com tamanho amostral reduzido, não apresentando poder estatístico adequado para discriminar risco de morte ou infarto não fatal entre os grupos estudados.^147 – 149^

O processo de estratificação de risco em grupos de baixo, intermediário ou alto é por vezes subjetivo, não se valendo da aplicação dos escores prognósticos conhecidos, o que pode limitar a ampla aplicabilidade dos resultados.^149 , 150^

A despeito das várias questões metodológicas, os trabalhos tinham como critérios de inclusão apenas pacientes com dor torácica aguda com suspeita clínica de origem isquêmica. Diferentes etiologias de dor torácica aguda, bem como outras condições em que o TE não está indicado, são melhor detalhadas na Tabela 30 .

**Tabela 30:** Condições clínicas em que o teste de esforço não está indicado na unidade de emergência

TE contraindicado nas seguintes situações:
Dor torácica claramente de origem traumática
Dissecção aguda da aorta
Embolia pulmonar aguda
Pericardites e miocardites
Dor torácica associada a estado febril agudo
Estenose aórtica grave sintomática
Evidências de insuficiência cardíaca descompensada
Sinais de má perfusão periférica
Arritmias e HAS não controladas

HAS: hipertensão arterial sistêmica; PA: pressão arterial; TE: teste de esforço.

Os eletrocardiogramas e os biomarcadores de injúria miocárdica seriados devem ser normais ou não diagnósticos de SCA. As alterações do ECG basal que afetam a acurácia diagnóstica do TE para isquemia miocárdica estão arroladas na Tabela 31 .

**Tabela 31:** Alterações no eletrocardiograma de base que limitam a acurácia diagnóstica do teste de esforço para isquemia miocárdica

Limitam a acurácia do TE:
Bloqueio de ramo esquerdo
Sobrecarga ventricular esquerda
Sobrecarga ventricular direita
Estimulação cardíaca artificial
Pré-excitação ventricular
Fibrilação e flutter atriais
Infradesnível de ST de base ≥ 1 mm

TE: teste de esforço.

Poucos trabalhos incluíram uma proporção expressiva de indivíduos acima de 65 anos,^145 , 150 , 151^ com a média de idades variando, em geral, entre 50 e 60 anos. Indivíduos do sexo masculino predominam na maior parte dos grupos estudados, com proporções variando de 40% a 75%.

As maiores vantagens do TE na unidade de emergência estão na sua grande segurança e no seu poder de excluir a presença de obstrução coronária crítica. Entretanto, sua capacidade discriminatória total é reportada como baixa, considerando-se todos os seus resultados – positivos, negativos ou inconclusivos – para isquemia miocárdica.^150 – 158^

Existem raríssimos estudos abordando subgrupos específicos. Em um trabalho que incluiu apenas mulheres de baixo risco com ECG e troponinas normais, a realização do TE ou métodos de imagem não melhorou a predição de infarto ou revascularização miocárdica em 30 dias.^159^ Indivíduos com doença coronária conhecida e/ou revascularização prévia não foram analisados em estudos específicos. É importante ressaltar, no entanto, que esse grupo limita sobremaneira a indicação do TE convencional, pois apresenta múltiplas comorbidades, alterações no ECG de base e uso de medicamentos antianginosos.^160^ Não há estudos convincentes que suportem a indicação de TE na dor torácica aguda associada ao uso de drogas estimulantes, como cocaína e anfetaminas.

Uma recente revisão sistemática com metanálise^161^ teve como objetivo analisar a ocorrência de eventos cardiovasculares maiores em até 1 ano após um exame cardíaco negativo, dentre esses o TE. Os autores concluíram que pacientes com dor torácica de risco baixo a intermediário não necessitam de um novo teste na unidade de emergência, dado o baixíssimo risco de eventos durante o período. Tendo em vista as limitações diagnósticas do teste ergométrico, esse método tem sido utilizado mais no sentido de refinar a avaliação prognóstica e de probabilidade diagnóstica do que como método para definir ou excluir a presença de DAC.^143^ De qualquer maneira, essa informação pode ser muito útil, especialmente em locais com recursos limitados ( Tabela 32 ).

**Tabela 32:** Indicações de teste ergométrico na dor torácica aguda

Indicações de teste ergométrico na dor torácica aguda		
O teste ergométrico pode ser recomendado para indivíduos com dor torácica aguda, em que haja suspeita clínica de isquemia miocárdica com probabilidade intermediária de DAC (ou seja, que não tenham sido classificados como baixa ou alta probabilidade de DAC), com ECG e biomarcadores de injúria miocárdica normais e que sejam elegíveis para a realização do esforço físico (idealmente associado a métodos de imagem).	IIa	B
O tempo para a realização do exame deve ser o tempo suficiente para afastar IAM com segurança (varia conforme o período de início da dor torácica e o tipo de biomarcador utilizado).	IIa	B
Em pacientes com dor torácica aguda de suposta origem isquêmica e probabilidade intermediária, admitidos na unidade de emergência com um TE máximo negativo para isquemia realizado nos últimos 12 meses, priorizar outro exame conforme suspeita clínica (não repetir TE * ).	IIa	B

DAC: doença arterial coronariana; ECG: eletrocardiograma; IAM: infarto agudo do miocárdio; TE: teste de esforço.

* Desde que não haja mudança relevante em relação ao quadro clínico pregresso.

#### 4.4.2. Papel da Ecocardiografia na Avaliação da Dor Torácica

O papel do ecocardiograma em repouso na avaliação de pacientes na sala de emergência com dor torácica se alicerça em vários estudos publicados.^162 – 164^ Estudos iniciais demonstraram valor da observação das alterações transitórias da motilidade segmentar do VE como marcadores precisos de isquemia miocárdica e, em estudos mais recentes, utilizando a ecocardiografia com agentes de realce miocárdico, demonstrou-se que a perfusão miocárdica se mostrou útil para determinar o diagnóstico e prognostico da dor torácica de origem cardíaca.^162 – 164^

Kalvaitis et al.^162^ avaliaram 957 pacientes que se apresentaram à sala de emergência com quadro clínico de dor precordial com duração menor de 12h e ECG inconclusivo e troponina normal ou discretamente alterada. Tiveram como base fisiopatológica a observação de que a maior parte da motilidade regional observada em repouso deriva do espessamento da porção mais endocárdica do miocárdio e que os eventos isquêmicos que afetam o miocárdio sempre começam na camada subendocárdica caminhando para a região subepicárdica, fenômeno conhecido por frente de onda da necrose miocárdica. Dessa forma, áreas isquêmicas tão pequenas quanto as que afetam 20 a 30% da espessura miocárdica já são capazes de causar significativa alteração na motilidade regional,^163^ que persiste por várias horas após a injuria isquêmica original, tendo sido demonstrada a presença de alterações na motilidade em 97% dos pacientes com infarto. Nesse mesmo estudo, nos 500 pacientes sem alterações na motilidade, em somente 2 casos foram confirmados pequenos infartos que não tinham sido detectados. Dessa forma, ao final de 12 horas do início da primeira dor precordial, o valor preditivo negativo para a presença de infarto nessa população foi de 94%.^162^

Por outro lado, nem sempre as alterações na motilidade significam presença de infarto, podendo significar, por exemplo, os efeitos da isquemia transitória sobre o miocárdio. As alterações prolongadas da motilidade ventricular apresentam mais clara consequência prognóstica. Estudo de Rinkevich et al.^164^ avaliou 1.017 pacientes com dor torácica, ECG não diagnóstico e troponina normal ou discretamente alterada. Destes, 292 (28,7%) tiveram eventos em um acompanhamento médio de 7,7 meses. Infarto ocorreu em 13,1%, AI em 6,7%, morte em 1,9% e insuficiência cardíaca (IC) em 3,7%. Ainda, 2% precisaram de intervenção percutânea, e 1,3%, de revascularização cirúrgica do miocárdio. Já de 43 pacientes que sofreram eventos e não possuíam alteração na motilidade regional, somente 10 tiveram pequenos infartos. Quando a motilidade e perfusão eram normais, as taxas de eventos cardíacos de 1 a 2 anos foram de 9,8% e 12,6%. A maioria dos eventos era de natureza "suave". Essas taxas aumentaram, respectivamente, para 18,9% e 21% quando motilidade era normal, mas perfusão era anormal, e para 40,1% e 48,8% quando motilidade era anormal, mas perfusão era normal. Finalmente, essas taxas aumentaram ainda mais, para 64,7% (1 ano) e 74,4% (2 anos) quando ambas motilidade e perfusão eram anormais.

Quando se busca também o diagnóstico de AI (além de infarto) em pacientes com dor torácica e ECG inconclusivo, a sensibilidade do ecocardiograma passa a variar de 40 a 90%, sendo que o valor preditivo negativo fica entre 50 e 99%. Nesses pacientes, um ecocardiograma normal não parece agregar informações diagnósticas significativas, além daquelas já fornecidas pela história e ECG.

Estudos sobre a utilização do ecocardiograma de estresse com dobutamina na avaliação de uma população heterogênea com dor torácica são extensos. Sua sensibilidade para o diagnóstico de doença coronariana ou isquemia miocárdica detectada por outros métodos é de 90%, com especificidade variando de 80 a 90% e valor preditivo negativo de 98% (este último varia conforme probabilidade pré-teste). É um método muito útil para definição do prognóstico e oferece segurança para liberar o paciente que necessita estratificação não invasiva.

Além da análise miocárdica, o ecocardiograma é muito útil na análise do pericárdio. Importante relembrar que este é constituído pelas camadas visceral (composta por células mesoteliais aderidas ao epicárdio) e parietal (estrutura fibrosa de colágeno e elastina, com espessura menor que 2 mm), separadas por um espaço que, normalmente, contém de 15 a 35 mL de líquido seroso. Nesse cenário, o ecocardiograma transtorácico é de especial importância, primeiramente por excluir a presença de alterações na motilidade segmentar e, por fim, por permitir a observação de derrame pericárdico, seu espessamento e a presença de sinais inflamatórios, como traves ou grumos no interior do saco pericárdico.

Na suspeita de SAA, o ecocardiograma transtorácico pode identificar complicações (por exemplo, regurgitação aórtica, tamponamento), mas sua acurácia diagnóstica para SAA é limitada (sensibilidade: 78%–100% para o tipo A, 31%-55% para o tipo B). Já a ecocardiografia transesofágica tem acurácia > 90% e é conveniente para uso à beira do leito.

No TEP, o ecocardiograma permite identificação de trombos móveis em câmaras direitas, no tronco e/ou nos ramos principais da artéria pulmonar (especialmente pela via transesofágica), entretanto, estes achados estão presentes numa minoria dos casos. A avaliação ecocardiográfica funcional e das dimensões do VD é limitada, o que gera variação na literatura sobre a *performance* do ecocardiograma. De qualquer forma, as anormalidades só são identificadas em casos mais graves (com repercussão em câmaras direitas) ao mesmo tempo que sobrecarga ventricular direita pode ser identificada também na ausência de embolia pulmonar (doença cardíaca ou pulmonar concomitante). Tendo em vista esses aspectos, o ecocardiograma tem grande valor na estratificação de risco após o diagnóstico de TEP. Do ponto de vista diagnóstico, o ecocardiograma tem valor distinto no paciente estável e instável com suspeita de TEP:

1. Pacientes com estabilidade hemodinâmica: é um método que ajuda no diagnóstico diferencial de dispneia e dor torácica e pode eventualmente flagrar comprometimento de câmaras direitas em paciente aparentemente estável ao exame clínico. Os sinais ‘60/60’ (combinação de um tempo de aceleração da artéria pulmonar de < 60 ms com um pico de gradiente da valva tricúspide sistólica de < 60 mmHg) e de McConnell (hipocinesia da parede livre em comparação com o "ápice ecocardiográfico" do VD) têm valor diagnóstico, embora sejam pouco frequentes em casos de TEP não selecionados (10 a 20% dos casos);
2. Pacientes com instabilidade hemodinâmica: o ecocardiograma tem valor diagnóstico mais elevado nestes casos, uma vez que a ausência de sinais de sobrecarga ou disfunção do VD praticamente exclui TEP como causa da instabilidade hemodinâmica – especialmente se associada a um Doppler de membros inferiores sem sinais de trombose venosa profunda. Além de ser muito útil para afastar TEP em paciente instável, os casos hemodinamicamente comprometidos com suspeita de TEP e que apresentam sinais inequívocos de sobrecarga pressórica do VD, especialmente com achados ecocardiográficos mais específicos (sinal 60/60, sinal de McConnell ou trombo visível), podem justificar o tratamento de reperfusão de emergência quando a angiotomografia imediata não é viável em um paciente com alta probabilidade e nenhuma outra causa óbvia para a sobrecarga de pressão do VD. Nesse sentido, o próprio ecocardiograma pode ser muito útil para afastar outras causas de choque, como tamponamento, disfunção valvar aguda, disfunção do VE, dissecção aórtica e hipovolemia.

Dessa forma, há diversas recomendações para uso do ecocardiograma na investigação da dor torácica na emergência ( Tabela 33 ), sendo útil para avaliação diagnóstica das DCVs potencialmente fatais, como SCA, pericardite/tamponamento, dissecção de aorta e embolia pulmonar.

**Tabela 33:** Indicações de ecocardiograma na dor torácica aguda

	Classe de recomendação	Nível de evidência
Nos pacientes com dor torácica na sala de emergência, nos quais o eletrocardiograma e a troponina sejam inconclusivos e persiste a suspeita, um ecocardiograma transtorácico em repouso, com ou sem agentes de realce miocárdico, deve ser recomendado em até 12 horas do início da dor, a fim avaliar a alterações isquêmicas.	I	B
Nos pacientes com dor torácica na sala de emergência, nos quais já se exclui a possibilidade de necrose e de isquemia miocárdica de repouso, por ausência de sinais ecocardiográficos ou eletrocardiográficos de isquemia, um exame sob estresse indutor de isquemia miocárdica, farmacológico ou sob esforço pode ser utilizado antes da alta, quando não houver segurança para investigação ambulatorial.	I	B
Uso do ecocardiograma transtorácico para o diagnóstico de pericardite aguda naqueles pacientes com dor torácica na sala de emergência, associada a outros achados, como moléstia febril aguda, com ou sem queda do estado geral, e característica fásica respiratória dessa dor, com ou sem alterações características do segmento ST/T ao eletrocardiograma de repouso.	I	B

#### 4.4.3. Cintilografia de Perfusão Miocárdica (SPECT) e Tomografia por Emissão de Pósitrons (PET-CT)

Entre os exames funcionais que podem ser aplicados a pacientes com dor torácica aguda destacam-se os exames de medicina nuclear utilizados para avaliação da perfusão miocárdica: cintilografia de perfusão miocárdica com 99mTc-sestamibi e a tomografia por emissão de pósitrons com 82Rubídio ou 13N-Amônia, ainda limitada em nosso meio. Esses exames de perfusão miocárdica de repouso isoladamente ou de estresse associado ao repouso, têm sido utilizados com segurança e eficácia na avaliação de pacientes com dor torácica na sala de emergência, tendo sido testados em estudos randomizados multicêntricos.^79 , 80 , 165^ As características principais dos estudos de medicina nuclear nesse contexto são de um método com elevada sensibilidade para detecção de isquemia miocárdica e excelente capacidade prognóstica.

##### 4.4.3.1. Indicações para o uso de Testes de Medicina Nuclear

Após a avaliação inicial padrão de pacientes com dor torácica na sala de emergência (anamnese, exame clínico, ECG e marcadores de injúria miocárdica), um percentual desses indivíduos ainda apresenta risco considerável de doença coronariana. Esses pacientes requerem avaliação por métodos de imagem de forma a fim de identificar aqueles com síndrome coronariana aguda e aqueles com maior risco de eventos adversos. Da mesma forma que a avaliação anatômica com a angiotomografia das artérias coronárias (CTA), a utilização de métodos funcionais é especialmente útil na identificação desse grupo de pacientes.^79 , 80^

Uma das indicações mais consistentes para o uso da cintilografia de perfusão miocárdica em pacientes com dor torácica aguda é em pacientes sem diagnóstico de DAC prévia e que têm um risco intermediário de DAC. Nesse caso, o uso da cintilografia de perfusão miocárdica de estresse e de repouso é consolidado como uma técnica útil e eficaz.^79 , 80^ Em estudo unicêntrico, o uso da cintilografia de perfusão miocárdica em pacientes com dor torácica aguda teve a mesma segurança e efetividade que o uso da CTA, não apresentando diferenças no acompanhamento de desfechos adversos em 40 meses de acompanhamento.^166^ Outros estudos comparativos entre CTA e cintilografia de perfusão miocárdica também mostraram que as técnicas são comparáveis e que a técnica de avaliação da anatomia coronariana tem um potencial de aumento de angiografias invasivas e revascularizações. Da mesma forma, na investigação ambulatorial, há também evidências que colocam a medicina nuclear como boa opção.^167 , 168^ No estudo CE MARC 2 ( *Clinical Evaluation of Magnetic Resonance imaging in Coronary heart disease* 2), o teste funcional com cintilografia miocárdica ou RM levou a taxas mais baixas de angiografias desnecessárias, sem aumento significativo de eventos coronários adversos maiores.^167^ No estudo PROMISE ( *PROspective Multicenter Imaging Study for Evaluation of Chest Pain* ), que incluiu 10 mil pacientes sintomáticos com suspeita de DAC, uma estratégia de CTA inicial, em comparação com o teste funcional, não melhorou os desfechos clínicos ao longo de um acompanhamento médio de 2 anos.^168^

Uma técnica avaliada em estudo randomizado e que, quando disponível, é bastante útil, é a imagem de perfusão com sestamibi em repouso durante episódio de dor torácica aguda. As imagens normais auxiliam na tomada de decisão de triagem de emergência para pacientes com sintomas sugestivos de isquemia cardíaca aguda sem anormalidades no ECG inicial, e reduzem as internações desnecessárias em pacientes sem isquemia aguda, sem reduzir a admissão apropriada para pacientes com isquemia aguda.^169^ Usando essa técnica, conseguimos descartar IAM em 98% dos pacientes com dor torácica na sala de emergência.^170^ Embora avalie isquemia e não necessariamente necrose (infarto), a indicação deste exame tende a reduzir com o uso mais amplo de algoritmos de descarte precoce com troponina cardíaca de alta sensibilidade.

Outra indicação clínica robusta para uso da cintilografia de perfusão miocárdica de estresse em pacientes com dor torácica de risco intermediário na sala de emergência é nos casos em que foi realizada uma estratificação anatômica inicial com CTA ou angiografia invasiva e cujo resultado seja inconclusivo Nesses casos, o uso da cintilografia de perfusão miocárdica de estresse ou da PET-CT de perfusão com estresse é capaz de avaliar a funcionalidade da lesão coronariana e determinar se ela é causadora de isquemia miocárdica e dos sintomas.^79 , 80^

Outra importante indicação para uso da cintilografia de perfusão miocárdica de estresse e repouso (SPECT, do inglês *single photon emission computed tomography* ) é para avaliação de isquemia em pacientes com DAC conhecida ou daqueles em que já se saiba que há uma elevada carga de cálcio nas artérias coronárias, seja por uma tomografia de tórax prévia ou um escore de cálcio elevado.

O PET-CT, apesar de ainda incipiente no cenário nacional, tem como grande vantagem a capacidade de mensurar a reserva de fluxo coronariano, aumentando a sensibilidade da técnica em relação ao SPECT, incluindo também a possibilidade de avaliação da doença coronariana microvascular.^79 , 80^ Estudos recentes têm sugerido que a avaliação da doença microvascular com câmaras de CZT SPECT têm valor prognóstico para pacientes com doença isquêmica em coronárias não obstrutivas (INOCA), o que pode permitir a estratificação para prevenção e intervenção precoce.^171 – 174^ Essa técnica pode ser uma alternativa em cenários em que não há acesso ao PET-CT, permitindo a avaliação da reserva de fluxo miocárdica e estratificação de pacientes com dor torácica.^171 , 172^

Em resumo, as técnicas de medicina nuclear são parte importante na investigação de pacientes com dor torácica aguda na emergência para avaliação de isquemia miocárdica com ou sem doença obstrutiva epicárdica ( Tabela 34 ).

**Tabela 34:** Recomendações para uso das técnicas de medicina nuclear em cardiologia em pacientes com dor torácica aguda na emergência

	Classe de recomendação	Nível de evidência
Para pacientes de risco intermediário com dor torácica aguda e sem DAC conhecida, o uso do PET/SPECT de estresse é útil para o diagnóstico de isquemia miocárdica.	I	B
Para pacientes com risco intermediário com dor torácica aguda presente ou até em 2 horas do desaparecimento da dor e com marcadores de injúria miocárdica iniciais negativos, é útil a realização de administração do radiotraçador durante a dor torácica para afastar isquemia miocárdica.	I	B
Para pacientes de risco intermediário com dor torácica aguda e DAC conhecida, que apresentam novos sintomas ou piora dos sintomas, PET/SPECT de estresse pode ser considerada para avaliação de isquemia miocárdica.	IIa	B
Para pacientes de risco intermediário, com dor torácica aguda e sem DAC conhecida, com angiografia (invasiva ou não invasiva) inconclusiva, a realização de PET/SPECT de estresse pode ser útil para o diagnóstico de isquemia miocárdica.	IIa	C

DAC: doença arterial coronariana.

Os achados de alto risco na cintilografia miocárdica de perfusão em pacientes com dor torácica aguda geralmente indicam uma alta probabilidade de DAC significativa ou eventos cardiovasculares adversos iminentes. Entre os achados associados a um pior prognóstico, destacamos:

1. Isquemia miocárdica extensa: uma extensão dos defeitos de perfusão que ultrapasse 10% do miocárdio ou que envolva múltiplos territórios coronarianos;
2. Diminuição da fração de ejeção ventricular esquerda (FEVE) após estresse em pelo menos 10% em relação ao repouso;
3. Dilatação ventricular esquerda transitória.

Em geral, a presença de quaisquer desses achados sugere a presença de lesões anatômicas de alto risco e um potencial benefício de revascularização miocárdica, sendo indicada a avaliação angiográfica da anatomia coronariana.^79 , 80^

Um ponto novo e de rápido desenvolvimento é a adição da avaliação da reserva de fluxo coronariano aos exames funcionais de cardiologia nuclear. A reserva de fluxo coronariano (RFC), medida por meio do PET-CT de perfusão, desempenha um papel importante na estratificação de risco de pacientes com dor torácica na sala de emergência. A RFC é uma medida da capacidade das artérias coronárias de dilatar e aumentar o fluxo sanguíneo em resposta a uma demanda aumentada, como durante estresse ou exercício. Ela fornece informações valiosas sobre o estado funcional da circulação coronariana e pode auxiliar na avaliação da gravidade e prognóstico da DAC e na avaliação da disfunção microvascular. Outro ponto importante é que a RFC reduzida está associada a um maior risco de eventos cardiovasculares futuros, incluindo infarto do miocárdio, IC e morte cardíaca. Ela serve como um preditor independente de desfechos adversos, mesmo em pacientes com artérias coronárias normais ou DAC não obstrutiva. A RFC pode ajudar a identificar indivíduos com maior risco, que podem se beneficiar de estratégias de tratamento mais agressivas.^173 – 175^ Há demonstração de que a disponibilidade de PET-MPI (imagem de perfusão miocárdica) para realização de exames de pacientes da emergência foi associada a um aumento do número de encaminhamentos para a avaliação de pacientes com dor torácica aguda, bem como a um tempo de internação mais curto. Além disso, a utilização de PET-CT foi associada a uma redução de 40% no uso de testes adicionais, em comparação com o uso do SPECT em pacientes avaliados com dor torácica aguda no PS.^176^

Na Figura S8, ilustramos o caso de um paciente com dor torácica aguda admitido com ECG e marcadores de injúria miocárdica normais. A cintilografia de perfusão miocárdica de estresse físico demonstrou defeitos reversíveis de perfusão miocárdica em território da artéria coronária direita, correspondendo a 20% do miocárdio. A coronariografia demonstrou obstrução de 90% na artéria coronária direita, que foi tratada com *stent* farmacológico.

##### 4.4.3.2. Considerações Especiais em Mulheres com Dor Torácica Aguda

Um ponto adicional deve ser destacado para investigação de DAC através das técnicas funcionais: as mulheres são mais propensas a ter DAC não obstrutiva em comparação aos homens. Isso deve ser levado em consideração para que a utilização de métodos anatômicos de avaliação das coronárias não acarrete diagnósticos inadequados. A DAC não obstrutiva está associada a maior risco de infarto do miocárdio e mortalidade em comparação a ausência de DAC.^177^ Para a avaliação de pacientes com dor torácica persistente e DAC não obstrutiva, o PET MPI com avaliação da reserva de fluxo coronariano pode ser utilizado para diagnosticar disfunção microvascular e melhorar a estratificação de risco. Estudos com as câmaras de CZT SPECT sugerem que esta técnica pode ser uma possível alternativa para situações em que o PET-CT não esteja acessível.

Em relação a diagnósticos não cardíacos no protocolo de dor torácica, a cintilografia pulmonar de ventilação e perfusão é um método não invasivo, classicamente utilizado para a estratificação da probabilidade de embolia pulmonar, ao analisar o número de segmentos pulmonares acometidos, permitindo classificar a probabilidade de doença em ausente (normal), baixa, média ou alta. Deve ser interpretada com base na probabilidade pré-teste (escores Wells, Genebra), sendo uma alternativa muito útil, especialmente nos casos com limitações para realização ou análise da angiotomografia computadorizada para investigação de TEP.

#### 4.4.4. Tomografia Computadorizada Cardiovascular

O uso da tomografia computadorizada cardiovascular (CTA) para análise da luz das artérias coronárias de maneira não invasiva já se encontra estabelecido na literatura. Como bem demonstrado por diversos estudos, há excelente acurácia da angiografia por CTA das artérias coronárias, quando comparada à angiografia convencional, para o diagnóstico de estenose em pacientes de baixo a moderado risco cardiovascular, com destaque para seu alto valor preditivo negativo ( Tabela 35 ).^178 – 184^

**Tabela 35:** Sumário dos trials multicêntricos sobre acurácia da CTA em detectar estenose coronária (50% de estreitamento luminal) em pacientes de baixo a intermediário risco sem diagnóstico prévio de doença arterial coronária

ESTUDO	Sensibilidade %	VPN %	Especificidade%	VPP%
CATSCAN (Garcia et al., 2006). 7 Países, 11 centros^182^	94 (89-100)	98 (94-100)	51 (43-59)	28 (19-36)
NIMISCAD (Marano et al., 2009). 20 centros na Itália^183^	94 (89-97)	91 (85-95)	88 (81-93)	91 (86-95)
ACCURACY (Budoff et al., 2008). 16 centros nos EUA^179^	95 (85-99)	99 (96-100)	83 (76-88)	64(53-75)
CORE64 (Miller et al., 2008). 7 Países, 9 centros^184^	85 (79-90)	83 (75-89)	90 (83-94)	91 (86-95)
Meijboom et al. (2008). 3 centros na Holanda^180^	99 (98-100)	97 (94-100)	4 (55-73)	86 (82-90)

O uso da CTA na avaliação da dor torácica aguda foi avaliado de forma segura em diversos estudos, na estratificação, redução de custo e diminuição do tempo de permanência intra-hospitalar. Estudos prospectivos, controlados e randomizados avaliaram seu uso no contexto da dor torácica no PS em pacientes de baixo a intermediário risco, associado ao uso da troponina convencional negativa.^185^ Em destaque, temos três estudos.

O primeiro estudo é o multicêntrico CT-STAT ( *Coronary Computed Tomographic Angiography for Systematic Triage of Acute Chest Pain Patients to Treatment* ), que randomizou 699 pacientes com dor torácica de baixo risco para estratégias de estratificação utilizando a CTA ou a cintilografia miocárdica de repouso e estresse.^186^ A estratégia com a CTA reduziu em 54% o tempo para o diagnóstico e em 38% os custos da internação, sem que houvesse diferença na taxa de eventos adversos com relação à estratégia com a cintilografia.

O segundo estudo foi o multicêntrico ACRIN-PA ( *Angiography for Safe Discharge of Patients with Possible Acute Coronary Syndromes* ), que teve como objetivo primário avaliar a segurança da utilização da CTA na avaliação de pacientes com dor torácica de risco baixo a intermediário (TIMI RISK 0 a 2), em comparação com a abordagem tradicional.^187^ Nenhum dos pacientes com CTA normal apresentou o desfecho primário (morte cardíaca ou infarto nos primeiros 30 dias após a admissão). Além disso, os pacientes do grupo CTA tiveram maior taxa de alta das unidades de emergência (49,6% *versus* 22,7%) e menor tempo de internação (18 horas *versus* 24,8 horas, p < 0,0001), sem diferença no número de revascularizações ou cateterismos.

O terceiro estudo foi o ROMICAT-II ( *Rule Out Myocardial I schemia/Infarction by Computer Assisted Tomography* ), que avaliou, em grupos semelhantes de pacientes, o tempo de permanência na emergência e os custos hospitalares.^188^ Esse estudo incluiu 1.000 pacientes, com idade média de 54 anos, sendo o tempo de permanência hospitalar significativamente menor nos pacientes estratificados para CTA, quando comparados ao grupo submetido à avaliação tradicional (23,2 ± 37,0 horas *versus* 30,8 ± 28,0 horas, p = 0,0002). O tempo até a exclusão do diagnóstico de SCA também foi menor no grupo submetido à CTA (17,2 ± 24,6 horas *versus* 27,2 ± 19,5 horas, p < 0,0001). Em relação às metas de segurança, não houve qualquer diferença entre os grupos. No grupo estratificado pela CTA, houve aumento significativo dos pacientes que receberam alta hospitalar diretamente da emergência (46,7% *versus* 12,4%, p = 0,001). O uso de outros testes diagnósticos foi significativamente maior no grupo submetido à CTA (97% *versus* 82%, p < 0,001). No entanto, apesar do custo mais elevado associado ao maior número de cateterismos e revascularizações, os custos globais foram muito similares entre os dois grupos, devido ao menor tempo de permanência hospitalar (p = 0,65).

Em resumo, a utilização da CTA é uma estratégia segura na avaliação de pacientes com dor torácica aguda de risco baixo a intermediário, com troponina convencional negativa (sem critérios de alta sensibilidade), reduzindo a taxa e o tempo de internação e, provavelmente, os custos. O impacto sobre o número de procedimentos invasivos e a taxa de revascularização ainda é conflitante, apesar de não aumentar mortalidade.^189 , 190^

O HEART *score,* para estratificação da dor torácica em pacientes na sala de emergência, tem ganhado amplo uso com o apoio das atuais diretrizes. A associação do HEART *score* com a CTA foi avaliado por alguns *trials* .^190 – 193^ Em uma análise dos principais estudos que utilizaram essa ferramenta na predição de eventos cardiovasculares em 30 dias, verificou-se que em pacientes com HEART *score* entre 3 a 6 pontos, quando direcionados para a CTA, a tomografia mostrou uma capacidade do método para diagnóstico de estenose coronária acima de 50%, com sensibilidade de 97,8%, especificidade de 84,1% e valor preditivo negativo de 99,6%.^193^ Dessa forma, o uso associado do HEART *score* à CTA de coronárias tem sua validação, principalmente em pacientes de baixo a intermediário risco, com elevado valor preditivo negativo para eventos cardiovasculares, especialmente naqueles estratificados com HEART *score* entre 3 a 6.

Portanto, a utilização da CTA na avaliação de pacientes com dor torácica aguda de risco baixo a intermediário, com ECG não diagnóstico e marcadores de injúria miocárdica convencionais (ou seja, não TCas) abaixo do percentil 99 ("negativos"), tem a indicação como Classe I, com nível de evidência A.

O uso da CTA de coronárias na sala de emergência, em pacientes com dor torácica associada a elevação da troponina convencional e, pelo menos, um fator de risco clínico cardiovascular, foi avaliado pelo VERDICT *trial* ( *Very Early versus Deferred Invasive Evaluation Using Computerized Tomography* ).^192^ Este estudo comparou a capacidade da CTA em identificar estenose coronária ≥ 50%, em relação à estratégia convencional com uso do cateterismo, tanto nas primeiras horas (inferior a 12 horas) de internação, como na fase tardia, após 48-72 horas. O valor preditivo negativo foi similar nas duas estratégias para identificar lesões ≥ 50% (VPN de 90,9% com 12 horas) e com alto valor preditivo positivo para lesões multiarteriais. Portanto, nesse estudo, o uso da CTA em pacientes com alterações da troponina teve alta acurácia para afastar doença coronária significativa e auxiliar no manejo clínico desses pacientes de forma precoce.

Entretanto, a adoção dessa estratégia não foi testada em seu impacto clínico e prognóstico após alta. Por isso, o RAPID-CTCA *trial (Rapid Assessment of Potential Ischaemic Heart Disease with CT Coronary Angiography* )^195^ testou a estratégia do uso de CTA *versus* manejo convencional em pacientes com diagnóstico de síndrome coronariana sem elevação de ST (com elevação de troponina convencional). Os dados deste estudo mostram que a estratégia com uso de CTA não reduziu o desfecho primário proposto (mortalidade por todas as causas ou IAM tipo 1 ou 4b no período de 1 ano), com incidência de 5,8% para CTA *versus* 6,1% para tratamento convencional (p = 0,65) ou revascularizações ( *odds ratio* 1,03; IC 0,87-1,21). Porém, reduziu o número de cateterismos ( *odds* ratio 0,81; IC 0,72-0,92), à custa do aumento discreto do tempo de internação de 2 para 2,2 dias.

O uso da TCas tem ganhado espaço nas salas de emergência por trazer segurança para a alta hospitalar quando negativas.^88^ Poucos estudos têm avaliado o uso da CTA nesse contexto. O BEACON *trial (Better Evaluation of Acute Chest Pain with Computed Tomography Angiography* ), estudo multicêntrico randomizado, avaliou o uso da CTA na sala de emergência em pacientes de baixo a intermediário risco que após troponina de alta sensibilidade negativa eram randomizados para realização de CTA ou abordagem padrão na sala de emergência, tendo como objetivo primário avaliar o número de revascularizações em 30 dias.^191^ O uso da CTA nesse cenário não resultou em diferenças no número de revascularizações ou de SCA não detectadas e nem no número de altas da unidade de emergência (65% *versus* 59%, p = 0,16), e apresentou tempo similar de internação hospitalar em ambos os grupos (6,3 horas). Porém, o uso de CTA conseguiu reduzir os custos de atendimento (337 v *ersus* 511 euros, p < 0,01) e testes adicionais após alta hospitalar (4% *versus* 10%, p < 0,01).^174 , 188 – 195^

Portanto, a CTA, aplicada no início da investigação de suspeita de SCA na sala de emergência, é segura e associada a menos testes e menores custos, devido à menor necessidade de investigação complementar a nível ambulatorial. No entanto, em pacientes com dosagens de troponina de alta sensibilidade negativas, a tomografia de coronárias não identificou mais pacientes com DAC significativa requerendo revascularização coronariana, nem encurtou a internação hospitalar ou permitiu maior proporção de altas a partir do PS, quando comparada à estratégia convencional. Por outro lado, o estudo randomizado PRECISE-CCTA ( *Prospective Randomized Trial of the Optimal Evaluation of Cardiac Symptoms and Revascularization* )^196^ avaliou pacientes com dor torácica na sala de emergência que apresentavam níveis detectáveis de troponina de alta sensibilidade entre 5ng/L a 14ng/L (níveis considerados limítrofes, porém ainda dentro do padrão normal e não diagnóstico para infarto sem supra,). Estes casos foram submetidos à CTA após a alta e apresentaram maior prevalência tanto de DAC não obstrutiva (71,9% *versus* 43,4%; *odds ratio* 3,33) quanto de DAC obstrutiva (29,9% *versus* 19,3%; *odds ratio* 1,79), independentemente da característica da dor torácica. Portanto, pacientes com dor torácica, especialmente com níveis limítrofes de troponinas cardíacas de alta sensibilidade, sem diagnóstico de IAM, podem se beneficiar da realização da CTA após alta, por terem a oportunidade de receber um tratamento eficaz na prevenção de eventos futuros relacionados à DAC em nível ambulatorial. O TARGET-CCTA (NCT03952351) ( *Trial of On-Site Computed Tomography–Derived Fractional Flow Reserve to Guide Management of Stable Coronary Artery Disease* ) está em andamento para avaliar se o tratamento precoce da DAC identificada pela tomografia, em pacientes com elevações intermediárias de troponina de alta sensibilidade, apresenta impacto na prevenção de eventos futuros após 36 meses da alta.

Alguns estudos têm avaliado o uso da CTA de coronárias no contexto de uma troponina de alta sensibilidade elevada. O CARMENTA *trial (CARdiovascular Magnetic rEsoNance imaging And computed Tomography Angiography* )^197^ avaliou se o uso da CTA ou RM seria seguro, em pacientes com troponina de alta sensibilidade positiva, para melhor selecionar os pacientes que se beneficiariam do cateterismo no diagnóstico de lesões críticas, em relação à abordagem conservadora. Ressaltam-se importantes fatores de exclusão como idade acima de 85 anos e alterações dinâmicas do ST, nesse estudo. O estudo demonstrou ser seguro o uso de CTA ou RM nesse contexto, antes do cateterismo, quando comparado à rotina clínica para selecionar os pacientes com dosagens de troponina de alta sensibilidade positivas (com elevações baixa a intermediária) para o cateterismo e sem aumento de eventos cardiovasculares. O uso da CTA em pacientes com elevações de troponina de alta sensibilidade até níveis intermediários (média do estudo de 78 ng/mL) e sem indicadores de alto risco reduziu a necessidade de cateterismo de forma segura, quando comparado à estratégia padrão ( *odds ratio* 0,66, p < 0,001), sem aumento de eventos cardiovasculares após 1 ano de acompanhamento (p = 0,265).

Estudos tentaram avaliar o uso do escore de cálcio como forma de predizer estenose coronária na sala de emergência. Subanálises do CORE 64^198^ mostraram baixo valor preditivo negativo (VPN 0,62), sendo que até 39% dos pacientes de alto risco com SCA apresentavam escore de cálcio de zero, e 46% tinham valores inferiores a 100 unidades Agatston. Portanto, o uso do escore de cálcio na sala de emergência para predizer lesões coronárias significativas não deve ser usado, à luz dos estudos atuais.

##### 4.4.4.1. Descarte Triplo

A CTA pode também ser utilizada na sala de emergência para avaliar o diagnóstico diferencial das SCAs, especialmente em casos de suspeita de embolia pulmonar e SAA.

A angiotomografia em "protocolo para tromboembolismo pumonar" apresenta-se como método de imagem não invasivo mais utilizado para os casos com probabilidade pré-teste intermediária a alta. Importante reforçar limitações para análise de casos de embolia que acometem somente vasos pulmonares de menor diâmetro (subsegmentares).

Quando há suspeita clínica de SAA, a confirmação diagnóstica deve ser rápida e precisa já que a doença tem elevada mortalidade imediata e o tratamento definitivo é, geralmente, cirúrgico. A decisão de utilização de um determinado método de imagem na SAA deve ser baseada não só na sua acurácia diagnóstica, mas também na sua disponibilidade imediata e na experiência dos emergencistas e ecocardiografistas/radiologistas com esse(s) método(s).

Além dos exames específicos de acordo com a suspeita de embolia pulmonar, SAA ou SCA, há protocolos de aquisição que podem obter informações relativas às artérias coronárias, à aorta e às artérias pulmonares, permitindo a avaliação de SAA e TEP, além de permitir análise de outras alterações torácicas (pneumonias, traumas etc.).^199 – 203^ Essa abordagem recebe o nome de descarte triplo ( *triple rule-out* ). Entretanto, mesmo com técnicas otimizadas, o protocolo de aquisição para o descarte triplo é menos eficiente do que os protocolos individuais para avaliação das artérias coronárias, aorta e artérias pulmonares. Portanto, os protocolos de descarte triplo só devem ser utilizados em situações específicas, nas quais a avaliação clínica é incapaz de direcionar a investigação diagnóstica.

Em resumo, a CTA possibilita, de forma não invasiva, avaliar com alta acurácia obstruções epicárdicas e identificação de placas ateroscleróticas, o que gera alto valor preditivo negativo na investigação clínica do paciente sem diagnóstico estabelecido de IAM ( Tabela 36 ).

**Tabela 36:** Recomendações da angiotomografia das artérias coronárias na suspeita de síndrome coronariana aguda

	Classe de recomendação	Nível de evidência
Avaliação de pacientes com suspeita de SCA de risco baixo ou intermediário com ECG normal ou não diagnóstico e marcadores de injúria miocárdica normais ou alterados, porém sem definição de infarto miocárdico * .	I	A
Avaliação de pacientes com dor torácica aguda pela técnica do descarte triplo ( *triple rule-out* ).	IIb	B
Avaliação de pacientes com suspeita de SCA e critérios de risco alto.	III	C
Avaliação de pacientes com diagnóstico definitivo de infarto do miocárdio	III	C

SCA: síndrome coronariana aguda; ECG: eletrocardiograma.

* Se kit disponível for a troponina convencional (sem critérios de alta sensibilidade), a aplicação da CTA nos casos negativos reduz a taxa e o tempo de internação; nos locais com troponina de alta sensibilidade, a CTA ajuda em casos sem definição diagnóstica, incluindo aqueles com elevações até níveis intermediários (reduz necessidade de cateterismo, sem aumento de eventos cardiovasculares após 1 ano de acompanhamento).

#### 4.4.5. Ressonância Magnética Cardiovascular

##### 4.4.5.1. Detecção de Isquemia Miocárdica

Atualmente, a presença de isquemia do miocárdio pode ser detectada através da perfusão de primeira passagem sob efeito do estresse farmacológico e em repouso, ou através da avaliação da contratilidade através da indução de isquemia com dobutamina, sendo a primeira com maior sensibilidade e a mais indicada no cenário de dor torácica, e a segunda mostrando maior especificidade.^204 – 206^

Considerando apenas a avaliação da perfusão miocárdica, alguns estudos recentes colocam a RM em posição de destaque para o diagnóstico nesse cenário, incluindo o aspecto de predição prognóstica. O estudo MR-INFORM ( *Magnetic Resonance Perfusion Imaging or Fractional Flow Reserve in Coronary Disease* ), publicado em 2019, avaliou 918 pacientes sintomáticos e comparou diferentes estratégias de investigação, demonstrando que a estratégia com RM não apresenta inferioridade em relação à estratégia com utilização da RFC invasiva (3,7% para RFC *versus* 3,6% para RM), sem diferença em 12 meses para desfechos primários.^207^ Já o estudo SPINS *(Stress CMR Perfusion Imaging in the United States* ), publicado também em 2019, avaliou 2.349 pacientes com dor torácica e demonstrou que ausência de isquemia ou realce-tardio está associado a número baixo de eventos cardiovasculares em até 5 anos após o exame de RM.^208^

A RM também pode ser utilizada em pacientes com dor precordial, biomarcadores normais e ECG não diagnóstico, demonstrando sensibilidade de 100% e especificidade de 93% na detecção de eventos cardiovasculares futuros.^204 – 209^

##### 4.4.5.2. Diagnóstico Diferencial de Troponina Positiva com Coronárias Normais (TINOCA / MINOCA)

Embora o diagnóstico de IAM esteja habitualmente ligado à presença de obstrução coronária, sabemos que existe um considerável número de pacientes com SCA associados a artérias coronárias angiograficamente normais (entre 6-8%) (190). O diagnóstico de MINOCA requer documentação de um IAM e coronariografia invasiva ou CTA sem obstrução significativa.^56 , 77 , 88^

O diagnóstico de MINOCA, assim como o diagnóstico de qualquer tipo de IAM, necessita da presença de um mecanismo isquêmico responsável pela lesão miocárdica (além de excluir causas não isquêmicas, como miocardites ou síndrome de Takotsubo).

Três características necessitam estar presentes para que o diagnóstico de MINOCA possa ser confirmado:^56 , 77 , 88^ 1) os mesmos critérios diagnósticos de IAM (cenário clínico com biomarcadores cardíacos alterados); 2) angiografia normal ou lesão < 50%; 3) que não se identifique outra causa clínica que possa ser responsável pelos achados compatíveis com a presença de lesão miocárdica (por exemplo miocardite ou embolia pulmonar). Muitos pesquisadores consideram MINOCA, assim como IC, um diagnóstico em andamento ( *working diagnosis* ) por muitas vezes haver dificuldade na identificação precisa da etiologia para correta orientação terapêutica.^56 , 77 , 88^ Assim alguns diagnósticos diferenciais podem ser possível etiologia, visto cumprirem os princípios característicos de IAM tipo I ou II. Incluem-se doenças isquêmicas decorrentes da placa coronariana (erosão, rutura ou ulceração), dissecção coronária, tromboembolismo, espasmo coronário microvascular, embolia coronária, além das cardiomiopatias inflamatórias (miocardite de qualquer etiologia), síndrome de Takotsubo ou mesmo embolia pulmonar. Dessa forma, o diagnóstico preciso de MINOCA é fundamental para escolher-se a melhor opção terapêutica para pacientes isquêmicos e não isquêmicos.^56 , 77 , 88^ Pela sua complexidade diagnóstica, foi sugerido englobarem-se esses diagnósticos sob a denominação inicial de TINOCA, que seriam síndromes de elevação de troponina sem obstruções coronarianas, por terem os níveis elevados de troponina como marcador comum.

Essa denominação pode ser subcategorizada em causas isquêmicas (MINOCA), causas miocárdicas (miocardites, por exemplo) e causas extracardíacas (embolia pulmonar, por exemplo).^88^

A RM consiste em uma das mais importantes ferramentas na determinação da etiologia dos casos de TINOCA, podendo definir até 74% desses casos.^209^ O realce tardio, quando presente, permite a localização da área de lesão miocardica, além de fornecer evidências dos mecanismos envolvidos. Adicionalmente, a RM permite identificar os pacientes com pior prognóstico, e, quando realizada, muda a terapêutica em aproximadamente metade dos casos de MINOCA.^204^

Dessa forma, a utilização de um recurso diagnóstico tão preciso que permite diagnosticar infartos com até 1 grama de necrose miocárdica,^205^ permite personalizar adequadamente a terapia médica (incluindo prevenção secundária), podendo prevenir novos eventos isquêmicos e evitar prescrição desnecessária de drogas com seus respectivos efeitos colaterais, como sangramento no caso de uso de antiagregantes plaquetários.^209^

Aproximadamente 23% dos casos diagnosticados como MINOCA são decorrentes de aterosclerose coronariana em sua forma não obstrutiva, decorrentes de erosão ou ulceração de placas com consequente trombose momentânea e recanalização do vaso comprometido, associado a vasoespasmo prolongado.^206^ A RM consegue confirmar o diagnóstico de infarto pela técnica de realce tardio, diferenciando sua etiologia isquêmica pela visualização do acometimento obrigatório do subendocárdio como marca da etiologia isquêmica. Adicionalmente, permite diferenciar de outras lesões pelas técnicas que identificam edema, (como em casos de miocardite), seja pelas técnicas tradicionais ponderadas em T2, seja pelos novos mapas paramétricos (Mapas T1 e T2).^210^

Outra causa relevante de TINOCA, e que se confunde com o diagnóstico de MINOCA, são as miocardites. Correspondem a aproximadamente 29% dos casos,^7^ sendo a etiologia viral a mais frequente, e que ganhou maior repercussão após a pandemia de Covid-19, evidenciando alterações miocárdicas em aproximadamente 50% dos casos recuperados.^211 – 213^

Aproximadamente 16% destes casos sem obstrução coronária se apresentarão como síndrome de Takotsubo^206^ (síndrome do coração partido) e, embora inicialmente reconhecida como "benigna" devido à reversão das áreas discinéticas, acompanhamento evolutivo dessa síndrome demonstra que o prognóstico pode ser desfavorável.^214^ A RM se mostra como excelente ferramenta diagnóstica, uma vez que permite a identificação das áreas discinéticas em qualquer segmento do VE (embora a discinesia apical transitória seja a mais frequente manifestação na síndrome de Takotsubo). Além disso, permite caracterizar as áreas de edema miocárdico com as técnicas ponderadas em T2 ou mais recentemente com os mapas paramétricos T1 e T2, sendo colocada como método de escolha em consenso publicado recentemente.^215^ A fase inflamatória, em que é possível fazer a detecção do edema miocardico, costuma desaparecer em 3 meses.^216^ RM de controle, nesses casos, pode ser solicitada para verificação de reversão da área discinética, bem como desaparecimento do edema miocárdico, com a ratificação do diagnóstico. Habitualmente não se observam áreas de realce tardio nesta síndrome; entretanto, na fase aguda, pequenas áreas de realce tardio podem ser visualizadas nas áreas discinéticas, pelo aumento do espaço intersticial das áreas inflamadas.

Outros diagnósticos possíveis são menos frequentes e podem ser observados na investigação de TINOCA, como cardiomiopatia hipertrófica, em aproximadamente 3% dos casos, cardiomiopatia dilatada não isquêmica, em 2% dos casos, e amiloidose, em menos de 5% dos casos.^206 , 210 , 216 , 217^

Assim, a excelente *performance* da RM em realizar o diagnóstico preciso das áreas de infarto relacionadas à MINOCA faz com que a ressonância magnética cardíaca seja considerada na investigação diagnóstica, com classe de recomendação I, nível de evidência B^200 , 202^ ( Tabela 37 ).

**Tabela 37:** Pesquisa de doença arterial coronariana pela ressonância magnética – isquemia miocárdica

	Classe de recomendação	Nível de evidência
Investigação de doença isquêmica em pacientes com dor torácica aguda e probabilidade pré-teste intermediária de DAC.	I	B
Investigação de isquemia miocárdica em pacientes revascularizados (cirurgicamente ou de forma percutânea), com sintomatologia sugestiva de DAC obstrutiva.	I	B
Diagnóstico diferencial de síndromes de elevação de troponina com coronárias não obstrutivas (TINOCA).	I	B
Avaliação de pacientes com DAC não obstrutiva conhecida, sem elevação de troponina, porém com suspeita de INOCA.	IIa	C

DAC: doença arterial coronariana; TINOCA: troponin-positive non-obstructive coronary arteries; INOCA: ischemia with non-obstructive coronary arteries.

* Definida como estenose ≥ 50% em TCE, e triarteriais com acometimento coronariano proximal.

##### 4.4.5.3. Miocardites

A miocardite é uma doença inflamatória do músculo cardíaco que pode ocorrer em consequência de infecção, exposição a substâncias tóxicas ou ativação do sistema imune.^218^ A etiologia infecciosa viral é a mais prevalente, sendo o quadro clínico bastante variável (desde indivíduos assintomáticos até morte súbita). Em geral, cursa com dor precordial, dispneia, fadiga, palpitações e síncope.^219^ Alterações eletrocardiográficas diversas estão presentes em 85% dos casos (ex: supradesnível do segmento ST, alargamento do QRS ou arritmias), associadas à elevação de marcadores de injúria miocárdica (TCas).^218^

O diagnóstico de miocardite inclui a associação de quadro clínico, exame físico, exames laboratoriais e de imagem. A RM auxilia no diagnóstico sendo um exame sensível às alterações teciduais que ocorrem pela inflamação do miocárdio.^220 , 221^ Os critérios de Lake Louise foram atualizados em 2018 associando as técnicas de mapas paramétricos e volume extracelular, aumentando a acurácia diagnóstica. A inflamação miocárdica aguda pode ser detectada se ao menos um critério de cada categoria estiver presente.^220^ Uma das categorias é o edema miocárdico, identificado por meio de imagens ponderadas em T2 ou mapa de T2; a outra é a injúria miocárdica, identificada por meio de realce tardio, aumento do T1 nativo ou do volume extracelular.^220 , 222^ O mapa de T2 encontra-se mais elevado na fase aguda da miocardite e tende a normalizar ao longo dos meses, é um recurso útil tanto no diagnóstico quanto no monitoramento do tratamento.^223^ O tempo de relaxamento T1 se prolonga por edema intracelular ou extracelular, hiperemia e devido à presença de áreas de fibrose. O volume extracelular pode aumentar em decorrência de expansão do meio extracelular pela inflamação.^220 , 223^ Se ambos os critérios forem positivos, aumentam a especificidade diagnóstica. Por outro lado, se apenas um deles estiver presente em um cenário de suspeição clínica, permite auxiliar no diagnóstico.^220^ Na ausência de realce tardio e quadro clínico positivo, a presença de alteração nos mapas de T1 nativo e volume extracelular pode ser indicativa de injúria miocárdica. Nessa situação, a elevação do T1 nativo em áreas sem realce tardio mostrou elevação na sensibilidade do método, sem aumentar falsos positivos.^216^

Além da utilidade diagnóstica, a RM também pode ser usada para prognóstico. Nesse contexto, a disfunção biventricular resultante de envolvimento miocárdico significativo é o maior preditor de mortalidade. A presença de realce tardio também é preditor de mortalidade, estando mais relacionada ao risco de morte súbita e evolução com dilatação ventricular esquerda e queda de FEVE.^216^ Os pacientes que apresentam FE ≤ 40% associada a realce tardio positivo apresentam um aumento do risco de evento cardiovascular desfavorável de 10% ao ano.^216^

O SARS-CoV2, vírus causador da Covid-19, tem sido frequentemente associado à injúria miocárdica. Elevação de troponina acima do percentil 99 está presente em 62% dos pacientes com Covid-19. No seguimento dos pacientes, o achado mais comum é a disfunção diastólica (55%), e apenas 2,8% apresentaram FEVE reduzida.^224^ Na fase aguda da doença, os achados de RM mais comuns são alterações nos mapas de T1 e T2, alterações pericárdicas (miopericardite) e padrões de realce tardio não coronariano. Os pacientes com sintomas leves ou assintomáticos não apresentaram alterações significativas em relação ao controle.^224^

Atualmente, a realização de RM na suspeita de miocardite é indicação classe I nas diretrizes internacionais e na Diretriz da Sociedade Brasileira de Cardiologia,^90 , 225^ corroborado neste documento ( Tabela 38 ). A incorporação recente dos dados de mapas paramétricos T1 e T2 e volume extracelular eleva a sensibilidade do método.

**Tabela 38:** Indicações de ressonância magnética cardíaca em casos de miocardite

	Classe de recomendação	Nível de evidência
RMC na avaliação da função, geometria e morfologia ventricular na suspeita de miocardite aguda, subaguda e crônica.	I	B
RMC na investigação diagnóstica e prognóstica de miocardite aguda, crônica e/ou suspeita de miocardite prévia.	I	B
RMC no acompanhamento de 4 a 12 semanas do episódio agudo, para diferenciação de evolução complicada *versus* não complicada.	IIa	B
RMC na miocardite fulminante com instabilidade hemodinâmica.	III	B

RCM: ressonância magnética cardíaca.

## 5. Modelo para Implantação das Unidades de Dor Torácica

### 5.1. Investigação de Síndrome Coronariana Aguda em Ambiente Pré-hospitalar

De uma forma geral, de acordo com a Quarta Definição Universal de Infarto Agudo do Miocárdio, não existem diferenças conceituais no diagnóstico, seja ele realizado no ambiente intra ou pré-hospitalar.^77^

Um dos grandes desafios no contexto pré-hospitalar é a agilidade no que diz respeito aos tempos diagnósticos/terapêuticos e à indisponibilidade de alguns exames complementares que auxiliam no diagnóstico, já que tais pacientes são atendidos principalmente em unidades de pronto-atendimento ou nas unidades móveis de serviço médico de emergência (ex: ambulância do SAMU). Boa acurácia e rapidez no diagnóstico são aspectos prioritários na avaliação pré-hospitalar, a fim de otimizar recursos para direcionar atendimentos e determinar transferências de forma mais assertiva ( Tabela 39 ).

**Tabela 39:** Investigação de síndrome coronariana aguda em ambiente pré-hospitalar

	Classe de recomendação	Nível de evidência
Os critérios diagnósticos são os mesmos em ambiente pré-hospitalar (unidade móvel de emergência) ou em uma unidade fixa (por exemplo, pronto-socorro).	I	C
Sistemas de atendimento pré-hospitalar devem ter protocolos para identificação de casos com critérios de suspeita de SCA para direcionar a unidade móvel avançada (com eletrocardiograma, médico e recursos para suporte avançado à vida).	I	B
O eletrocardiograma deve ser realizado em até 10 minutos do primeiro contato médico, seja em nível hospitalar ou no contexto pré-hospitalar.	I	B
O uso de escores de risco, como o *pre* HEART *score* , pode ser útil como ferramenta adicional da estratificação de risco pré-hospitalar.	IIa	B
O uso de troponina *point of care* pré-hospitalar pode ser considerado, quando disponível em casos que apresentem ECG não diagnóstico, especialmente nos casos com baixa probabilidade clínica e tempo adequado para avaliação do biomarcardor.	IIb	B

SCA: síndrome coronariana aguda; ECG: eletrocardiograma.

#### 5.1.1. Percepção do Paciente e Rede Pré-hospitalar Organizada

**Figura 16:**
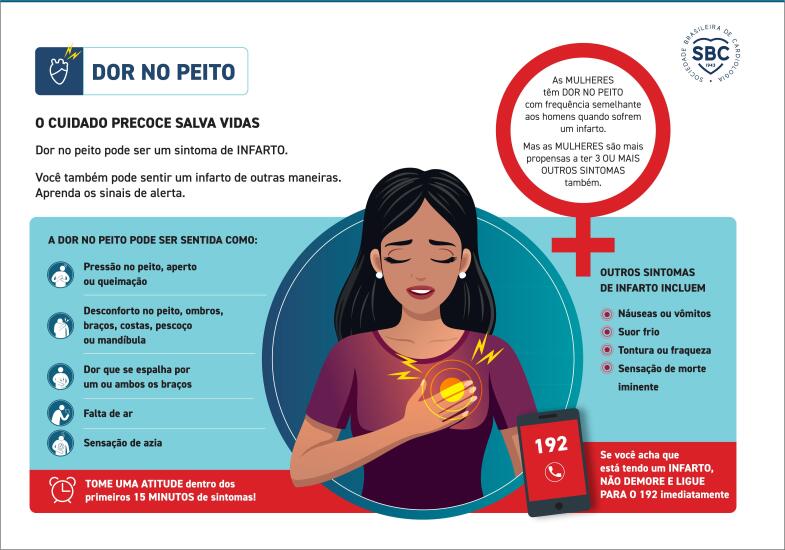
Dor no peito: cuidados precoces salvam vidas (Material educativo para campanhas públicas visando aumentar a percepção dos pacientes sobre os sintomas).

O aforisma de que "tempo é músculo" persiste atual dentro do tratamento do IAM.^226^ Desde os primeiros estudos com trombolíticos evidenciando que, quanto menor o tempo da estratégia de reperfusão, melhores os benefícios em mortalidade, busca-se agilidade desde a percepção dos sintomas pelo paciente até a estratégia de reperfusão.^227^ Campanhas públicas melhoram a percepção do paciente sobre sintomas de IAM, aumentam a procura de serviços de emergência de pacientes com IAM (incluindo maior número de ligações para o serviço pré-hospitalar) o que, finalmente, reduz os tempos na assistência pré-hospitalar ( Figura 16 ).^228^ Vale ressaltar que as mulheres tendem ter um maior tempo entre a percepção dos sintomas e o acionamento do pré-hospitalar em relação aos homens, com maiores taxas de mortalidade intra-hospitalar, destacando a importância deste público específico.^229^ Implementar um serviço pré-hospitalar de ambulância para o atendimento de tais pacientes leva à redução de taxa de mortalidade global e intra-hospitalar nas regiões territoriais contempladas,^230^ com destaque para as linhas de cuidado em IAM *,* já incorporadas e estimuladas em portarias do serviço público.^231^

#### 5.1.2. Papel do Eletrocardiograma no Ambiente Pré-hospitalar

Assim como no atendimento hospitalar, o ECG deve ser realizado em até 10 minutos do primeiro contato médico, também no contexto pré-hospitalar. A cada 30 minutos de atraso nas estratégias de reperfusão, há um aumento de 7,5% na mortalidade desses pacientes.^91^ Uma metanálise reunindo mais de 80 publicações verificou que o uso do ECG pré-hospitalar implicou em um menor número de óbitos, menor tempo à estratégia de reperfusão, menor tempo de internação hospitalar e maior proporção de pacientes que obtiveram um tempo porta/FMC – reperfusão < 90 minutos.^232^ Vale ressaltar que diversos estudos apontam que o uso de ferramentas de telemedicina (tele-ECG) beneficiam o atendimento desses pacientes, incluindo redução de eventos cardiovasculares.^232 – 236^

#### 5.1.3. Papel da Troponina no Ambiente Pré-hospitalar

Na ausência de critérios eletrocardiográficos de SCA, o uso da troponina é de fundamental importância no diagnóstico e estratificação de risco desses casos. Seu uso no contexto hospitalar já é bem estabelecido, mas no contexto pré-hospitalar, ainda necessita de maior robustez de evidências.

Vários estudos avaliaram seu uso nas ambulâncias como uma forma de triagem nas transferências para os centros de referência. De uma maneira geral, foram estudos pequenos que incluíram pacientes com dor torácica, e utilizou-se a TCas *point-of-care* (Tn-POC) em conjunto com o HEART *score* modificado, tendo capacidade de "descarte" em pacientes com SCA de baixo risco.^237 – 239^ Em 2023, Camaro et al. publicaram o estudo ARTICA ( *Acute Rule-Out of Non-ST-Segment Elevation Acute Coronary Syndrome in the (Pre)hospital Setting by HEART Score Assessment and a Single Point-of-Care Troponin* ),^240^ sob o mesmo enfoque, sendo o *end point* primário custos hospitalares, tendo demonstrado que a estratégia do uso da Tn-POC pré-hospitalar resultou em menor custo, com eventos cardiovasculares comparáveis entre a estratégia pré-hospitalar *versus* serviço de emergência hospitalar.

#### 5.1.4. Papel dos Escores de Estratificação de Risco

No atendimento desses pacientes no contexto pré-hospitalar, basicamente a história clínica, exame físico e a realização do ECG seguem como padrão assistencial inicial, a fim de identificar os IAMCSST e deflagrar as estratégias de reperfusão. Contudo, na ausência de elevação de ST (ou alteração equivalente), não existe uma estratificação de risco validada que atue como uma "triagem" no direcionamento de pacientes direto para os hospitais com suporte de hemodinâmica ou para unidades de atendimento clínico inicial de menor porte. Sagel et al., em publicação recente avaliando o uso do HEART *score* e da Tn-POC na estratificação de tais pacientes, propuseram um novo escore, *pre* HEART *score* , como nova ferramenta estratificadora no ambiente pré-hospitalar, com boa acurácia em relação aos demais (VPN 99,3% [98.1-99.8], VPP 49,9% [42-56.9] e AUC 0.82 [0.82-0.88]).^241^ De uma forma geral, preconiza-se, espelhando-se na propedêutica hospitalar, que se utilize uma combinação de escores, sendo que o *pre* HEART *score* surge como escore promissor no ambiente pré-hospitalar.^242^

#### 5.1.5. Outras Ferramentas Diagnósticas

São raros os estudos que utilizam o ecocardiograma transtorácico/POCUS como ferramenta no auxílio diagnóstico das SCAs no atendimento pré-hospitalar. Em um dos estudos direcionado na temática, os autores sugerem o seu potencial uso, porém críticas à metodologia, principalmente quanto à acurácia e treinamento dos examinadores, são imperativas.^243 , 244^ Mesmo assim, a Sociedade Europeia de Imagem Cardiovascular recomenda o uso do ecocardiograma emergencial em pacientes com dor torácica aguda sugestiva de SCA – porém estudos robustos e sua padronização no contexto pré-hospitalar são necessários para sua incorporação prática.^243 – 248^ Lembrar que, além de SCA, outros diagnósticos também podem ser considerados como dissecção de aorta e TEP (escores, D-dímero-POC e o próprio POCUS podem ajudar a direcionar para o serviço com melhor recurso de acordo com suspeita diagnóstica).

### 5.2. Modelo para Implantação das Unidades de Dor Torácica: Evidências para Melhores Práticas

A ênfase do atendimento da dor torácica tem sido não somente o reconhecimento do diagnóstico, mas a identificação e pronto manejo das condições mais graves e de risco potencial de vida. O desafio, usualmente, é grande e recai sobre o médico emergencista e nas unidades de pronto atendimento, em condições sobrecarregadas e com recursos, às vezes, escassos. Nesse contexto, nas últimas décadas foram fortalecidas as estratégias para auxiliar em uma rápida e correta separação de pacientes em alto risco de desenvolver complicações daqueles de baixo risco que não precisam ficar nas unidades dedicadas, as quais são consideradas pilares estruturantes do manejo de casos de dor torácica aguda: as UDTs.^8 , 21 – 23 , 246^

A avaliação inicial de todo paciente com dor torácica deve permitir responder a três perguntas-chave: 1) qual a probabilidade dos sinais e sintomas serem devidos a uma SCA?; 2) quais as chances para o desenvolvimento de eventos cardíacos adversos, como infarto do miocárdio, acidente vascular cerebral, IC, sintomas recorrentes de isquemia ou arritmias graves?; 3) como otimizar recursos adicionais para decisão de diagnóstico e de alocação do paciente? Por meio da história clínica, ECG e marcadores de injúria miocárdica é possível responder a estas questões na maioria dos casos. Entretanto, estudos demonstraram que a precisão, eficiência e agilidade desse modelo sem protocolos e rotinas estruturadas estão relacionadas a risco não desprezível de condutas inadequadas, erros diagnósticos e custo elevado. ^8 , 21 – 23^

As UDTs se difundiram no início dos anos 2000 com objetivo de oferecer cuidado de alta qualidade e rápido para pacientes com dor torácica e baixa probabilidade de SCA, mas não suficientemente baixa para permitir a alta hospitalar. As UDTs são definidas como unidades organizacionais de curta permanência, com o objetivo de facilitar e otimizar o atendimento por meio de protocolos diagnósticos. ^8 , 21 – 23^

Inúmeros estudos observacionais e ensaios clínicos randomizados descreveram que o atendimento em UDT oferece uma maior adesão às condutas baseadas em diretrizes e evidências científicas, com resultados clínicos semelhantes à admissão tradicional em enfermarias, mas com um tempo de permanência menor e custo reduzido. Dados da Austrália, EUA e Alemanha apontam que as melhorias nos processos com a adoção das UDTs levaram à redução do tempo de permanência, das admissões hospitalares e da realização de testes não invasivos, resultando em economias de 20 a 25% aos sistemas de saúde. Várias instituições internacionais e brasileiras implementaram UDTs com características em comum sobre questões organizacionais e logísticas, as quais evoluíram ao longo dos anos.^8 , 21 – 23^ A Tabela 40 descreve as recomendações acerca da implementação de unidades dedicadas à avaliação de pacientes com sintomas de dor torácica aguda (ou equivalente).

**Tabela 40:** Recomendações para implementação de unidades de dor torácica

	Classe de recomendação	Nível de evidência
As UDTs devem ser implementadas em centros de emergência de alto volume e/ou de referência cardiovascular.	I	A
As UDTs devem coletar indicadores de qualidade de forma sistemática, com avaliação contínua de melhorias baseadas nas métricas identificadas.	I	A
As UDTs devem dispor de médicos e profissionais de apoio habilitados para execução dos protocolos e interpretação de ECG.	I	C
As UDTs devem dispor de ECG e marcadores de injúria miocárdica (preferencialmente troponina de alta sensibilidade).	I	A
As UDTs devem dispor de acesso à realização de ecocardiograma de urgência.	I	C
As UDTs devem dispor de acesso a laboratório de hemodinâmica 24/7 (no próprio serviço ou em um sistema integrado de referenciamento de emergência).	I	C
As UDTs devem dispor de protocolos de diagnóstico e tratamento para as principais síndromes de dor torácica aguda potencialmente fatais.	I	C
As UDTs devem dispor de métodos não invasivos para investigação de doença coronária e/ou isquemia.	IIa	C

UDT: unidade de dor torácica; ECG: eletrocardiograma.

#### 5.2.1. Avaliação do Paciente

A obtenção de uma história clínica sucinta e dirigida é essencial na avaliação inicial de pacientes com dor torácica. As características da dor, presença de comorbidades, agravantes e alterações no exame físico permitem identificar risco clínico imediato.^97 – 107^ O escore HEART se mostrou fácil, útil e efetivo para estratificação de risco de paciente que não estabeleceu o diagnóstico.^97 – 101^ Com base na história do paciente, ECG, idade, fatores de risco cardiovascular e valores de troponina, é calculada uma pontuação entre 0 e 10 pontos, representando o risco do paciente de desenvolver um evento cardíaco adverso maior dentro de 6 semanas após apresentação inicial. Uma metanálise de 16 estudos de coorte prospectivos observou uma sensibilidade e especificidade do escore HEART para predizer eventos cardíacos maiores de 0,96 (IC 95% 0,91-0,98; I^2^ = 94,87%) e 0,50 (IC 95% 0,41-0,60; I^2^ = 98,84%), respectivamente. Entretanto, a alta heterogeneidade entre os estudos incluídos é um fator limitante para a interpretabilidade e validade de sua análise combinada nesta metanálise.^247^ Um escore abaixo de 4 e troponinas normais tem um valor preditivo negativo elevado (99%), sugerindo segurança na alta do paciente. Vários estudos demonstraram que o uso de protocolos clínicos, como *Heart Pathway* , ADAPT e EDACS *Pathways* reduziram a necessidade de internação de 20 a 45% de pacientes com suspeita de SCA. Outros escores bem conhecidos, como TIMI e GRACE, também podem ser úteis para estratificação do risco de pacientes em avaliação diagnóstica de SCA, embora tenham sido desenvolvidos e validados como instrumentos de prognóstico na população com diagnóstico de SCA e apresentem *performance* inferior ao HEART em pacientes ainda em rota diagnóstica.^97 – 107 , 247^

#### 5.2.2. Estrutura Organizacional e Recursos Humanos

As UDTs, tradicionalmente, são áreas designadas próximas, ou mesmo dentro, das emergências ou unidades de pronto atendimento, com uma integração entre médico emergencista, cardiologista e as equipes multidisciplinares dedicadas.^248 , 249^ Em um cenário ideal, as UDTs são supervisionadas por cardiologistas, com atendimento por médicos com formação em clínica médica e/ou emergência. Na perspectiva de gestão, o número de leitos de uma UDT deveria ser calculado pelo tamanho do hospital e pelo número de atendimentos na emergência por ano. Estima-se que, para cada 10.000 atendimentos mês, 250-500 serão por dor torácica, dependendo da referência hospitalar; desses, a metade teria indicação de atendimento em uma UDT, sendo necessários de dois a quatro leitos.^250^ As recomendações europeias sugerem um médico por unidade e uma enfermeira a cada quatro a seis leitos, sendo a legislação no Brasil semelhante, de acordo com o grau de cuidado, distribuídos na razão de 1:3 entre enfermeiros e técnicos de enfermagem. Usualmente, são unidades com dois a seis leitos dedicados a esses pacientes, podendo ser uma unidade completa de leitos monitorados. É importante acesso a laboratório de hemodinâmica e procedimentos invasivos, no caso de confirmação diagnóstica de SCA. Para aquelas instituições sem laboratório de hemodinâmica disponível em regime 24/7, termos de colaboração e protocolos de transferência devem estar delineados para casos críticos.

Dentro da construção de uma UDT, um ponto fundamental é a existência de protocolos clínicos escritos e validados para todos os profissionais que atendem esses pacientes. Esses protocolos devem ser atualizados periodicamente, com mecanismos de gestão implementados e vinculados à sua efetiva utilização e acompanhamento de indicadores, com ações de melhoria contínua. Um programa de treinamento continuado deve ser oferecido, com validação e revisão dos protocolos institucionais, treinamento em interpretação de ECG, biomarcadores e métodos não invasivos.

#### 5.2.3. Necessidades Técnicas

O ECG de 12 derivações deve estar disponível em todas UDTs, para pronta realização e repetição quando necessário. Idealmente, o ECG deve ser obtido nos primeiros 10 minutos da chegada do paciente ao hospital, a partir da identificação pela equipe da triagem de casos suspeitos de SCA. As unidades devem estar equipadas com dispositivos não invasivos de monitorização de pressão arterial para cada paciente e monitoramento eletrocardiográfico contínuo, bem como com um desfibrilador e material de ressuscitação cardiopulmonar. Uma central de monitoramento por telemetria ou estação central não é absolutamente essencial, embora desejável. A monitorização do ritmo cardíaco contínua é indicada para pacientes com suspeita de SCA ou alto risco de eventos cardiovasculares. O uso de monitor de segmento ST contínuo permite identificar pacientes com isquemia dinâmica; entretanto, estudos não mostraram valor adicional expressivo em pacientes de risco mais baixo (atualmente, são considerados opcionais em UDT). A mensuração de sinais de vitais a cada 15-30 minutos é recomendada nesse perfil de paciente.

Para detecção de lesão miocárdica é recomendada a dosagem das TCas (T ou I) para pacientes com suspeita de SCA. Alguns estudos testaram protocolos de avaliação de 0-3 horas de intervalo, e outros, de 0 e 1/2 hora de intervalo. A disponibilização desses exames requer um laboratório clínico 24 horas na emergência, com tempo ideal para liberação de resultado inferior a 60 minutos. Para instituições sem laboratório no local, o uso de testes rápidos ( *point of care* ) é uma opção a ser considerada, para não atrasar avaliação e liberação do paciente.

Além desses exames, a UDT deve contar com exames adicionais bioquímicos, radiografia de tórax e métodos alternativos de imagem para elaboração de diagnósticos diferenciais de dor torácica. A CTA tem se mostrado um excelente método para excluir doença coronariana obstrutiva, especialmente no grupo de risco intermediário ou baixo.^251^

Uma tomografia computadorizada de múltiplos canais deve estar disponível para uma investigação mais aprofundada de diagnósticos diferenciais relevantes após a exclusão de SCA (como TEP e SAA) ou para descartar DAC de baixa ou intermediária probabilidade.

Para pacientes com risco intermediário ou baixo de complicação coronária tem sido aceita a realização de um TE, que é uma ferramenta útil e disponível na maioria dos hospitais.^252 , 253^ Esta recomendação baseia-se no valor deste teste para refinar a probabilidade diagnóstica e nas respectivas informações prognósticas. No entanto, considerando que a prevalência de doença coronariana é baixa nesse grupo de pacientes com risco intermediário e baixo de DAC, a probabilidade de resultados falsos positivos é alta. Por isso, os protocolos atuais não consideram mais esse exame como recomendação essencial. A complementação diagnóstica com outros métodos não invasivos de isquemia – cintilografia miocárdica, ecocardiografia de estresse e RM – acrescenta informações e auxilia a identificar pacientes com DAC, podendo ser utilizados nas UDTs ou em nível ambulatorial precoce após a alta.

#### 5.2.4. Terapias Específicas

Os pacientes sem diagnóstico estabelecido, porém com suspeita de SCA, devem ser tratados inicialmente apenas com ácido acetilsalicílico (AAS) até elucidação do caso (na ausência de contraindicações ou situações de risco de sangramento). Se suspeita de TEP, deve-se considerar o início da anticoagulação em situações de alta probabilidade pré-teste (Wells, Genebra). Medicações sintomáticas e fármacos para estabilização hemodinâmica também podem ser administrados conforme necessidade.

À medida que um diagnóstico é identificado, os protocolos específicos devem ser adotados, como para SCA, SAA, TEP etc. Pacientes de baixo risco, com resultados negativos em toda avaliação e sem diagnóstico estabelecido, podem ter alta da UDT para investigação ambulatorial precoce.

#### 5.2.5. Indicadores de Gestão

A participação da UDT em registros locais e nacionais deve ser incentivada para coleta prospectiva de indicadores de qualidade, adesão a práticas baseadas em evidências e desempenho.^249 , 250^ Alguns indicadores internacionais são usualmente empregados para acompanhamento do desempenho dessas unidades, entretanto cada instituição deve implementar os seus indicadores de acordo com a disponibilidade das informações ou oportunidades de melhorias. Abaixo, estão alguns indicadores previstos (tanto para dor torácica como para casos confirmados de SCA).

- Tempos de atendimento:
  ➢ Tempo porta-eletrocardiograma (TPE) (ideal até 10 minutos);
  ➢ Tempo porta-agulha ou porta-balão (para IAM com supra-ST);
  ➢ Permanência na UDT;
  ➢ Transferência da UDT para outra unidade ( *boarding time* para terapia intensiva ou internação).
- Prescrição de dupla antiagregação plaquetária na alta e de AAS na admissão;
- Prescrição de estatina de alta intensidade na alta hospitalar;
- Percepção do paciente com o atendimento.
- Outros indicadores podem ser acrescentados em situações específicas (por exemplo, paciente que evolui com IC deve receber alta com terapia para IC).

É considerado boa prática a produção de relatórios em intervalos regulares (por exemplo, trimestralmente), cujos resultados devem ser documentados em reuniões de equipe e discussão de casos. Mecanismos de *feedback* , que reflitam os resultados e a qualidade do manejo dos pacientes na unidade, também devem ser introduzidos.

#### 5.2.6. Avanços Tecnológicos

Muitas pesquisas têm apontado que é possível melhorar ainda mais o atendimento e a estratificação de pacientes com dor torácica, de modo que os pacientes sejam estratificados mais precocemente, com mais segurança e com acesso mais ágil a quem precisa. Por exemplo, uso de ECG no domicílio ( *wearable* ), uso de troponina *point-of-care* e aplicação do escore HEART pré-hospitalar podem orientar pacientes de baixo risco para exames adicionais em nível ambulatorial ou orientar pacientes de alto risco para ir diretamente a hospitais de referência cardiológico, com laboratório de hemodinâmica e centros de terapia intensiva. O uso de algoritmos sistematizados de atendimento, integrados aos prontuários do paciente, tem sido implementado para reduzir erros e acelerar os processos de atendimento, muitas vezes com suporte de inteligência artificial.^254 , 255^

**Figura 17:**
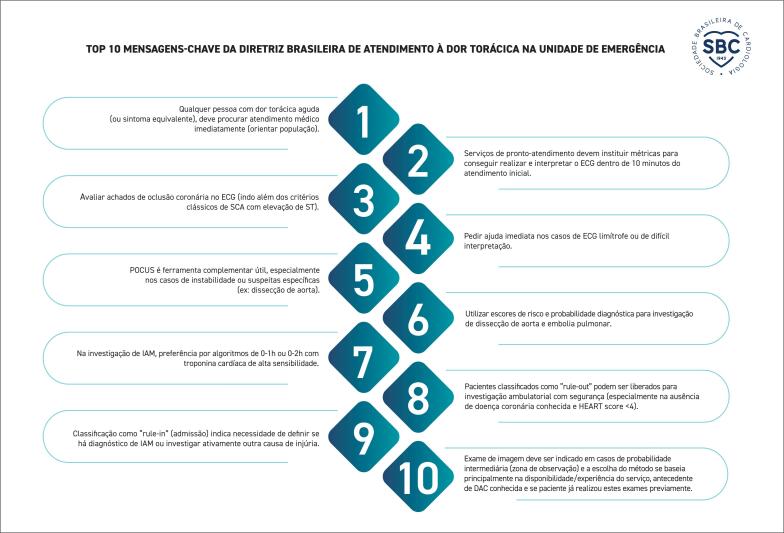
10 Mensagens Principais da Diretriz de Atendimento à Dor Torácica na Unidade de Emergência. ECG: eletrocardiograma; SCA: síndrome coronariana aguda; POCUS: Ponint of Care Ultrasound; IAM: infarto agudo do miocárdio; DAC: doença arterial coronária.

Finalmente, todo o conteúdo desta diretriz ( Figura 17 ) só será plenamente aplicado e haverá possibilidade de melhoria contínua, caso sejam implementados protocolos locais com métricas e acompanhamento pela equipe assistencial ( Tabela 40 ).
