# Supplementary material for: Diretriz Brasileira de Atendimento à Dor Torácica na Unidade de Emergência – 2025
Source: Arq Bras Cardiol. 2025 Oct 13;122(9):e20250620. [Article in Portuguese] doi: 10.36660/abc.20250620 (PMC12981354; doi:10.36660/abc.20250620)
Supplement: Translation

# Translation: Brazilian Guideline for the Evaluation and Diagnosis of Chest Pain in the Emergency Department - 2025

**DOI:** 10.36660/abc.20250620i

**Authors:** Pedro Gabriel Melo de Barros e Silva, Alexandre de Matos Soeiro, Carlos Eduardo Ornelas, Gilson Soares Feitosa-Filho, Renato D. Lopes, Danielli Oliveira da Costa Lino, Remo Holanda de Mendonça Furtado, Hélio Penna Guimarães, André Volschan, Bruno Ferraz de Oliveira Gomes, Carisi Anne Polanczyk, Carlos Eduardo Rochitte, Carlos Vicente Serrano, Cláudio Marcelo Bittencourt das Virgens, Claudio Tinoco Mesquita, Edgardo Jorge Menendez, Eduardo Leal Adam, Fabio Mastrocola, Fábio Serra Silveira, Felipe Souza Maia da Silva, Giovanni Possamai Dutra, Humberto Graner Moreira, Isly Maria Lucena de Barros, João Luiz Fernandes Petriz, José Roberto de Oliveira Silva, Julio Flavio Meirelles Marchini, Louis Nakayama Ohe, Ludhmila Abrahão Hajjar, Maria Camila Lunardi, Mucio Tavares de Oliveira, Nivaldo Menezes Filgueiras, Odilson Marcos Silvestre, Paolo Blanco Villela, Paulo Rogério Soares, Pedro Paulo Nogueres Sampaio, Renée Sarmento de Oliveira, Ronaldo de Souza Leão Lima, Sandro Pinelli Felicioni, Sergio Timerman, Tatiana de Carvalho Andreuci Torres Leal, Wilson Mathias

**Affiliations:** 1 São Paulo SP Brazil Hospital do Coração (Hcor), São Paulo, SP – Brazil; 2 São Paulo SP Brazil Brazilian Clinical Research Institute, São Paulo, SP – Brazil; 3 São Paulo SP Brazil Centro Universitário São Camilo, São Paulo, SP – Brazil; 4 São Paulo SP Brazil Instituto do Coração (Incor) do Hospital das Clínicas da Faculdade de Medicina da Universidade de São Paulo (HCFMUSP), São Paulo, SP – Brazil; 5 Belo Horizonte MG Brazil Hospital Felício Rocho, Belo Horizonte, MG – Brazil; 6 Salvador BA Brazil Escola Bahiana de Medicina e Saúde Pública, Salvador, BA – Brazil; 7 Salvador BA Brazil Hospital Santa Izabel, Salvador, BA – Brazil; 8 Salvador BA Brazil Santa Casa de Misericórdia da Bahia, Salvador, BA – Brazil; 9 Durham USA Duke University Medical Center, Durham – USA; 10 Fortaleza CE Brazil Hospital de Messejana Dr. Carlos Alberto Studart Gomes, Fortaleza, CE – Brazil; 11 São Paulo SP Brazil Hospital Israelita Albert Einstein, São Paulo, SP – Brazil; 12 São Paulo SP Brazil Universidade de São Paulo (USP), São Paulo, SP – Brazil; 13 Rio de Janeiro RJ Brazil Hospital Pró-Cardíaco, Rio de Janeiro, RJ – Brazil; 14 Rio de Janeiro RJ Brazil Hospital Barra D’Or, Rio de Janeiro, RJ – Brazil; 15 Rio de Janeiro RJ Brazil Hospital Universitário Clementino Fraga Filho, Universidade Federal do Rio de Janeiro (UFRJ), Rio de Janeiro, RJ – Brazil; 16 Porto Alegre RS Brazil Hospital de Clínicas da Universidade Federal do Rio Grande do Sul (UFRS), Porto Alegre, RS – Brazil; 17 Salvador BA Brazil Hospital Universitário Professor Edgard Santos (HUPES), Universidade Federal da Bahia, Salvador, BA – Brazil; 18 Rio de Janeiro RJ Brazil Universidade Federal Fluminense (UFF), Rio de Janeiro, RJ – Brazil; 19 Buenos Aires Argentina Hospital Churruca, Buenos Aires – Argentina; 20 Curitiba PR Brazil Adam Cardiologia, Curitiba, PR – Brazil; 21 Natal RN Brazil Hospital Universitário Onofre Lopes, Universidade Federal do Rio Grande do Norte (UFRN), Natal, RN – Brazil; 22 Aracaju SE Brazil Clínica do Coração, Aracaju, SE – Brazil; 23 Rio de Janeiro RJ Brazil Hospital Quinta D’Or, Rio de Janeiro, RJ – Brazil; 24 Rio de Janeiro RJ Brazil Hospital Universitário Pedro Ernesto, Universidade do Estado do Rio de Janeiro (UERJ), Rio de Janeiro, RJ – Brazil; 25 Goiânia GO Brazil Universidade Federal de Goiás (UFG), Goiânia, GO – Brazil; 26 Recife PE Brazil Pronto Socorro Cardiológico de Pernambuco (PROCAPE), Recife, PE – Brazil; 27 Recife PE Brazil Universidade de Pernambuco (UPE), Recife, PE – Brazil; 28 São Paulo SP Brazil Hospital Sírio Libanês, São Paulo, SP – Brazil; 29 São Paulo SP Brazil Instituto Dante Pazzanese de Cardiologia, São Paulo, SP – Brazil; 30 São Paulo SP Brazil Hospital das Clínicas da Faculdade de Medicina da Universidade de São Paulo (HCFMUSP), São Paulo, SP – Brazil; 31 Salvador BA Brazil Universidade do Estado da Bahia (UNEB), Salvador, BA – Brazil; 32 Rio Branco AC Brazil Clínica Silvestre Santé, Rio Branco, AC – Brazil; 33 São Paulo SP Brazil Fundação Zerbini, Instituto do Coração (Incor), São Paulo, SP – Brazil; 34 Rio de Janeiro RJ Brazil Hospital Samaritano, Rio de Janeiro, RJ – Brazil; 35 Rio de Janeiro RJ Brazil Universidade Federal do Rio de Janeiro (UFRJ), Rio de Janeiro, RJ – Brazil; 36 Rio de Janeiro RJ Brazil Universidade Federal do Estado do Rio de Janeiro (UNIRIO), Rio de Janeiro, RJ – Brazil; 37 Rio de Janeiro RJ Brazil Fonte Imagem, Rio de Janeiro, RJ – Brazil

**Table:** 

Brazilian Guideline Approach to Chest Pain in the Emergency Department – 2025	
The report below lists declarations of interest as reported to the SBC by the experts during the period of the development of these statement, 2024/2025.	
Expert	Type of relationship with industry
Alexandre de Matos Soeiro	Financial declaration B - Research funding under your direct/personal responsibility (directed to the department or institution) from the brazilian or international pharmaceutical, orthosis, prosthesis, equipment and implants industry: - Biomerieux.
André Volschan	Other relationships Participação societária de qualquer natureza e qualquer valor economicamente apreciável de empresas na área de saúde, de ensino ou em empresas concorrentes ou fornecedoras da SBC: - Private Practice Partner.
Bruno Ferraz de Oliveira Gomes	Nothing to be declared
Carisi Anne Polanczyk	Nothing to be declared
Carlos Eduardo Ornelas	Financial declaration A - Economically relevant payments of any kind made to (i) you, (ii) your spouse/partner or any other person living with you, (iii) any legal person in which any of these is either a direct or indirect controlling owner, business partner, shareholder or participant; any payments received for lectures, lessons, training instruction, compensation, fees paid for participation in advisory boards, investigative boards or other committees, etc. from the brazilian or international pharmaceutical, orthosis, prosthesis, equipment and implants industry: - Bayer: Firialta; Novartis: Sybrava; Chiesi: Trimbow. B - Research funding under your direct/personal responsibility (directed to the department or institution) from the brazilian or international pharmaceutical, orthosis, prosthesis, equipment and implants industry: - Novartis: Sybrava; Daiichi Sankyo: bempedoic acid. Other relationships Funding of continuing medical education activities, including travel, accommodation and registration in conferences and courses, from the brazilian or international pharmaceutical, orthosis, prosthesis, equipment and implants industry: - Chiesi: Trimbow.
Carlos Eduardo Rochitte	Financial declaration A - Economically relevant payments of any kind made to (i) you, (ii) your spouse/partner or any other person living with you, (iii) any legal person in which any of these is either a direct or indirect controlling owner, business partner, shareholder or participant; any payments received for lectures, lessons, training instruction, compensation, fees paid for participation in advisory boards, investigative boards or other committees, etc. from the brazilian or international pharmaceutical, orthosis, prosthesis, equipment and implants industry: - Canon: Health Imaging Technology; GE: Cardiovascular resonance and tomography.
Carlos Vicente Serrano Jr.	Nothing to be declared
Cláudio Marcelo Bittencourt das Virgens	Nothing to be declared
Claudio Tinoco Mesquita	Financial declaration A - Economically relevant payments of any kind made to (i) you, (ii) your spouse/partner or any other person living with you, (iii) any legal person in which any of these is either a direct or indirect controlling owner, business partner, shareholder or participant; any payments received for lectures, lessons, training instruction, compensation, fees paid for participation in advisory boards, investigative boards or other committees, etc. from the brazilian or international pharmaceutical, orthosis, prosthesis, equipment and implants industry: B - Research funding under your direct/personal responsibility (directed to the department or institution) from the brazilian or international pharmaceutical, orthosis, prosthesis, equipment and implants industry: - AstraZeneca: amyloidosis; Alnylam: amyloidosis.
Danielli Oliveira da Costa Lino	Financial declaration A - Economically relevant payments of any kind made to (i) you, (ii) your spouse/partner or any other person living with you, (iii) any legal person in which any of these is either a direct or indirect controlling owner, business partner, shareholder or participant; any payments received for lectures, lessons, training instruction, compensation, fees paid for participation in advisory boards, investigative boards or other committees, etc. from the brazilian or international pharmaceutical, orthosis, prosthesis, equipment and implants industry: - Boehringer: Tenecteplase. Other relationships Funding of continuing medical education activities, including travel, accommodation and registration in conferences and courses, from the brazilian or international pharmaceutical, orthosis, prosthesis, equipment and implants industry: - Boehringer: Tenecteplase.
Edgardo Jorge Menendez	Nothing to be declared
Eduardo Leal Adam	Nothing to be declared
Fabio Mastrocola	Financial declaration A - Economically relevant payments of any kind made to (i) you, (ii) your spouse/partner or any other person living with you, (iii) any legal person in which any of these is either a direct or indirect controlling owner, business partner, shareholder or participant; any payments received for lectures, lessons, training instruction, compensation, fees paid for participation in advisory boards, investigative boards or other committees, etc. from the brazilian or international pharmaceutical, orthosis, prosthesis, equipment and implants industry: - Novo Nordisk: semaglutide.
Fábio Serra Silveira	Nothing to be declared
Felipe Souza Maia da Silva	Nothing to be declared
Gilson Soares Feitosa-Filho	Financial declaration B - Research funding under your direct/personal responsibility (directed to the department or institution) from the brazilian or international pharmaceutical, orthosis, prosthesis, equipment and implants industry: - Amgen: Olpasiran; Idorsia: Selatogrel; Anthos: Abelacimabe; Jansen e Bayer: Milvexiana. Other relationships Funding of continuing medical education activities, including travel, accommodation and registration in conferences and courses, from the brazilian or international pharmaceutical, orthosis, prosthesis, equipment and implants industry: - Servier/Trimetazidine: Congress Registration.
Giovanni Possamai Dutra	Nothing to be declared
Hélio Penna Guimarães	Nothing to be declared
Humberto Graner Moreira	Financial declaration A - Economically relevant payments of any kind made to (i) you, (ii) your spouse/partner or any other person living with you, (iii) any legal person in which any of these is either a direct or indirect controlling owner, business partner, shareholder or participant; any payments received for lectures, lessons, training instruction, compensation, fees paid for participation in advisory boards, investigative boards or other committees, etc. from the brazilian or international pharmaceutical, orthosis, prosthesis, equipment and implants industry: - Pfizer: amyloidosis and immunizations; Novo Nordisk: obesity and inflammation; Novartis: dyslipidemia; Daichii-Sankyo: dyslipidemia; Bayer: cardio-oncology. Other relationships Funding of continuing medical education activities, including travel, accommodation and registration in conferences and courses, from the brazilian or international pharmaceutical, orthosis, prosthesis, equipment and implants industry: - Novo Nordisk: obesity.
Isly Maria Lucena de Barros	Nothing to be declared
João Luiz Fernandes Petriz	Financial declaration A - Economically relevant payments of any kind made to (i) you, (ii) your spouse/partner or any other person living with you, (iii) any legal person in which any of these is either a direct or indirect controlling owner, business partner, shareholder or participant; any payments received for lectures, lessons, training instruction, compensation, fees paid for participation in advisory boards, investigative boards or other committees, etc. from the brazilian or international pharmaceutical, orthosis, prosthesis, equipment and implants industry: - Servier: Vastarel.
José Roberto de Oliveira Silva Filho	Financial declaration A - Economically relevant payments of any kind made to (i) you, (ii) your spouse/partner or any other person living with you, (iii) any legal person in which any of these is either a direct or indirect controlling owner, business partner, shareholder or participant; any payments received for lectures, lessons, training instruction, compensation, fees paid for participation in advisory boards, investigative boards or other committees, etc. from the brazilian or international pharmaceutical, orthosis, prosthesis, equipment and implants industry: - Chiesi: Trimbow; GSK: Vaccines; Libbs: ticagrelor, ramipril and candesartan; Biolab: vasopressin. Other relationships Funding of continuing medical education activities, including travel, accommodation and registration in conferences and courses, from the brazilian or international pharmaceutical, orthosis, prosthesis, equipment and implants industry: - Daiichi Sankyo: prasugrel.
Julio Flavio Meirelles Marchini	Nothing to be declared
Louis Nakayama Ohe	Financial declaration A - Economically relevant payments of any kind made to (i) you, (ii) your spouse/partner or any other person living with you, (iii) any legal person in which any of these is either a direct or indirect controlling owner, business partner, shareholder or participant; any payments received for lectures, lessons, training instruction, compensation, fees paid for participation in advisory boards, investigative boards or other committees, etc. from the brazilian or international pharmaceutical, orthosis, prosthesis, equipment and implants industry: - Abbott: Coroflow; BBraun: Sequent Please; Libbs: Artag; Meril: Stent.
Ludhmila Abrahão Hajjar	Nothing to be declared
Maria Camila Lunardi	Nothing to be declared
Mucio Tavares de Oliveira Junior	Financial declaration A - Economically relevant payments of any kind made to (i) you, (ii) your spouse/partner or any other person living with you, (iii) any legal person in which any of these is either a direct or indirect controlling owner, business partner, shareholder or participant; any payments received for lectures, lessons, training instruction, compensation, fees paid for participation in advisory boards, investigative boards or other committees, etc. from the brazilian or international pharmaceutical, orthosis, prosthesis, equipment and implants industry: - Sanofi-Pasteur: Efluelda/influenza; Torrent: Iccor/insuficiência cardíaca; Merck: Concor/insuficiência cardíaca; Biolab: Simdax/insuficiência cardíaca; GSK: Arexvy/vírus sincicial respiratório; Pfizer: Abrysvo/vírus sincicial respiratório; Ache: Edistride/insuficiência cardíaca. B - Research funding under your direct/personal responsibility (directed to the department or institution) from the brazilian or international pharmaceutical, orthosis, prosthesis, equipment and implants industry: - Sanofi-Pasteur: Efluelda/Influenza.
Nivaldo Menezes Filgueiras Filho	Financial declaration A - Economically relevant payments of any kind made to (i) you, (ii) your spouse/partner or any other person living with you, (iii) any legal person in which any of these is either a direct or indirect controlling owner, business partner, shareholder or participant; any payments received for lectures, lessons, training instruction, compensation, fees paid for participation in advisory boards, investigative boards or other committees, etc. from the brazilian or international pharmaceutical, orthosis, prosthesis, equipment and implants industry: - AstraZeneca: Lokelma, Forxiga; Servier: Adhesion/Acertanlo, Acertil; Boehringer: Jardiance. Other relationships Funding of continuing medical education activities, including travel, accommodation and registration in conferences and courses, from the brazilian or international pharmaceutical, orthosis, prosthesis, equipment and implants industry: - Servier: Acertanlo; AstraZeneca: Lokelma.
Odilson Marcos Silvestre	Nothing to be declared
Paolo Blanco Villela	Financial declaration A - Economically relevant payments of any kind made to (i) you, (ii) your spouse/partner or any other person living with you, (iii) any legal person in which any of these is either a direct or indirect controlling owner, business partner, shareholder or participant; any payments received for lectures, lessons, training instruction, compensation, fees paid for participation in advisory boards, investigative boards or other committees, etc. from the brazilian or international pharmaceutical, orthosis, prosthesis, equipment and implants industry: - FQM: Exforge HCT. Other relationships Funding of continuing medical education activities, including travel, accommodation and registration in conferences and courses, from the brazilian or international pharmaceutical, orthosis, prosthesis, equipment and implants industry: - FQM: Flebon.
Paulo Rogério Soares	Financial declaration A - Economically relevant payments of any kind made to (i) you, (ii) your spouse/partner or any other person living with you, (iii) any legal person in which any of these is either a direct or indirect controlling owner, business partner, shareholder or participant; any payments received for lectures, lessons, training instruction, compensation, fees paid for participation in advisory boards, investigative boards or other committees, etc. from the brazilian or international pharmaceutical, orthosis, prosthesis, equipment and implants industry: - Merck: Glifage XR/Concor; Libbs: resident program; AstraZeneca: Forxiga. Other relationships Funding of continuing medical education activities, including travel, accommodation and registration in conferences and courses, from the brazilian or international pharmaceutical, orthosis, prosthesis, equipment and implants industry: - AstraZeneca: European Congress 2024.
Pedro Gabriel Melo de Barros e Silva	Financial declaration B - Funding for research under your direct/personal responsibility (directed to the department or institution) from the pharmaceutical, orthosis, prosthetic, equipment, and implant industries, Brazilian or foreign: - Bayer, Novartis, Idorsia, Roche Diagnostics
Pedro Paulo Nogueres Sampaio	Other relationships Participação em comitês de compras de materiais ou fármacos em instituições de saúde ou funções assemelhadas: - Cardiology Coordinator at Samaritano Hospital.
Remo Holanda de Mendonça Furtado	Financial declaration A - Economically relevant payments of any kind made to (i) you, (ii) your spouse/partner or any other person living with you, (iii) any legal person in which any of these is either a direct or indirect controlling owner, business partner, shareholder or participant; any payments received for lectures, lessons, training instruction, compensation, fees paid for participation in advisory boards, investigative boards or other committees, etc. from the brazilian or international pharmaceutical, orthosis, prosthesis, equipment and implants industry: - AstraZeneca: Forxiga; Bayer: Firialta; Boehringer: Trayenta; Daiichi-Sankyo: Lixiana; Server: Diamicron; Libbs: Artag; Novartis: Sybrava. B - Research funding under your direct/personal responsibility (directed to the department or institution) from the brazilian or international pharmaceutical, orthosis, prosthesis, equipment and implants industry: - Aché: protocol consultancy; Libbs: protocol consultancy; AstraZeneca: Heart failure; Pfizer: atrial fibrillation; Regeneron: atrial fibrillation; Viatris: myocardial infarction; Brainfarma: analgesia; Bayer: prostate cancer; Novartis: Heart failure; Roche: statistical analysis. Other relationships Funding of continuing medical education activities, including travel, accommodation and registration in conferences and courses, from the brazilian or international pharmaceutical, orthosis, prosthesis, equipment and implants industry: - Novartis: Sybrava.
Renato D. Lopes	Financial declaration A - Economically relevant payments of any kind made to (i) you, (ii) your spouse/partner or any other person living with you, (iii) any legal person in which any of these is either a direct or indirect controlling owner, business partner, shareholder or participant; any payments received for lectures, lessons, training instruction, compensation, fees paid for participation in advisory boards, investigative boards or other committees, etc. from the brazilian or international pharmaceutical, orthosis, prosthesis, equipment and implants industry: - Pfizer, Daiichi Sankyo, Novo Nordisk, Bayer, Boehringer Ingelheim, Bristol-Myers Squibb. B - Research funding under your direct/personal responsibility (directed to the department or institution) from the brazilian or international pharmaceutical, orthosis, prosthesis, equipment and implants industry: - Amgen, Bristol-Myers Squibb, GlaxoSmithKline, Medtronic, Pfizer, Sanofi-Aventis. Other relationships Funding of continuing medical education activities, including travel, accommodation and registration in conferences and courses, from the brazilian or international pharmaceutical, orthosis, prosthesis, equipment and implants industry: - Pfizer, Daiichi Sankyo, Novo Nordisk, Novartis.
Renée Sarmento de Oliveira	Financial declaration A - Economically relevant payments of any kind made to (i) you, (ii) your spouse/partner or any other person living with you, (iii) any legal person in which any of these is either a direct or indirect controlling owner, business partner, shareholder or participant; any payments received for lectures, lessons, training instruction, compensation, fees paid for participation in advisory boards, investigative boards or other committees, etc. from the brazilian or international pharmaceutical, orthosis, prosthesis, equipment and implants industry: - Novo Nordisk: semaglutide lecture. Outros relacionamentos Financiamento de atividades de educação médica continuada, incluindo viagens, hospedagens e inscrições para congressos e cursos, provenientes da indústria farmacêutica, de órteses, próteses, equipamentos e implantes, brasileiras ou estrangeiras: - Novo Nordisk: semaglutide (Wegovy).
Ronaldo de Souza Leão Lima	Outros relacionamentos Participação societária de qualquer natureza e qualquer valor economicamente apreciável de empresas na área de saúde, de ensino ou em empresas concorrentes ou fornecedoras da SBC: - Partner at Fonte Imagem.
Sandro Pinelli Felicioni	Nothing to be declared
Sergio Timerman	Nothing to be declared
Tatiana de Carvalho Andreuci Torres Leal	Other relationships Funding of continuing medical education activities, including travel, accommodation and registration in conferences and courses, from the brazilian or international pharmaceutical, orthosis, prosthesis, equipment and implants industry: - Eli Lilly: Mounjaro.
Wilson Mathias Junior	Nothing to be declared

## Table of contents

**1. Introduction** 10
**1.1. Scope of the Problem** 10
**1.2. The Role of Chest Pain Units** 11
**1.3. Objectives of this Guideline** 11
**2. Methods** 12
**2.1. Definitions of Classes of Recommendations and Levels of Evidence** 12
**3. Initial Approach** 12
**3.1. Criteria for Activation of the Chest Pain Pathway** 12
**3.1.1. Concept of First Medical Contact** 12
**3.1.2. Assessment of Possible Causes of Chest Pain** 13
**3.1.3. General Eligibility Criteria for the Chest Pain Pathway** 14
**3.1.4. Specific Criteria for Chest Pain Pathway Activation by Nursing Staff** 14
**3.1.5. Subjective Criteria at the Physician's Discretion** 14
**3.2. What to Do After Activating the Chest Pain Pathway** 15
**3.2.1. When and How to Perform Detailed Assessment of Chest Pain (Differential Diagnosis)** 15
**3.3. Best Practices for Door-to-ECG Time (DTE)** 15
**3.3.1. Importance of ECG Within the First 10 Minutes** 15
**3.3.2. How to Measure DTE** 15
**3.3.3. Recommendations for Obtaining the Ideal Door-to-ECG Time** 16
**3.4. ECG Interpretation** 17
**3.4.1. Role of the ECG in Assessment of the Patient with Chest Pain** 17
**3.4.2. How Sensitive is Single ECG for Assessment of the Patient with Chest Pain? What Additional Benefit is Gained from Serial ECGs?** 17
**3.4.3. ECG Diagnostic Criteria for ACS** 17
**3.4.4. Differential Diagnoses on ECG** 18
**3.4.4.1. Primary and Secondary Repolarization Abnormalities** 18
**3.3.4.2. Other ECG Diagnoses** 20
**3.3.4.3. The Nondiagnostic Electrocardiogram** 21
**3.5. How is the Diagnosis of Acute Coronary Syndrome Defined?** 21
**3.5.1. What about Unstable Angina?** 21
**3.5.2. How to Classify ACS by ECG Findings** 21
**4. Diagnostic Pathway: After the Initial ECG** 22
**4.1. Approach to the Patient Meeting Criteria for Instability** 24
**4.1.1. Routine Echocardiography in the Unstable Patient with Chest Pain** 26
**4.2. Clinical Risk Stratification and Diagnostic Probability Scores** 27
**4.2.1. Clinical Risk Scores for Suspected ACS Cases** 28
**4.2.1.1 Which Patients Should Have Their HEART Score Calculated?** 29
**4.2.2. Clinical Scores for Suspected Acute Aortic Syndromes** 29
**4.2.3. Clinical Scores for Suspected Pulmonary Embolism** 29
**4.2.4. Other Differential Diagnoses** 33
**4.3. Troponin Algorithms** 33
**4.3.1. Use and Interpretation of Biomarkers of Myocardial Injury** 33
**4.3.1.1. What is the Biomarker of Choice for Diagnosis of MI?** 34
**4.3.1.2. How to Interpret the Abnormal Troponin Test** 34
**4.3.1.3. How to Interpret Serial Troponin Tests** 34
**4.3.1.4. Diagnostic Criteria for MI** 35
**4.3.1.5. What is the Time Limit to Rule Out a Diagnosis of MI?** 35
**4.3.1.6. What is the Algorithm of Choice?** 35
**4.3.2. Routing According to Classification in the Algorithm** 35
**4.3.2.1. ACS Ruled out** 36
**4.3.2.2. Admission Confirmed (ACS Ruled in)** 37
**4.3.3. Initial Disposition of Intermediate ("Grey-Zone") Cases (Observation)** 39
**4.3.4. Approach to the Patient with Myocardial Injury and Nonobstructive Coronary Arteries** 42
**4.3.4.1 Investigation of cases of elevated troponin without coronary obstruction (troponin-positive nonobstructive coronary arteries – TINOCA or TpNOCA)** 42
**4.4. Rational Use of Noninvasive Tests** 42
**4.4.1. The Role of Exercise Electrocardiogram Testing** 42
**4.4.2. Role of Echocardiography in Assessment of the Patient with Chest Pain** 45
**4.4.3. Myocardial Perfusion Scintigraphy with Single-Photon Emission Computed Tomography (SPECT) and Positron Emission Tomography (PET-CT)** 47
**4.4.3.1. Indications for Myocardial Perfusion Imaging** 47
**4.4.3.2. Special Considerations in Women with Acute Chest Pain** 48
**4.4.4. Computed Tomography Angiography (CTA)** 48
**4.4.4.1. Triple Rule-out** 50
**4.4.5. Cardiovascular Magnetic Resonance Imaging** 51
**4.4.5.1. Detection of Myocardial Ischemia** 51
**4.4.5.2. Differential Diagnosis of Positive Troponin with Normal Coronary Arteries (TINOCA / MINOCA)** 51
**4.4.5.3. Myocarditis** 52
**5. A Model for Implementation of Chest Pain Units** 53
**5.1. Investigation of Acute Coronary Syndrome in the Prehospital Care Setting** 53
**5.1.1. Patient Perceptions and Organization of the Prehospital Care Network** 53
**5.1.2. Role of ECG in the Prehospital Setting** 54
**5.1.3. Role of Troponin in the Prehospital Setting** 55
**5.1.4. Role of Risk Stratification Scores** 55
**5.1.5. Other Diagnostic Tools** 55
**5.2. A Model for Implementation of Chest Pain Units: Evidence for Best Practice** 55
**5.2.1. Patient Assessment** 55
**5.2.2. Organizational Structure and Human Resources** 56
**5.2.3. Technical Requirements** 56
**5.2.4. Specific Therapies** 57
**5.2.5. Management Indicators** 57
**5.2.6. Technological Advances** 57
**Supplement** 58
**Figure S1** 58
**Figure S2** 59
**Figure S3** 59
**Figure S4** 60
**Figure S5** 60
**Figure S6** 61
**Figure S7** 62
**Figure S8** 62
**References** 63

**Figure S1:** 52-year-old woman seen with resolving angina of 15 minutes’ duration. A) ECG on admission B) ECG at the time of angina recurrence showing horizontal ST-segment depression > 0.5 mm in leads II, III, aVF, and V3 to V6. Diagnosed with NSTE-ACS.

**Figure S2:** 73-year-old man, current smoker with history of dyslipidemia, presenting 30 minutes after onset of burning precordial pain. Note presence of diffuse downsloping ST-segment depression plus ST elevation > 0.5 mm in lead aVR. Administration of sublingual nitrate was followed by improvement of pain and reversal of ST-segment changes. This pattern is associated with LMCA involvement and/or triple-vessel disease. The patient should undergo early coronary angiography.

**Figure S3:** Examples of aortic dissection. A: Note that flow (in blue) follows the true lumen. B: Note aneurysm in the descending aorta. C: visible dissection line. D: dissection and aneurysm in the ascending aorta seen on parasternal long-axis view. ASC AO: ascending aorta; DESC AO: descending aorta.

**Figure S4:** RV pressure overload causing LV dysfunction. Note the enlarged RV pushing the interventricular septum towards the LV.

**Figure S5:** Examples of echocardiographic changes caused by myocardial infarction. A: significant left ventricular enlargement with spontaneous echo contrast; B: severe mitral regurgitation secondary to papillary muscle ischemia; C: post-MI ventricular septal defect. Note left-to-right shunting in red.

**Figure S6:** Massive pericardial effusion. A: fluid surrounding the entire heart seen on parasternal short-axis view. B: fluid surrounding the entire heart viewed through subcostal window.

**Figure S7:** Approach to the patient with chest pain and hemodynamic instability.

**Figure S8:** Exercise myocardial perfusion scan demonstrating reversible myocardial perfusion defects in the right coronary artery territory corresponding to 20% of the myocardium.

## Introduction

### 1.1. Scope of the Problem

Cardiovascular disease (CVD) is the number-one cause of death in Brazil and worldwide, and acute coronary syndrome (ACS) is the leading specific cause of cardiovascular death.^1^ACS represents the spectrum of clinical and laboratory manifestations of acute myocardial ischemia. According to current definitions, it comprises unstable angina (UA), non-ST-segment-elevation myocardial infarction (NSTEMI), and ST-segment-elevation myocardial infarction (STEMI), with each of these diagnoses respectively accounting for 30%, 34%, and 36% of confirmed ACS cases in the Brazilian Registry of Acute Coronary Syndromes (ACCEPT).^2^

In the United States of America (USA), the prevalence of any acute myocardial infarction (MI) is approximately 3% in adults, with coronary heart disease accounting for about 41% of all cardiovascular deaths in 2020.^3 , 4^In Brazil, the prevalence of acute MI was approximately 4% in a cross-sectional study involving 7,260 individuals from different ethnicities and regions.^5^From an economic standpoint, myocardial infarction represents the highest financial burden of heart disease for the Brazilian Unified Health System ( *Sistema Único de Saúde* , SUS), with an expenditure of R$ 22.4 billion (roughly 6.9 billion U.S. dollars) in 2015.^6^Considering that ACS represents not only the leading cause of death in the overall population^7^but also the costliest heart disease for the Brazilian health system, the implementation of methods for early, accurate, and efficient diagnosis and management of these patients is a priority public health concern.

The main symptom of ACS is chest pain. It is estimated that, each year, 5 to 8 million individuals present to emergency departments (EDs) in the United States with chest pain or ischemic-equivalent symptoms suggestive of acute myocardial ischemia.^8^This represents approximately 5% of all U.S. emergency visits, demonstrating the societal burden of chest pain.^8 – 10^In addition to being a frequent complaint in the emergency setting, chest pain can represent up to 40% of the causes of hospital admission through the ED, with a high cost burden for publicly funded health systems.^10 , 11^Although chest pain is the most common symptom leading to recognition of ACS, only 5 to 20% of patients presenting to the ED with chest pain have ACS, and more than half of chest pain cases seen in the ED do not have a cardiac cause.^12 – 14^Rapid, assertive diagnosis and management can significantly improve the prognosis of these patients and reduce costs for the healthcare system.

Due to the high incidence of this complaint in EDs, patients with chest pain often receive care from providers and/or at health facilities that do not specialize in cardiovascular diseases. Appropriate guidance for all facilities and providers is essential for the optimal management of these cases, ensuring that patients receive care safely and that the health system provides care efficiently. Although ACS is the most frequent cause of death in the population with chest pain, there are other potentially fatal and time-sensitive diagnoses that should also be considered in these patients. Therefore, assessment of chest pain in the ED aims at early recognition not only of ACS, but also of such alternate diagnoses that are associated with immediate risk of death, such as aortic dissection and pulmonary embolism.

### 1.2. The Role of Chest Pain Units

In the 1960s, coronary care units (CCU) were established with the primary objective of providing a safe environment for diagnostic confirmation and, especially, treatment of acute MI.^15^The advent of these units has had a substantial clinical impact, reducing acute MI mortality by approximately 50%, particularly through early recognition and effective treatment of arrhythmias and cardiac arrest.^15 , 16^Despite the clinical benefit for confirmed cases of acute MI, the availability of a safer environment for patient management led to a more liberal approach in clinical practice, in which physicians also admitted many unconfirmed cases – i.e., patients with only a clinical suspicion of ACS – to the CCU.^15 , 16^As a consequence of this approach based on heterogeneous clinical judgment rather than objective protocols, more than half of patients admitted to CCUs did not, in fact, have ACS.^16^A large proportion of these high-complexity, high-cost beds thus began to be occupied by patients with low diagnostic probability of ACS and a low risk of complications, resulting not only in saturation of the CCUs but also in misuse of resources.

The absence of protocols that would allow greater assertiveness in selecting which patients should be hospitalized led both to overcrowding of coronary care units and to a high risk of inadvertent discharge of patients from the ED with unrecognized acute MI. Historically, in the U.S., 1 to 10% of patients who were actually having an MI were inappropriately discharged from the emergency room because their diagnosis was not recognized, and this inadvertently discharged group had a higher risk of death.^17 – 20^In settings with fewer resources and/or where doctors are less prepared to care for such cases, the rate of unrecognized MI is even higher.^20^Thus, although ACS represents a relatively small proportion of chest pain cases, in the absence of systematization of care (chest pain pathways), patients become more vulnerable to misdiagnosis (both underdiagnosis and overdiagnosis) despite the availability of resources. This entails a greater risk of fatal complications (when undiagnosed MI cases are inadvertently discharged home) and higher hospital costs (when high-complexity resources, such as CCU beds, are overused for lower-risk patients).

In light of this scenario, Chest Pain Units (CPUs) were created in 1982.^8 , 21 – 23^CPUs do not necessarily represent a dedicated physical space for care of the patient with chest pain; instead, they primarily constitute care pathways and processes that allow for the systematic, best practices-based assessment of patients with a suspected diagnosis of ACS.^24 , 25^To achieve this, CPU doctors and nurses must be trained in and familiar with the management of cardiovascular emergencies.

In addition to their importance in guiding physicians to the correct diagnosis, chest pain pathways enable rapid implementation of evidence-based therapies. Although life-threatening diagnoses usually represent < 10 to 20% of chest pain cases, such situations are time-sensitive; therefore, the earlier the diagnosis, the faster the intervention and, consequently, the more lives will be saved (especially in conditions such as STEMI and acute aortic dissection).

In summary, the protocolized, systematic care of patients with chest pain or suspected ACS aims to:

1. Facilitate and prioritize access to care for patients with chest pain or ischemic-equivalent symptoms who present to the emergency department (initial care); and
2. Provide an organized strategy for the diagnostic and therapeutic management of such patients in the emergency department, aiming for both speed and quality in care to achieve the best possible outcomes for patients while simultaneously focusing on the efficient use of available resources, i.e., delivering value in health (diagnostic pathway and treatment pathway).^25^

### 1.3. Objectives of this Guideline

- Provide practical guidance to physicians (cardiologists and non-cardiologists) and multidisciplinary teams on the initial management of patients with acute-onset chest pain (or ischemic-equivalent symptoms), in accordance with evidence-based best practices;
- Establish a standardized care pathway for the management of patients with suspected ACS (and relevant differential diagnoses), minimizing the time to completion of critical diagnostic and therapeutic procedures to ensure the fastest possible patient recovery;
- Provide guidance on the utilization of healthcare resources, with a view to delivering value in a manner adaptable to each setting of care;
- Provide guidance on how to collect information on care practices to inform assessment of care quality by establishing measurable time targets and goals for the best practices included in the chest pain pathway. This information is essential for identifying gaps in care and assessing the results of ongoing quality improvement efforts.

In summary, there are three main objectives in implementing a chest pain protocol ( Table 1 ):

- Excellence in care process indicators (e.g., metrics for time to diagnosis);
- Improved efficiency of the health system (e.g., metrics assessing appropriate resource allocation);
- Reduction in the rates of adverse outcomes for confirmed cases of potentially life-threatening diseases (e.g., reducing outcomes such as death and ventricular dysfunction in ACS).

**Table 1:** Objectives of chest pain pathways and the corresponding action required to achieve each objective

Objectives	Action
Early identification of patients with ACS (and other time-sensitive conditions)	Electrocardiogram (ECG) within 10 minutes (repeat ECG, serial biomarkers, and order other tests as indicated for differential diagnoses)
Avoid inadvertent discharge and Avoid unnecessary hospitalization	Decision pathways based on best practices
Save lives and Reduce sequelae (e.g., post-MI heart failure)	Early evidence-based treatment (follow disease-specific treatment guidelines, such as ACS guidelines)

ACS: acute coronary syndrome; ECG: electrocardiogram.

## 2. Methods

### 2.1. Definitions of Classes of Recommendations and Levels of Evidence

#### Recommendations

**Class I:** Conditions for which there is conclusive evidence or, failing that, general agreement that a given procedure is safe and useful/effective.

**Class II:** Conditions for which there is conflicting evidence and/or a divergence of opinion about the safety and usefulness/efficacy of a procedure.

**Class IIa:** Weight or evidence/opinion in favor of the procedure. Most approve.

**Class IIb:** Safety and usefulness/efficacy are less well established, with no predominance of opinions in favor.

**Class III:** Conditions for which there is evidence and/or general agreement that a procedure is not useful/effective and, in some cases, may be harmful.

**Evidence**

**Level A:** Data derived from multiple concordant randomized trials and/or robust meta-analysis of randomized clinical trials.

**Level B:** Data derived from less robust meta-analyses, from a single randomized trial or from non-randomized (observational) studies.

**Level C:** Data derived from consensus opinion of experts and/or small non-randomized (observational) studies.

## 3. Initial Approach

### 3.1. Criteria for Activation of the Chest Pain Pathway

#### 3.1.1. Concept of First Medical Contact

The term "first medical contact" (FMC) refers to the first interaction with a healthcare professional or service, ideally occurring in the pre-hospital setting. The initial approach to patients presenting with chest pain is always aimed at ruling in or ruling out a diagnosis of ACS or another potentially fatal condition (e.g., acute aortic dissection). Given the numerous possible differential diagnoses, establishing the correct cause of chest pain remains a challenge for physicians working on the front lines in emergency departments, urgent care facilities, and pre-hospital emergency medical services.

When faced with a patient complaining of chest pain, the ED provider (particularly the first medical professional to have contact with the patient) must decide whether immediate initiation of specific treatment measures is warranted, considering that several acute conditions that may present as chest pain carry an immediate risk of death, including the acute aortic syndromes (dissection, aneurysm, and hematoma), ACS (unstable angina and acute MI), pulmonary embolism (PE), and tension pneumothorax. In the emergency department, this so-called first medical contact (FMC) may be performed by a physician or by another healthcare professional (e.g., triage nurse).

Prompt provision of appropriate care is essential, and algorithm-based emergency triage systems play a key role in this process. One such example is the Manchester triage system, a patient selection protocol based on color-coded levels of urgency/emergency. This method emerged in its namesake city in England in 1997, and quickly gained worldwide acceptance due to its efficiency. In Brazil, the Manchester triage model arrived in earnest around 10 years later, in an attempt to reduce long wait times in hospitals and prevent potential losses of patients who present in more serious condition. The Manchester protocol is based on a five-tier color code which is also used on patients’ ID bracelets, as seen in Figure 1 .^26^Considering that every patient who presents to the ED with chest pain or equivalent symptom of ischemia must receive first medical contact within 10 minutes,^27^when applying the Manchester triage system, all such patients must be assigned a "red" or "orange" priority level in the interest of safety and to ensure they are seen in a timely fashion (in both cases, the patient must receive an ECG within 10 minutes). Studies have demonstrated the ability of nurses using the Manchester triage system to detect high-risk patients with chest pain,^28^the impact of Manchester triage use on short-term mortality of patients with ACS,^29^and the sensitivity and specificity of Manchester triage for patients with ACS,^30^and have assessed whether this triage system was used effectively in patients admitted with a diagnosis of ACS.^31^Adaptations to the Manchester protocol (or equivalent triage systems) can be made in response to continuous assessments of local quality of care and according to the specificities of each service, with the aim of improving performance in triaging these patients.

**Figure 1:** Manchester triage – ID wristbands.

Finally, appropriate activation of the chest pain pathway may prevent inappropriate discharge of patients with ACS and reduce unnecessary hospitalizations and diagnostic tests. The chest pain pathway acts as a guide that provides safety and avoids unnecessary variability; however, clinical assessment is a key element for its optimal application.^32 , 33^

#### 3.1.2. Assessment of Possible Causes of Chest Pain

When assessing a patient with chest pain, it is imperative to consider those causes that require prompt management, not only because of their high potential for lethality but also because of the short time window for intervention. There are no pathognomonic signs of any one such condition, and anchoring on a single diagnosis – even if it is the most prevalent diagnosis – will lead to mismanagement. The present section suggests an initial assessment logic focused on those conditions that require the earliest management.

Thus, the first cause to be considered in terms of severity is aortic dissection. Although the prevalence of aortic dissection is lower (approximately one case for every 20 to 90 cases of acute myocardial infarction),^34 , 35^a diagnosis that is never considered will never be made, and the estimated lethality is about 50% within 48 hours. Moreover, if it is mistaken for ACS, the initial use of antiplatelet or anticoagulant therapy – contraindicated in cases of dissection – will be harmful to patients.^36 , 37^

- Aortic dissection: patients should be assessed for the presence of risk factors for aortic dissection, perfusion deficits (pulse or blood pressure differentials), and a widened mediastinum. Several decision-making algorithms are available, such as the *Aortic Dissection Detection Risk Score* (ADD-RS) and the *Aortic Simplified Score* (AORTA). The ADD-RS included Brazilian patients from the Heart Institute of the University of São Paulo Medical School (InCor-HCFMUSP), improving its applicability to the Brazilian population. In cases of suspected aortic dissection, any antiplatelet or anticoagulant therapy should be deferred until the diagnosis has been confirmed or excluded using the selected algorithm (see section 4.2.2 for further details);
- Acute coronary syndrome (ACS): ACS is the most common potentially fatal cause of chest pain and will be discussed in greater detail in the next section;^38^
- Pulmonary embolism (PE): PE is another cause of chest pain with high lethality potential. PE-related chest pain is typically described as pleuritic (worsens with inspiration) and of sudden onset. Situations that promote immobility – such as orthopedic injuries, treatments, or other postoperative conditions – increase the likelihood of deep vein thrombosis (DVT) with subsequent pulmonary embolization. Clinical prediction rules such as the Wells and Geneva scores can be used to stratify PE risk and guide the diagnostic pathway. If D-dimer testing is indicated, use a value ≥500 ng/mL (μg/L) as the cutoff for a "positive" level, or use an age-adjusted value (age x 10 for patients 50 years and older), which helps with the specificity of the test cutoff (if using the YEARS criteria, the D-dimer cutoff may be 500 or 1000 ng/mL, depending on the presence or absence of YEARS clinical criteria, respectively). 39,40 Furthermore, in patients with an intermediate or high probability of PE and without bleeding, the first dose of full anticoagulation may be indicated (see section 4.2.3 for further details);
- Pneumothorax: simple pneumothorax may present as chest pain and can occur spontaneously in slender (ectomorphic) individuals, usually as a benign condition. Conversely, tension pneumothorax is a life-threatening emergency requiring immediate treatment. It most often occurs in the setting of trauma, with identification and management forming part of the primary trauma survey in thoracic or multiple trauma cases. In the in-hospital setting, tension pneumothorax can occur as a complication of procedures such as endotracheal intubation (due to tracheal perforation) or central venous catheterization;^41^
- Pericardial disease: cardiac tamponade typically manifests as chest discomfort. Patients usually present with hypotension or overt shock. Beck's triad has limited diagnostic utility; the sensitivities of its individual components for cardiac tamponade are low: hypotension (26 ± 10%), muffled heart sounds (28 ± 7%), and elevated jugular venous pressure (76 ± 14%).^42^Another study reported an even lower sensitivity for the triad as a whole.^43^Differentiation from other forms of circulatory shock has been greatly facilitated by bedside point-of-care ultrasound, which also allows ultrasound-guided management;
- Severe esophageal conditions: these may also present with chest pain. Esophageal rupture with mediastinitis is rare, with a reported incidence of 3 per 1,000,000 population.^44^When associated with forceful vomiting – often in alcoholic patients – it is referred to as Boerhaave syndrome. Early assessment and intervention significantly impact prognosis;^45^
- Other diagnoses: after excluding the most urgent conditions, other causes with potential for complications should be considered, particularly in cases of myocardial injury (elevated troponin). An example is *Takotsubo syndrome* (stress cardiomyopathy), which in approximately two-thirds of cases is associated with intense emotional or physical stressors (e.g., sepsis, intracranial hemorrhage). It produces severe left ventricular dysfunction, typically with basal segment sparing (although variants exist). It is often indistinguishable from ACS on presentation, with diagnosis usually made retrospectively. In cases of cardiogenic shock, it is important to recognize that up to one-fifth of patients have concomitant left ventricular outflow tract obstruction (LVOTO), which may worsen with inotropic therapy.^46^

#### 3.1.3. General Eligibility Criteria for the Chest Pain Pathway

All patients presenting from home or transferred from other health facilities who have a history of chest pain (or ischemic equivalent) of acute onset (at rest or originally with exertion) are eligible for the chest pain pathway.

#### 3.1.4. Specific Criteria for Chest Pain Pathway Activation by Nursing Staff

The general criteria outlined above define the population covered by this guideline; however, a protocol that is designed to be activated by different providers (doctors, nurses) with different levels of expertise requires objective and highly sensitive criteria. Although the overarching objective of this type of criteria would be to offer safety through high sensitivity, thus preventing delayed recognition of a significant number of serious cases, on the other hand, overly broad criteria can overload emergency departments by applying to patients with a very low likelihood of deriving benefit. Thus, the criteria for activation of a chest pain pathway must be defined according to the specific objective of the desired early diagnoses and must follow a minimum standard ( Tables 2 and 3 ).

**Table 2:** Routine criteria for chest pain pathway activation

	Class of recommendation	Level of evidence
Any CURRENT pain (at the time of admission) anywhere from the navel to the jaw.	I	C
Any chest pain of more than 10 minutes’ duration (even if absent on admission).	I	C

These routine criteria focus on the recognition of acute coronary syndrome with coronary occlusion, and therefore with potential benefit from reperfusion therapy, as well as other diagnoses carrying a high risk of lethality (e.g., acute aortic dissection).

**Table 3:** Additional criteria for chest pain pathway activation

	Class of recommendation	Level of evidence
Patient asymptomatic on admission, but who reports epigastric discomfort or pain in the arms or jaw before arrival at the emergency department.	IIa	C
Ischemic equivalent (e.g., dyspnea, diaphoresis, and/or SBP < 90 mmHg) in patients over 50 years of age and/or with a history of diabetes or known cardiovascular disease (e.g., prior myocardial infarction, angioplasty, stroke).	IIa	C
Upon request, after medical evaluation, in the absence of the above criteria (clinical suspicion of ACS or other life-threatening conditions such as aortic dissection).	IIa	C

SBP: systolic blood pressure.

* Considering that 10 to 30% of patients with ACS do not present with chest pain (especially older adults or those with comorbidities such as diabetes),^47 , 48^the use of more sensitive criteria that include ischemic equivalents allows for a broader detection of ACS cases. These broader criteria must be accompanied by clinical assessment to ensure adequate use of resources (i.e., staff must always remain vigilant and act to avoid misuse of resources and overcrowding of chest pain units)

#### 3.1.5. Subjective Criteria at the Physician's Discretion

As per the last recommendation listed in Table 3 , in addition to cases presenting with objective criteria that allow rapid identification by the nursing staff, patients who after medical evaluation are suspected of having an ACS mimic or other life-threatening diagnosis (pulmonary embolism, aortic dissection, etc.) may also be enrolled in the chest pain pathway.

Less specific manifestations such as syncope, weakness, confusion/delirium, "indigestion," unexplained nausea, or vomiting may be considered angina equivalents, particularly in high-risk patients (e.g., elderly, patients with diabetes, or those with established cardiovascular disease). In such cases, initiation of a chest pain protocol maybe appropriate even in the absence of objective diagnostic criteria. Because routine ECG evaluation is recommended in patients with syncope, this presentation may serve as a standard objective criterion to perform a 12-lead ECG within 10 minutes of arrival to the emergency department. However, further investigation for ACS beyond the initial ECG is not necessarily required unless additional clinical features are present.

### 3.2. What to Do After Activating the Chest Pain Pathway

Every patient with suspected ACS (i.e., who meets criteria for activation of the chest pain pathway) must be treated as a medical emergency, with immediate referral for an electrocardiogram (ECG), and the medical staff must be notified to promptly assess the patient and review the ECG ( Table 4 ). Ideally, the " MOVE " mnemonic should be followed:

M: Place patient on m onitor (with a defibrillator readily available).
O: Check O_2_saturation and provide supplemental o xygen if < 90%.
V: V enipuncture (draw blood for laboratory tests while obtaining peripheral venous access).
E: E lectrocardiogram within 10 minutes of arrival, with immediate review by a physician.

**Table 4:** Initial Diagnostic Approach after Activation of the Chest Pain Pathway

	Class of recommendation	Level of evidence
In patients who meet criteria for the chest pain pathway, a 12-lead ECG should be performed (ideally with addition of supplemental leads) and reviewed by a physician within 10 minutes (total time since first contact with a healthcare professional/service).	I	B

* For patients in the chest pain pathway, ECG should ideally be performed with the patient in a monitored bed (especially if symptoms persist) or, at least, with a defibrillator readily available nearby.

#### 3.2.1. When and How to Perform Detailed Assessment of Chest Pain (Differential Diagnosis)

Considering that the initial patient assessment usually takes place in the nurse-led triage area of the ED and that there is a class I recommendation for routine acquisition of an ECG within 10 minutes in patients presenting with chest pain or chest pain equivalents, every patient who meets criteria for activation of the chest pain pathway must immediately undergo an ECG and, simultaneously (in parallel), a physician must be immediately alerted of their arrival. As this rule applies to both "typical" and "atypical" chest pain (now-deprecated terms), the priority is to obtain an ECG as quickly as possible. In-depth assessment of chest pain characteristics (and/or chest pain-equivalent symptoms) should be done in parallel; it should never delay the ECG.

### 3.3. Best Practices for Door-to-ECG Time (DTE)

#### 3.3.1. Importance of ECG Within the First 10 Minutes

Resting 12-lead ECG is the first-line diagnostic modality of choice for the evaluation of patients with suspected ACS. It should be performed within 10 minutes of the patient's ED arrival – or ideally prehospital, upon first contact with the emergency medical services (EMS) – and immediately reviewed by a qualified physician.^49 , 50^This means the ECG may precede even such essential steps of clinical assessment as a detailed history or a complete physical examination.^51^

An analysis of over 7,500 patients from a registry in the United States and Canada demonstrated that only 40% of patients with STEMI received an ECG within 10 minutes (median time 14 minutes) and that a DTE of > 10 minutes was associated with an increased risk of recurrent MI or death (odds ratio [OR] 3.95, 95% CI 1.06–14.72, p = 0.04).^52^

The rationale for this approach is the evidence that, in the presence of ST-segment elevation of ischemic origin (or an equivalent change), early ECG acquisition will prompt rapid myocardial reperfusion therapy and, consequently, have a positive impact on morbidity and mortality. Indeed, increased DTE is associated with increased door-to-balloon time for percutaneous coronary intervention^53^and increased door-to-needle time for thrombolysis.^54^Furthermore, the ECG performed in the prehospital setting can help reduce the time to diagnosis and allow appropriate referral of patients with STEMI to centers capable of performing early primary angioplasty, which can receive the ECG before ambulance arrival and prepare to send the patient directly to the catheterization laboratory without a "stopover" in the emergency department.^55^

#### 3.3.2. How to Measure DTE

The most important aspect in deciding which parameters to use for DTE measurement is to choose objective metrics which are readily assessable and reliable in the facility. Although "door" time most properly refers to the patient's entry into the emergency department, this parameter is not always easily assessable enough to serve as a quality indicator. The following are recommended as definitions for benchmarking:

Door time: One widely used method is to use the time of "first medical contact" (FMC), i.e., the patient's first contact with a healthcare provider, which usually occurs in ED triage, as the "door" time. Use of this parameter allows comparison with other services that follow this internationally adopted standard.^56^Its usefulness is, however, limited when delays occur between the time the patient arrives at the ED and triage is actually performed; therefore, even if the time to FMC is adopted as the "door" time for purposes of external benchmarking, it is essential that pre-triage time be monitored as well (even if only for internal improvement purposes).

ECG time: Although theoretically the time of ECG analysis (reporting*) is the optimal "ECG time," this parameter is not always available. Therefore, the time recorded on the ECG strip can be considered instead, as long as it is accurate (both the nursing staff and clinical engineering should periodically check that the device is keeping good time) and that it is routine practice at the facility for ECGs to be given immediately to a doctor for review (there is no benefit to the patient if an ECG is performed within the first 10 minutes of arrival but is not reviewed immediately).

#### 3.3.3. Recommendations for Obtaining the Ideal Door-to-ECG Time

When evaluating DTE, the recommended first step is to assess the current time for each stage – from arrival until an ECG is performed – at the facility, to allow mapping of local processes and practices. This will allow for a greater understanding of which interventions can have the greatest impact on the processes identified. Ongoing feedback should also be provided to the entire team involved, including the ED physician and nursing staff.^54 – 58^

A strategy commonly reported in successful implementations involves tailoring the triage space to allow for a dedicated ECG and skilled technician.^57 – 65^Sprockel *et al.* evaluated 373 patients, 204 in the pre-intervention phase and 169 in the post-intervention phase. The median time to ECG was 16 minutes in the pre-intervention phase (< 10 minutes in 41% of cases); after making a dedicated ECG device available in the triage area (with a dedicated technician), the median time to ECG fell to 5 minutes (< 10 minutes in 63% of cases), a statistically significant difference.^61^

Another commonly recommended strategy is to train triage staff both to prioritize performing an ECG in cases meeting criteria for activation of the protocol and to improve recognition of signs and symptoms of ACS beyond typical chest pain, including ischemic equivalents (e.g., epigastric pain).^60 , 62 , 63^

Phelan *et al.* initially identified two main causes of DTE > 10 minutes at their institution: (1) priority delay (e.g., completing triage and registration data entry tasks before ECG) and (2) failure to recognize patients with ACS symptoms other than chest pain. After interventions that included a patient prioritization process for triage, assignment of staff to perform ECGs immediately, continuous feedback, and an educational initiative for triage staff to identify high-risk patients, the mean time to ECG was significantly reduced from 21.28 ± 5.49 minutes to 9.47 ± 2.48 minutes (p < 0.033), representing a 55% improvement.^64^

A Brazilian study aimed at identifying issues with DTE logistics detected three major problems: 1) delay between arrival at the hospital and first medical contact (solved by a full-time triage protocol); 2) in-hospital communication and lack of prioritization (improved by a standardized code); 3) diagnostic delay (expedited by the presence of a cardiologist in the ED).^65^

There are also data suggesting that sex, race, language fluency, diabetes, and type of symptoms are associated with delays in the diagnosis of STEMI. In a 3-year retrospective cohort study of 676 patients across 10 U.S. hospitals, a door-to-ECG time > 10 minutes versus ≤ 10 minutes was more likely to occur in women (32.8% vs. 22.6%, p = 0.005), Black patients (23.4% vs. 12.4%, p = 0.005), patients with limited English proficiency (24.6% vs. 19.5%, p = 0.032), patients with diabetes (40.2% vs. 30.2%, p = 0.010), and those less likely to report chest pain (63.3% vs. 87.4%, p < 0.001).^66^

In summary, there is no single specific strategy for reducing door-to-ECG time, but rather a combination of actions that may be adapted to individual EDs ( Table 5 ). Most studies used various interventions implemented simultaneously, which makes it difficult to identify one specific optimal strategy in isolation. Therefore, it is essential to map and understand the local context and identify barriers so that an efficient bundle of interventions with the overarching goal of reducing DTE can then be implemented ( Table 6 ).

**Table 5:** Key interventions to reduce DTE

1. Process mapping to identify bottlenecks
2. Dedicated ECG (and a trained ECG technician) in triage
3. Training of triage staff to recognize suspicious symptoms
4. Improved organization of triage flow (reduce bureaucracy and prioritize ECG for patients in whom it is indicated)
5. Constant feedback to the DTE team

DTE: door-to-ECG Time; ECG: electrocardiogram; ACS: acute coronary syndrome.

**Table 6:** Door-to-ECG time

	Class of recommendation	Level of evidence
Emergency departments must monitor their door-to-electrocardiogram time, provide continuous feedback to staff charged with each stage of the initial approach to patients with chest pain, and implement actions for improvement in accordance with identified opportunities.	I	B

### 3.4. ECG Interpretation

History taking and physical examination may occur concomitantly with acquisition and interpretation of the ECG. It bears stressing that STEMI can be caused by type A aortic dissection with involvement of the coronary arteries. Therefore, even when the ECG is obtained immediately (within the first 10 minutes), clinical assessment must proceed in parallel, and even in cases meeting unequivocal ECG criteria for STEMI, the clinician must also be alert to other, potentially co-occurring diagnoses.^67^

#### 3.4.1. Role of the ECG in Assessment of the Patient with Chest Pain

The ECG is a widely available, low-cost diagnostic tool that is easy to interpret locally – and even remotely – enabling its use in various healthcare systems.^68^Performing an ECG is a fundamental step in the diagnostic evaluation of patients with chest pain (and ischemic-equivalent symptoms) in different settings (emergency department, pre-hospital care). Based on its findings, it is possible to promptly identify patients who will benefit from reperfusion therapy, determine the culprit coronary artery and the site of obstruction, stratify risk, and decide on the most appropriate initial therapy for each case.^69^

#### 3.4.2. How Sensitive is Single ECG for Assessment of the Patient with Chest Pain? What Additional Benefit is Gained from Serial ECGs?

Although electrocardiography is essential for the diagnosis and treatment of ACS, a single 12-lead ECG performed upon first medical contact in a patient with chest pain has low sensitivity, detecting only < 50% of acute MIs (including STEMI and NSTEMI).^70 , 71^

Acute myocardial ischemia is often associated with dynamic ECG changes. Serial electrocardiograms can provide relevant information on these changes, significantly increasing sensitivity to 70% to 90%.^72^Some conditions, such as circumflex artery occlusion, may present without significant changes on the 12-lead ECG; therefore, the addition of leads V7, V8, and V9 is indicated to detect "posterior" wall infarction.^73^(Posterior MI – a now-deprecated term, as cardiac magnetic resonance studies have shown it is actually a portion of the lateral wall which is affected^74^– should always be suspected, especially in patients with persistent chest pain suggestive of myocardial ischemia.) These patients – with persistent symptoms and a nondiagnostic initial ECG – are also those in whom more frequent repetition of ECG (every 10 to 20 minutes) is recommended, with electrode placement unchanged, while symptoms last or until the diagnosis is established (at least 2 to 4 serial ECGs are usually required); alternatively, computer-assisted continuous 12-lead ECG recording (if available) can be used to detect dynamic ECG changes.^75^

#### 3.4.3. ECG Diagnostic Criteria for ACS

From a practical standpoint, there are three points to remember:

1. A normal ECG (or nonspecific ECG changes) cannot rule out ACS;
2. Serial electrocardiograms increases accuracy for the diagnosis of ACS;
3. ECG changes suggestive of acute ischemia occur mainly in the ST segment ( Figure 2 ) and/or T waves ( Figure 3 ), ST-segment changes should be measured at the J point, using the PR segment as the baseline reference.^19 , 21^

**Figure 2:** Representation of ST-segment changes. A- Normal ST segment, B- horizontal ST-segment depression, and C- downsloping ST-segment depression, described in ECG reports as subendocardial "current of injury"; D- ST-segment elevation and the proper way to measure it.

**Figure 3:** Representation of T-wave changes. A) Normal T wave. Note asymmetry with slow rise and faster descent. B) Ischemic T wave. Note symmetry, inversion, and increased amplitude (subepicardial ischemia or post-transmural ischemia). C) So-called hyperacute T waves (magnitude and symmetrical base, representing subendocardial ischemia) usually precede the onset of ST-segment elevation. The magnitude represents the area (i.e., a broad-based T wave, which usually also presents with greater amplitude).

The ECG criteria recommended by this guideline for the diagnosis of ACS allow not only the diagnosis of STEACS and NSTE-ACS but also the definition of coronary occlusion and stratification of patient risk ( Table 7 ). Thus, in addition to the classic objective criteria for ST elevation, some specific changes have been identified as "equivalent" to ST-elevation myocardial infarction (STEMI) and may indicate a high probability of coronary artery occlusion even in the absence of classic criteria for *ACS with persistent ST elevation* . This comprehensive assessment provides a better basis for decision-making.^76 – 80^Details on the evidence base and rationale for classification of major ECG patterns are given in a separate article written by the authors of this guideline.^78^

**Table 7:** ECG changes diagnostic of ACS (when associated with a compatible clinical picture):^56 , 77 – 80^

STEMI without left bundle branch block (LBBB)
New ST-segment elevation (measured at the J point) in at least 2 contiguous leads with the following cutoff points: ≥ 2.5 mm in men < 40 years old in V2 and V3; ≥ 2 mm in men ≥ 40 years old in V2 and V3; ≥ 1.5 mm in women in V2 and V3; ≥ 1 mm in other leads (regardless of age or sex)* *If supplementary leads are added (V7, V8, V9, V3R, V4R), elevation ≥ 0.5 mm in at least 2 contiguous leads can be considered for diagnostic purposes (borderline elevations in leads I and aVL should be considered significant, especially if reciprocal ST depression is present in the inferior leads).
**STEMI in a patient with prior LBBB**
• Concordant ST elevation ≥ 1 mm in leads with a positive QRS complex; • Concordant ST depression ≥ 1 mm in V1-V3 (Sgarbossa); • Discordant ST elevation ≥ 5 mm in leads with a negative QRS complex and/or ST/S ratio with ST-segment deviation amplitude of at least 25% of the previous S wave** *New LBBB alone is no longer necessarily considered a diagnostic criterion for STEMI, although it denotes higher risk (clinical correlation required). **Also consider the discordant ST/R ratio when ST depression is at least 25% of the preceding R wave. In the Barcelona criteria, discordant deviation ≥ 1 mm in any lead with maximum voltage (R or S) ≤ 6 mm can be considered for diagnostic purposes
**Findings consistent with coronary occlusion, i.e., STEMI equivalents**
• "Posterior" (inferolateral) infarction: - Criteria to be assessed in leads V1–V3 (usually one or more of the criteria below are present): ST depression; Upright (terminal) T waves in the anterior leads; Prominent and broad R wave (>30 ms), usually dominant (R>S) in V2. - Confirmed by: ST elevation ≥ 0.5 mm in at least 1 lead of V7-V9
• De Winter pattern: - Tall, prominent, symmetrical T waves preceded from upsloping ST depression >1 mm at the J point in the precordial leads; - ST elevation 0.5–1 mm may be seen in aVR
Hyperacute T waves: Broad, symmetric, tall (hyperacute) T waves may be observed early in STEMI. Serial electrocardiograms performed at very short intervals are useful to confirm progression to the classic ST elevation criteria.
Aslanger pattern: ST elevation in lead III but not in the other inferior leads (ST elevation may be present in aVR, as well as in lead III), ST depression in any of V4-V6 with a positive (or at least terminally positive) T wave, and ST elevation in V1 > V2.
Terminal QRS distortion (TQRSD): Absence of an S wave below the isoelectric line and absence of a J wave in either of V2 and/or V3.
Acute coronary syndrome without persistent ST-segment elevation (NSTE-ACS) with high-risk ECG criteria (left main coronary artery or multivessel disease*)
ST-segment elevation in aVR and/or V1 (no contiguous elevation, but ST depression in 6 or more leads) * *Most often caused by diffuse (circumferential) subendocardial ischemia; usually occurs in the setting of significant left main coronary artery obstruction or multivessel coronary artery disease.*
**Acute coronary syndrome without persistent ST-segment elevation (NSTE-ACS) with high-risk ECG criteria (proximal left anterior descending artery occlusion)**
• Wellens syndrome: A clinical syndrome characterized by: - Biphasic T waves (type A – 25% of cases) or deeply inverted, symmetrical T waves (type B – 75% of cases) in leads V2 and V3 (may extend from V1 to V6). - Absence of Q waves. - No significant ST elevation (isoelectric or elevation less than 1 mm) - R-wave progression in the precordial leads - Recent angina (typical ECG changes are more often seen after pain relief)
**Acute coronary syndrome without persistent ST-segment elevation (NSTE-ACS) with high-risk ECG criteria (active ischemia)**
ST depression/T-wave inversion:* - Horizontal or downsloping ST depression ≥ 0.5 mm at the J point in 2 or more contiguous leads, suggestive of myocardial ischemia; and/or - Inverted T wave ≥ 1 mm in complex with prominent R wave or R/S ratio >1 may indicate ischemia in the absence of secondary causes for abnormal ventricular repolarization (e.g., overload); in cases of acute myocardial infarction, the negative T wave may be associated with pathological Q waves. *ST depression and T-wave inversion have even greater diagnostic value when there are dynamic changes. If a negative T wave becomes positive, this finding is called pseudonormalization, and is also an important marker for the diagnosis of NSTE-ACS.

Notes: Cases of transient ST elevation (e.g., vasospasm) require a different approach compared to cases with persistent ST elevation (actual STEMI).

It bears stressing that inverted T waves and ST depression should only be considered diagnostic of NSTE-ACS in the absence of other explanations, including the absence of STEMI criteria (remember that "mirror-image" reciprocal ST depressions are common in STEMI). The presence, extent, persistence, and magnitude of ST depression on admission have major prognostic value in determining whether early invasive treatment is warranted.^77 – 80^

In addition to diagnostic and nondiagnostic ECGs, this guideline includes a third category, "concerning" (i.e., equivocal/borderline) ECG, to place special emphasis on this common driver of errors in decision-making. The objective of creating this category is to reduce mismanagement of time-sensitive situations and add a measure of safety by recommending specific measures for such challenging cases ( Table 8 ).

**Table 8:** Recommended routine measures for the patient with equivocal/borderline ECG

1) The first step when there is any doubt regarding an ECG should be to immediately request review by one or several more experienced physicians. This first step will determine whether the ECG is in fact borderline or whether it only appeared equivocal due to the limited experience of the interpreting physician;
2) When a previous ECG is available, it should be compared to the acute-phase ECG; this can help identify new changes, as long as it does not delay treatment initiation (this is especially important in patients whose baseline ECG already exhibited repolarization abnormalities);
3) Proper clinical correlation is also essential, as the predictive value of any test (including ECG) depends on its clinical pretest probability;
4) Finally, confirmed cases with a borderline ECG (after validation by an experienced physician) and/or clinical instability (e.g., persistent symptoms) require not only continued patient monitoring but also serial electrocardiograms and prompt evaluation for other differential diagnoses. Bedside echocardiography is often an especially useful additional test in these situations.

#### 3.4.4. Differential Diagnoses on ECG

##### 3.4.4.1. Primary and Secondary Repolarization Abnormalities

The morphology of ventricular repolarization, which is represented by the ST segment, T wave, and – occasionally – the U wave, depends directly on ventricular depolarization and should always be interpreted in the morphological context of the preceding QRS complex. Changes due to ischemia (or other forms of intrinsic myocardial damage) are called primary repolarization abnormalities, whereas those preceded by derangements of depolarization, such as bundle branch blocks, ventricular pre-excitation, ventricular pacing, and left ventricular hypertrophy, are called secondary repolarization abnormalities.^13 , 81^RBBB does not hinder the diagnosis of STEMI as LBBB does, but the clinician must be cautious in diagnosing ST segment depression, as the presence of ST depression in V1–V4 (a secondary repolarization abnormality) is common in RBBB.

The "standard" ECG criteria for diagnosis of coronary ischemia refer to electrocardiograms with a narrow QRS complex, i.e., no ventricular conduction disturbances.^77 , 82^In addition to the magnitude of the elevation itself and the extent of the affected territory (presence of contiguous leads), cases of ST elevation resulting from a so-called current of injury (representing transmural ischemia/infarction) usually have the following characteristics:

1. Convex pattern (although it may be concave initially);
2. Progressive dynamic changes on serial ECGs (including development of a pathological Q wave);
3. "Mirror image," i.e., reciprocal ventricular repolarization changes in opposite leads.

Even if ECG criteria are met, clinical correlation is required (ACS symptoms). The most significant cause of ST elevation in this scenario is acute MI, which must be promptly recognized, as the faster treatment is instituted, the lower the morbidity and mortality. However, the clinician must be aware of the many other conditions that may present with ST-segment elevation^83^( Table 9 ) and, when trying to distinguish them ( Table 10 ), it is essential to analyze their ECG features within the clinical context of the patient with chest pain.

**Table 9:** Causes of ST-segment elevation

Leading causes of ST elevation
- Acute myocardial infarction
- Left bundle branch block
- Pericarditis
- Left ventricular hypertrophy
- Early repolarization
- Vasospastic angina (elevation is usually transient)
- Hyperkalemia
- Takotsubo syndrome
- Pacemaker rhythm
- Brugada syndrome
- Ventricular pre-excitation
- Left ventricular aneurysm

**Table 10:** Differential diagnosis between acute MI, pericarditis, and early repolarization

ECG	STEMI	Acute pericarditis	Early repolarization
ST segment morphology	Upwardly convex (may be concave in the early stages, but progresses if left untreated)	Upwardly concave	Upwardly concave
Mirror image (reciprocal ST depression in leads opposite those showing elevation)	Present in most cases	Absent	Absent
Pathological Q waves	Present in most cases	Absent	Absent
Localization of ST elevation	Wall involved in MI	Diffuse/widespread (usually spares V1 and aVR)	Most commonly in precordial leads
PR segment depression	Usually absent	Present	Absent
T-wave inversion	Occurs while ST elevation is present	Occurs after normalization of ST changes	T waves unchanged at rest
ST/T wave ratio (to differentiate between early repolarization and pericarditis)	Not applicable	≥ 0.25	< 0.25 (T wave usually has greater amplitude)

ECG: electrocardiogram; STEMI: ST-segment-elevation myocardial infarction. Although the ECG in pericarditis as a rule does not localize to any coronary artery territories and exhibits characteristic features (concave ST elevation/T-wave changes), there are exceptional cases in which pericarditis (or myopericarditis) can be localized.

##### 3.3.4.2. Other ECG Diagnoses

In addition to its essential role in the diagnosis of ACS, the ECG can provide relevant information for the diagnosis of other major causes of chest pain:

- **Acute pulmonary embolism** : sinus tachycardia is the most common ECG change. The classic S1Q3T3 pattern (wide S wave in DI, inverted Q and T wave in D3) is absent in most cases (seen in only 8.5% of PE cases presenting to an ED).^84^Other ECG changes seen in PE include ventricular repolarization abnormalities, complete or incomplete RBBB, qR in V1 and T-wave inversion in V1-V4 (associated with more severe PE), and atrial tachyarrhythmias;^85^
- **Takotsubo syndrome or stress cardiomyopathy** : a major differential diagnosis of ACS, especially in postmenopausal women experiencing chest pain after emotional and/or physical stress. Patients may present with marked ECG changes, such as ST elevation (44%) and T-wave inversion (41%); QT interval prolongation is likewise common. ST depression (8%) and LBBB (5%) are much less frequent.^86^

Other ECG abnormalities such as bradyarrhythmias or tachyarrhythmias may be present in cases of myocardial ischemia; however, they are nonspecific. Therefore, although an electrocardiographic diagnosis (arrhythmia) can be established in these cases, this should not prevent further investigation of ACS if there is clinical suspicion, as both can occur concomitantly (ischemia may lead to arrhythmia, and the reverse is also true). From the point of view of the ACS workup, this type of finding alone on ECG is considered nondiagnostic (although brady- or tachyarrhythmias should be managed accordingly when present).^87^

##### 3.3.4.3. The Nondiagnostic Electrocardiogram

In less than 5% of patients on a chest pain pathway, the ECG exhibits unequivocal diagnostic criteria; in these cases, if the patient's clinical picture is consistent, a specific diagnosis should be established (e.g., STEMI) and follow the corresponding treatment pathway in accordance with the specific guideline.^88 , 89^However, in more than 90% of cases (especially in patients whose symptoms have already resolved by the time of admission), the ECG proves "nondiagnostic" of acute ischemia. This gives the physician some peace of mind to carry out a more detailed clinical workup and better define the patient's condition and possible differential diagnoses (including through subsequent reassessment at various time points).

Serial ECGs are mandatory when there is recurrence of precordial pain or any change in the clinical status (immediate repetition); in addition, in cases with persistent symptoms, the ECG should be repeated every 10 to 20 minutes. Dynamic ST-segment and T-wave changes are extremely valuable in identifying high-risk patients with myocardial ischemia and should be actively sought.^72 , 87^ Figures S1 and S2(supplement) illustrate dynamic ST-segment changes as evidence of intermittent subendocardial ishemia/current of injury, while Figure 4 presents a flowchart for patient classification according to the ECG findings.

**Figure 4:** Initial classification of ECG in the chest pain pathway. POCUS: point-of-care ultrasound; ECHO: Echocardiogram. ECG: electrocardiogram; STEMI: ST-elevation myocardial infarction; NSTE-ACS: Non-ST Elevation Acute Coronary Syndrome; PE: pulmonary embolism.^1^See Table 7 .^2^In case of conditions that may occur concomitantly with ACS (e.g., arrhythmias), further investigation of ACS should be considered (e.g., myocardial ischemia may cause arrhythmias, and an arrhythmia may lead to myocardial ischemia due to a supply/demand imbalance).^3^Repeat ECG (include supplemental leads) if: 1) Borderline/equivocal ECG and/or persistent symptoms (repeat ECG every 10-20 minutes); 2) Recurrence of symptoms or clinical deterioration (repeat immediately upon recurrence/deterioration); 3) Positive troponin (above 99th percentile); 4) Persistent clinical suspicion despite negative troponin (e.g., diagnostic hypothesis of unstable angina).^4^For clinical assessment, in addition to the clinician's subjective judgment, patient-centered algorithms should be used as appropriate for the suspected diagnosis(es) (e.g., ACS, PE, aortic dissection).

### 3.5. How is the Diagnosis of Acute Coronary Syndrome Defined?

As a rule, the diagnosis of ACS is based on the following elements:

1. Clinical presentation;
2. Electrocardiogram (ECG) findings;
3. Biomarkers of myocardial injury ("cardiac markers"), namely troponin.

Overall, when two of these elements are "positive" or present (e.g., consistent clinical picture + ECG with well-established diagnostic patterns), a definitive diagnosis of ACS can be made.

#### 3.5.1. Current Criteria for the Diagnosis of Unstable Angina

The advent of high-sensitivity troponin has led to a marked reduction in the diagnosis of unstable angina and in the severity profile of these patients. However, cases presenting with a "typical" clinical picture (e.g., ischemic cardiac pain) in the absence of ECG and troponin changes, and for which no alternative diagnosis is established, can be classified as unstable angina and managed initially as ACS –although definitive confirmation will usually depend on other findings, such as those from noninvasive testing or coronary angiography.

In addition to angina at rest, symptoms on exertion with increasing frequency, duration, or severity are also consistent with unstable angina and should raise suspicion of instability of coronary lesions, as should changes in anginal radiation pattern and changes in the response to nitrates. Post-infarction angina and new-onset angina (usually Canadian Cardiovascular Society class III within a period of less than 2 months) are also manifestations of unstable angina.

#### 3.5.2. How to Classify ACS by ECG Findings

Whatever the suspected clinical manifestation (i.e., the criterion for activation of the chest pain pathway), ECG is always the first diagnostic investigation to be performed. Considering that the ECG performed within the first 10 minutes determines subsequent management, it is important that this initial ECG tracing be classified beyond merely checking it for ST elevation criteria ( Tables 7 to 10 ). Thus, although the criteria for STEMI should still be considered in decision-making, the current guideline recommends that the ECG be examined for other changes that may not define a diagnosis of ACS, but are still indicative of acute coronary occlusion even in the absence of the classic findings of ST elevation ( Table 11 ).

**Table 11:** Electrocardiogram classification

	Class of recommendation	Level of evidence
Patients with a suspicious clinical presentation and diagnostic changes on the ECG should follow specific treatment guidelines as appropriate for the established diagnosis.	I	A
In addition to established ST elevation criteria, "STEMI equivalents" (or acute coronary occlusion equivalents) should also be sought.	I	B
Cases in which the electrocardiogram does NOT allow a diagnosis to be established definitively ("nondiagnostic" ECG) should follow the diagnostic pathway as efficiently as possible (as available resources allow).	I	B
Patients on the diagnostic pathway with a borderline/equivocal or difficult-tointerpret ("concerning") ECG and/or cases that meet clinical criteria for instability (Table 12) should undergo repeat ECG every 15 to 30 minutes (with supplemental leads and review by an experienced second physician), as well as active investigation until the cause of chest pain is ascertained.	I	C

ECG: electrocardiogram; STEMI: ST-elevation Myocardial Infarction.

Figure 5 summarizes the initial approach to the patient with chest pain (Step 1 of the Central Illustration ).

**Figure 5:** Step 1: initial care in the chest pain protocol (first 10 minutes).

**Figure:** ECG: electrocardiogram; POCUS: point-of-care ultrasound; MI: Myocardial Infarction.

## 4. Diagnostic Pathway: After the Initial ECG

The diagnostic pathway consists of up to 4 steps ( Central Illustration ). The first step is part of the initial approach to the patient with chest pain and consists of initial clinical assessment + ECG performed within the first10 minutes, which can establish the diagnosis in a minority of cases ( Figures 4 and 5 ) and rapidly direct these cases to a treatment pathway appropriate for the established diagnosis (e.g., STEMI). When the ECG is nondiagnostic (the majority of cases), the patient will be placed on the diagnostic pathway, the elements of which (including algorithms) will guide subsequent decision-making in the emergency department (Step 3 of the Central Illustration ). A summary of the 4 essential steps of the chest pain pathway is given below.

- Step 1 (initial assessment): Overall clinical assessment (signs and symptoms) + ECG within the first 10 minutes to determine if diagnostic criteria are present (e.g.: ACS, pericarditis) and to direct subsequent investigation when the initial assessment is nondiagnostic;
- Step 2 (define initial pretest risk/probability): The main objective at this step is to determine whether the patient has persistent symptoms and/or a "concerning" ECG and/or hemodynamic instability, since these cases require a differentiated approach, including repeat ECG every 10 to 20 minutes and bedside imaging (consider point-of-care ultrasound [POCUS] and/or emergency echocardiography). In situations where a nondiagnostic ECG and combined clinical assessment are not sufficient to safely rule out acute syndromes (e.g., NSTE-ACS, PE, acute aortic syndrome [AAS]), further active investigation should be undertaken (Step 3), with risk assessment and estimation of the pretest probability of the main diagnostic hypotheses, ideally using clinical scores;
- Step 3 (complementary tests according to diagnostic hypothesis and pretest probability): In addition to risk assessment and estimation of diagnostic probability—both of which are established from the initial evaluation and may change over time—this third step involves ordering complementary tests according to the diagnostic hypothesis (ACS, PE, AAS). Pretest probability will determine the most appropriate type of test to perform and how to interpret it (e.g., if PE is suspected but pretest probability is low, D-dimer measurement would be the recommended test to rule out the diagnosis). Cardiac troponin (preferably high-sensitivity) is the most important test for investigating ACS when clinical assessment and ECG are nondiagnostic; even when it does not establish the diagnosis, it can still support decision-making within diagnostic algorithms;
- Step 4 (anatomical and/or functional investigation): Step 4 is required only in a minority of cases, those in which differential diagnoses have been ruled out by the first 3 steps of the pathway but uncertainty still persists regarding a diagnosis of ACS and/or the patient's individual risk is not low enough to allow discharge (e.g., high-sensitivity cardiac troponin (hs-cTn) algorithm with an intermediate value. In these cases, additional testing can be performed before the patient is discharged home, or may be scheduled as part of early outpatient follow-up (see sections 4.3-4.4 and Figures 13 and 14 ).

Therefore, the diagnostic pathway is necessary for cases in which the initial assessment (clinical + ECG) was unable to elucidate the cause of the patient's symptoms. This pathway begins with a detailed clinical assessment. The first concern should be to define whether the patient meets criteria for instability, since, in unstable cases, the diagnostic pathway must be expedited and must include bedside imaging in addition to frequent serial ECGs ( Figure 6 summarizes Step 2 of the Central Illustration ).

**Figure 6:** Step 2: initial assessment of the diagnostic pathway.

### 4.1. Approach to the Patient Meeting Criteria for Instability

Initial screening of the patient with chest pain in the emergency department must check for signs of instability ( Table 12 ) which are necessarily indicative of a worse prognosis. In this scenario, an accelerated approach, aiming for an even faster diagnosis, can play a key role in therapeutic intervention, with potential impact on reducing mortality ( Table 13 ).^91^

**Table 12:** Clinical indicators of instability

Persistent chest pain
Low blood pressure
Tachyarrhythmia / bradyarrhythmia
Severe dyspnea / acute pulmonary edema
Diaphoresis
Cold / clammy extremities
Reduced distal pulses
Decreased level of consciousness / confusion
Reduced urinary output
Prolonged capillary refill time (> 3 seconds)

* In recent years, echocardiography and point-of-care ultrasound (POCUS) have become increasingly available in emergency and intensive care settings. When used properly, this tool can aid in the rapid differential diagnosis of many serious conditions.

**Table 13:** Recommendations for patients with chest pain and hemodynamic instability

	Class of recommendation	Level of evidence
When identifying clinical characteristics suggestive of instability, leading causes should be investigated first, through a history and summary physical examination.	I	**C**
Especially when the etiology of shock remains unclear after clinical assessment, STAT echocardiography and/or point-of-care ultrasound, performed by a trained healthcare provider, is indicated.	I	**C**

In addition to clinical criteria for instability ( Table 12 ), patients with a "concerning" ECG should undergo an expedited workup. In all such cases (classical instability criteria and a "concerning" initial ECG), in addition to expedited investigation with bedside imaging, early repetition of ECG is mandatory to assess dynamic changes and facilitate rapid diagnosis.

It bears stressing that patients in life-threatening condition are generally very anxious, severely dyspneic and diaphoretic. The main conditions that should be investigated in unstable patients on the chest pain pathway are: ACS, aortic dissection, pulmonary embolism, cardiac tamponade, tension pneumothorax, and esophageal rupture. In addition to the classic basic assessment methods (history and physical examination, ECG, chest X-ray), echocardiography/POCUS is now an essential tool in the differential diagnosis of these patients:

- **Cardiogenic shock (ventricular failure):** the most common cause of non-arrhythmic hemodynamic instability during evaluation within a chest pain protocol (first 24 hours). The main predictors of cardiogenic shock in patients with ACS are age > 70 years, hypotension, Killip class ≥ 2, and tachycardia.^92^On echocardiography, there is marked left ventricular (LV) systolic dysfunction, dilation of the inferior vena cava (without respiratory variation), and the presence of B-lines on lung ultrasound. Early identification is important to enable rapid revascularization and the use of ventricular assist devices – measures with the potential to reduce mortality in patients with cardiogenic shock.^93^Cardiogenic shock may also occur in right ventricular (RV) infarction; in these cases, findings typically include RV systolic dysfunction and inferior vena cava dilation (without respiratory variation), but without B-lines on lung ultrasound;
- **Mechanical complications of myocardial infarction:** these generally occur later in the disease course, after several days of hospitalization, not during the initial approach to the patient with chest pain. The typical presentation is a non-revascularized patient who suddenly develops hemodynamic instability and physical examination changes (new murmur, jugular venous distension, new crackles on lung auscultation). The echocardiogram may show a variety of abnormalities depending on the type of mechanical complication: pericardial effusion in LV free wall rupture; severe mitral regurgitation in chordae tendineae rupture or papillary muscle rupture; or left-to-right shunt with RV dilation in post-infarction ventricular septal defect (VSD);
- **Aortic dissection:** ^94^the diagnostic evaluation of aortic dissection, including clinical probability algorithms, is described in item 4.2.2. When hemodynamic instability is present, aortic rupture, acute aortic regurgitation, or cardiac tamponade should be suspected – conditions associated with high mortality. On transthoracic echocardiography, the suprasternal window can aid in identifying dissection, as can the parasternal long-axis view. The presence of an intimal flap in a patient with a compatible clinical presentation supports the diagnosis. However, the flap will not always be visible. In such cases, indirect findings such as aortic dilatation reinforce the need to continue diagnostic investigation for dissection. In general, transthoracic echocardiography has limited accuracy for diagnosing acute aortic syndrome (AAS) and therefore is not used to establish the definitive diagnosis, although it does provide accurate information on the presence of aortic regurgitation and on left ventricular systolic function. Transesophageal echocardiography has an accuracy > 90% and a negative predictive value close to 100%, and is the preferred diagnostic method in unstable patients who cannot undergo CT angiography;
- **Pulmonary thromboembolism:** PE is another major cause of chest pain with hemodynamic instability. As for AAS, the diagnostic workup of PE (including clinical prediction algorithms) is described in item 4.2.3. Hemodynamic instability is indicative of a massive PE, with right ventricular strain significant enough to compress the left ventricle and cause a drop in blood pressure. Physical examination will show jugular venous distension and no evidence of congestion on pulmonary auscultation. The echocardiogram will demonstrate a dilated right ventricle (RV) with significant contractile dysfunction. There may be apical hypercontractility with akinesis of the free portions of the RV (McConnell's sign). The left ventricle may be "D-shaped" due to significant pressure overload from the RV. In patients with clinical suspicion of PE who are too unstable to undergo CT pulmonary angiography, evidence of right ventricular overload on transthoracic echocardiography (once other causes of hemodynamic instability have been ruled out) can be used as indirect evidence of PE to indicate treatment. Transesophageal echocardiography, by allowing direct visualization of the thrombus within the pulmonary artery, can establish a definitive diagnosis of PE even in hemodynamically unstable patients;
- **Cardiac tamponade:** Cardiac tamponade should be a clinical diagnosis, usually defined by the classic triad of shock, jugular venous distension and muffled heart sounds. Pulsus paradoxus is a common finding and reflects ventricular interdependence. Echocardiography is useful for assessing pericardial effusion volume and evidence of restricted ventricular filling. Every patient in shock with any degree of pericardial effusion should be treated as a potential case of cardiac tamponade, as the speed of onset of the effusion may be more significant than its volume to the development of tamponade;
- **Tension pneumothorax:** Tension pneumothorax is also a clinical diagnosis and one that must be recognized quickly, as it causes significant hemodynamic instability and can be fatal. It generally occurs in cases of thoracic trauma, post-procedural events (e.g., accidental central venous puncture), and/or in younger patients or those with underlying lung disease. In equivocal cases, ultrasound will demonstrate absence of pleural sliding and prominence of A lines;
- **Esophageal rupture (Boerhaave syndrome):** esophageal rupture poses a greater diagnostic challenge. The key to clinical diagnosis is the finding of subcutaneous emphysema and pneumomediastinum in a patient with a suspicious presentation (e.g., elevated body temperature, a history of vomiting followed by sudden onset of chest and/or upper abdominal pain, and hemodynamic instability). Plain chest radiography and CT scan play an important role in establishing the diagnosis.

#### 4.1.1. Routine Echocardiography in the Unstable Patient with Chest Pain

Echocardiographic examination should be performed routinely in these patients to ascertain the cause of instability. The echocardiogram will allow evaluation of global and regional ventricular function; size of the cardiac chambers; valvular, pericardial, or aortic diseases; and blood volume. Lung ultrasound can further identify pulmonary involvement.^95 , 96^

The recommended routine ultrasound protocol for these patients is as follows:

1. Parasternal window: Assess left ventricular function, mitral involvement, check aortic diameter and RV size;
2. Apical 4-chamber view: Assess left and right ventricular function, valve involvement, and chamber sizes;
3. Subcostal window: Assess left and right ventricular function, check for pericardial effusion, assess blood volume;
4. Suprasternal view: useful for evaluating the aortic arch (e.g., in suspected aortic dissection);
5. Mid-clavicular and anterior axillary lines: lung ultrasound (to check for pulmonary congestion and/or pneumothorax).

Figures 7 and 8 demonstrate these echocardiographic views and the structures that should be identified.

**Figure 7:** Echocardiographic windows.

**Figure 8:** Identification of structures according to the assessed windows. AO: aorta, AS: acoustic shadow, AV: aortic valve, ICS: intercostal space, LA: left atrium, LCCA: left common carotid artery, LIV: liver, LSA: left subclavian artery, LV: left ventricle, MV: mitral valve, PA: pulmonary artery, PL: pleura, RV: right ventricle, TV: tricuspid valve.

The Supplement includes examples of some pathological conditions identified on echocardiography/ultrasound ( Figures S3 to S6 ) and an algorithm for assessment of the unstable patient with chest pain (Figure S7).

### 4.2. Clinical Risk Stratification and Diagnostic Probability Scores

Chest pain evaluation involves an extensive interpretation of predictors and clinical findings to ascertain the most likely diagnosis. Several classifications for chest pain have been proposed (typical vs. atypical, types A/B/C/D); however, the authors of the present guideline recommend that it be classified into 3 groups: 1) highly suspicious symptoms (e.g., ischemic cardiac pain); 2) moderately suspicious symptoms (e.g., possibly ischemic cardiac pain); and 3) slightly/not at all suspicious symptoms (e.g., noncardiac chest pain). This classification facilitates application of the HEART score and avoids terms that can lead to miscommunication (e.g., "atypical" pain may be interpreted as noncardiac pain). Furthermore, avoiding the term "chest pain" means chest pain equivalents are covered as well. At any rate, this classification only represents part of the basic assessment; other elements must be evaluated jointly.

Risk assessment and clinical probability scores can be useful to avoid having a subjective assessment (i.e., one dependent on the evaluator's experience) as the only factor underlying diagnostic decision-making in a patient with chest pain (or equivalent symptoms) and a nondiagnostic ECG, and should thus be used routinely in the ED. In an attempt to enhance this assessment with more assertive investigations, optimize utilization of diagnostic resources, and, most importantly, avoid inadvertent discharges, several scores have been designed and internationally validated to stratify the risk and diagnostic probability of those conditions having the greatest impact on morbidity and mortality.

Scores validated in clinical practice can be used in patients with suspected ACS, acute aortic syndromes (most commonly acute aortic dissection), and PE. These scores integrate data from the patient's history, symptoms, clinical examination findings, ECG/imaging findings, and biomarker levels, and should be used as part of the chest pain workup. High-sensitivity cardiac troponin algorithms can be used concomitantly with ACS clinical scores (e.g., HEART score), although the incremental benefit of adding clinical scores to high-sensitivity cardiac troponin algorithms remains controversial. (see section 4.3).

#### 4.2.1. Clinical Risk Scores for Suspected ACS Cases

The scores used in the initial assessment of suspected ACS cases do not have the capacity to rule out or rule in this diagnostic hypothesis, and are not intended to. However, these scores can predict the diagnostic probability of ACS and, especially, stratify the future risk of adverse outcomes in these patients, depending on the score and its classification.

The best-studied such score in the population with acute chest pain is the HEART score^97 – 101^( Table 14 ), which is most applicable in the urgent-care or emergency department setting. The original study evaluatx conventional troponin levels as a predictor. However, more recent studies have validated the use of high-sensitivity cardiac troponin (hs-cTn), which is currently the preferred biomarker, as a parameter in the HEART score. Furthermore, the original study was designed to assess the risk of major adverse cardiovascular events (MACE) at 6 weeks; however, as this risk is associated with the clinical likelihood of ACS, the HEART score result can assist the physician's decision on further investigation (in addition to troponin).

**Table 14:** HEART Score

Escore HEART	
History	2 = highly suspicious
	1 = moderately suspicious
	0 = slightly/not at all suspicious
ECG	2 = significant ST deviation
	1 = nonspecific repolarization disturbance
	0 = normal
Age (years)	2 = ≥ 65
	1 = ≥ 45 to < 65
	0= < 45
Risk (factors * )	2 = ≥ 3 or history of atherosclerotic disease
	1 = 1 or 2
	0 = none
Troponin (conventional) **	2 = ≥ 3x the upper limit of normal
	1 = 1–3x the upper limit of normal
	0 = ≤ upper limit of normal

ECG: electrocardiogram.

* Risk factors: Hypertension, hypercholesterolemia, diabetes, obesity (BMI >30 kg/m²), smoking (current or cessation ≤3 months), early positive family history (first-degree relative).

** The original HEART score used conventional troponin; its modified version for high-sensitivity troponin is based on the HEAR score (History, ECG, Age, Risk) and also uses specific cut-off points from high-sensitivity troponin algorithms.

Other scores like ADAPT^102^and EDACS,^103^although they do provide alternatives to the HEART score, do not offer significant advantages; furthermore, HEART outperformed both in a Brazilian study.^104^Some risk stratification and prognostic scores also applied to patients with ACS, such as TIMI and GRACE, have been analyzed in the setting of initial assessment of chest pain and also demonstrated inferior performance to that of HEART.^104^Therefore, the HEART score is preferred, as it has superior accuracy, better validation, and is extremely easy to use in clinical practice.^105 – 107^It bears stressing that patients with diagnostic ECG changes or troponin levels (in the presence of a consistent clinical picture) do not require the HEART score and should follow the appropriate treatment pathway, as the diagnosis would already be established ( Figures 4 , 11A and 11B ).

**Figure 11-A:** 0-1H and 0-2H pathways – High-sensitivity cardiac troponin (hs-cTn).

**Figure 11-B:** Troponin values to be used in the 0-3 hour flowchart.

##### 4.2.1.1 Which Patients Should Have Their HEART Score Calculated?

The HEART score was developed to assess the risk of major cardiovascular events within 6 weeks in patients with suspected ACS; this is the population that benefits most from the score. Therefore, within the context of this guideline, the HEART score is most useful when the clinician has to decide whether it is safe to discharge a patient home ( Table 15 ). Since the risk of short-term cardiovascular events (within 6 weeks) is associated with diagnostic probability, the HEART score is also useful to inform the calculation of pretest probability, which assists in the decision to order additional investigations:

- HEART SCORE: 0 to 3 points (low risk of MACE within 6 weeks);
- HEART SCORE: 4 to 6 points (moderate risk of MACE within 6 weeks);
- HEART SCORE: 7 to 10 points (high risk of MACE within 6 weeks).

**Table 15:** Risk stratification and diagnostic probability scores in the assessment of patients with chest pain and suspected acute coronary syndrome

	Class of recommendation	Level of evidence
The HEART score is the preferred clinical score for patients with chest pain who are undergoing a diagnostic workup for ACS.	I	B

ACS: acute aortic syndrome.

#### 4.2.2. Clinical Scores for Suspected Acute Aortic Syndromes

Although rare, acute aortic syndromes (AAS) are extremely serious conditions with high mortality rates. The largest international registries record a mortality rate of approximately 1 to 2% per hour in the first 48 hours.^108^Acute aortic dissection (AAD) accounts for approximately 70% of acute aortic syndromes.

Aortic dissection has a wide spectrum of clinical presentations, but chest pain is the most common symptom; it is usually severe, radiating to the back, and not relieved by any factors. Widening of the superior mediastinum is the most common finding on chest radiography (present in 60 to 90% of cases). However, it is important to emphasize that a normal chest X-ray cannot rule out aortic dissection.

In addition to their heterogeneous clinical presentation, the manifestations of AAS may sometimes be similar to those of other causes of chest pain.^109^Therefore, the assessment of patients with suspected AAS is often challenging for the physician, who may sometimes prescribe inappropriate treatment due to overlap of signs and symptoms with those of other conditions; order excessive imaging tests to rule out the diagnosis; or even inadvertently discharge an undiagnosed patient (failure to diagnose these time-sensitive diseases drastically worsens prognosis and hinders treatment).

Thus, in an attempt to facilitate the initial assessment, reduce the possibility of errors, and improve the cost-effectiveness diagnostic imaging, two clinical prediction scores for AAS were developed: the Aortic Dissection Detection Risk Score (ADD-RS) and the Aorta Simplified Score (AORTAs).^110 – 112^

The ADD-RS encompasses 12 variables distributed across 3 categories, as shown in Table 16 . Presenting with any of the variables under each category scores 1 point; the maximum sum score is therefore 3 points. Patients with a score of 0 or 1 are characterized as low-risk, and those with a score of 2 or 3, as high-risk.^110 , 111^The addition of bedside focused cardiac ultrasound (FoCUS) to the ADD-RS improved assessment of diagnostic probability.^111^

**Table 16:** Aortic Dissection Detection Risk Score (ADD-RS)

Predisposing conditions	Pain characteristics	Clinical examination findings
- Marfan syndrome or other connective tissue disease - Family history of aortic disease - Known aortic valve disease - Recent aortic manipulation - Known thoracic aortic aneurysm	Chest or abdominal pain described as: - Abrupt in onset - Severe in intensity - "Tearing" or "ripping"	- Pulse deficit or blood pressure differential - Focal neurological deficit (in conjunction with pain) - New aortic regurgitation murmur (in conjunction with pain) - Hypotension or shock

The AORTAs, in turn, is composed of 6 clinical features as shown in Table 17 . Patients with a score of 0 or 1 are classified as having low risk of AAS, and those with a score of 2 or higher, as high-risk. It is a simplified score with high sensitivity, but still requires further validation before its use in clinical practice can be expanded.^112 , 113^

**Table 17:** AORTA pretest probability score – for acute aortic syndrome

Clinical feature	Score
Hypotension/shock	2
Aneurysm	1
Pulse deficit	1
Neurological deficit	1
Severe pain	1
Sudden onset of pain	1

It is worth noting that the ADD-RS and AORTAs are pretest probability scores, which rely on clinical data alone to identify patients with low or high risk of AAS. Neither has sufficient accuracy to rule in or rule out the diagnosis of AAS if used alone ( Table 18 ).^113^

**Table 18:** Risk stratification and diagnostic probability scores in the assessment of patients with chest pain and suspected acute aortic syndrome

	Class of recommendation	Level of evidence
The ADD-RS can be used as part of the initial assessment of patients with suspected AAS.	IIa	B
Point-of-care ultrasound (POCUS), performed by a trained physician, can be added to the ADD-RS to enhance the assessment of diagnostic probability.	IIa	B
The AORTAs can be used in addition to or as an alternative to the ADD-RS in patients with suspected AAS.	IIb	C
The ADD-RS and AORTAs must **NOT** be used in isolation to rule out or rule in a diagnosis of AAS in the initial assessment of patients with suspected AAS.	III	B

AAS: acute aortic syndrome; ADD-RS: Aortic Dissection Detection Risk Score.

In cases where AAS is suspected and the pretest diagnostic probability has been established, the appropriate investigation can be pursued (steps 2 and 3) according to the flowchart ( Figure 9 ):

**Figure 9:** Flowchart for the investigation of acute aortic syndrome.

- Low pretest probability: Perform D-dimer (if negative, pursue an alternate diagnosis; if positive, obtain imaging*);
- Pretest probability above the "low" threshold: Obtain imaging.

*Imaging exams usually indicated for AAS workup include CT angiography (most common) and transesophageal echocardiography (when CT angiography cannot be performed).

#### 4.2.3. Clinical Scores for Suspected Pulmonary Embolism

In pulmonary embolism (PE), chest pain is usually sudden in onset and accompanied by dyspnea and pleuritic symptoms. The finding of risk factors for thromboembolic disease usually aids in diagnosis.

Plain chest radiography is often abnormal in PE; however, *t* hese abnormalities are usually nonspecific (in any case, it is a useful modality to rule out other causes of dyspnea and chest pain, such as pneumothorax). The most common radiographic changes in PE are areas of atelectasis, elevated hemidiaphragm, pleural effusion, and enlargement of the trunk and branches of the pulmonary artery. Areas of regional pulmonary oligemia (Westmark sign) and a wedge-shaped pulmonary opacity with the base facing the pleura (Hampton hump) are the X-ray findings most specific for PE, but unfortunately, they are not very sensitive.

ECG changes indicative of right heart strain (T-wave inversion from V1–V4, S1Q3T3, complete or incomplete RBBB) are only seen in the most severe cases of PE; in less severe cases, the most common finding is sinus tachycardia, which, in addition to being nonspecific, is only present in approximately 40% of patients.

Considering that the major manifestations of PE are all nonspecific, the use of clinical prediction rules is important to reduce unnecessary variability in the subjective adjudication of diagnostic probability. The combination of clinical findings in patients with risk factors for PE allows these patients to be stratified into clinical categories by pretest probability, in an attempt to identify which investigation would be most appropriate to rule in or rule out PE.

Three scores are commonly used in patients with suspected PE: the Wells score, the Geneva score (or rule), and the simplified Geneva score. It is worth noting that these scores have not been validated in pregnant patients or patients with thrombophilia.

The Wells score encompasses 7 variables ( Tables 19 and 20 ).^114^

**Table 19:** Wells score: criteria and respective scores

Criteria	Score
Clinical evidence of DVT	+3
No alternative diagnosis more likely than PE	+3
History of DVT or PE	+1.5
HR > 100 bpm	+1.5
Immobilization for > 2 days or surgery in the previous 4 weeks	+1.5
Hemoptysis	+1
Malignancy (current or treated in the last 6 months, or if the patient is under palliative care)	+1

DVT: deep vein thrombosis; PE: pulmonary embolism; HR: heart rate; bpm: beats per minute.

**Table 20:** Modified Wells score and probability/risk and risk of PE depending on the score

	3 levels	
Score	Probability of PE	Pretest probability
< 2	1 to 3%	Low
2-6	16 to 28%	Intermediate
> 6	> 40%	High
	**2 levels**	
**Pontuação**	**Probability of PE**	**Pretest probability**
≤ 4	3%	Unlikely
> 4	28 %	Likely

PE: pulmonary embolism.

The Geneva score is more laborious in daily practice, but it determines pretest probability of PE patients with suspected thromboembolism as well as the Wells score.^115^The simplified Geneva score allows for greater ease of application and has been validated in studies with the same utility as the revised Geneva score ( Table 21 ).^116^

**Table 21:** Revised and simplified Geneva score and probability and risk of pulmonary embolism depending on the total score

Geneva score	Revised – points	Simplified – points
Age > 65 years	1	1
Previous DVT or PE	3	1
Surgery or fracture within last month	2	1
Active malignant condition	2	1
Unilateral lower limb pain	3	1
Hemoptysis	2	1
Pain on deep palpation of lower limb/unilateral edema	4	1
HR 75-94 bpm	3	1
HR ≥ 95 bpm	5	2

**Score – revised Geneva**	**Probability of PE**	**Pretest probability**
0-3	8%	Low
4-10	28%	Intermediate
≥ 11	> 60%	High

**Score – simplified Geneva**	**Pretest probability**	
**2 levels**		
0-2	Unlikely	
≥ 3	Likely	

DVT: deep vein thrombosis; PE: pulmonary embolism; HR: heart rate; bpm: beats per minute.

Some diagnostic protocols for PE incorporate a two-tiered or dichotomous assessment of the Wells and Geneva scores to categorize patients more easily (PE "likely" or "unlikely" rather than low, intermediate, and high probability). PE is considered "likely" when a patient scores 5 or higher on the Wells score, and 3 or higher on the simplified Geneva score. In turn, PE is considered "unlikely" when the score is less than or equal to 4 for the Wells score, or less than or equal to 2 for the simplified Geneva score. Both forms of stratification can be used (traditional three-tiered and two-tiered), each following a specific assessment protocol.^117^

In patients whose initial clinical assessment shows that a diagnosis of PE is unlikely (estimated probability less than 15%, e.g., Wells score < 2), the Pulmonary Embolism Rule-out Criteria (PERC) can be used. This is an easy-to-administer score, in which the absence of all items ( Table 22 ), the diagnosis can be ruled out without the obligation further diagnostic testing.^118^Thus, more than one score can be used to assess the diagnostic probability of PE ( Table 23 ).

**Table 22:** Pulmonary Embolism Rule-out Criteria (PERC)

PERC
- Age ≥ 50 years
- Heart rate ≥ 100 bpm
- O2 saturation < 95% on room air
- Asymmetric lower limb edema
- Hemoptysis
- Surgery or trauma within last 4 weeks
- Prior DVT or PE
- Hormone therapy

DVT: deep vein thrombosis; PE: pulmonary embolism.

**Table 23:** Risk stratification and diagnostic probability scores in the assessment of patients with chest pain and suspected pulmonary embolism

	Class of recommendation	Level of evidence
The Wells score, revised Geneva score, and simplified Geneva score should be used to stratify pretest probability as part of the initial assessment of patients with suspected PE.	I	A
The PERC score can be used in the initial assessment of patients with suspected PE to rule out PE if the pretest probability has been established as low.	IIa	B

PE: pulmonary embolism; PERC: pulmonary embolism rule-out criteria.

In cases where PE is suspected and the pretest diagnostic probability has been established, the appropriate investigation can be pursued (steps 2 and 3) according to the flowchart ( Figure 10 ):

**Figure 10:** Diagnostic flowchart for PE.

- Low pre-test probability and zero PERC: D-dimer not mandatory;
- Low pre-test probability (especially if PERC > 0) or intermediate (PE unlikely in a two-level assessment): Perform D-dimer using ≥500 ng/mL (μg/L) as the cutoff value or an age-adjusted cutoff (age × 10 for patients aged 50 years or older); if positive, perform imaging* (if negative, seek an alternative diagnosis);
- High pretest probability in a three-level assessment (or likely in a two-level assessment): Obtain imaging.

*Imaging exams usually included in the PE workup: CT angiography (most commonly used), ventilation-perfusion scan (in selected cases when there are limitations to CT angiography), and echocardiographic evaluation of PE (when the patient is too unstable and/or otherwise unable to undergo CT angiography).

#### 4.2.4. Other Differential Diagnoses

Although validated probability scores are not available for all differential diagnoses of chest pain, it is important to keep the major features of these syndromes (e.g., pericarditis) in mind as part of clinical reasoning. In the setting of chest pain, acute pericarditis or myopericarditis (if pericarditis is associated with myocarditis) causes precordial pain (usually respirophasic) and often presents with fever and malaise. Rapid and/or massive increase in pericardial fluid volume may cause restriction of cardiac filling (tamponade). The diagnosis of pericarditis is generally established when 2 of the following 4 findings are present:

1. Characteristic chest pain;
2. Pericardial friction rub;
3. Characteristic ECG findings;
4. Characteristic imaging findings (e.g., on echocardiogram).

Other life-threatening causes of chest pain (e.g., esophageal rupture, tension pneumothorax) are rarer still but should be considered in the investigation if the clinical picture is consistent. Physical examination is usually sufficient for the diagnosis of tension pneumothorax (absent breath sounds, hyperresonance to percussion, jugular venous distension); in the case of esophageal rupture, onset of chest pain after vomiting with subsequent fever and hypotension should raise suspicion of this diagnosis (many cases present with changes indicative of pneumomediastinum on chest radiography).

Once life-threatening diagnoses have been ruled out, more benign possibilities (anxiety, uncomplicated dyspepsia, etc.) can be entertained.

### 4.3. Troponin Algorithms

#### 4.3.1. Use and Interpretation of Biomarkers of Myocardial Injury

Over 90% of patients on a chest pain pathway have a "nondiagnostic" ECG. Even among cases that will eventually have a confirmed diagnosis of ACS, about half will have a nondiagnostic ECG.^119^Therefore, when a patient with chest pain has no changes characteristic of ACS on a resting ECG, biomarkers of myocardial injury should be measured to rule in or rule out this diagnosis.

##### 4.3.1.1. What is the Biomarker of Choice for Diagnosis of MI?

Tests to rule in or rule out acute coronary syndrome must be effective and fast. The ideal biomarker should be as specific and sensitive as possible, i.e., it should be able to detect changes specific to the heart muscle even at low serum concentrations.^77^

Currently, the biomarker that best combines these characteristics is troponin, whether cardiac troponin T (cTnT) or cardiac troponin I (cTnI). In centers where quantitative troponin measurement is available, measurement of CK-MB or other so-called cardiac biomarkers is no longer recommended as part of the diagnostic workup, as troponin has superior sensitivity and specificity.^77 , 120^

As troponin assays have gradually improved, several "generations" are available with varying characteristics, especially with regard to sensitivity. High-sensitivity cardiac troponin (hs-cTn) is now the preferred biomarker of myocardial injury for use in patients with suspected ACS. As the name implies, high-sensitivity cardiac troponins (hs-cTn) are significantly more sensitive than conventional troponins for the diagnosis of MI.^77 , 120^

##### 4.3.1.2. How to Interpret the Abnormal Troponin Test

Troponin is a biomarker of myocardial injury. To ascertain the etiology of this injury (e.g., diagnose an AMI), we must integrate findings from clinical examination and from other investigations. Although the accuracy of high-sensitivity cardiac troponin (hs-cTn) assays has improved in recent years, several clinical conditions other than type 1 acute myocardial infarction can cause myocardial injury and, consequently, elevated troponin levels.

##### 4.3.1.3. How to Interpret Serial Troponin Tests

The results of hs-cTn tests must be analyzed in relation to the timing of symptom onset and must be repeated serially at predetermined intervals for adequate interpretation of any changes over time.

Acute myocardial injury is defined as a rise and/or fall in hs-cTn values > 20% between measurements, provided that at least 1 of these values is above the 99th percentile defined for that particular assay.^77^Reference values differ by sex and age.^77 , 120 , 121^

Chronic myocardial injury is defined by a variation in troponin values ≤ 20% between measurements, with at least one of these values above the 99th percentile of the assay used. Analysis of absolute values is more useful in patients with lower troponin levels; therefore, early rule-out algorithms are based on the change (delta) in absolute values.^121^

In addition to the need to assess pretest probability when interpreting troponin results, it is important to remember that high-sensitivity troponins, although highly accurate for detecting myocardial injury, do not determine the cause of the injury (e.g., whether it is myocardial infarction or another cause), and that false-positive and/or false-negative results, although rare, may occur.^122^

##### 4.3.1.4. Diagnostic Criteria for MI

The diagnosis of acute myocardial infarction is established when the cause of acute myocardial injury is found to be coronary insufficiency. To confirm myocardial infarction, acute myocardial injury (defined as a rise and/or fall of troponin with at least one measurement above the 99th percentile^114^) must be combined with other clinical and/or laboratory findings of ischemia:^77^

1. Symptoms characteristic of ischemia;
2. ECG changes indicative of new ischemia (including development of pathological Q waves);
3. Imaging evidence of new loss of viable myocardium or new regional wall motion abnormality with an ischemic pattern;
4. Angiography or autopsy evidence (the latter alone may be sufficient for the diagnosis of MI).*

*A finding of coronary thrombus on angiography or autopsy is a diagnostic criterion for type 1 MI, but is not part of the criteria for type 2 or type 3 MI.

The criteria for type 4b (stent thrombosis) and 4c (stent restenosis) MI are the same as for type 1 MI; however, there must be evidence of stent thrombosis or restenosis respectively on angiography (in type 1 AMI, the mechanism is atherothrombosis, usually due to plaque erosion or rupture). Type 3 AMI represents those cases of sudden death in which there is not enough time to establish a diagnosis of MI before the fatal outcome, whereas type 4a and type 5 MI are periprocedural. Therefore, these types of MI (3, 4a, and 5) fall outside the scope of this guideline.

##### 4.3.1.5. What is the Time Limit to Rule Out a Diagnosis of MI?

This depends on the troponin assay.

In patients with high-sensitivity troponin testing, if symptom onset occurred more than 3 hours earlier (and without recurrence), a level below the threshold classified as "very low" would be sufficient to rule out acute MI (although it would not exclude other diagnoses, such as unstable angina). In other situations, at least one repeat troponin measurement must be obtained in addition to the initial measurement, although very high values (e.g., > 5 times the upper limit of normal) on a single measurement are indicative of a high likelihood of MI if there is clinical suspicion. Interpretation of serial measurements should be based on validated algorithms or pathways.^104^

For troponin assays that do not meet high-sensitivity criteria, it is necessary to wait 6 to 12 hours (depends on the cutoff point of the conventional troponin assay kit). A negative result after this interval from the last episode of symptoms can rule out acute MI, although it does not exclude other high-risk diagnoses.

##### 4.3.1.6. What is the Algorithm of Choice?

Several high-sensitivity troponin algorithms have been proposed with the aim of reducing the time to rule-in and rule-out of acute MI. Regardless of algorithm, it is recommended that the first troponin measurement be collected upon patient intake. In cases without sufficient time and biomarker levels to allow for rule-out, the biomarker measurement should be repeated.

The 0/3-hour algorithm, in which 2 troponin values below the 99th percentile obtained at these time points are sufficient to rule out MI, was evaluated in several studies (including a meta-analysis) and was shown to be inferior to earlier algorithms such as the 0/1-hour and 0/2-hour algorithms.^123^Use of either the 0/1-hour or 0/2-hour algorithm should be preferred, respecting the cutoff and absolute variation values predefined for whichever assay or kit is used ( Tables 24 , 25 and 26 . Figures 11A and 11B ).

**Table 24:** High-sensitivity troponin clinical decision algorithms

Algorithm	Rule-out criteria	Advantages	Disadvantages
**0/3h (should only be considered if the 0/1-h or 0/2-h algorithms cannot be used)**	- If symptom duration > 6 hours (currently asymptomatic), single measurement < 99th percentile OR - If symptom duration < 6 hours, with serial measurements (every 3 hours) < 99th percentile	Uses 99th percentile as decision threshold (similar to the use of the reference value of conventional troponin, with greater physician familiarity)	Fewer patients meet the rule-out criteria and, when they do, this happens later (lower sensitivity)
**0h (single measurement)**	Measurement below the threshold or detection or a validated cutoff if symptom duration > 3h	Immediate decision (no need for a second measurement)	It is recommended that the patient seek medical care early (late presentation is undesirable from a public health standpoint)
**0-1h (preferred algorithm)**	Uses admission troponin (0h) and change (delta) at 1h to define disposition: discharge (rule out), observe, or admit (rule in)	Bypasses issues inherent to use of the 99th percentile and is able to rule out more cases, earlier 1 *	The timing of collection is crucial to interpretation of this algorithm, and its values are difficult to memorize 2 *
**0-2h (alternative when the 0/1-h algorithm cannot be performed)**	Identical to 0-1h algorithm, except the change (delta) is evaluated at 2 hours rather than at 1 hour	Advantages similar to those of 0-1h algorithm 1 * and it is more feasible at centers that cannot implement 0-1h	Disadvantages similar to those of 0-1h plus less validation and fact that definition occurs with a 1-hour "delay" 2 *
**High-STEACS**	- If symptom duration > 3 hours (currently asymptomatic), single measurement < 5 or 6 ng/L 3 * OR - If change (delta) between 0h and 3h is < 3 ng/L, with troponin remaining < 99th percentile (adjusted for sex)	Also takes advantage of the sensitivity and accuracy of hs-cTn and uses a sex-adjusted percentile	Fewer patients are categorized in the rule-out group than by the 0-1h and 0-2h algorithms (in addition to a lower rule-out cutoff, rule-out occurs with a longer delay)

1 Both (0-1h and 0-2h) take advantage of the improved sensitivity and accuracy of high-sensitivity cardiac troponin (hs-cTn).

2 Both require caution in cases of elevated troponin without dynamic change (delta) at 1 or 2 hours as this could be the plateau phase of an acute MI (when troponin levels are quite high; this issue would be mitigated as a high 1-hour level would already "rule in" MI regardless of the delta).

3 < 5 ng/L for high-sensitivity troponin I or 6 ng/L for high-sensitivity troponin T

**Table 25:** Myocardial injury biomarker algorithms and their use

	Class of recommendation	Level of evidence
High-sensitivity cardiac troponin is the biomarker of choice for investigation of acute MI.	I	B
If high-sensitivity cardiac troponin is not available, measure an alternative available biomarker * every 3 hours until enough time has elapsed to rule in or rule out the diagnosis of acute MI (this depends on the biomarker used).	I	B
The 0/1h and 0/2h high-sensitivity cardiac troponin algorithms are preferable to 0/3h algorithms.	IIa	B
In patients with chest pain of more than 3 hours’ duration, a single high-sensitivity cardiac troponin measurement below the limit of detection of the assay may be sufficient to rule out AMI if clinical scores have stratified the patient as low risk, there are no ischemic changes on ECG, and there is no recurrent or persistent pain.	IIa	B
If quantitative troponin is available, CK-MB should not be part of the MI workup.	III	B

* In the absence of high-sensitivity cardiac troponin, conventional troponin may be used or, if not available, CK-MB mass. AMI: acute myocardial infarction.

**Tabela 26:** Troponin values to be used with the 0-1h/2h algorithm

Assay	Very low	Low	No 1 h Δ	High	1 h Δ
hs-cTnT (Elecsys; Roche)	<5	<12	<3	>=52	>=5
hs-cTnI (Architect; Abbott)	<4	<5	<2	>=64	>=6
hs-cTnI (Centaur; Siemens)	<3	<6	<3	>=120	>=12
hs-cTnI (Access; Beckman Coulter)	<4	<5	<4	>=50	>=15
hs-cTnI (Clarity; Singulex)	<1	<2	<1	>=30	>=6
hs-cTnI (Vitros; Ortho-Clinical Diagnostics)	<1	<2	<1	>=40	>=4
hs-cTnI (Pathfast; LSI Medience)	<3	<4	<3	>=90	>=20
hs-cTnI (TriageTrue; Quidel)	<4	<5	<3	>=60	>=8
hs-cTnI (Dimension EXL; Siemens)	<9	<9	<5	>=160	>=100
**Assay**	**Very low**	**Low**	**No 2 h Δ**	**High**	**2 h Δ**
hs-cTnT (Elecsys; Roche)	<5	<14	<4	>=52	>=10
hs-cTnI (Architect; Abbott)	<4	<6	<2	>=64	>=15
hs-cTnI (Centaur; Siemens)	<3	<8	<7	>=120	>=20
hs-cTnI (Access; Beckman Coulter)	<4	<5	<5	>=50	>=20
hs-cTnI (Clarity; Singulex)	<1	TBD	TBD	>=30	TBD
hs-cTnI (Vitros; Ortho-Clinical Diagnostics)	<1	<2	<3	>=40	>=5
hs-cTnI (Pathfast; LSI Medience)	<3	<4	<4	>=90	>=55
hs-cTnI (TriageTrue; Quidel)	<4	TBD	TBD	>=60	TBD

The cut-offs apply irrespective of age, sex, and renal function. Optimized cut-offs for patients above 75 years of age and patients with renal dysfunction have been evaluated, but not consistently shown to provide better balance between safety and efficacy as compared with these universal cut-offs. The physician should always check with their laboratory which assay kit is being used and the recommended cutoff values (as new tests and assays are under development and cutoff values may change). TBD: to be determined.

If high-sensitivity troponin is not available, conventional quantitative troponin measurement can be used ( Table 25 ). The standard approach consists of an initial measurement (0 h) followed by repeat collections every 3 hours until a sample is obtained more than 6 to 12 hours after symptom onset (the required interval depends on the cutoff value of the troponin assay). In cases of recurrent pain or high clinical suspicion, additional troponin measurements may be obtained. A value above the cutoff will serve as the reference to classify cases as "positive" for myocardial injury (and probable acute MI) or "negative" (not meeting criteria for myocardial injury and, consequently, for acute MI – although some of these could be infarctions if high-sensitivity assays were used).

Figures 11-A and 11-B show presents the recommended algorithms for high-sensitivity cardiac troponin.

#### 4.3.2. Routing According to Classification in the Algorithm

Chest pain protocols and pathways in the emergency department setting seek to quickly diagnose any life-threatening conditions while also identifying patients who can be discharged from the facility without the need for hospital admission. This strategy prevents unnecessary investigations in the emergency department, ward, or CCU for patients with a very low probability or risk of ACS.^79 , 80 , 88^

For this purpose, the combined use of clinical prediction scores, ECG, troponin measurement, and clinical judgment, allows stratification of patients with chest pain who present to the emergency department into three "zones": ACS ruled out, ACS ruled in, and the so-called intermediate zone (observation)^88^( Figure 12 ).

**Figure 12:** Patient stratification according to high-sensitivity troponin algorithms (0/1h, 0/2h, 0/3h).^1^ECG: electrocardiogram; AMI: acute myocardial infarction. * Except for patients with a history of CAD, whose probability of acute MI is > 1% even if the 0/1-h algorithm places them in the "rule-out" group (also consider further evaluation if HEART > 3). **If troponin is ordered indiscriminately (i.e., even in patients with low clinical suspicion), its positive predictive value may be < 50%.

##### 4.3.2.1. ACS Ruled out

Patients without a definite diagnosis after appropriate investigation who have been stratified as low risk on clinical prediction scores^126 , 127^(e.g., HEART ≤ 3 or EDACS < 16), have no ischemic changes on ECG, and have negative troponins within an appropriate time frame are considered to have very low risk/probability of ACS ("rule-out" criteria).

High-sensitivity troponins allow for an earlier and more accurate diagnosis of MI compared to conventional troponins. As with conventional troponin, however, the initial collection must be performed upon the patient's arrival, regardless of the time elapsed since onset of symptoms. In patients whose initial high-sensitivity troponin measurement is very low (i.e., generally below the limit of detection). A single high-sensitivity troponin measurement may be sufficient to rule out acute MI when the ECG is normal and, especially, when clinical scores indicate low risk or low probability of ACS (HEART ≤ 3 or EDACS < 16), provided that the time elapsed between the onset of symptoms and collection of high-sensitivity troponin is greater than or equal to 3 hours.^88 , 128 , 129^

In other cases (symptoms < 3 hours and/or detectable troponin in the first measurement), repeat collection should be performed 1 or 2 hours after the first (if using a high-sensitivity troponin assay validated for 0/1-hour and/or 0/2-hour algorithms). Absence of significant change in high-sensitivity troponin levels after 1 or 2 hours are consistent with a very low probability of ACS. It bears stressing that the cutoff values considered to define a change in hs-cTn as significant vary according to the assay used, as does the 99th percentile ( Table 25 ).^130 – 133^

**Table:** 

What should be done when high-sensitivity troponin is not available?
In situations where hs-cTn are not available, conventional troponin can be used as a biomarker for the diagnosis of AMI. Due to the longer time required for conventional troponin levels to rise, it is considered appropriate to perform two or more collections at a longer interval, the first upon the patient's arrival and the second between 3 and 6 hours later (most conventional kits require more than 6 hours and a third measurement is common).
If there is an elevation of conventional troponin in a compatible clinical and/or electrocardiographic context, the diagnosis of AMI is made.
Despite the lower sensitivity for diagnosing AMI, if at the end of the observation period (generally ranging from 6 to 12 hours according to the conventional troponin kit), patients present with negative troponin and meet (for example, HEART score ≤ 3) with ECG without ischemic alterations, these patients could be discharged from the emergency room, according to the clinical judgment of the medical team (exclusion of high-risk diagnoses).^124 , 125^
CKMB measurement should only be considered in the absence of troponin and preference should be given to CKMB mass.

When there is no significant change in troponin levels at 1 or 2 hours, ruling out other diagnoses, hemodynamically stable patients with no recurrent pain and no ischemic changes on ECG can be discharged from the ED for further investigation in an early outpatient setting, especially if clinical prediction scores are consistent with low risk.^134 – 137^(These 0- and 1-hour algorithms were validated without necessarily adding clinical prediction scores.) However, there is evidence that the troponin algorithm alone is not sufficient for patients with a known history of coronary artery disease; in these cases, a more conservative approach involving additional use of clinical prediction scores may provide an added measure of safety when discharging the patient from the ED after assessment with a high-sensitivity troponin algorithm.^136 – 142^In any case, with or without the support of a clinical prediction score, the physician's judgment and clinical examination are essential at all stages of the decision-making process, as hs-cTn values within normal range rule out MI but do not rule out other diagnoses (e.g., unstable angina).

##### 4.3.2.2. Admission Confirmed (ACS Ruled in)

After clinical assessment and initial ECG, patients meeting diagnostic ECG criteria are classified as having confirmed ACS and should be managed thereafter as per specific ACS guidelines.^88 , 89 , 139 , 140^

However, such patients represent the minority of cases. In most patients investigated for chest pain, the ECG is nondiagnostic and the diagnostic pathway must be followed. The cornerstone of this pathway is serial measurement of troponins to establish a possible diagnosis of acute MI.

A high-sensitivity troponin level above the 99th percentile and/or significant change in troponin levels between measurements, within the criteria of each assay (even if below the 99th percentile), is usually considered to "rule in" myocardial injury ( Figure 12 ). It is then important to evaluate whether the injury is chronic or acute and, in cases of the latter, apply criteria for diagnosis of ACS. If the diagnosis of ACS is confirmed, these patients should then be treated according to ACS guidelines.^88 , 89 , 139 , 140^

As illustrated in Figure 12 , the preferred high-sensitivity troponin algorithms (0/1h and 0/2h) stratify patients into 3 groups and guide subsequent management, always in conjunction with clinical assessment ( Table 27 ).^141 , 142^

**Table 27:** Case disposition according to the troponin algorithm

	Class of recommendation	Level of evidence
In the absence of other high-risk diagnostic hypotheses (e.g., unstable angina, aortic dissection), patients with a "non-diagnostic" clinical-electrocardiographic assessment and who are stratified into the "rule-out" zone by a 0/1h or 0/2h high-sensitivity troponin algorithm may be considered for discharge to further outpatient investigation, especially in the absence of known CAD and presence of low-risk clinical prediction scores (HEART ≤ 3).	I	B
Patients stratified into the "rule-in" zone by a 0/1h or 0/2h high-sensitivity troponin algorithm should be admitted and actively examined for diagnostic criteria of spontaneous AMI and other causes of myocardial injury, initially as inpatients.	I	B
Patients who do not fit into the "rule-in" or "rule-out" groups ("grey-zone" troponin) and/or whose clinical assessment does not meet low-risk criteria (e.g., a high HEART score, persistent suspicion of life-threatening diagnoses) should be considered as having intermediate probability and evaluated on an individual basis, with consideration of additional noninvasive testing (observation group).	I	B

ACS: acute coronary syndrome; CAD: coronary artery disease; ECG: electrocardiogram; AMI: acute myocardial infarction.

#### 4.3.3. Initial Disposition of Intermediate ("Grey-Zone") Cases (Observation)

In the intermediate zone of diagnostic algorithms (i.e., those patients that cannot be classified as "rule-in" or "rule-out"), the probability of acute MI is generally 5 to 20%. These patients are not safe to be discharged home, and additional tools to guide the differential diagnosis are necessary, with careful re-checking of the criteria for spontaneous (type 1 or type 2) acute MI being especially advisable.

In the absence of diagnostic criteria for MI (common for patients in the intermediate zones of hs-cTn algorithms), there is no formal indication for invasive stratification; therefore, further investigation should initially proceed as follows:

- Additional serial troponin measurements (any additional elevations after the second troponin measurement could increase the likelihood of acute MI);
- Echocardiogram to actively search for differential diagnoses;

Noninvasive tests to evaluate coronary artery disease/myocardial ischemia may be considered in non-low-risk patients without a clear diagnosis. The choice of noninvasive test should be based primarily on the availability and experience of each center. Other factors may help in this decision, such as a history of previous CAD. In patients without known CAD, a history of prior invasive or noninvasive testing may influence the physician's decision to continue or terminate the investigation ( Figure 13 ).^79 , 80^In patients with known CAD, the presence or absence of obstructive disease (≥ 50% or history of PCI or CABG) may also influence the choice of noninvasive method ( Figure 14 ).^79 , 80^

**Figure 13:** Flow diagram for noninvasive investigation in a patient without known CAD. ACS: acute coronary syndrome; CAD: coronary artery disease; CT: computed tomography.

**Figure 14:** Flow diagram for noninvasive investigation in a patient with known CAD. CAD: coronary artery disease; CATH: cardiac catheterization; PCI: percutaneous coronary intervention; CABG: coronary artery bypass grafting; LMCA: left main coronary artery; FFR-CT: computed tomography-derived fractional flow reserve.

These flow diagrams for test selection ( Figures 13 and 14 ) can also be used if there is strong clinical suspicion of cardiac ischemia in a stable patient with chest pain who does not meet criteria for ACS (e.g., patient classified as "rule-out" by an hs-cTn algorithm but with additional risk criteria or higher probability of ACS).

In cases where further investigation has ruled out acute MI and other high-risk differential diagnoses but suspicion of ACS persists and access to imaging is more limited, performing an exercise test (in the absence of contraindications) can help refine the diagnostic probability and direct the patient to the best resource.^143 – 161^

If the diagnosis remains unclear at the end of the noninvasive investigation algorithm, coronary angiography may be considered in patients with high clinical suspicion of acute MI (especially if tests are inconclusive after investigation or the patient develops hemodynamic instability while under observation). The main recommendations for this intermediate-probability group are given in Table 28 .

**Table 28:** Initial investigations in cases of intermediate probability

	Class of recommendation	Level of evidence
Order at least one additional troponin measurement (third measurement) and a repeat electrocardiogram (with additional leads).	I	C
Review possible differential diagnoses (with support from clinical prediction scores) and order further testing according to diagnostic probability (e.g., D-dimer testing in suspected PE with low pretest probability).	I	C
If troponin is > 99th percentile, systematically check for acute myocardial infarction criteria as well as for alternate causes of myocardial injury.	I	C
Order an echocardiogram to support diagnostic elucidation if the diagnosis remains unclear after initial investigation of patients with an intermediate probability of ACS.	IIa	C
Investigate coronary artery disease and/or ischemia (preferably with noninvasive modalities) if the diagnosis remains unclear after initial investigation of patients with an intermediate probability of ACS.	IIa	C
The choice of noninvasive test should be based primarily on the availability and experience of each center and on the patient's known history of coronary artery disease or lack thereof.	IIa	C
In patients who have had a completely normal CT coronary angiography in the last 2 years or an appropriate stress test with no evidence of ischemia (negative) in the last year, consider forgoing noninvasive testing if symptoms are stable.	IIb	C

ACS: acute coronary syndrome; PE: pulmonary embolism.

#### 4.3.4. Approach to the Patient with Myocardial Injury and Nonobstructive Coronary Arteries

All patients with abnormal troponin values ("rule-in" and "gray zone") in whom coronary angiography is indicated (including patients meeting MI criteria) must receive a definite diagnosis, even if no obstructions are found on angiography. Therefore, patients presenting with elevated troponin but no coronary obstruction on angiography may be classified initially as TINOCA (troponin-positive nonobstructive coronary arteries), an umbrella classification which can be further divided into three major subgroups:

1. MINOCA (myocardial infarction with nonobstructive coronary arteries);
2. Other cardiac causes (e.g., myocarditis, Takotsubo cardiomyopathy);
3. Extracardiac causes.

##### 4.3.4.1 Investigation of cases of elevated troponin without coronary obstruction (troponin-positive nonobstructive coronary arteries – TINOCA or TpNOCA)

Approximately 5 to 15% of patients diagnosed with acute MI who undergo coronary angiography are found not to have with obstruction ≥ 50% stenosis on angiographic assessment.^88^Often, a patient who is referred for angiography does not have an established diagnosis of MI; this larger group that presents with myocardial injury but no obstructive CAD (i.e., with obstruction ≥ 50% stenosis) on angiography, regardless of etiology, is classified TINOCA,^88^which encompasses the MINOCA group when there is a confirmed diagnosis of acute MI. Recommendations for investigation of TINOCA are given in a flowchart ( Figure 15 ) and table of recommendations ( Table 29 ) below.

**Figure 15:** Diagnostic pathway for patients with acute MI and normal coronary angiography. PE: pulmonary embolism; CAD: coronary artery disease; IVUS: v; OCT: optical coherence tomography; MR: Magnetic Resonance.

**Table 29:** Further investigation of patients with myocardial injury and normal or nonobstructive (< 50% stenosis) coronary angiography

	Class of recommendation	Level of evidence
Confirm whether classification as TINOCA is indeed appropriate (review angiography images in search of obstruction, dissection, embolism, coronary thrombus; check other tests for differential diagnoses such as Takotsubo).	I	C
Request cardiac magnetic resonance imaging and other additional tests (intracoronary imaging and/or tests for coronary spasm or microvascular disease) according to clinical suspicion.	IIa	C

### 4.4. Rational Use of Noninvasive Tests

The rational selection of noninvasive methods for investigation of ACS is essential, given that the differential diagnosis of chest pain is broad and requires a structured approach for adequate, rapid distinction between cardiac and noncardiac causes, due to the mortality and morbidity associated with cardiovascular diseases. These tests can be performed while the patient is under observation in hospital or in an early outpatient setting, depending on patient risk and availability of resources.

During in-hospital observation, stress tests (exercise or pharmacological) should only be performed on stable patients in whom MI has been safely ruled out (the time needed to rule out MI varies depending on the time elapsed since onset of chest pain and the type of biomarker used, as described in sections 3.3, 4.1, 4.2, and 4.3).

In eligible patients with acute chest pain (or chest pain equivalents), noninvasive techniques are useful to rule out obstructive coronary artery disease, to stratify the risk of adverse outcomes, and to distinguish life-threatening cardiovascular diseases from low-risk conditions.

Several factors are important in deciding which modality to pursue, such as availability, local experience, type of clinical presentation, and, most importantly, the patient's characteristics and choices. In challenging cases, more than one method may be necessary; multimodality investigation is essential in these situations. In addition to general guidance on selection of noninvasive methods ( Figures 13 , 14 , and 15 ), aspects specific to each of these methods will be discussed in detail in this section to inform optimal decision-making for each clinical scenario (patient-centered recommendations). Finally, when mentioning diagnostic methods, reasoning should always be integrated with Bayes’ theorem, in which the post-test probability of an event or disease is conditional on its pretest probability.

#### 4.4.1. The Role of Exercise Electrocardiogram Testing

Exercise testing (ET) is a widely used functional method for diagnosis and risk stratification in various clinical scenarios, having accumulated a robust body of evidence over several decades. It has the advantages of being readily accessible, somewhat inexpensive, and not exposing the patient to ionizing radiation or contrast.

In the approach to acute chest pain in the emergency department, ET has been used since the early 1990s, both as an ancillary method to help rule out ACS as well as to stratify short- and medium-term risk of death, which increases the safety of hospital discharge.^144^Much of the evidence for this indication is based on observational studies.^146 , 146^What few randomized clinical trials have been performed have mostly been single-center with small sample sizes, lacking adequate statistical power to discriminate risk of death or nonfatal MI between groups.^147 – 149^

The process of stratifying patients into low-, intermediate-, or high-risk groups has sometimes been subjective, with failure to use established clinical prediction scores, which can hinder broader application of the findings of these studies.^149 , 150^

Their various methodological issues notwithstanding, these studies were all limited to patients with acute chest pain clinically suspected to be of ischemic origin. Non-ischemic etiologies of acute chest pain, as well as other conditions in which ET is contraindicated, are described in Table 30 .

**Table 30:** Clinical conditions in which exercise testing in the emergency department is not indicated

ET is contraindicated in:
Chest pain of clearly traumatic etiology
Acute aortic dissection
Acute pulmonary embolism
Pericarditis and myocarditis
Chest pain in the acutely febrile patient
Symptomatic severe aortic stenosis
Evidence of acute decompensated heart failure
Signs of poor peripheral perfusion
Uncontrolled arrhythmias and hypertension

HTN: hypertension; BP: blood pressure; ET: exercise test.

Serial electrocardiograms and biomarkers of myocardial injury must be normal or nondiagnostic of ACS. Baseline ECG changes that affect the diagnostic accuracy of ET for detection of myocardial ischemia are listed in Table 31 .

**Table 31:** Baseline electrocardiogram changes that limit the diagnostic accuracy of exercise testing for detection of myocardial ischemia

Limit the accuracy of ET:
Left bundle branch block
Left ventricular strain
Right ventricular strain
Artificial cardiac pacing
Ventricular pre-excitation
Atrial fibrillation and flutter
Baseline ST-segment depression ≥ 1 mm

ET: exercise testing.

Few studies have included a significant proportion of individuals over 65 years of age,^145 , 150 , 151^with the average age generally ranging between 50 and 60 years. Men have predominated in most samples, with proportions ranging from 40% to 75%.

The greatest advantages of ET in the emergency department are its excellent safety and its ability to rule out presence of critical coronary obstruction. However, its overall discriminatory capacity is low, considering the potential to test positive, negative, or inconclusive for myocardial ischemia.^150 – 158^

Very few studies have addressed specific subgroups. In a study that included only low-risk women with normal ECG and troponins, stress testing or coronary imaging did not improve prediction of acute myocardial infarction or revascularization within 30 days.^159^Individuals with known CAD and/or prior myocardial revascularization have not been analyzed in specific studies. However, it bears stressing that the indications for conventional ET are extremely limited in this group, which is affected by multiple comorbidities, baseline ECG changes, and widespread use of antianginal medication.^160^No compelling studies were found to support the indication of ET in acute chest pain associated with the use of stimulants such as cocaine and amphetamines.

A recent systematic review with meta-analysis^161^aimed to analyze the occurrence of major adverse cardiovascular events within 1 year after a negative cardiac examination, including stress testing. The authors concluded that patients with low- to intermediate-risk chest pain do not require repeat advanced testing if they present to the emergency department with chest pain, given the very low risk of events during this period. Given the diagnostic limitations of cardiac stress testing, this method has been used more to refine prognostic assessment and diagnostic probability than to rule in our rule out CAD.^143^Nonetheless, the information it provides can be very useful, especially in resource-limited settings ( Table 32 ).

**Table 32:** Indications for exercise testing in acute chest pain

Indications for exercise testing in acute chest pain	Class of recommendation	Level of evidence
Exercise stress testing can be recommended for individuals with acute chest pain who have clinical suspicion of myocardial ischemia, an intermediate probability of CAD (i.e., who have not been classified as having low or high probability of CAD), a normal ECG and negative biomarkers of myocardial injury, and who are eligible to perform physical exertion (ideally with concomitant imaging).	IIa	B
Enough time must have elapsed to safely rule out acute MI (this varies depending on the time since onset of chest pain and the type of biomarker used) before exercise testing is performed.	IIa	B
In patients presenting with acute chest pain of presumed ischemic origin and intermediate probability, admitted to the emergency department with a maximal exercise test negative for ischemia performed within the last 12 months, another test should be prioritized according to clinical suspicion (do not repeat exercise testing * ).	IIa	B

CAD: coronary artery disease; ECG: electrocardiogram.

* As long as there has been no relevant change in relation to the previous clinical picture.

#### 4.4.2. Role of Echocardiography in Assessment of the Patent with Chest Pain

A role for resting echocardiography in the assessment of patients with chest pain in the emergency department is supported by several published studies.^162 – 164^Early studies demonstrated the value of observing transient left ventricular regional wall motion abnormalities as accurate markers of myocardial ischemia, while more recent studies using myocardial contrast echocardiography have demonstrated that assessment of myocardial perfusion is useful in establishing the diagnosis and prognosis of chest pain of cardiac origin.^162 – 164^

Kalvaitis et al.^162^evaluated 957 patients who presented to the emergency room with chest pain of < 12 hours’ duration, an inconclusive ECG, and normal or slightly elevated troponin levels. Their pathophysiological rationale was the observation that most of the regional wall motion observed at rest derives from thickening of the most endocardial portion of the myocardium, and that ischemic events that affect the myocardium always begin in the subendocardial layer and progress toward the subepicardium, which is known as the wavefront phenomenon of myocardial necrosis. Thus, ischemic areas affecting as little as 20% to 30% of myocardial thickness are enough to cause significant changes in regional wall motion,^163^which persist for several hours after the original ischemic injury; indeed, wall motion abnormalities were demonstrated in 97% of patients with MI in this study. In this Kalvaitis study, among the 500 patients without regional wall thickening abnormalities, small infarctions that had gone undetected were confirmed in only 2 cases. Thus, at the end of 12 hours after onset of first precordial pain, the negative predictive value for the presence of MI in this population was 94%.^162^

On the other hand, wall motion abnormalities do not always denote infarction; they may reflect, for example, the effects of transient ischemia on the myocardium. Prolonged changes in ventricular wall motion have clearer prognostic repercussions. Rinkevich et al.^164^studied left ventricular regional function and perfusion in 1,017 patients with chest pain, a nondiagnostic ECG, and normal or slightly elevated troponins. Of these, 292 (28.7%) had events at a median follow-up of 7.7 months; MI occurred in 13.1%, unstable angina in 6.7%, heart failure in 3.7%, and death in 1.9%. Overall, 2% of those experiencing events required percutaneous coronary intervention and 1.3% required CABG. Among the 43 patients with no changes in regional function who had events, only 10 had MIs. When both regional function and perfusion were normal, the 1- and 2-year cardiac event rates were 9.8% and 12.6%. Most events were "soft" in nature (i.e., neither MI nor cardiac death); these rates increased, respectively, to 18.9% and 21% when regional function was normal but perfusion was abnormal, and to 40.1% and 48.8% when regional function was abnormal but perfusion was normal. Finally, these rates increased further to 64.7% (1 year) and 74.4% (2 years) when both regional wall motion and perfusion were abnormal.

When also seeking a diagnosis of unstable angina (in addition to MI) in patients with chest pain and nondiagnostic ECG, the sensitivity of echocardiography ranges from 40 to 90%, and its negative predictive value from 50 to 99%. In these patients, a normal echocardiogram does not appear to add significant diagnostic information beyond that already provided by the history and ECG.

There have been extensive studies on the use of dobutamine stress echocardiography for evaluation of a heterogeneous population with chest pain. Its sensitivity for the diagnosis of CAD or myocardial ischemia detected by other methods is 90%, with specificity ranging from 80 to 90% and a negative predictive value of 98% (the latter varying with pretest probability). It is a very useful method for defining prognosis and provides a measure of safety before discharging patients who require noninvasive risk stratification.

In addition to myocardial examination, echocardiography is very useful in analyzing the pericardium. Remember that the pericardium is made up of a visceral layer (composed of mesothelial cells adhered to the epicardium) and a parietal layer (a fibrous structure composed of collagen and elastin, with a thickness < 2 mm), separated by a space that normally contains 15 to 35 mL of serous fluid. In this scenario, transthoracic echocardiography is particularly important, first because it rules out the presence of regional wall motion abnormalities and second because it allows visualization of pericardial effusion, thickening, and the presence of inflammatory signs such as fibrinous strands or debris within the pericardial sac.

In suspected acute aortic syndrome (AAS), transthoracic echocardiography (TTE) can identify complications (e.g., aortic regurgitation, tamponade), but its diagnostic accuracy for AAS itself is limited (sensitivity: 78%–100% for type A, 31%-55% for type B). Transesophageal echocardiography (TEE) has > 90% accuracy and can be performed conveniently at bedside.

In pulmonary embolism (PE), echocardiography (especially TEE) allows identification of mobile thrombi in the right chambers of the heart and in the trunk and/or main branches of the pulmonary artery; however, these findings are only present in a minority of cases. Functional echocardiographic assessment and measurement of right ventricular dimensions are limited, which has led to variation in the literature on echocardiogram performance. In any event, abnormalities can only be identified in more severe cases (leading to right heart strain), while right ventricular overload can also be identified in the absence of pulmonary embolism (concomitant heart or lung disease). Given these aspects, echocardiography is of great value in risk stratification after a diagnosis of PE. From a diagnostic standpoint, echocardiography has different value in stable and unstable patients with suspected PE:

1. Hemodynamically stable patients: echocardiography can assist in the differential diagnosis of dyspnea and chest pain and can occasionally detect right heart strain in a patient who appears stable on clinical examination. The "60/60" sign (combination of a pulmonary artery acceleration time of < 60 ms and a peak systolic tricuspid valve gradient of < 60 mmHg) and McConnell sign (RV free wall hypokinesis with sparing of the RV "echocardiographic apex") also have diagnostic value, although they are uncommon in unselected cases of PE (10 to 20% of cases);
2. Hemodynamically unstable patients: echocardiography has higher diagnostic value in these cases, since the absence of signs of RV overload or dysfunction essentially rules out PE as the cause of hemodynamic instability (especially if Doppler ultrasonography of the lower limbs shows no evidence of deep vein thrombosis). In addition to being very useful in ruling out PE in the unstable patient, echocardiography may justify emergency reperfusion therapy in hemodynamically compromised patients with a high probability of PE who present with unequivocal signs of RV pressure overload, especially if there are more specific echocardiographic findings (60/60 sign, McConnell sign, or visible thrombus), when immediate CT angiography is not feasible and there is no other obvious cause for RV pressure overload. In this line, echocardiography can also be very useful in ruling out other causes of shock (such as tamponade, acute valve dysfunction, left ventricular dysfunction, aortic dissection, hypovolemia).

Thus, there are several recommendations for the use of echocardiography in the investigation of chest pain in the emergency department ( Table 33 ), especially for the diagnostic assessment of life-threatening cardiovascular diseases (ACS, pericarditis/tamponade, aortic dissection, and pulmonary embolism).

**Table 33:** Indications for echocardiography in acute chest pain

	Class of recommendation	Level of evidence
In patients presenting to the emergency department with chest pain, in whom ECG and troponin are inconclusive and suspicion persists, a resting transthoracic echocardiogram with or without myocardial contrast should be performed within 12 hours of chest pain onset to evaluate potential ischemic changes.	I	B
In patients presenting to the emergency department with chest pain in whom the possibility of myocardial necrosis and ischemia at rest has already been ruled out due to the absence of echocardiographic or electrocardiographic evidence of ischemia, cardiac stress testing (pharmacologic or exercise) to induce myocardial ischemia may be performed before hospital discharge if outpatient investigation would be considered unsafe.	I	B
Transthoracic echocardiography may be used for the diagnosis of acute pericarditis in patients presenting to the emergency department with chest pain and other suggestive findings, such as acute febrile illness (with or without a decline in general condition) and pleuritic chest pain, regardless of whether characteristic ST/T segment changes are present on resting ECG.	I	B

#### 4.4.3. Myocardial Perfusion Scintigraphy with Single-Photon Emission Computed Tomography (SPECT) and Positron Emission Tomography (PET-CT)

Among the functional tests that can be administered to patients with acute chest pain, two myocardial perfusion imaging modalities ("nuclear stress tests") stand out: myocardial perfusion scintigraphy with single-photon emission computed tomography (SPECT), which uses^99m^Tc-sestamibi as the radiotracer, and positron emission tomography (PET) with^82^Ru (rubidium) or^13^N-ammonia (still limited in Brazil). These myocardial perfusion tests, performed at rest alone or both at rest and after stress ("nuclear stress testing"), have been used safely and effectively in the evaluation of patients with chest pain in the emergency department, having been tested in multicenter randomized trials for this purpose.^79 , 80 , 165^The main characteristics of nuclear medicine studies in this setting are their high sensitivity for detection of myocardial ischemia and their excellent prognostic ability.

##### 4.4.3.1. Indications for the Use of Nuclear Medicine Tests

After the standard initial assessment of patients presenting to the emergency department with chest pain (history, physical examination, ECG, biomarkers of myocardial injury), a percentage of these individuals will still have substantial risk of coronary disease. These patients must undergo imaging to identify those with acute coronary syndrome and those at higher risk of adverse events. Like CT coronary angiography (CTA) for anatomical evaluation, imaging methods for functional evaluation are especially useful in identifying this group of patients.^79 , 80^

One of the most consistent indications for myocardial perfusion scintigraphy in acute chest pain is in patients without a known history of CAD and who are stratified as being at intermediate risk of CAD. In these cases, the use of stress and resting myocardial perfusion scintigraphy is well established as a useful, effective technique.^79 , 80^In a single-center study, myocardial perfusion scintigraphy in patients with acute chest pain had the same safety and effectiveness as CTA, with no differences in adverse outcomes over 40 months follow-up.^166^Other head-to-head comparisons of CTA and myocardial perfusion scintigraphy have also shown that the two techniques are comparable, and that anatomical assessment has the potential to increase invasive angiography and revascularization rates. Similarly, in the outpatient setting, there is also evidence supporting nuclear medicine as a good option.^167 , 168^In the CE-MARC 2 study (Clinical Evaluation of Magnetic Resonance Imaging in Coronary Heart Disease 2), functional testing with myocardial perfusion scintigraphy or cardiac MRI resulted in lower rates of unnecessary angiography, without a significant increase in major adverse coronary events.^167^In the PROMISE study, which included 10,000 symptomatic patients with suspected CAD, a CTA-first strategy did not improve clinical outcomes over a median follow-up of 2 years compared with functional testing.^168^

One technique which has been evaluated in a randomized trial, and which is quite useful when available, is to obtain a resting^99m^Tc-sestamibi scan during the episode of acute chest pain. Normal imaging improves emergency triage decision-making for patients with symptoms suggestive of acute cardiac ischemia with no abnormalities on initial ECG and reduces unnecessary admission of patients without acute ischemia, without reducing the rate of appropriate admission of patients with acute ischemia.^169^Use of this technique was able to rule out acute MI in 98% of patients presenting to an emergency department with chest pain.^170^Although it assesses ischemia rather than necrosis (infarction), the indication for this test tends to decrease with the broader use of early rule-out algorithms with high-sensitivity cardiac troponin.

Another robust clinical indication for stress myocardial perfusion imaging in patients with intermediate-risk chest pain in the emergency department is for those in whom initial anatomical stratification by CTA or invasive angiography was inconclusive. In these cases, stress myocardial perfusion scintigraphy or stress perfusion PET-CT is able to assess the functionality of the territory subtended by the coronary lesion and determine whether it is causing myocardial ischemia and symptoms.^79 , 80^

Another important indication for stress and rest myocardial perfusion scintigraphy with SPECT (single photon emission computed tomography) is the assessment of ischemia in patients with known CAD or in those already known to have a high coronary calcium load, whether demonstrated on a previous chest CT or by an elevated calcium score.

PET-CT, although still in its infancy in Brazil, has the great advantages of being able to measure coronary flow reserve, increasing sensitivity in relation to SPECT, and providing the additional possibility of evaluating microvascular coronary disease.^79 , 80^Recent studies have suggested that assessment of microvascular disease with cadmium-zinc-telluride (CZT)-SPECT detectors has prognostic value in ischemia with non-obstructive coronary artery disease (INOCA), potentially allowing stratification of these patients for prevention and early intervention.^171 – 174^This technique may be an alternative in scenarios where there is no access to PET-CT, allowing assessment of myocardial flow reserve and risk stratification of patients with chest pain.^171 , 172^

In summary, nuclear medicine studies are an important part of the workup of acute chest pain in the emergency room, allowing assessment of myocardial ischemia with or without obstructive epicardial disease ( Table 34 ).

**Table 34:** Recommendations for the use of nuclear medicine cardiac imaging techniques in patients presenting to the emergency department with acute chest pain

	Class of recommendation	Level of evidence
For intermediate-risk patients with acute chest pain and no known CAD, stress PET/SPECT imaging is useful for the diagnosis of myocardial ischemia.	I	B
For intermediate-risk patients with acute chest pain, either ongoing or within 2 hours of pain resolution, and with negative initial biomarkers of myocardial injury, administration of the radiotracer during chest pain is useful to rule out myocardial ischemia.	I	B
For intermediate-risk patients with acute chest pain and known CAD who present with new or worsening symptoms, stress PET/SPECT imaging may be considered to evaluate for myocardial ischemia.	IIa	B
For intermediate-risk patients with acute chest pain and no known CAD with inconclusive invasive or noninvasive angiography findings, stress PET/SPECT imaging may be useful for the diagnosis of myocardial ischemia.	IIa	C

CAD: coronary artery disease.

High-risk findings on myocardial perfusion imaging in patients with acute chest pain generally indicate a high likelihood of significant coronary artery disease or an impending major adverse cardiovascular event. Findings associated with worse prognosis include:

1. Extensive myocardial ischemia: perfusion defects affecting more than 10% of total myocardium or involving multiple coronary territories;
2. A decrease in left ventricular ejection fraction (LVEF) after stress by at least 10% compared to rest; and
3. Transient left ventricular dilation.

Any one of these findings generally suggests the presence of high-risk anatomical lesions and potential benefit from myocardial revascularization; angiographic evaluation of coronary anatomy is indicated.^79 , 80^

A novel and rapidly advancing development is the addition of coronary flow reserve assessment to functional nuclear cardiology studies. Coronary flow reserve (CFR) measured by perfusion PET-CT can play an important role in risk stratification of patients with chest pain in the emergency department. CFR is a measure of the ability of coronary arteries to dilate and increase blood flow in response to increased demand, such as during stress or exercise. It provides valuable information about the functional status of the coronary circulation and can help assess the severity and prognosis of CAD, as well as evaluate microvascular dysfunction. Importantly, reduced CFR is associated with a higher risk of future cardiovascular events, including myocardial infarction, heart failure, and cardiac death. It serves as an independent predictor of adverse outcomes, even in patients with normal coronary arteries or nonobstructive CAD. CFR may help identify higher-risk individuals who may benefit from more aggressive treatment strategies.^173 – 175^There is evidence that the availability of PET-MPI (myocardial perfusion imaging) for examination of emergency room patients was associated with an increase in the number of referrals for evaluation of patients with acute chest pain, as well as with a shorter length of hospital stay. Furthermore, the use of PET-CT was associated with a 40% reduction in additional testing compared with the use of SPECT in patients presenting to the emergency department with acute chest pain.^176^

Figure S8 illustrates the case of a patient with acute chest pain with a normal ECG and myocardial injury biomarkers on admission. An exercise myocardial perfusion scan demonstrated reversible myocardial perfusion defects in the right coronary artery territory corresponding to 20% of the myocardium. Coronary angiography subsequently demonstrated a 90% obstruction in the right coronary artery, which was treated with a drug-eluting stent.

##### 4.4.3.2. Special Considerations in Women with Acute Chest Pain

An additional point should be emphasized regarding functional investigation of CAD: women are more likely than men to have nonobstructive CAD. This should be taken into account so that the use of anatomical methods to assess the coronary arteries does not lead to inappropriate diagnoses. Nonobstructive CAD is associated with a higher risk of myocardial infarction and mortality compared with the absence of CAD.^177^For evaluation of patients with persistent chest pain and nonobstructive CAD, myocardial perfusion PET with CFR assessment can be used to diagnose microvascular dysfunction and improve risk stratification. Studies with CZT-SPECT detectors suggest that this technique may be a feasible alternative for settings in which PET-CT is unavailable or unattainable.

Regarding noncardiac diagnoses of chest pain, ventilation/perfusion (V/Q) scanning is a noninvasive method classically used to stratify the risk of pulmonary embolism by analyzing the number of affected lung segments, allowing the probability of PE to be classified as none (normal), low, moderate, or high. V/Q scan findings should be interpreted on the basis of pretest probability (Wells and Geneva scores). It is a very useful alternative modality for investigation of PE, especially when there are limitations to performing or analyzing CT angiography.

#### 4.4.4. Computed Tomography Angiography (CTA)

The use of CTA for noninvasive assessment of the lumen of the coronary arteries is well established in the literature. As demonstrated by several studies, CTA of the coronary arteries has excellent accuracy compared to conventional angiography for the diagnosis of stenosis in patients at low to moderate cardiovascular risk, with particular emphasis on its high negative predictive value ( Table 35 ).^178 – 184^

**Table 35:** Summary of multicenter trials on the accuracy of CTA in detecting coronary stenosis (≥ 50% luminal narrowing) in low- to intermediate-risk patients without a previous diagnosis of coronary artery disease

TRIAL	Sensitivity (%)	NPV (%)	Specificity (%)	PPV (%)
CATSCAN (Garcia et al., 2006). 7 countries, 11 centers^182^	94 (89-100)	98 (94-100)	51 (43-59)	28 (19-36)
NIMISCAD (Marano et al., 2009). 20 centers in Italy^183^	94 (89-97)	91 (85-95)	88 (81-93)	91 (86-95)
ACCURACY (Budoff et al., 2008). 16 centers in the USA^179^	95 (85-99)	99 (96-100)	83 (76-88)	64 (53-75)
CORE64 (Miller et al., 2008). 7 countries, 9 centers^184^	85 (79-90)	83 (75-89)	90 (83-94)	91 (86-95)
Meijboom et al. (2008). 3 centers in the Netherlands^180^	99 (98-100)	97 (94-100)	64 (55-73)	86 (82-90)

The use of CTA in the assessment of acute chest pain has been evaluated in several studies regarding safety, risk stratification, and reductions in cost and length of hospital stay. Prospective, controlled, randomized trials have evaluated its use in the context of low- to intermediate-risk patients presenting with chest pain to the emergency department when combined with a negative conventional troponin.^185^Three of these studies are worth highlighting:

The first is the multicenter Coronary Computed Tomographic Angiography for Systematic Triage of Acute Chest Pain Patients to Treatment (CT-STAT) trial, which randomized 699 patients with low-risk chest pain to stratification strategies using CTA or rest-stress myocardial perfusion imaging.^186^The strategy with CTA reduced the time to diagnosis by 54% and hospitalization costs by 38%, with no difference in the rate of adverse events, compared to the myocardial perfusion imaging strategy.

The second study was the multicenter Angiography for Safe Discharge of Patients with Possible Acute Coronary Syndromes (ACRIN-PA) trial, which had the primary objective of evaluating the safety of using CTA in the workup of patients with low- to intermediate-risk chest pain (TIMI RISK 0 to 2) compared with the traditional approach.^187^None of the patients with a normal CTA had a primary endpoint event (cardiac death or MI within the first 30 days after admission). Furthermore, patients in the CTA group had a higher rate of discharge from the emergency department (49.6% vs. 22.7%) and shorter hospital stay (18 hours vs. 24.8 hours; p < 0.0001), with no difference in the number of revascularizations or catheterizations.

The third study was ROMICAT II (Rule Out Myocardial Ischemia/Infarction using Computer Assisted Tomography), which evaluated the length of ED stay and hospital costs in similar groups of patients.^188^This study included 1000 patients with a mean age of 54 years, and found that length of hospital stay was significantly shorter in patients stratified for CTA when compared to the conventional workup group (23.2 ± 37.0 hours vs. 30.8 ± 28.0 hours; p = 0.0002). The time to rule out diagnosis of ACS was also shorter in the CTA group (17.2 ± 24.6 hours vs. 27.2 ± 19.5 hours; p < 0.0001). There was no between-group difference in safety endpoints. The CTA group had a significantly higher percentage of patients who were discharged directly from the emergency department (46.7% vs. 12.4%, p = 0.001). Use of other diagnostic tests was significantly higher in the CTA group (97% vs. 82%, p < 0.001). Nevertheless, despite the higher expenditure associated with a greater number of catheterizations and revascularizations, overall costs were very similar between the two groups due to shorter hospital stays in the CTA group (p = 0.65).

In summary, CTA is a safe strategy for assessment of patients with low- to intermediate-risk acute chest pain and a **negative conventional (non-high-sensitivity) troponin,** reducing the rate of admission, the length of hospital stay and, probably, costs as well. The impact on the number of invasive procedures and revascularization rate is still conflicting, although it certainly does not increase mortality.^189 , 190^

The HEART score for stratifying chest pain in the emergency department has gained widespread use and support in current guidelines. Some trials have evaluated a combination of the HEART score with CTA.^190 – 193^In an analysis of the major studies that used this tool for prediction of cardiovascular events at 30 days, when patients with a HEART score of 3 to 6 were directed to CTA, the latter had a sensitivity of 97.8%, specificity of 84.1%, and negative predictive value of 99.6% for diagnosing coronary stenosis > 50%.^193^Thus, combined use of the HEART score with CTA has been validated mainly for low- to intermediate-risk patients, with a high negative predictive value for cardiovascular events, especially in those stratified with a HEART score between 3 and 6.

There is a Class I recommendation (Level of Evidence: A) for CTA in the assessment of patients with low- to intermediate-risk acute chest pain, a nondiagnostic ECG, and conventional biomarkers of myocardial injury (i.e., not hs-cTn) below the 99th percentile ("negative").

CTA in the emergency department for patients with chest pain, elevated conventional troponin levels, and at least one clinical cardiovascular risk factor was evaluated in the VEREDICT-TRIAL.^194^This study assessed the ability of CTA to identify coronary stenosis ≥ 50% as compared to a conventional invasive angiography strategy, both in the early stage (within 12 hours of hospitalization) and later in the course (after 48-72 hours). The negative predictive value to identify lesions ≥ 50% was similar in both strategies (NPV 90.9% at 12h), and there was a high positive predictive value for multivessel disease. Therefore, the use of CTA in patients with elevated troponin levels had high accuracy to rule out significant coronary disease and was thus useful in the early clinical management of such patients in this study.

However, the adoption of this strategy has not been tested for clinical prognostic impact after discharge. The RAPID-CTCA TRIAL^195^tested a strategy of CTA vs. conventional management in patients with a diagnosis of NSTE-ACS (elevated conventional troponin). The data from this study show that the CTA strategy did not reduce the proposed primary outcome (all-cause mortality or type 1 or 4b AMI within 1 year), with an incidence of 5.8% for CTA vs. 6.1% for conventional treatment (p = 0.65), nor did it reduce the number of revascularizations (odds ratio 1.03, CI 0.87-1.21). However, it did reduce the number of cardiac catheterizations (odds ratio 0.81, CI 0.72-0.92), at the expense of a slight increase in length of stay from 2.0 to 2.2 days.

The use of hs-cTn in the emergency department is becoming increasingly popular, as they provide a measure of safety to discharge the patient when negative.^88^Few studies have evaluated the use of CTA in this context. The BEACON TRIAL,^191^a randomized multicenter study, evaluated the use of CTA in the ED in low- to intermediate-risk patients who, after a negative high-sensitivity troponin, were randomized to undergo CTA or receive the standard of care, with the primary outcome being the number of revascularizations at 30 days. In this scenario, CTA did not result in any differences in the number of revascularizations or undetected ACS, rate of discharge from the emergency department (65% vs 59%; p = 0.16), or length of hospital stay (6.3 hours in both groups). However, CTA did manage to reduce the costs of care (337 vs 511 euros, p < 0.01) and the percentage of patients requiring additional tests after discharge (4% vs 10%, p < 0.01).^174 , 188 – 195^

Therefore, when performed early in the workup of suspected ACS in the emergency department, CTA is safe and associated with fewer additional tests and lower costs, due to a reduced need for additional investigations in the outpatient setting after discharge. However, in patients with negative hs-cTn levels, coronary CT angiography did not identify more patients with significant CAD requiring CABG, nor did it shorten hospital stay or allow a higher proportion of patients to be discharged from the ED, when compared to a conventional strategy. On the other hand, the PRECISE-CCTA^196^randomized trial evaluated patients presenting to the ED with chest pain who had detectable levels of high-sensitivity troponin in the 5 ng/L to 14 ng/L range (considered borderline, but still within normal range and nondiagnostic for NSTEMI). These cases underwent CTA after discharge and were found to have a higher prevalence of both nonobstructive CAD (71.9% vs 43.4%; odds ratio 3.33) and obstructive CAD (29.9% vs 19.3%; odds ratio 1.79), regardless of their chest pain characteristics. Therefore, patients with chest pain (especially those with borderline hs-cTn) levels without a diagnosis of AMI may benefit from CTA after hospital discharge, as this provides them with the opportunity to receive effective treatment to prevent future CAD-related events in an outpatient setting. The TARGET-CCTA trial (NCT03952351) is ongoing to evaluate whether early treatment of CT-identified CAD in patients with intermediate elevations of high-sensitivity troponin has any impact on preventing future events 36 months after discharge.

Some studies have evaluated the use of coronary CT angiography in the context of frankly elevated high-sensitivity troponin levels. The CARMENTA trial^197^evaluated whether CTA or cardiac MR would be safe to better select those with patients positive high-sensitivity troponin who would benefit from cardiac catheterization for diagnosis of critical lesions compared to a conservative approach. It bears stressing that the study had some significant exclusion criteria, such as age over 85 years and dynamic St-segment changes. The study demonstrated that, in this context, use of CTA or cardiac MR to select patients with positive hs-cTn (low to moderate elevation) for invasive angiography was safe, with no increase in cardiovascular events when compared to a routine conservative approach. Therefore, the use of CTA in patients with elevation of high-sensitivity troponin to intermediate levels (study mean of 78 ng/ml) and no high-risk criteria safely reduced the need for cardiac catheterization when compared to the standard strategy (odds ratio 0.66, p < 0.001), with no increase in cardiovascular events after 1 year of follow-up (p = 0.265).

Studies have attempted to evaluate the use of calcium scoring as a way to predict coronary stenosis in the emergency department. Subgroup analyses of CORE 64^198^showed a low negative predictive value (NPV 0.62), with up to 39% of high-risk patients with ACS having a calcium score of zero and 46% having values below 100 Agatston units. Therefore, considering the current evidence, calcium scoring should not be used in the emergency department to predict significant coronary lesions.

##### 4.4.4.1. Triple Rule-out

CTA can also be used in the emergency department to support the differential diagnosis of ACS, especially in cases of suspected pulmonary embolism and acute aortic syndrome.

CT angiography with a "pulmonary embolism protocol" is the most widely used noninvasive imaging method for cases with an intermediate or high pretest probability of PE. It bears stressing that CTA has limitations in cases of embolism that affect only the smaller (subsegmental) pulmonary vessels.

When there is clinical suspicion of AAS, speed and accuracy in diagnostic confirmation are of the essence, since this condition has a high immediate mortality rate and definitive treatment is usually surgical. The decision to use a particular imaging method in AAS should be based not only on its diagnostic accuracy but also on its immediate availability and the experience of local emergency physicians and echocardiographers/radiologists with the method(s).

In addition to specific protocols for suspected pulmonary embolism, acute aortic syndrome, or acute coronary syndrome, some acquisition protocols can provide information related to the coronary circulation, aorta, and pulmonary arteries, allowing not only assessment of AAS and PE but also checking for other abnormalities in the chest cavity (pneumonia, trauma, etc.).^199 – 203^This approach is known as *triple rule-out* . However, even with optimized techniques, the triple rule-out acquisition protocol is less efficient than individual protocols for evaluating the coronary arteries, aorta, and pulmonary arteries. Therefore, triple-rule out protocols should be used only in specific situations in which clinical assessment is unable to direct the diagnostic workup.

In summary, CT angiography of the coronary arteries allows for the noninvasive assessment of epicardial obstructions and identification of atherosclerotic plaque with high accuracy, leading to a high negative predictive value for clinical investigation of patients without an established diagnosis of AMI ( Table 36 ).

**Table 36:** Indications for CT angiography of the coronary arteries in suspected acute coronary syndrome

	Class of recommendation	Level of evidence
Assessment of low- or intermediate-risk patients with suspected ACS with a normal or nondiagnostic ECG, normal biomarkers or myocardial injury, or abnormal biomarkers not meeting criteria for myocardial infarction * .	I	A
Assessment of patients with acute chest pain by the "triple rule-out" technique	IIb	B
Assessment of high-risk patients with suspected ACS.	III	C
Assessment of patients with an established definitive diagnosis of myocardial infarction.	III	C

ACS: acute coronary syndrome; ECG: electrocardiogram.

* If only a conventional (i.e., not high-sensitivity) troponin assay is available, CTA of negative cases reduces the rate of hospital admission and length of hospital stay. If high-sensitivity troponin is available, CTA helps elucidate cases with no definitive diagnosis, including those with "gray-zone" troponin levels (reducing the need for cardiac catheterization without increasing the rate of cardiovascular events at 1-year follow-up).

#### 4.4.5. Cardiovascular Magnetic Resonance Imaging

##### 4.4.5.1. Detection of Myocardial Ischemia

Currently, the presence of myocardial ischemia can be detected by cardiovascular magnetic resonance (CMR) using first-pass perfusion imaging with pharmacological stress or at rest, or by evaluation of contractility after induction of ischemia with dobutamine; the former is more sensitive and is preferred in the setting of chest pain, while the latter has greater specificity.^204 – 206^

Considering only the assessment of myocardial perfusion, some recent studies have given CMR pride of place in this scenario, both for diagnostic purposes and for prognostic prediction. The MR-INFORM study, published in 2019, compared different investigation strategies in 918 symptomatic patients and found that the CMR strategy was noninferior to a strategy using invasive CFR measurement (3.7% for CFR vs 3.6% for MRI), with no difference in primary outcomes at 12 months.^207^The SPINS study, also published in 2019, evaluated 2,349 patients with chest pain and found that absence of ischemia or late enhancement is associated with a low incidence of cardiovascular events within 5 years after CMR.^208^

CMR can also be used in patients with chest pain who have normal biomarkers and a nondiagnostic ECG, demonstrating 100% sensitivity and 93% specificity for detection of future cardiovascular events in this population.^204 – 209^

##### 4.4.5.2. Differential Diagnosis of Positive Troponin with Normal Coronary Arteries (TINOCA / MINOCA)

Although the diagnosis of acute MI is usually associated with the presence of coronary obstruction, we know that a substantial number of patients with ACS (between 6-8%) have angiographically normal coronary arteries.^190^The diagnosis of MINOCA requires documentation of an acute MI and invasive coronary angiography or CTA without significant obstruction.^56 , 77 , 88^

Just as for any other type of MI, the diagnosis of MINOCA requires that the myocardial injury be explained by an ischemic mechanism, and that nonischemic causes such as myocarditis or Takotsubo syndrome be ruled out.

Three characteristics need to be present for a diagnosis of MINOCA to be confirmed:^56 , 77 , 88^1) The same diagnostic criteria as for acute MI (consistent clinical picture with abnormal biomarkers of myocardial injury); 2) normal angiography or lesion; and 3) no other clinical cause may have been identified that could explain the findings consistent with myocardial injury (e.g., myocarditis or pulmonary embolism). Many researchers consider MINOCA, like "heart failure," to be a working diagnosis, as oftentimes it is difficult to identify the precise etiology for proper therapeutic disposition.^56 , 77 , 88^Therefore, some differential diagnoses may actually be potential etiologies, as they meet the characteristic criteria for type I or II MI. These include ischemic diseases resulting from coronary plaque (erosion, rupture, or ulceration), coronary dissection, thromboembolism, microvascular coronary spasm, coronary embolism, as well as inflammatory cardiomyopathies (myocarditis of any etiology), Takotsubo cardiomyopathy, and even pulmonary embolism. Therefore, accurate diagnosis of MINOCA is essential to allow selection of the optimal therapeutic option for ischemic and nonischemic patients.^56 , 77 , 88^Due to their diagnostic complexity, it has been suggested that these diagnoses be grouped under the term TINOCA (troponin-positive nonobstructive coronary arteries), as they all share elevated troponin levels as a common marker.

This umbrella term would then be subcategorized into ischemic causes (MINOCA), myocardial causes (e.g., myocarditis), and extracardiac causes (e.g., pulmonary embolism).^88^

Cardiac MRI is one of the most important tools for determining the etiology of TINOCA cases, and can define the cause in up to 74% of them.^209^Late enhancement, when present, allows localization of the area of myocardial injury, in addition to providing evidence of the mechanisms involved. Furthermore, MR allows identification of patients with a worse prognosis, and, when performed, changes management in approximately half of MINOCA cases.^204^

Thus, the use of a diagnostic resource so precise that it can detect infarctions with less than 1 gram of myocardial necrosis^205^allows true personalization of medical therapy (including secondary prevention), which can prevent new ischemic events and avoid unnecessary prescription of drugs with their respective side effects, such as bleeding (in the case of use of antiplatelet agents).^209^

Approximately 23% of cases diagnosed as MINOCA are due to coronary atherosclerosis in its nonobstructive form, resulting from erosion or ulceration of plaques with consequent transient thrombosis and recanalization of the compromised vessel, resulting from prolonged vasospasm.^206^Cardiac MR can confirm the diagnosis of infarction using the late enhancement technique, and discern ischemic causes by visualizing involvement of the subendocardium (a hallmark of ischemic etiology). Additionally, it allows differentiation from other lesions using techniques that present with edema (as in myocarditis), either through traditional T2-weighted techniques or through new parametric T1 and T2 mapping.^210^

Another relevant cause of TINOCA that is commonly confused with MINOCA is myocarditis. It corresponds to approximately 29% of such cases,^7^is most commonly of viral etiology, and has gained greater prominence in the wake of the COVID-19 pandemic, with approximately 50% of recovered cases exhibiting myocardial changes.^211 – 213^

Approximately 16% of MINOCA cases will present as Takotsubo cardiomyopathy^206^("broken heart syndrome"). Although initially recognized as "benign" due to the reversibility of dyskinetic areas, monitoring of the natural history of this syndrome over time has shown that its prognosis may be unfavorable.^214^Cardiac MR is an excellent diagnostic tool, as it allows identification of dyskinetic areas in any segment of the LV (although transient apical dyskinesia is the most frequent manifestation in Takotsubo syndrome). Furthermore, it allows characterization of areas of myocardial edema with T2-weighted techniques or, more recently, parametric T1 and T2 mapping, and was considered the method of choice in a recently published consensus statement.^215^The inflammatory phase, in which myocardial edema can be detected, usually disappears within 3 months.^216^In these cases, a control MRI may be ordered to verify reversal of the dyskinetic area and resolution of myocardial edema, which confirm the diagnosis. Late enhancement is not usually observed in this syndrome; however, in the acute phase, small islands of late enhancement can be seen in dyskinetic areas, due to the increased interstitial space in areas of inflammation.

Other possible diagnoses that may be encountered during investigation of TINOCA are less frequent, such as hypertrophic cardiomyopathy (approximately 3% of cases), nonischemic dilated cardiomyopathy (2% of cases), and amyloidosis (less than 5% of cases).^206 , 210 , 216 , 217^

Thus, its excellent performance in making an accurate diagnosis of infarcted areas related to MINOCA leads to a Class 1 recommendation (Level of Evidence: B) for cardiac magnetic resonance in this setting^200 , 202^( Table 37 ).

**Table 37:** Indications for assessment of myocardial ischemia by cardiac magnetic resonance imaging

	Class of recommendation	Level of evidence
Investigation of ischemic heart disease in patients with acute chest pain and intermediate pretest probability of CAD.	I	B
Investigation of myocardial ischemia in patients who have undergone surgical or percutaneous revascularization and present with symptoms suggestive of obstructive CAD.	I	B
Differential diagnosis of syndromes of troponin elevation with nonobstructive coronary arteries (TINOCA).	I	B
Assessment of patients with known nonobstructive CAD and no troponin elevation, but with suspected INOCA.	IIa	C

CAD: coronary artery disease.

* Defined as LMCA stenosis ≥ 50% or triple-vessel disease with proximal coronary involvement.

##### 4.4.5.3. Myocarditis

Myocarditis is a disease of inflammation of the heart muscle, which can occur as a result of infection, exposure to toxic substances, or activation of the immune system.^218^Viral infectious etiology is most prevalent. The clinical picture is highly variable clinical picture (ranging from completely asymptomatic to sudden death), but patients generally present with precordial pain, dyspnea, fatigue, palpitations, and syncope.^219^Various electrocardiographic changes are present in 85% of cases (e.g., ST-segment elevation, QRS widening, or arrhythmias), associated with elevated biomarkers of myocardial injury (hs-cTn).^218^

The diagnosis of myocarditis requires integration of clinical presentation, physical examination findings, laboratory tests, and imaging. Cardiac MR is helpful in diagnosis, as it is highly sensitive for tissue changes that occur due to myocardial inflammation.^220 , 221^The Lake Louise criteria were updated in 2018 to combine parametric mapping techniques and extracellular volume, increasing diagnostic accuracy. Acute myocardial inflammation can be detected if at least 1 criterion from each category is present.^220^One category is myocardial edema by T2-weighted imaging or T2 mapping; the other is myocardial injury by late enhancement, native T1 augmentation, or increased extracellular volume.^220 , 222^T2 values are higher in the acute phase of myocarditis and tend to normalize over a period of months, making T2 mapping a useful resource both for diagnosis and in monitoring treatment response.^223^The T1 relaxation time is prolonged by intracellular or extracellular edema, hyperemia, and presence of areas of fibrosis, and the extracellular volume may increase due to expansion of the extracellular medium by inflammation.^220 , 223^If criteria from both categories are positive, this increases the diagnostic specificity. On the other hand, if only one category is present in a patient with clinical suspicion, it only aids in the diagnosis.^220^In the absence of late enhancement and a clinical picture consistent with myocarditis, the presence of changes in native T1 mapping and extracellular volume may be indicative of myocardial injury. In this scenario, native T1 augmentation in areas without late enhancement was found to increase sensitivity without increasing the number of false positives.^216^

In addition to its diagnostic utility, cardiac MR can also be used for prognostic purposes. In this context, biventricular dysfunction resulting from significant myocardial involvement is the greatest predictor of mortality. The presence of late enhancement is also predictive of mortality, being associated with risk of sudden death and progression to left ventricular enlargement and decline in LVEF.^216^Patients with EF ≤ 40% associated with late enhancement have a 10% year-on-year increase in their risk of an adverse cardiovascular event.^216^

SARS-CoV-2, the virus that causes COVID-19, has been frequently associated with myocardial injury. Troponin elevation above the 99th percentile is present in 62% of patients with COVID-19. During follow-up of these patients, the most common finding is diastolic dysfunction (55%); only 2.8% had reduced LVEF.^224^In the acute phase of COVID-19, the most common cardiac MR findings are changes in T1 and T2 mapping, pericardial changes (myopericarditis), and non-coronary late enhancement patterns. Mild or asymptomatic cases do not show significant changes compared to controls.^224^

Currently, there is a Class I recommendation for CMR to be performed in suspected myocarditis, both in international guidelines and in the Brazilian SBC guideline;^92 , 225^the present document corroborates this recommendation ( Table 38 ). The recent incorporation of parametric T1 and T2 mapping and extracellular volume data increases the sensitivity of CMR.

**Table 38:** Indications for CMR in myocarditis

	Class of recommendation	Level of evidence
Assessment of ventricular function, geometry, and morphology in suspected acute, subacute, and chronic myocarditis.	I	B
Diagnostic and prognostic investigation of acute, chronic, and/or suspected previous myocarditis.	I	B
At 4-week to 12-week follow-up of an acute episode of myocarditis, to distinguish complicated from uncomplicated disease.	IIa	B
In fulminant myocarditis with hemodynamic instability.	III	B

## 5. A Model for Implementation of Chest Pain Units

### 5.1. Investigation of Acute Coronary Syndrome in the Prehospital Care Setting

According to the Fourth Universal Definition of Acute Myocardial Infarction, there are no conceptual differences in diagnosis between the hospital and prehospital settings.^77^

Major challenges in the prehospital setting include agility in diagnosis and institution of therapy and the unavailability of some helpful diagnostic tests, since in Brazil such patients are mainly treated in freestanding emergency departments (FSEDs) or Advanced Life Support (ALS) ambulances operated by emergency medical services (EMS) (e.g., SAMU ambulance). Accuracy and speed of diagnosis are priority goals of prehospital assessment, in order to optimize distribution of EMS resources and ensure more assertive transport/transfer of patients to definitive care ( Table 39 ).

**Table 39:** Investigation of acute coronary syndrome in the prehospital care setting

	Class of recommendation	Level of evidence
Diagnostic criteria are the same in the prehospital setting (e.g., ambulance) as in the in.hospital setting (e.g., emergency department).	I	C
Prehospital care systems must have protocols in place to identify cases meeting suspected ACS criteria and deploy specialized mobile units (with ECG capabilities, a physician, and Advanced Life Support resources).	I	B
An electrocardiogram must be performed within 10 minutes of first medical contact, whether at a hospital or in the prehospital setting.	I	B
Clinical prediction scores such as the preHEART score may be useful additional tools for prehospital risk stratification.	IIa	B
Prehospital point-of-care troponin testing may be considered when available for cases presenting with a nondiagnostic ECG, especially those with a low clinical probability of ACS and within the adequate time window for biomarker measurement.	IIb	B

ACS: acute coronary syndrome; ECG: electrocardiogram.

#### 5.1.1. Patient Perceptions and Organization of the Prehospital Care Network

The aphorism "time is muscle" remains relevant in the treatment of acute MI.^226^Since the first trials of thrombolysis showing that the shorter the time to reperfusion, the greater the mortality benefit, agility has been sought from the patient's perception of symptoms to the reperfusion strategy.^227^Public campaigns increase patient awareness of MI symptoms and increase emergency care-seeking by AMI patients (including increased calls to EMS), which ultimately reduces prehospital wait times ( Figure 16 ).^228^It is worth noting that women tend to experience longer times between first perception of symptoms and prehospital activation as compared to men, with higher in-hospital mortality rates, highlighting the importance of this specific population.^229^Implementing an EMS system to care for such patients has led to reductions in the overall and in-hospital mortality rates in the regions covered.^230^Also worth noting are the *Acute Myocardial Infarction Care Pathways,* which have been incorporated into the Brazilian Unified Health System and had ordinances passed encouraging their use.^231^

**Figure 16:** Chest pain: early care saves lives. (Public awareness material to improve patient perception of symptoms).

#### 5.1.2. Role of ECG in the Prehospital Setting

Just as in the hospital environment, ECG must be performed within 10 minutes of first medical contact in the prehospital setting as well. Every 30-min delay in reperfusion is associated with a 7.5% increase in mortality in these patients.^91^A meta-analysis of more than 80 publications found that the use of prehospital ECG resulted in a lower number of deaths, shorter time to reperfusion, shorter hospital stay, and higher proportion of patients who achieved a door-to-FMC-to-reperfusion time < 90 min.^232^It is worth noting there is evidence from several studies that telemedicine tools (e.g., remote ECG) are beneficial to the care of these patients, including a reduction in cardiovascular events.^232 – 236^

#### 5.1.3. Role of Troponin in the Prehospital Setting

Troponin measurement is essential for diagnosis and risk stratification of patients who do not meet ECG criteria for NSTE-ACS. In-hospital troponin testing is well-established practice, but more robust evidence is needed to support its use in the prehospital setting.

Several studies have evaluated its use by ambulance crews to screen patients before transfer to designated heart attack centers. Overall, these were small studies that included patients with chest pain and used point-of-care troponin testing (POC cTn) in conjunction with the modified HEART score to rule out ACS in low-risk patients.^237 – 239^In 2023, Camaro et al. published the ARTICA study,^240^demonstrating that a prehospital POC cTn strategy resulted in lower healthcare costs (primary endpoint) with a comparable rate of major adverse cardiovascular events (secondary endpoint) as compared to a strategy of direct transfer to the ED without prehospital troponin measurement.

#### 5.1.4. Role of Risk Stratification Scores

Clinical history, physical examination, and ECG essentially constitute the standard of care of the chest pain patient in the prehospital setting, seeking to identify cases of STEMI and trigger reperfusion strategies. However, in the absence of ST elevation (or STEMI equivalents), there is no validated risk stratification strategy to screen patients for direct transfer to primary PCI centers versus transport to lower-complexity facilities. Sagel et al., in a recent publication assessing use of the HEART score and POC cTn for stratification of these patients, proposed a new *preHEART score* as a tool for risk stratification specifically in the prehospital setting, with good accuracy compared to the other methods (NPV 99.3% [98.1-99.8], PPV 49.9% [42-56.9], AUC 0.82 [0.82-0.88]).^241^Mirroring hospital practice, it is generally advocated that a combination of tests be used for prehospital risk stratification; in this setting, the preHEART score has emerged as a promising alternative.^242^

#### 5.1.5. Other Diagnostic Tools

Few studies have used transthoracic echocardiogram or POCUS as a diagnostic aid for ACS in the prehospital setting. One study which suggested its potential utility has been criticized for its methodological flaws, mainly regarding the accuracy of the method and the limited training of examiners.^243 , 244^The European Society for Cardiovascular Imaging nevertheless recommends the use of emergency echocardiography in patients with acute chest pain suggestive of ACS, but robust studies and standardization of methods for its use in the prehospital setting are needed before it can be incorporated into practice.^243 – 248^It should be remembered that, in addition to ACS, other diagnoses such as aortic dissection and PE should also be considered (scores, point-of-care D-dimer testing, and POCUS itself can help guide referral to the facility with the most appropriate resources according to the diagnostic suspicion).

### 5.2. A Model for Implementation of Chest Pain Units: Evidence for Best Practice

The approach to the patient with chest pain places emphasis not only on recognizing the diagnosis, but rather on identifying and promptly managing the most serious and potentially life-threatening conditions. This challenging task falls to the emergency physician and emergency department staff, who are invariably overburdened and often have scarce resources at their disposal. In this context, in recent decades, emphasis has focused on strategies to assist in the rapid, accurate discrimination of patients at high risk of developing complications from those at low risk who do not need to stay in what is considered the cornerstone of management of acute chest pain: the chest pain unit (CPU).^8 , 21 – 23 , 246^

The initial assessment of every patient with chest pain should allow for three key questions to be answered: (1) How likely are the signs and symptoms to be due to ACS? (2) What are the patient's odds of developing a major adverse cardiac event, such as myocardial infarction, stroke, heart failure, recurrent symptoms of ischemia, or serious arrhythmias? and (3) How can additional resources be optimized to support diagnostic and patient disposition decisions? The clinical history, electrocardiogram, and biomarkers of myocardial injury are usually sufficient to answer these questions in most cases. However, studies have shown that the accuracy, efficiency, and agility of this model in the absence of structured protocols and routines are associated with a non-negligible risk of mismanagement, misdiagnosis, and high costs.

CPUs became widespread in the early 2000s, with the aim of providing high-quality, rapid care for patients with chest pain with a low likelihood of ACS, but not low enough to allow hospital discharge. CPUs are defined as short-stay units organized so as to facilitate and optimize care through diagnostic protocols.^8 , 21 – 23^

Several observational studies and randomized clinical trials have reported that CPU care provides greater adherence to guidelines and evidence-based practices, with clinical outcomes similar to those of admission to conventional wards, but with shorter lengths of stay and reduced costs. Data from Australia, the United States, and Germany suggest that process improvements resulting from the adoption of CPUs have led to reduced length of stay, fewer hospital admissions, and greater use of noninvasive tests, resulting in 20–25% cost savings for healthcare systems. Several international and Brazilian centers have implemented CPUs with shared organizational and logistic characteristics, which have evolved over the years.^8 , 21 – 23^ Table 40 provides recommendations for the implementation of dedicated units for the assessment of patients with acute chest pain (or chest pain equivalents).

**Table 40:** Recommendations for the implementation of Chest Pain Units

	Class of recommendation	Level of evidence
Chest pain units should be implemented in high-volume emergency departments and/or cardiovascular referral centers.	I	A
Chest pain units should systematically collect quality indicators and conduct continuous assessment of improvement based on the metrics identified.	I	A
Chest pain units must be staffed by physicians and support providers trained to follow protocols and interpret ECGs.	I	C
Chest pain units must be capable of performing ECG and measuring biomarkers of myocardial injury (preferably high-sensitivity cardiac troponin).	I	A
Chest pain units must have access to STAT echocardiography.	I	C
Chest pain units must have access to a 24/7 catheterization laboratory, whether at the same facility or as part of an integrated emergency referral system.	I	C
Chest pain units must have diagnostic and treatment pathways in place for all major life-threatening acute chest pain syndromes.	I	C
Chest pain units must have access to noninvasive methods for investigation of coronary artery disease and/or ischemia.	IIa	C

ECG: electrocardiogram.

#### 5.2.1. Patient Assessment

Obtaining a brief, focused clinical history is essential in the initial assessment of patients with chest pain. Pain characteristics, presence of comorbidities, aggravating factors, and abnormalities on physical examination allow identification of immediate clinical risk.^97 – 107^The HEART score has proved to be easy, useful, and effective for risk stratification of patients with no established diagnosis.^97 – 101^Based on the patient's history, ECG, age, cardiovascular risk factors, and troponin values, a score between 0 and 10 is calculated, representing the patient's risk of developing a major adverse cardiac event within 6 weeks of initial presentation. A meta-analysis of 16 prospective cohort studies obtained a sensitivity and specificity of the HEART score for predicting major cardiac events of 0.96 (95% CI: 0.91-0.98; I^2^= 94.87%) and 0.50 (95% CI: 0.41-0.60; I^2^= 98.84%), respectively. However, the high heterogeneity among the included studies was a limiting factor for the interpretability and validity of this meta-analysis.^247^The combination of a HEART score < 4 and normal troponins has a high negative predictive value (99%), suggesting these patients can be safely discharged. Several studies have shown that the use of clinical pathways such as the Heart Pathway, ADAPT, and EDACS reduced the need for hospitalization by 20-45% in patients with suspected ACS. Other well-known scores, such as TIMI and GRACE, may also be useful for risk stratification of patients undergoing diagnostic assessment for suspected ACS, although they were developed and validated as prognostic instruments for the population already diagnosed with ACS and have inferior performance compared to HEART in patients still on the diagnostic pathway.^97 – 107 , 247^

#### 5.2.2. Organizational Structure and Human Resources

Traditionally, CPUs are designated areas near or within hospital-based or freestanding emergency departments, with integration between emergency physicians, cardiologists, and dedicated multidisciplinary teams.^248 , 249^In an ideal scenario, CPUs are supervised by cardiologists, while routine care is provided by internists and/or emergency physicians. From a management perspective, the number of beds in a CPU should be calculated based on the size of the hospital to which it is attached and the number of ED visits per year. It is estimated that, of every 10,000 monthly ED visits, 250-500 will be for chest pain (depending on whether the hospital is a designated referral center) and half of these would be eligible for CPU admission, i.e., 2 to 4 beds would be required.^250^European recommendations suggest one physician per unit and one nurse for every 4 to 6 beds; Brazil legislation calls for similar staffing levels, depending on the level of care provided and using a 1:3 ratio of nurses to nursing technicians. In general, CPUs have 2 to 6 dedicated beds, all of which may be monitored. Access to a cardiac catheterization suite and invasive procedures is important for cases in which the diagnosis of ACS is ultimately confirmed. For facilities lacking a 24/7 cath lab, cooperation agreements with other centers and protocols for transfer thereto must be in place for critical cases.

When establishing a CPU, one critical aspect is the availability of written and validated clinical protocols for all providers who will be involved in the care of these patients. These protocols must be updated periodically and management mechanisms must be implemented to ensure their effective use, as well as monitoring of indicators to inform continuous improvement. A continuing education program should be offered to providers, including validation and review of institutional protocols and training in ECG interpretation, biomarkers, and noninvasive methods.

#### 5.2.3. Technical Requirements

All CPUs must have a 12-lead ECG available for prompt performance and repetition whenever necessary. Ideally, the ECG should be obtained within the first 10 minutes after the patient's arrival at the hospital, starting from the moment the triage team identifies a suspected ACS case. Units must be equipped with a noninvasive blood pressure monitoring device for each patient, continuous ECG monitoring, a defibrillator, and cardiopulmonary resuscitation equipment. A telemetry monitoring center or central station is not absolutely essential, although it is desirable. Continuous cardiac rhythm monitoring is indicated for patients with suspected ACS or at high risk of cardiovascular events. Continuous ST-segment monitoring allows identification of patients with dynamic ischemia; however, studies have not shown significant additional value in lower-risk patients, and this method is currently considered optional in CPUs. Measurement of vital signs every 15-30 minutes is recommended for this patient profile.

Measurement of cardiac troponin (T or I) for detection of myocardial injury is recommended for all patients with suspected ACS. Some studies tested assessment protocols with sample collection at 0 and 3 hours, while others used 0/1-hour and 0/2-hour intervals. Serial troponin testing is contingent on a 24-hour clinical laboratory being available in the emergency department, ideally one capable of a < 60-minute turnaround for test results. For facilities without an on-site laboratory, rapid point-of-care testing can be considered as an alternative so as not to delay patient assessment and disposition.

In addition to these tests, a CPU must be capable of performing additional blood chemistry tests, plain chest radiography, and alternative imaging methods to refine the differential diagnosis of chest pain. CTA has been shown to be an excellent method to rule out obstructive coronary disease, especially in intermediate- or low-risk groups.^251^

Multislice CT should be available for further investigation of relevant differential diagnoses (such as PE and AAS) once ACS has been ruled out, or to rule out CAD in patients with low or intermediate probability.

For patients with intermediate or low risk of coronary complications, stress testing has become accepted practice and is available at most centers.^252 , 253^This recommendation is based on the value of this test for refining diagnostic probability and on the corresponding prognostic information it provides. However, considering the low prevalence of CAD in this group of low- and intermediate-risk patients, the likelihood of false-positive results is high. Therefore, current protocols no longer consider this an essential component of the chest pain workup. Supplemental noninvasive methods for diagnosis of ischemia – myocardial perfusion imaging, stress echocardiography, and CMR – provide additional information and help identify patients with CAD, and can be performed in the CPU or on an outpatient basis after discharge.

#### 5.2.4. Specific Therapies

Patients with suspected ACS but no established diagnosis should initially be treated with aspirin alone (in the absence of contraindications or risk of bleeding) until the case is elucidated. If PE is suspected, preemptive anticoagulation should be considered in patients with a high pretest probability as determined by the Wells and/or Geneva score. Medications may also be administered as needed for symptom relief and hemodynamic stabilization.

Once a diagnosis is identified (e.g., STEMI, NSTE-ACS, AAS, PE, etc.), the specific protocol for its management should be followed. Low-risk patients with negative results on all tests and no definitive diagnosis may be discharged from the CPU to early outpatient investigation.

#### 5.2.5. Management Indicators

CPU participation in local and national registries should be encouraged to allow prospective collection of indicators of care quality, adherence to evidence-based practices, and performance.^249 , 250^Some internationally accepted indicators are usually employed to monitor the performance of these units; however, each institution should implement its own set of indicators as dictated by the availability of information or opportunities for improvement. Common indicators include (for both chest pain and confirmed ACS):

- Time domain:
  ➢ Door-to-ECG time (ideally within 10 minutes);
  ➢ Door-to-needle or door-to-balloon time (for STEMI);
  ➢ CPU length of stay;
  ➢ CPU boarding time until transfer to intensive care or inpatient ward.
- Dual antiplatelet therapy prescribed at discharge and aspirin prescribed on arrival;
- High-intensity statins prescribed at hospital discharge;
- Patient perception of care received.

Other indicators may be added in specific situations; e.g., a patient who develops heart failure (HF) must be discharged on HF therapy.

It is considered good practice to issue reports at regular intervals (e.g., quarterly), the results of which should be documented in team meetings and case conferences. Feedback mechanisms should also be implemented to reflect the results and the quality of patient care in the CPU.

#### 5.2.6. Technological Advances

Many studies have shown that the care and risk stratification of patients with chest pain can be further improved, so that patients are stratified earlier, with greater safety, and with faster access to therapy for those who need it. Use of wearable ECG devices, point-of-care troponin measurement, and prehospital use of the HEART score are examples of interventions that can direct low-risk patients to further outpatient investigation or send high-risk patients directly to cardiology referral centers with cardiac catheterization and intensive care capabilities. Systematized algorithms are increasingly being integrated with patient records to reduce errors and speed up patient care processes, often with the support of artificial intelligence.^254 , 255^

Finally, the entire content of this guideline ( Figure 17 ) will only be fully implemented, and continuous improvement will only be possible, if local protocols are established with appropriate metrics and monitoring by the care team ( Table 40 ).

**Figure 17:** 10 Key Messages from the Chest Pain Care Guideline in the Emergency Department.
